# Development of the TSR-based computational method to investigate spike and monoclonal antibody interactions

**DOI:** 10.3389/fchem.2025.1395374

**Published:** 2025-03-19

**Authors:** Tarikul I. Milon, Titli Sarkar, Yixin Chen, Jordan M. Grider, Feng Chen, Jun-Yuan Ji, Seetharama D. Jois, Konstantin G. Kousoulas, Vijay Raghavan, Wu Xu

**Affiliations:** ^1^ Department of Chemistry, University of Louisiana at Lafayette, Lafayette, LA, United States; ^2^ The Center for Advanced Computer Studies, University of Louisiana at Lafayette, Lafayette, LA, United States; ^3^ Department of Computer and Information Science, The University of Mississippi, University, MS, United States; ^4^ High Performance Computing, 329 Frey Computing Services Center, Louisiana State University, Baton Rouge, LA, United States; ^5^ Department of Biochemistry and Molecular Biology, Tulane University School of Medicine, Louisiana Cancer Research Center, New Orleans, LA, United States; ^6^ Department of Pathobiological Sciences, LSU School of Veterinary Medicine, Louisiana State University, Baton Rouge, LA, United States

**Keywords:** TSR-based method, molecular interaction, monoclonal antibody, spike, amino acid structure, machine learning, TSR-STRSUM

## Abstract

**Introduction:**

Monoclonal antibody (mAb) drug treatments have proven effective in reducing COVID-19-related hospitalizations or fatalities, particularly among high-risk patients. Numerous experimental studies have explored the structures of spike proteins and their complexes with ACE2 or mAbs. These 3D structures provide crucial insights into the interactions between spike proteins and ACE2 or mAb, forming a basis for the development of diagnostic tools and therapeutics. However, the field of computational biology has faced substantial challenges due to the lack of methods for precise protein structural comparisons and accurate prediction of molecular interactions. In our previous studies, we introduced the Triangular Spatial Relationship (TSR)-based algorithm, which represents a protein’s 3D structure using a vector of integers (keys). These earlier studies, however, were limited to individual proteins.

**Purpose:**

This study introduces new extensions of the TSR-based algorithm, enhancing its ability to study interactions between two molecules. We apply these extensions to gain a mechanistic understanding of spike - mAb interactions.

**Method:**

We expanded the basic TSR method in three novel ways: (1) TSR keys encompassing all atoms, (2) cross keys for interactions between two molecules, and (3) intra-residual keys for amino acids. This TSR-based representation of 3D structures offers a unique advantage by simplifying the search for similar substructures within structural datasets.

**Results:**

The study’s key findings include: (i) The method effectively quantified and interpreted conformational changes and steric effects using the newly introduced TSR keys. (ii) Six clusters for CDRH3 and three clusters for CDRL3 were identified using all-atom keys. (iii) We constructed the TSR-STRSUM (TSR-STRucture SUbstitution Matrix), a matrix that represents pairwise similarities between amino acid structures, providing valuable applications in protein sequence and structure comparison. (iv) Intra-residual keys revealed two distinct Tyr clusters characterized by specific triangle geometries.

**Conclusion:**

This study presents an advanced computational approach that not only quantifies and interprets conformational changes in protein backbones, entire structures, or individual amino acids, but also facilitates the search for substructures induced by molecular binding across protein datasets. In some instances, a direct correlation between structures and functions was successfully established.

## 1 Introduction

Proteins are fundamental to sustaining life, requiring specific 3D structures to perform their biological functions. Among the pivotal elements driving various processes are noncovalent interactions, including protein - protein, protein - DNA/RNA, ligand - receptor, drug - target, or host - guest interactions. Structural and experimental methods such as X-ray crystallography, NMR or Cryo-EM techniques are employed to determine these molecular interactions. Additionally, laboratory-based functional assays, including yeast-two hybrid, pulldown experiment, or FRET method, can provide evidence of these interactions. Despite these methods, predicting molecular interactions computationally remains a formidable challenge. Physics-based methods for calculating binding free energy and empirical scoring functions for assessing binding affinities have been used to study protein - protein interactions (PPIs) (Massova and Kollman, 1999; Henrich et al., 2010). However, the lack of a mechanistic understanding of molecular interactions hinders the identification of binding partners and the elucidation of their regulatory mechanisms (Viswanathan et al., 2019).

Protein structures are intricately specified within their amino acid sequences. Sequence and structure comparison techniques benefit from the ever-expanding repositories of sequence and structure databases (Henrich et al., 2010). Yet despite decades of dedicated efforts, the mechanisms underlying molecular interactions remain elusive (Lupas et al., 2021; Marsh and Teichmann, 2015). Lack of accurate protein structural comparison and the precise prediction of molecular interactions remain as pivotal and enduring obstacles in the field of computational biology (Yang et al., 2020). To surmount the obstacle of precisely predicting PPIs, the field must confront two key challenges. The first challenge stems from the absence of a mechanistic understanding of a binding surface, leaving us uncertain about the distinguishing characteristics of a protein - protein binding site compared to the rest of the surface. This is manifested in the relatively low accuracy attained by computational methods, often employing various machine learning (ML) techniques, in predicting protein interfaces (Viswanathan et al., 2019). The second challenge lies in the dearth of mechanistic insights into conformational changes that occur when a molecule binds with its partner. Innovation in methods for representing protein 3D structures is critical for a mechanistic understanding of their functions. The development of computational methods capable of discerning binding surface from the rest of the surface, capturing subtle alterations at the residue level interactions, and tracing their propagation over local and global distances to predict conformational changes, which is the focus of this study, will have a profound impact on enhancing our understanding of PPIs.

The Triangular Spatial Relationship (TSR)-based method was developed for comparing protein 3D structures (Kondra et al., 2021). The input data for the TSR-based methods are experimentally determined 3D structures from the Protein Data Bank (PDB) (Bernstein et al., 1977). Structural data is an essential asset in understanding the mechanisms of protein functions (Abali et al., 2024). A TSR key is computed using the length, angle and vertex labels based on a rule-based label-assignment formula, which ensures the assignment of the same key to identical TSRs across different proteins. The features of the method include (i) A unique approach to representing molecular 3D structures, eliminating the need for structural superimposition or alignment (Kondra et al., 2021), (ii) Accurate quantification of structural similarity, either globally or locally, by counting the common TSR keys between two proteins (Kondra et al., 2021), (iii) The ability to search for similar structural motifs [e.g., drug binding sites (Kondra et al., 2021; Sarkar et al., 2021), metal binding sites (Sarkar et al., 2022), active sites (Kondra et al., 2021; Sarkar et al., 2021; Kondra et al., 2022), linking TSR keys to protein functions (Xu et al., 2020; Luo et al., 2023)] within protein structures, (iv) The utilization of different types of TSR keys to provide a unique way to interpret hierarchical relationships and distinctions between structures and sequences (Kondra et al., 2022), as well as the stereospecific properties of molecular binding or biochemical reactions (Sarkar et al., 2023).

The comparison of two protein structures, despite its apparent simplicity, is a non-trivial challenge. The TSR algorithm simplifies this complex problem to one of matching two integer vectors. This is the first step towards addressing the aforementioned challenges. Another advantage of this approach is that its adoption of an integer-based data structure allows the deployment of ML-based algorithms from the field of artificial intelligence (AI), for prediction purposes. To facilitate this study, focused on interaction between two molecules, we have created a curated dataset containing the annotated Third Complementarity Determining Region (CDR3) of heavy chain (CDRH3) and CDR3 of light chain (CDRL3) of spike monoclonal antibodies (mAbs). Additionally, we have developed new versions of the TSR-based method with a specific focus on probing interactions between spike proteins and their corresponding mAbs.

The COVID-19 pandemic was caused by the widespread infection with Severe Acute Respiratory Syndrome CoronaVirus 2 (SARS-CoV-2) (Hwang et al., 2022). SARS-CoV-2 is classified as a single-stranded RNA virus within the *Betacoronavirus* genus. The distribution of the virus-targeted receptor protein, angiotensin converting enzyme II (ACE2), determines which organs are susceptible to attack by SARS‐CoV‐2. Notably, ACE2 is highly expressed in several vital organs, including the lungs, immune system, heart, kidneys, esophagus, and small intestine (Chen et al., 2020).

The first critical step in the infection process occurs when the viral spike (S) glycoprotein binds to a host cell receptor, which can be either ACE2 (Zhou et al., 2020; Wrapp et al., 2020; Walls et al., 2020) or cluster of differentiation 147 (CD147) (Wang et al., 2020; Behl et al., 2022). Following the initial binding event, facilitated by the interaction with the receptor binding domain (RBD) of S and ACE2, host proteases, including furin (Peacock et al., 2021), transmembrane serine protease 2 (TMPRSS2) (Matsuyama et al., 2020), and cathepsin L (Simmons et al., 2005), cleave the head of S protein, transforming it into a spring-like structure. This structural change enables the viral membrane to fuse with the host membrane, facilitating entry into the host cell. This entry can occur through direct cell surface entry or endocytosis into endosomes (Matsuyama et al., 2020).

The RBDs of the spike protein display a dynamic hinge-like conformational equilibrium, shifting between a closed pre-fusion state (down conformation) and an open fusion-prone state (up conformation) (Wrapp et al., 2020; Shang et al., 2020; Henderson et al., 2020). mAb drugs, the fastest growing class of drugs on the market (Loomis et al., 2024), that are able to bind to and “neutralize” the virus in infected patients represent a novel class of antibodies for antiviral intervention (Renn et al., 2020). These mAbs, termed “neutralizing,” can be used as passive immunotherapy to minimize virulence (Taylor et al., 2021) as they prevent the virus from binding to ACE2 on the surface of human cells. This is a critical step for infection (Cox et al., 2023). Recent literature classifies mAbs into four classes depending on their binding mode to the spike protein (Miguez-Rey et al., 2022; Barnes et al., 2020; Kumar et al., 2021; Corti et al., 2021). Class 1 mAbs (e.g., regdanvimab, etesevimab and imdevimab) are IGHV3-encoded with short CDRH3 loops and exclusively bind to epitopes on the receptor binding motif (RBM) in the up conformation (Miguez-Rey et al., 2022; Barnes et al., 2020; Kumar et al., 2021). Class 2 mAbs (e.g., bamlanivimab) are also IGHV3-encoded but have longer CDRH3 loops, allowing them to bind to the RBD in both the up and down conformations (Miguez-Rey et al., 2022; Barnes et al., 2020; Kumar et al., 2021). Class 3 mAbs block the ACE2 binding site by binding outside the ACE2 binding site; they can also recognize both up and down RBD conformations (Miguez-Rey et al., 2022; Barnes et al., 2020; Kumar et al., 2021). Class 4 mAbs (e.g., casirivimab, sotrovimab) do not overlap with the ACE2 binding site; instead, they bind to a highly conserved epitope of RBD, specifically in the up conformation (Miguez-Rey et al., 2022; Barnes et al., 2020; Kumar et al., 2021). Classes 1–4 mAbs achieve their neutralizing effects through either direct competition with ACE2 for Classes 1 and 2 or steric hinderance of ACE2 interactions (Class 3, adjacent to RBM) and Class 4 (distal to RBM). Several mAbs drugs have received FDA approval, such as casirivimab (formerly REGN10933), imdevimab (REGN10987), bamlanivimab (LY-CoV555), etesevimab (CB6, JS016, LY-CoV016), tixagevimab (COV2-2196/AZD8895), and cilgavimab (COV2-2130/AZD1061). mAbs targeting the RBD have epitopes that either fully or partially overlap with the RBM on the RBD, effectively blocking viral entry by preventing ACE2 from binding to RBM (Mornese Pinna et al., 2021). In contrast, non-RBM mAbs like sotrovimab (VIR-7831, S309) appear to block viral infection by sterically interfering with the viral membrane fusion after ACE2 engagement with the RBM (Ji et al., 2022). Combining mAbs from different classes can synergistically enhance neutralization effects (Pandey et al., 2021). Due to the rapid emergence of new SARS-CoV-2 variants, mAb drugs recognizing conserved regions across new SARS-CoV-2 variants could be more effective (Guenthoer et al., 2024; Focosi et al., 2024).

Numerous experimental studies have been conducted to determine the structures of spike proteins and their complexes with ACE2 or mAbs. These 3D structures provide insight into the mechanistic understanding of spike - ACE2 and spike - mAb interactions, and they serve as a foundation for the development of diagnostic tools and therapeutic agents. This work has two objectives: to introduce a new computational methodology and software tools designed for probing PPIs and to take advantage of available 3D structures of spike - ACE2/mAb complexes and the computational methodology to provide mechanistic insight into how the binding of a mAb affects the binding of the spike protein to ACE2. Notably, certain mAbs can completely prevent the spike protein from binding to ACE2, while others may result in weaker binding of the spike to ACE2 (Supplementary Figure S1). The Results section of this study covers six key areas. First, we compare overall structures of the spike proteins as well as heavy and light chains of mAbs. The motivation for starting with global structure comparison is two folds: one is to examine correct labeling of the chains in the dataset and the other is to provide a solid foundation for local structural analyses. Second, we evaluate the performance of the TSR-based method in studying conformational changes and provide detailed annotations to the conformational changes using different types of TSR keys. Third, we introduce the concept of cross TSR keys, using either only C_α_ atoms or all atoms for defining binding surfaces. The approach aims to demonstrate the structural uniqueness of a binding surface from both the rest of the surface and the interior portion (non-surface) of the structures. Fourth, we discuss the structural similarities and differences of a dataset containing approximately 200 CDRH3 and CDRL3. Fifth, we introduce a novel computational methodology for quantifying amino acid structures, including the generation of the matrix of pairwise amino acid similarities, which is termed TSR-STRSUM (TSR-STRucture SUbstitution Matrix). This matrix opens the door for valuable applications in protein sequence and structure comparison. Sixth, we discuss the effect of mAb binding on the structural change of the ACE2 binding site. To clearly distinguish the mAbs already on the market from those that are not, we refer to those found in the PDB but not on the market as “mAbs” while those that have been approved and are available on the market are referred to as “mAb drugs.” In summary, this work introduces a new computational method with advantages in quantifying as well as interpreting conformational changes and defining binding surfaces. Through this extensive study of spike - mAb interactions, we have discovered unique substructures associated with the binding surfaces of specific spike - mAb pairs.

## 2 Materials and methods

### 2.1 Key generation

The process began with extracting C_α_ atoms from PDB files of each protein under analysis. Next, the three side lengths and angles of all triangles constructible from these C_α_ atoms were systematically calculated (Kondra et al., 2021). We mapped the amino acids associated with the three vertices of triangle *i* to corresponding integer IDs to three labels $l_{i1}$ , $l_{i2}$ and $l_{i3}$ ; we then ensured uniqueness of the same TSR triangle across proteins to be represented by the same integer keys by applying the rule-based label-determination of vertices of each triangle (Guru and Nagabhushan, 2001). Once $l_{i1}$ , $l_{i2}$ and $l_{i3}$ are determined for triangle *i*, we calculate *θ*_1_ using Equation 1 and *θ*_Δ_ based on *θ*_1_ values.

$$\theta_{1}=\cos^{-1}\left(\left({d_{13}}^{2}-{\left(\frac{d_{12}}{2}\right)}^{2}-{d_{3}}^{2}\right)/\left(2\times \left(\frac{d_{12}}{2}\right)\times d_{3}\right)\right)$$ (1)

$$\theta_{∆}=\left\{\begin{array}{c} \theta_{1}\;if\;\theta_{1}\leq 90{}^{\circ}\\ 180{}^{\circ}-\theta_{1}\;otherwise \end{array}\right.$$

where, for triangle *i,*

$d_{13}$ : distance between $l_{i1}$ and $l_{i3}$

$d_{12}$ : distance between $l_{i1}$ and $l_{i2}$

$d_{3}$ : distance between $l_{i3}$ and the midpoint of $l_{i1}$ and $l_{i2}$

We refer to the value of *D* as MaxDist and the value of *θ*_Δ_ as Theta (Kondra et al., 2021). MaxDist is defined as the distance of the longest edge of a triangle. Theta is defined as the angle that is <90° between the line from the midpoint of the edge of $l_{i1}$ and $l_{i2}$ to the opposite vertex $l_{i3}$ and half of the $l_{i1}$ - $l_{i2}$ edge. once the labels: $l_{i1}$ , $l_{i2}$ , $l_{i3}$ , *D* and *θ*_Δ_ are determined, we use Equation 2 to calculate the key for each triangle (Kondra et al., 2021). Crucially, the generated key for each triangle depends on $l_{i1}$ , $l_{i2}$ and $l_{i3}$ (vertex labels), Theta (*θ*_∆_) and MaxDist (D). This design ensures that the keys, while remaining invariant to rotations and translations, can effectively capture scale changes in protein structures, making them suitable for alignment-free, pairwise comparison of 3D structures.

$$k=\theta_{T}d_{T}\left(l_{i1}-1\right)m^{2}+\theta_{T}d_{T}\left(l_{i2}-1\right)m+\theta_{T}d_{T}\left(l_{i3}-1\right)+\theta_{T}\left(d-1\right)+\left(\theta -1\right)$$ (2)

### 2.2 Protein structural similarity and distance calculation

We apply the Generalized Jaccard coefficient measure (Jaccard, 1901), Equation 3, for the calculation of similarity between two proteins.

$${\text{Jac}}_{gen}=\sum_{i=1}^{n}\epsilon_{i}/\sum_{i=1}^{n}z_{i}$$ (3)

where *n* is the total number of unique keys in proteins *p*_*1*_ and *p*_*2*_.

Equivalence *ϵ* for a given key *k*_*i*_ in two different proteins *p*_*1*_ and *p*_*2*_ is defined as $\epsilon_{i}=k_{i}^{p_{1}}\cap k_{i}^{p_{2}}$ where *∩* is defined by the minimum of the count of corresponding keys.

Difference *z* for a given key *k*_*i*_ in a pair of proteins is defined as $z_{i}=k_{i}^{p_{1}}\cup k_{i}^{p_{2}}$

where ∪ is defined by the maximum of the count of corresponding keys. The count of a key is the number of times that key occurs (occurrence frequency) within a protein.

Once a similarity matrix is generated, the distance matrix is derived simply by taking each value in the similarity matrix and subtracting it from 1. Protein structure clustering is visualized using on Average Linkage Clustering (Ackerman and Ben-David, 2016). A six-layer fully connected neural network is used for classifying protein structures (Kondra et al., 2022). Structural images were prepared using the Visual Molecular Dynamics (VMD) package (Humphrey et al., 1996).

### 2.3 Definition of distinct, total, distinct common, total common, and specific keys

The TSR-based method utilizes different types of keys so that the clustering results can be interpreted. A key represents a triangle that is made up of three amino acids. Each triangle represents the smallest substructure of the protein being analyzed. Every possible combination of three amino acids in a protein’s amino acid sequence is detected and generated into a substructure by the TSR algorithm. Each key contains a specific geometry that allows for the further classification of the substructure. Five types of TSR keys (Kondra et al., 2022) are used to analyze the results. Distinct keys represent all keys for each individual protein without considering the key frequency. Total keys represent all the distinct keys but also account for key frequency. Distinct common keys represent all the common substructures that are shared amongst all proteins in a dataset. In other words, a triangle that is made up of the same three amino acids that have similar angles and distances and is found in all proteins of the dataset is identified as a *common* key. Total common keys represent all distinct common keys accounting for frequency. Specific keys represent substructures that are unique to a certain protein (sub)family. Determination of common and specific keys are useful in a protein structure analysis because *common* keys can provide information on how a protein is folded and *specific* keys are the structural foundations for motif discovery.

### 2.4 Dataset preparation and analysis

We have prepared a dataset containing approximately 200 SARS-CoV-2 spike proteins and their interacting mAbs. The chains of the spike proteins and their mAbs were visualized using the VMD software to ensure that the correct chains are selected for probing spike - heavy chain interactions, spike - light chain interactions and heavy chain - light chain interactions. In this study, we aimed to include all RBDs of spike structures complexed with one or more mAbs. There are full-length spike structures complexed with one or more mAbs. To avoid a potential structural effect of the outside RBD region of the spike on the RBD, those full-length spike structures were not included in this study. FDA approved mAb drugs: sotrovimab (six structures), bamlanivimab (one structure), cilgavimab (one structure) and tixagevimab (one structure) are included in the dataset. The PDB IDs, chain information, annotated CDRH3 and CDRL3, spike - heavy chain interfaces and spike - light chain interfaces can be found in the Supplementary Material S1. All proteins in the dataset were selected from the PDB (Berman et al., 2000).

### 2.5 Development of a new version of the TSR-based method using all atoms

We have previously reported the TSR-based method using C_α_ atoms to quantify protein (sub)structures and motif discovery (Kondra et al., 2021). To quantify the structures of the side chain of a protein, we have developed a new version of the TSR-based method where every possible triangle is constituted from all the atoms in a protein, especially those in a binding site. C, N, O and S atoms were assigned unique integer labels. The bin boundaries for Theta were used the same as we reported for the TSR algorithm using C_α_ atoms (Kondra et al., 2021). Seventeen bins with a one-angstrom interval were used for MaxDist. Equation 2 is used to generate keys when utilizing all atoms.

### 2.6 Development of a new version of the TSR-based method using cross keys

A *cross* TSR key is defined based on the location of the atoms associated with the vertices. Specifically, these are keys are constructed from triangles where one of the atoms is in one polypeptide and the other two are in a different polypeptide. The *cross* keys allow the 3D structure of the interface of a molecular complex to be represented and the similarities between different interfaces to be quantified.

### 2.7 Development of a new TSR algorithm for quantifying structures of amino acids

The TSR concept was used to develop a new algorithm for quantifying the similarities between structures of different amino acids and those between same amino acids at the different positions. The bin boundaries for Theta and MaxDist are the same as those for the TSR algorithms for using all atoms.

### 2.8 Sequence alignment

SnapGene was applied to conduct multiple sequence alignments. Sequence alignment and phylogenetic analysis were done using MEGA7 (Kumar et al., 2016). TM-align was used for pairwise sequence alignment (Zhang and Skolnick, 2005), and Muscle algorithm is used for multiple sequence alignment (Edgar, 2004).

### 2.9 Statistical analysis

*t*-test was used to identify statistical differences between different structural comparison methods. A threshold of *p* < 0.05 was used to determine significance.

## 3 Results

### 3.1 Global structural comparison of spike and monoclonal antibodies and interpretation of clustering results

**FIGURE 1:**
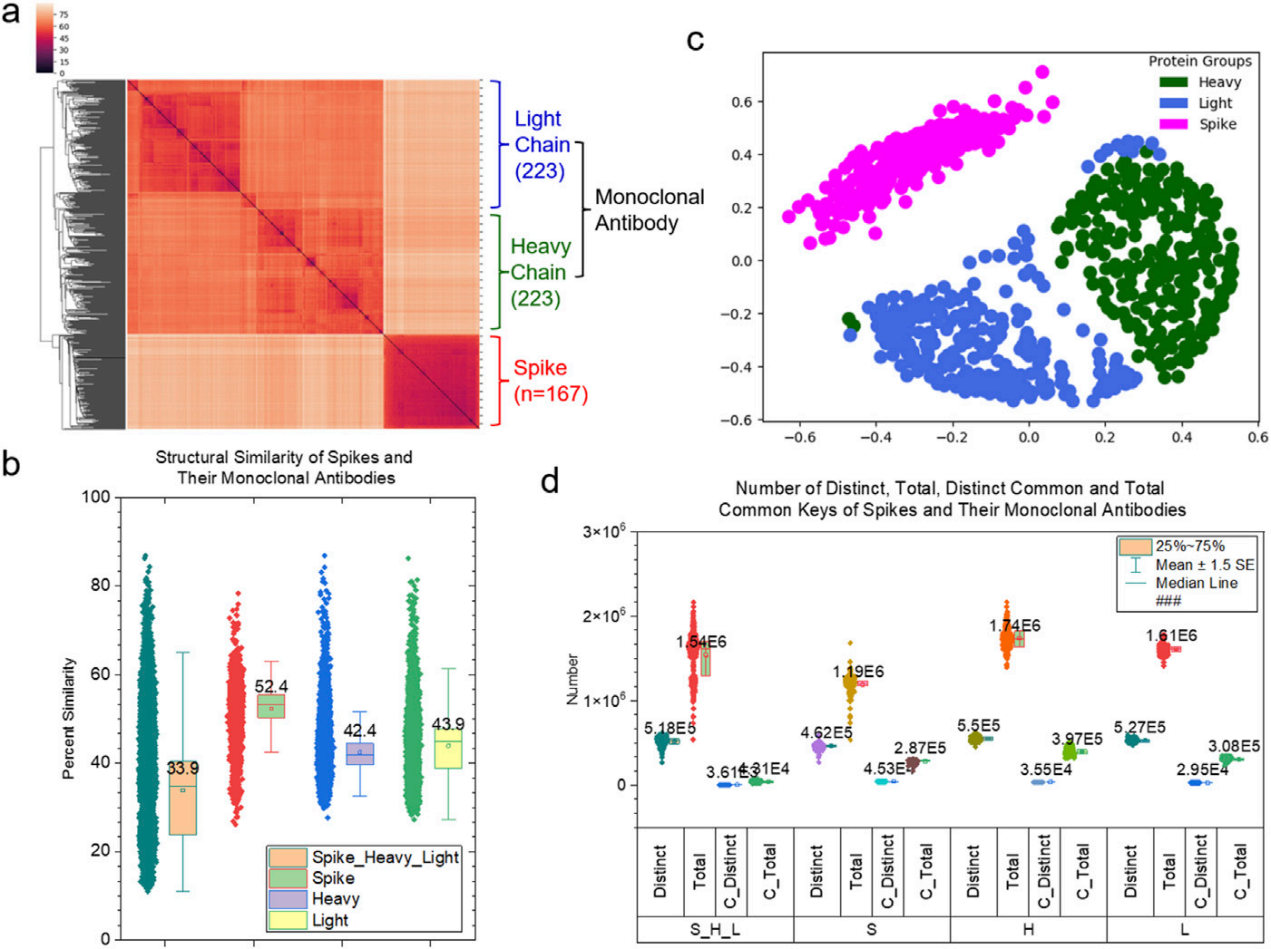
Clustering and MDS studies of the spike proteins and their monoclonal antibodies. **(a)** The clustering heatmap shows the hierarchical clustering result of the spike proteins and their monoclonal antibodies (heavy and light chains). The numbers of the polypeptides are indicated. The dissimilarity values are indicated in the upper left corner. This is applied to all the clustering heatmaps in this study. The PDB IDs, and chain and class labels can be found in Supplementary Material S1A. The complete list of the clustering result in the same order as the clustering heatmap are provided in Supplementary Material S1B; **(b)** The structural similarity of the spike proteins, heavy chains and light chains and all the polypeptides are shown. The average values and SDs are indicated; **(c)** The MDS analysis of the spike proteins and their monoclonal antibodies (heavy and light chains); **(d)** The distinct, total, distinct common and total common keys of the spike proteins, heavy chains, light chains and all three polypeptides were calculated and are present. The number of those keys are labeled.

More than 200 depositions of mAb structures against spike proteins are currently accessible in the PDB. Based on these available structures determined by experiments, it is possible to conduct a comprehensive study for estimating the comparative models of mAbs against the spike surface antigens. The structure of an antibody forms a Y-shaped glycoprotein that is composed of two identical heavy and two identical light chains. The first step of the comprehensive study is to compare the global structures of variable regions of heavy and light chains of mAbs that bind to target antigens. The hierarchical clustering result obtained using pairwise structure similarities based on the TSR keys calculated using C_α_ atoms (CA-TSR keys) demonstrates two distinct clusters: a mAb cluster and a spike cluster. The mAb cluster can be further divided into heavy chain and light chain clusters indicating that heavy chains are structurally different from light chains (Figure 1A). Light chains (43.9%) have a slightly higher structural similarity on average than heavy chains (42.4%). Spike proteins (52.4%) have higher structural similarity on average than both heavy chains and light chains of mAbs (Figure 1B). As the first step in interpreting structure similarity, we have performed sequence alignment analyses: the pairwise sequence using TM-align method (Zhang and Skolnick, 2005) and the multiple sequence using Muscle algorithm (Edgar, 2004). The sequence alignment study shows high sequence similarities (Supplementary Figure S2). The trends for structural similarities using the TSR-based method and for sequence similarities using the pairwise sequence comparison are the same. The multiple sequence alignment analysis shows that the heavy chains have a higher sequence similarity than the light chains, but an opposite scenario is observed when the corresponding structural similarities are compared. To verify the hierarchical clustering results, we have performed MDS and classification analyses. Both MDS (Figure 1C) and classification (Supplementary Figures S3A, B) results agree with the results from the hierarchical clustering (Figure 1A). We have identified *common* (3.61 × 10^3^/5.18 × 10^5^ = 0.697%) and *Common* (1.31 × 10^4^/1.54 × 10^6^ = 0.851%) TSR keys. The result demonstrates that only a small portion of the substructures are conserved across spike proteins, as well as for heavy and light chains (Figure 1D). The conserved substructures represented by *common* keys have shorter MaxDist values and larger Theta values; further, they enrich certain types of amino acids (valine for spike proteins and serine for heavy and light chains) (Supplementary Figures S4A–D). When considering 167 spike proteins as one group, 233 heavy chains as another group, and 233 light chains as the third group, the three groups share a significant number of common substructures (948213/1333811 = 71.1%) (Supplementary Figure S5). Small percentages of the *Specific* TSR keys were identified for the spike (0.496%), heavy chain (5.88%) and light chain (1.37%) groups (Supplementary Figure S5). The spike proteins share a high amino acid sequence similarity (on average 98.7%–98.9%) (Supplementary Figure S2). We carefully examined each spike structure and found that while some are variants (e.g., Beta, Delta, Omicron), others are just spike proteins alone or protein complexes with ACE2 and/or mAbs. This suggests that mutations and/or the binding of an interacting protein could lead to substantial structural changes. The *specific* TSR keys were identified for certain subsets of spike proteins (Supplementary Figure S6). In conclusion, the TSR-based method can distinguish structures of heavy chains from those of light chains. It provides a foundation for further substructural analyses.

### 3.2 Development of the TSR-based method for quantifying conformational changes upon binding of an interacting polypeptide

Proteins are inherently flexible molecules due to the non-covalent nature of their folded 3D structures, but crystal structures often make a protein appear as if it exists in a single state (Gutteridge and Thornton, 2005). However, a protein exists in a range of dynamic conformations that are triggered at a local binding site and propagated to the neighboring and distal regions upon the binding of an interacting protein. Dynamic conformations of a protein are often intimately coupled to its biochemical function. The objective of this section is to demonstrate that TSR keys offer a convenient way to probe such conformational changes. First, we will show the results on tracing global structural changes. Second, we will focus our discussion on structural changes at binding sites.

**FIGURE 2:**
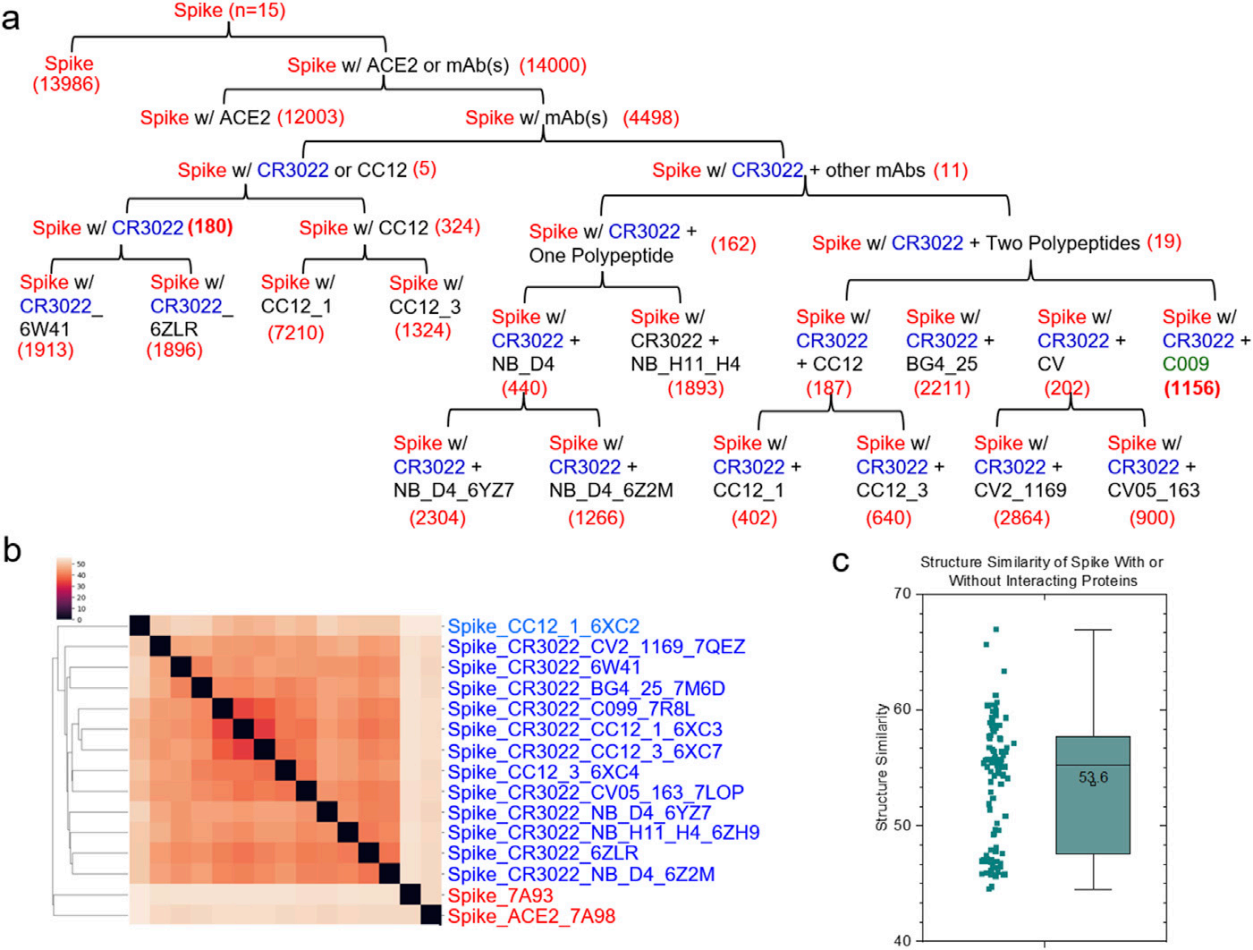
Hierarchical clustering of the spike proteins with or without an interacting protein. **(a)** The structures of the spike proteins were organized in a hierarchical fashion from the root to leaf nodes based on whether the spike proteins interact with CR3022 alone or an additional mAb. The free spike protein and the spike-ACE2 complex are included as the references. The numbers of the *specific* keys for each level of the hierarchical organization are indicated; **(b)** The clustering heatmap shows the hierarchical clustering result of the spike proteins. mAbs and PDB IDs are indicated; **(c)** The pairwise structural similarity of the spike proteins presented in **(b)** is shown. The average of the structural similarity is labeled; **(a–c)**, A total fifteen structures are presented in the panels of **(a–c)**.

To provide a detailed interpretation of global and local conformational changes, we prepared a small dataset containing the maximum number of available spike structures complexed with the same mAbs. Eleven spike proteins complexed with CR3022 were found by searching the PDB. CR3022, a member of Class 4 mAbs, promotes the destruction of the pre-fusion spike protein trimer by perturbing the folding of both the N-terminal domain (NTD) and RBD (Huo et al., 2020a). These eleven structures can be divided into nine groups: spike protein group with CR3022 alone (two structures) (Supplementary Figure S7A), spike protein group with CR3022 and NB-D4 (two structures), spike protein group with CR3022 and CV2-1169 (one structure), spike protein group with CR3022 and BG4-25 (one structure), spike protein group with CR3022 and C099 (one structure), spike protein group with CR3022 and CC12.1 (one structure) (Supplementary Figure S7B), spike protein group with CR3022 and CC12.3 (one structure) (Supplementary Figure S7C), spike protein group with CR3022 and CV05-163 (one structure) and spike protein group with CR3022 and NB-H11-H4 (one structure). CC12.1 and CC12.3 belong to Class 1 mAbs. Both overlap with the ACE2 binding site, but not with the CR3022 epitope. CR3022 (*K*_d_ = 6.3 nM or *K*_d_ = 15 or 30 nM) (Tian et al., 2020; Huo et al., 2020b) has a binding affinity similar to that of CC12.1 (*K*_d_ = 17 nM) and CC12.3 (*K*_d_ = 14 nM) (Yuan et al., 2020). The structures of spike proteins complexed with either CC12.1 or CC12.3 are available in the PDB (Supplementary Figures S7C, D). Therefore, we included two more structures in the dataset for quantifying the conformational change of spike - CR3022 upon the binding of CC12.1 or CC12.3 as well as the conformational change of spike - CC12.1 or spike - CC12.3 upon the binding of CR3022. Two structures of the spike alone and the spike with ACE2 were included as the controls. The final version of this small dataset contains a total of fifteen structures. This dataset has a total of 680,133 distinct keys. We have organized the structures in a hierarchical fashion that depends on whether the spike has an interacting polypeptide and whether the spike binds to ACE2, one mAb, or more mAbs (Figure 2A). The hierarchical structure of spike proteins has six levels (Supplementary Figure S8). To avoid accounting for the effect of different numbers of amino acids into conformational changes, we have trimmed the structures to make sure all fifteen structures have identical amino acid sequences.

#### 3.2.1 TSR keys are used to quantify conformational changes of proteins and interpret the results in the context of a hierarchical organization

**FIGURE 3:**
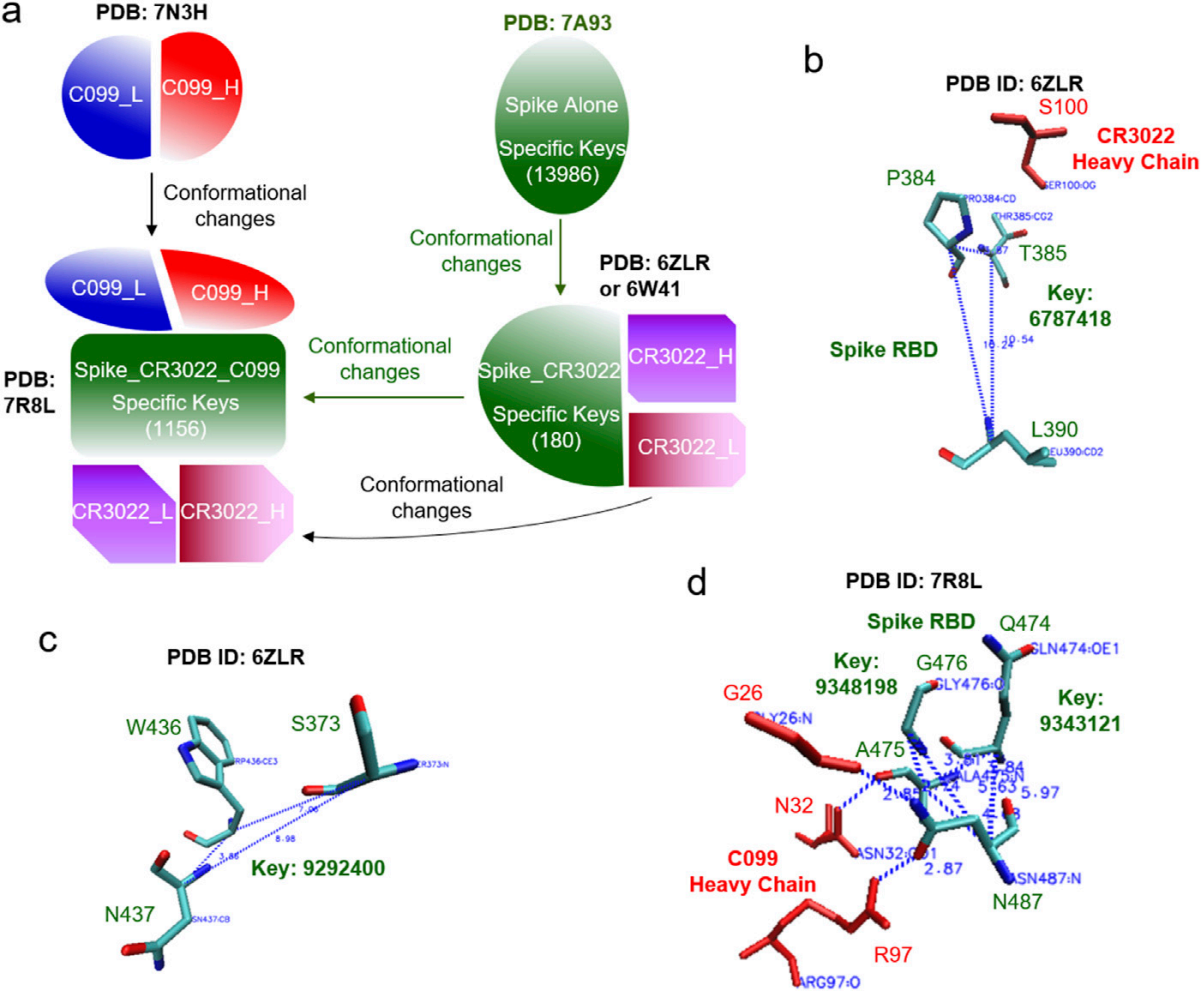
An example of how the TSR-based method can quantify structural changes is illustrated. **(a)** The structural changes of spike upon the binding of CR3022 are shown. The structural changes of C099 are also shown after it binds to spike and the structural changes of the spike-CR3022 complex. The number of specific keys for spike are indicated; **(b-d)** The representative triangles associated with the selected specific keys for spike complexed with CR3022 **(b, c)** and spike complexed with both CR3022 and C099 **(d)** are illustrated. Proteins, amino acids and keys are indicated; **(a–d)** PDB IDs are indicated.

The hierarchical cluster analysis of spike proteins shows two clusters. The first one, a smaller cluster, contains the spike alone and the spike with ACE2. The other, larger cluster contains spike proteins with mAbs (Figure 2B). No two structures are identical, even though they all have the same amino acid sequences. The average of the pairwise structural similarities is 53.6% (Figure 2C). The clustering result based on the hierarchical clustering method (Figure 2B) does not perfectly match with the hierarchical structure arranged based on the number of interacting polypeptides and which polypeptide (polypeptides) binds (bind) to spike proteins (Figure 2A; Supplementary Figure S8). *Specific* keys exclusively belonging to each node at every level of the hierarchical arrangement were identified. The *specific* keys not only serve as decision makers for each node to distinguish itself from other nodes, but they are also used to quantify conformational changes (Figure 2A; Supplementary Figure S8). For example, 19 distinct keys were identified as specific to the spike proteins complexed with CR3022 and two polypeptides at the level 4 (Figure 2A; Supplementary Figure S8). Two representative keys (7799302 and 8756479) out of 19 are shown in Supplementary Figure S9. The 7799302-associated triangle is formed from Cys361, Val362 and Cys525 (Supplementary Figure S9A) and the 8756479-associated triangle is constituted from Lys417, Arg454 and Leu455 (Supplementary Figure S9B). The latter triangle is close to Gly97 of the heavy chain of CC12.3 (Supplementary Figure S9B). Figure 3 shows another representative example of how *specific* keys can be used to quantify conformational changes. A total of 13,986 distinct *specific* keys (13,986/680,133 = 2.06%) are found to belong to the structure of the spike alone and are not found in any other fourteen structures in the dataset (Figure 3A). Similarly, 180 distinct *specific* keys (180/680,133 = 0.0265%) are found for the spike complexed with CR3022 only and 1156 distinct *specific* keys (1156/680,133 = 0.0170%) are identified for the spike complexed with both CR3022 and C099 (Figure 3A). The key set with 180 *specific* keys represents the substructure unique to the spike complexed only with CR3022. Two representative keys (6787418 and 9292400) out of 180 *specific* keys are shown in Figures 3B, C. The key of 6787418 was calculated from the triangle constituted from Pro384, Thr385 and Leu 390 of the spike. This triangle is close to Ser100 of the CR3022 heavy chain (Figure 3B). Similarly, the key set containing 1156 specific keys represents the substructure unique to the spike complexed with both CR3022 and C099. Two representative keys (9343121 and 9348198) out of 1156 specific keys are shown in Figure 3D. These two keys were calculated from two triangles: 9343121 formed by Gln474, Ala475 and Asn487 and 9348198 formed by Ala475, Gly476 and Asn487. The two triangles share a common edge and are close to three amino acids (Gly26, Asn32 and Arg97) of the C099 heavy chain (Figure 3D). The numbers of amino acids, distinct keys, total keys, distinct *common* keys and total *common* keys are summarized in Supplementary Table S1.

We can use the same approach to study the hierarchical relationships of the heavy and light chains of CR3022. The hierarchical cluster analysis shows that the heavy chains of CR3022 are grouped together and the light chains of CR3022 are also grouped together (Supplementary Figure S10). The hierarchical organization of the heavy chains of CR3022 is shown in Supplementary Figure S11. The *specific* keys are identified for every conformation. 154 *specific* keys were identified as belonging uniquely to the heavy chain of CR3022. They are not found in nine heavy chains of C099 (one), CC12.1 (two), CC12.3 (two), BG4-25 (one), CV05-163 (one) and CV2-1169 (one), nor in the light chains of CR3022 and all the other corresponding mAbs and ACE2. Two representative keys (5958107, 5960137) out of 154 are shown in Supplementary Figure S12. A total of 16 keys were identified as specific to the heavy chains of CR3022 complexed only the spike. Three *specific* keys (7114234, 7236018, 7666384) out of 16 are shown in Supplementary Figure S13. Those key-associated triangles are close to Phe377 and Cys379 of the spike (Supplementary Figure S12). Additionally, 4 keys were identified exclusively for the heavy chains of CR3022 complexed with the spike and other mAbs. Supplementary Figure S14 shows three *specific* keys (7263475, 7666383, 8866112) out of 4. The key7666383-associated triangle is close to Phe373 of the spike. In the results above from the study of the spike proteins and the heavy chains, we have demonstrated that TSR keys can be used to interpret structural differences and quantify conformational changes.

#### 3.2.2 TSR keys are used to quantify conformational changes of binding sites

**FIGURE 4:**
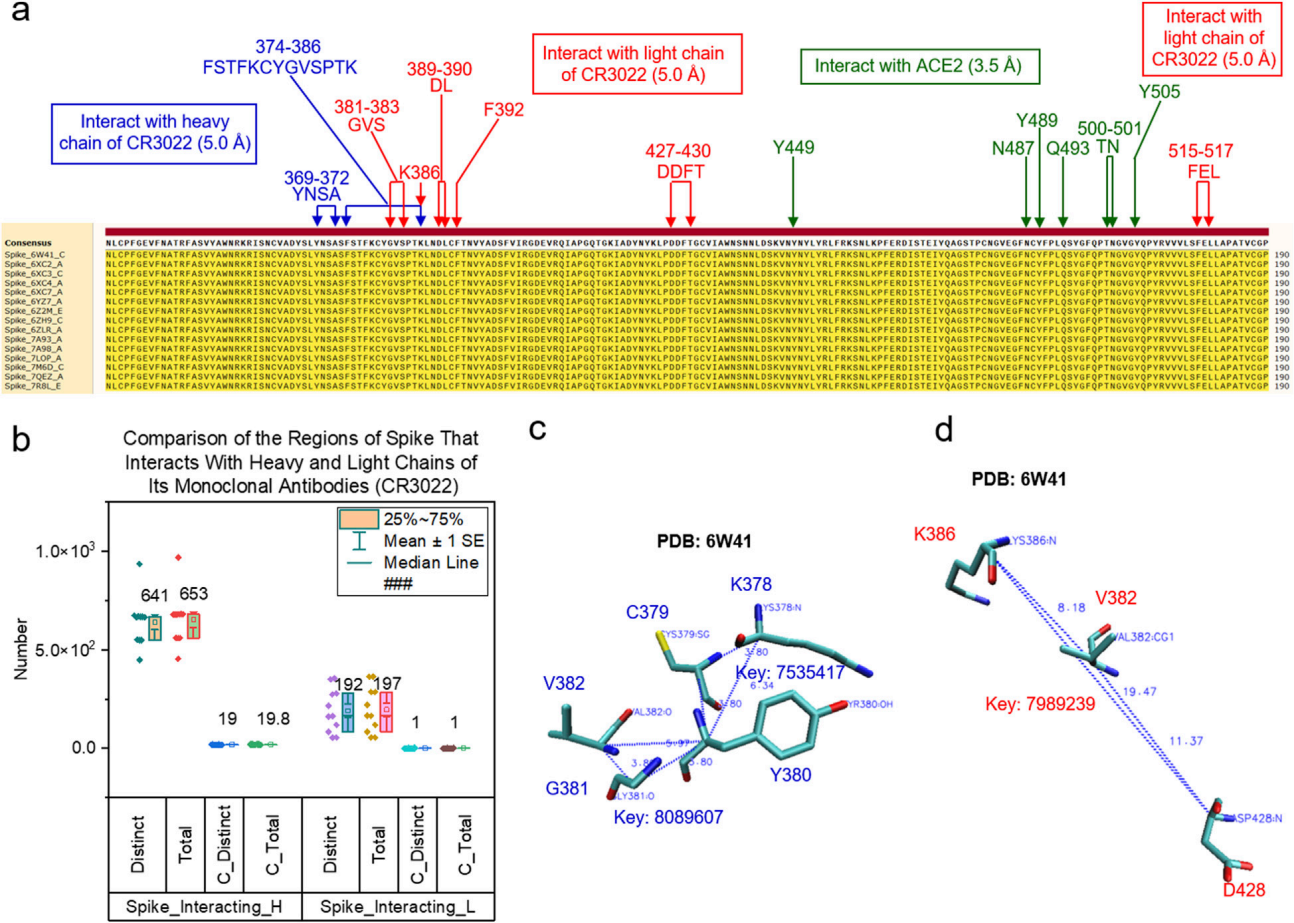
Different types of keys of the spike regions that interact with the heavy chains of CR3022 compared with those interacting with the light chains of CR3022 are shown. **(a)** The multiple sequence alignment shows the regions of spike that interact with the heavy chain and light chain of CR3022 and ACE2. Green color represents the spike region that interact with ACE2; **(b)** The distinct, total, distinct *common* and total *common* keys for the spike regions that interact with the heavy and light chains of CR3022 were calculated and are presented. The average is indicated; **(c, d)** The triangles associated with the common keys are shown for the spike regions that interact with the heavy chain **(c)** or the light chain **(d)** of CR3022. PDB IDs, amino acids and keys are indicated; **(a, c-d)** blue color represents the spike region that interacts with the heavy chain of CR3022 and red color represents the spike region that interacts with the light chain of CR3022.

To demonstrate the application of the TSR-based method in quantifying local conformational changes, we focused on three binding sites of the spike protein: a site for binding of heavy chain of CR3022, a site for binding of light chain of CR3022 and a site for interacting with ACE2 (Figure 4A). As stated earlier, the spike proteins in the dataset all have identical amino acid sequences (Figure 4A). The heavy and light chains of CR3022 physically interact with spike proteins. First, we will discuss these interactions. The binding of CR3022 to the spike protein could cause distal conformational changes at the ACE2 binding. It was reported that CR3022 binds tightly to the outside of RBM and allosterically perturbs ACE2 binding (Huo et al., 2020b). Second, we will discuss the distal conformational changes.

We used 5 Å as the cutoff to define an interface; that is, the interface includes all amino acid pairs between two molecules whose atomic distance falls within 5 Å. We performed hierarchical cluster analyses of the spike regions at the interfaces with either the heavy chains (Supplementary Figure S15A) or the light chains (Supplementary Figure S15B) of CR3022. The spike regions that interact with the heavy chains of CR3022 are structurally different (Supplementary Figures S15A, C). A scenario with greater structural diversity is observed for the spike regions that interact with the light chains of CR3022 (Supplementary Figures S15B, C). These structural changes could be due to (i) a difference in amino acid sequences of the CR3022 heavy (Supplementary Figure S16) or light (Supplementary Figure S17) chains, (ii) allosteric effect of other mAbs besides CR3022 (For example, binding C099 to the alters the spike region that interacts with CR3022. C099 belongs to Class 1 mAbs and has broad neutralizing activities (Witte et al., 2023). (Supplementary Figure S18) or (iii) a difference in amino acid sequences of the spike protein (They were trimmed to have identical sequences, but local structure difference caused by different sizes of spike RBD regions are preserved). The structural differences of the spike regions that interact with CR3022 can be quantified by the numbers of *specific* keys that exclusively belong to the situations where spike proteins interact with CR3022 alone or CR3022 plus another mAb (Supplementary Figure S19). All the spike proteins (Supplementary Figures S15A, B) interact with CR3022. It will be interesting to know whether there are conserved substructures in the regions that interact with CR3022. Nineteen *common* keys were identified for the spike regions interacting with the heavy chains of CR3022 (Figure 4B). Two representative *common* keys out of the nineteen are shown in Figure 4C. One *common* key was identified for the spike regions that interact with the light chains of CR3022 (Figure 4B). It is shown in Figure 4D.

**FIGURE 5:**
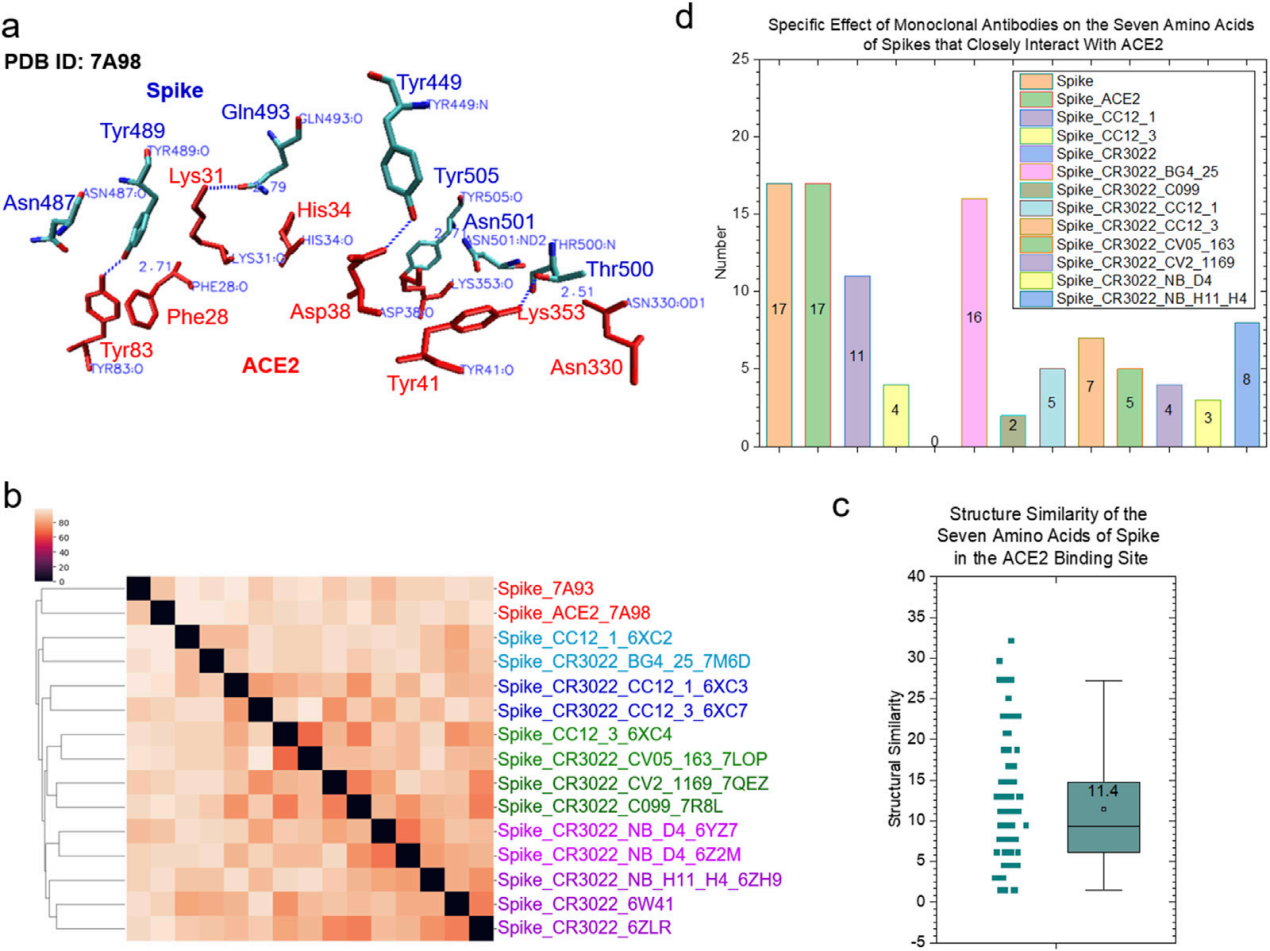
The clustering study shows the steric effect of the binding of CR3022 on the ACE2 binding of spike. **(a)** The seven amino acids that have close interactions with ACE2 are shown. The PDB IDs, proteins and amino acids are indicated. Red color represents ACE2 and blue color represents spike; **(b)** The result of the hierarchical clustering study is illustrated. PDB IDs and with or without ACE2 or mAbs are indicated; **(c)** The pairwise structural similarity of the spike proteins presented in **(b)** is shown. The average of the structural similarity is labeled; **(d)** The numbers of the specific keys of the seven amino acids that closely interact with ACE2 were calculated and are presented. The average values are indicated.

We have demonstrated that the *specific* and *common* keys can be used to quantify structural similarity and dissimilarity of the protein binding sites. To evaluate the allosteric effect of CR3022 binding on the ACE2 binding site of the spike protein, we used 3.5 Å as the cutoff to select spike residues that closely interact with ACE2. Seven amino acids (Tyr449, 489, 505, Asn487, 501, Gln493 and Thr500) satisfied this criterion (Figure 5A). These seven amino acids of the spike proteins have high structural diversity (Figures 5B, C) and do not share any common substructures. *Specific* keys were identified for the most spike groups depending on whether they have an interacting polypeptide and which polypeptide(s) interacts (interact) with the spike proteins (Figure 5D).

### 3.3 Introducing two new TSR strategies for probing spike - mAb interactions

**FIGURE 6:**
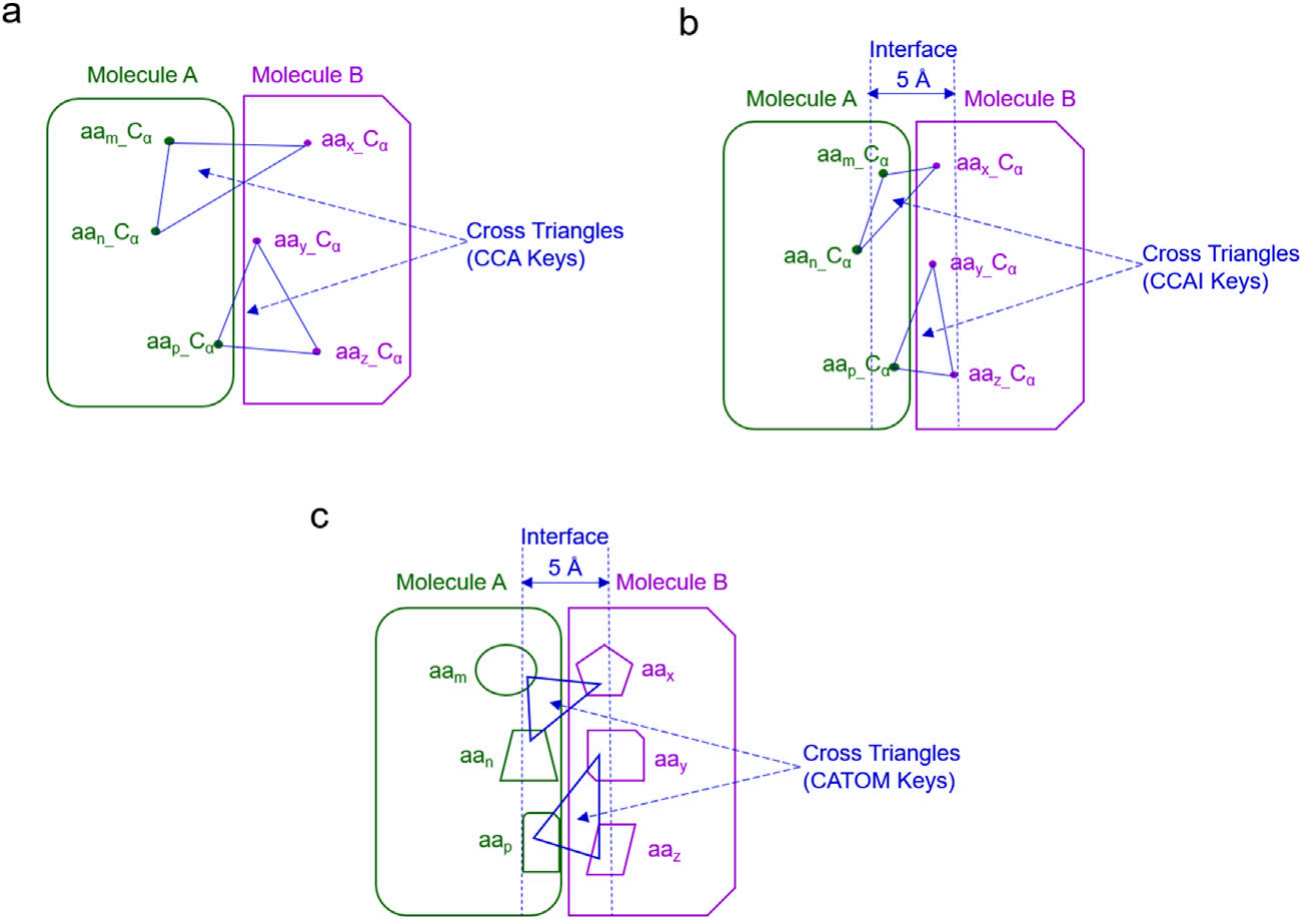
The schema for different types of *cross* keys between two molecules is shown. **(a)** An example of the *cross* TSR keys only using C_α_ atoms (CCA TSR keys) is shown; **(b)** An example of the *cross* TSR keys only using C_α_ atoms (CCA TSR keys) at the interface between two molecules (CCAI) is illustrated. **(c)** An example of the *cross* TSR keys using all atoms between two molecules (CATOM TSR keys) is shown; **(b, c)**, The cutoff distance for the interface is 5 Å.

Life is about relationships between molecules, not a property of any single molecule (Zuckerkandl et al., 1962). To understand assembly of protein complexes, one must understand biochemical underpinnings that facilitate exact and specific interactions at the interface. To achieve this goal, we have developed two new strategies: (i) TSR keys using all atoms including C_α_ atoms and (ii) *cross*-TSR keys that are specifically designed for probing molecular interactions. The *cross* keys are calculated from triangles constituted from 1 C_α_ atom of one molecule and 2 C_α_ atoms from another molecule. They are named CCA keys (Figure 6A). The *cross* keys at an interface are meaningful. When *cross* keys at an interface are calculated using only C_α_ atoms of the amino acid pairs between two molecules whose atomic distance falls within 5 Å, then they are named CCAI keys (Figure 6B). CCAI keys are a subset of CCA keys. When *cross* keys are calculated using all atoms at an interface whose pairwise atomic distance between two molecules are within 5 Å, then they are named CATOM keys (Figure 6C).

**FIGURE 7:**
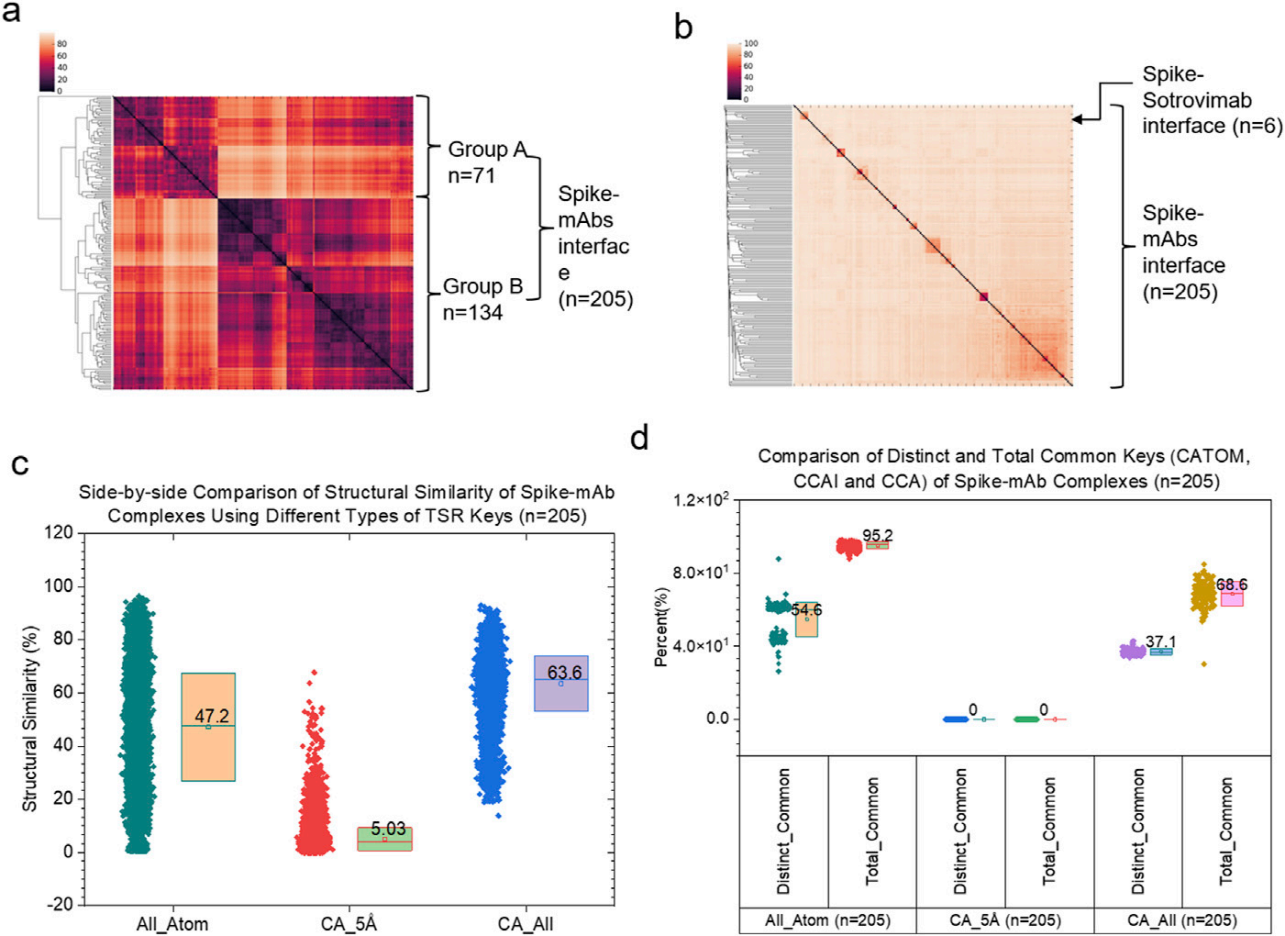
Hierarchical cluster analyses of interface between spike and mAbs using different types of cross TSR keys. **(a, b)** The hierarchical clustering using CATOM **(a)** and CCAI **(a)** keys. The numbers of total structures are labeled. Group A and Group B and numbers of structures in Group A and Group B are labeled for the clustering using CATOM keys **(a)**. The cutoff distance for the interface between spike and mAbs (heavy and light chains together) is 5 Å **(b)**; **(c)** Structural similarities using CATOM (All-Atom), CCAI (CA_5 Å) and CCA (CA_All) were calculated and are present. Average values are labeled. SDs are shown; **(d)** Percentages of distinct and total common keys using CATOM (All-Atom), CCAI (CA_5 Å) and CCA (CA_All) were calculated and are present. Distinct Common Key% = Number of Distinct Common Keys/Number of Distinct Keys. Total Common Key% = Number of Total Common Keys/Number of Total Keys. Average values are labeled. SDs are shown.

In this work, we introduce and study the concept of *cross* keys between two proteins for the first time. We compare the applications of different types of *cross* keys in clustering analyses of structures arising at molecular interaction surfaces. The cluster analysis using CATOM keys shows two large clusters (Figure 7A) while the analyses using either CCAI (Figure 7B) or CCA (Supplementary Figure S20) keys reveal several small clusters. As expected, six spike - sotrovimab complexes were grouped in one small cluster when CCAI keys were used (Figure 7B). A similar scenario was observed for the case using CCA keys. As expected, the structural similarity using CCAI keys is much smaller than those using CATOM and CCA keys (Figure 7C). The numbers of distinct and total keys using CATOM, CCAI and CCA are shown in Supplementary Figure S21. Distinct keys represent different types of substructures whereas total keys represent entire structures using the smallest units (triangle). One of the reasons why the structural similarity using CCAI keys is smaller is because spike - mAb pairs using CCAI keys do not share common substructures, demonstrating a high diversity among the backbone structures at the molecular interface. In contrast, the high percentages of common keys were observed for the situations using both CCA and CATOM keys (Figure 7D).

**FIGURE 8:**
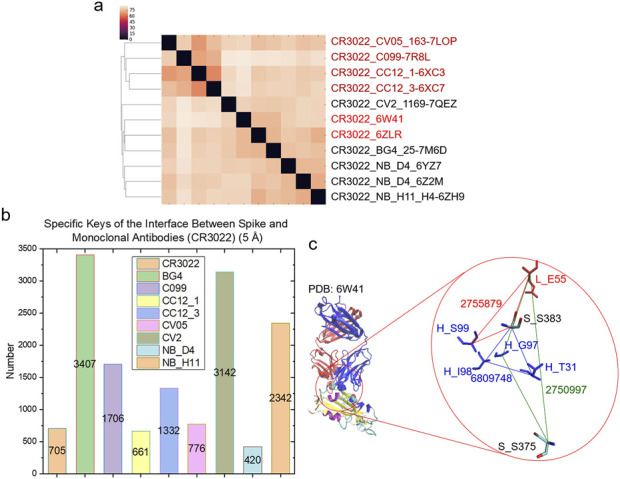
Hierarchical cluster analysis of spike and CR3022. **(a)** The hierarchical clustering analysis shows structural relationships of the interface between spike and CR3022. Two of eleven spike - CR3022 pairs do not have a second mAb besides CR3022. The rest nine spike - CR3022 pairs have one additional mAb. The additional mAbs are labeled. PDB IDs are shown; **(b)** Specific keys for each spike - CR3022 pair were calculated and are present. Average values are labeled; **(c)** Representative specific keys for a spike - CR3022 pair without an additional mAb are shown. PDB ID, amino acids and keys are labeled. S, spike; H, heavy chain of CR3022; L, light chain of CR3022. **(a–c)** The cutoff distance for the interface between spike and mAbs (heavy and light chains together) is 5 Å.

To demonstrate the specificity of *cross* keys, we focused on the spike - CR3022 interfaces. We chose CCAI keys as the representative example. The cluster analysis shows that the eleven interface structures are diverse although all are spike - CR3022 interfaces (Figure 8A). *Specific* CCAI keys were also identified for each of the eleven spike - CR3022 interfaces (Figure 8B). Three *specific* CCAI keys were identified as the representative of the spike - CR3022 alone interface when the spike is in a complex with only CR3022 (Figure 8C).

As stated earlier, mAbs can be divided into four classes depending on up or down conformation of RBD and whether a binding site is within RBM or outside RBM. Thus, we aimed to test whether *cross* keys can distinguish different types of mAbs. We prepared a small dataset containing 10 members for each class. The criterion for choosing such members is to keep high diversity by removing the mAbs having high sequence similarities. The cluster analysis using CCA keys demonstrates that the method cannot distinguish one class from other classes for both the spike - heavy chain pairs and the spike - light chain pairs (Supplementary Figure S22). This result raised the question whether one-molecule (C_α_) keys can distinguish each class. The cluster analysis using one-molecule keys cannot distinguish different types of classes although such keys can nicely separate the heavy and light chains (Supplementary Figure S23). We have shown that applying size filtering can improve clustering (Kondra et al., 2022), so we applied this to CCA keys. The clustering result shows that CCA keys with the size filtering can distinguish Class 1 from Classes 2, 3 and 4 for both the spike - heavy chain and the spike - light chain pairs (Supplementary Figure S24). The method, however, cannot distinguish Classes 2, 3 and 4. The size filtering shows that the CCA keys associated with smaller triangles will only help distinguish Class 1 from other classes. Next, we performed another cluster analysis using CCAI keys. If CCAI keys are used, the method can distinguish Class 1 only for the spike - heavy chain interfaces but not for the spike - light chain interfaces (Supplementary Figure S25). To support this clustering result, thirty-two *specific* CCAI keys were identified exclusively for the spike - heavy chain interfaces of Class 1 mAbs and no *specific* CCAI keys were identified for other Classes.

### 3.4 Application of the TSR-based method in probing structural differences of CDRH3 and CDRL3 of mAbs

Antibodies are formed by heavy and light chains composed of constant and variable regions. The latter include six CDRs (three: CDRL1, CDRL2 and CDRL3, for a light chain and three: CDRH1, CDRH2 and CDRH3, for a heavy chain) that constitute the antigen binding site (Gabrielli et al., 2009). The length and composition of the CDR sequences of mAbs are highly variable, especially in the CDR3 due to the gene recombination mechanism (Dondelinger et al., 2018). Despite sequence diversity, five out of six CDRs (CDRH3 being the exception) in antibodies assume a limited number of conformations called canonical structures (Teplyakov and Gilliland, 2014). Notably, CDRH3 plays a crucial role in mediating individual antibody recognition, sometimes by changing its conformation upon antibody binding (Shirai et al., 1996). The other five CDRs are also more or less implicated in increasing binding affinity to the antibody and some contact residues can even be situated within framework of variable regions (Davies and Cohen, 1996). We will focus on the discussion on CDRH3 and CDRL3 in this section.

The cluster analysis of the heavy chains using CA keys shows two clusters. One cluster contains smaller variable regions of the heavy chains (107–129 amino acids) and the other cluster has relatively larger variable regions (170–232 amino acids) (Supplementary Figure S26A). The cluster analysis of only the CDRH3 regions using CA keys shows a few very small clusters (Supplementary Figure S26B). We identified two such small clusters: one has twelve CDRH3s that have an identical amino acid sequence [AGGSGISTPMDV, named Group A (GA)] and the other has eleven CDRH3s that also have an identical amino sequence [AKDGGKLWVYYFDY, named Group B (GB)] (Supplementary Figure S26C). The pairwise structure comparisons demonstrated a low similarity on average for CDRH3 (0.883%) and a relatively high average similarity for the heavy chains (25.8%) (Supplementary Figure S26D). GA and GB have structural similarities of 21.2% and 24.5%, respectively (Supplementary Figure S33D) even GA has the same amino acid sequences as well as GB (Supplementary Figure S26C). We performed similar analyses of the light chains and CDRL3s (Supplementary Figure 27a–27d). CDRL3s have a low structural similarity (1.06%) (Supplementary Figure S27D) and three small clusters: CA, CD and DN were identified (Supplementary Figure S27C). Each of the three clusters (CA, CD and DN) has the same amino acid sequence but different structures (Supplementary Figures S27C, D). It is well-known that CDRH3 and CDRL3 are highly diverse. The TSR keys can quantify such structural diversities.

Besides quantifying structural differences, TSR keys can also be used to interpret the clustering results. To do so, we calculated *common* and *specific* keys. We could not find *common* keys for CDRH3 (Supplementary Figure S28) or CDRL3 (Supplementary Figure S29), further demonstrating their high structural diversities. As expected, we have identified common substructures of GA, GB, CA, CD and DN because the samples in each group have the same amino acid sequences (Supplementary Figures S28, S29). One *specific* key and eleven *specific* keys were identified for GA and GB, respectively (Supplementary Figure S30A). One representative triangle for the *specific* key (5960137) exclusively belonging to GA is shown in Supplementary Figure S30B. This key is constituted from Pro100A, Met100B and Asp101 of CDRH3 that have a close interaction with Lys386 and Ser383 of the spike (Supplementary Figure S30B). Two *specific* keys were identified for CA and no *specific* keys were identified for CD and DN (Supplementary Figure S31).

**FIGURE 9:**
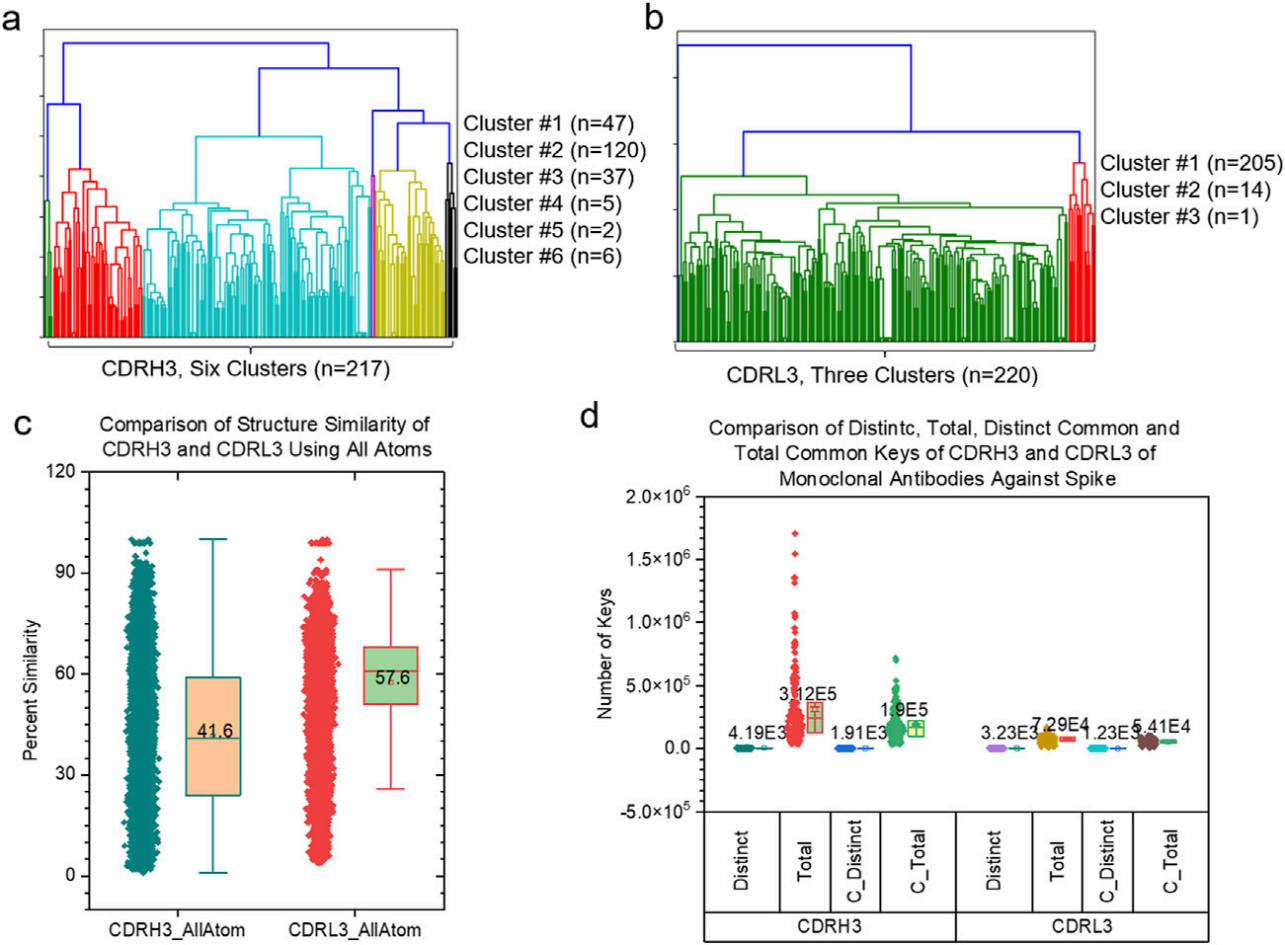
The side-by-side comparison of CDRH3 and CDRL3 using hierarchical clustering analyses and different types of TSR keys are shown. **(a, b)** The hierarchical clustering analyses of CDRH3 **(a)** and CDRL3 **(b)** using TSR CATOM keys. Numbers of CDRH3 **(a)** and CDRL3 **(b)** and the numbers of the CDRH3 **(a)** and CDRL3 **(b)** clusters and numbers of CDRH3 **(a)** and CDRL3 **(b)** in each cluster are indicated; **(c)** The pairwise structural similarity of CDRH3 and CDRL3 studied in **(a, b)** was calculated and is presented. **(d)** The numbers of distinct, total, distinct common and total common CATOM keys of CDHR3 and CDRL3 studied in a-c were calculated and are presented; **(c, d)** The average values are labeled.

We have shown high structural diversities of the backbones of CDRH3 and CDRL3 using CA keys. We wanted to know the similarity and difference of the entire structures of CDRH3 compared with the entire structures of CDRL3. Thus, we have developed a new version of the TSR algorithm using all atoms. We name such keys as ATOM TSR keys. The hierarchical cluster studies show six clusters of CDRH3 (Figure 9A) and three clusters of CDRL3 (Figure 9B). The CDRH3 entire structures represented by ATOM TSR keys are more similar (Average structure similarity: 41.6%) (Figure 9C) than the backbone structures represented by CA keys (Average structure similarity: 0.883%) (Supplementary Figure S26D). A similar scenario was observed for CDRL3 (57.6% for CDRL3 entire structures vs. 1.06% for backbone structures) (Figure 9C and Supplementary Figure S26D). The common substructures represented by *common* keys were identified for both CDRH3 and CDRL3 (Figure 9D). Combining the results using CA and ATOM TSR keys, we show that the backbones of CDRH3 (CDRL3) are highly diverse and CDRH3 (CDRL3) share a significant portion of similar substructures.

### 3.5 Development of a new version of the TSR algorithm for quantifying amino acid structures

#### 3.5.1 Introduction of the new TSR algorithm for quantifying amino acid structures

**FIGURE 10:**
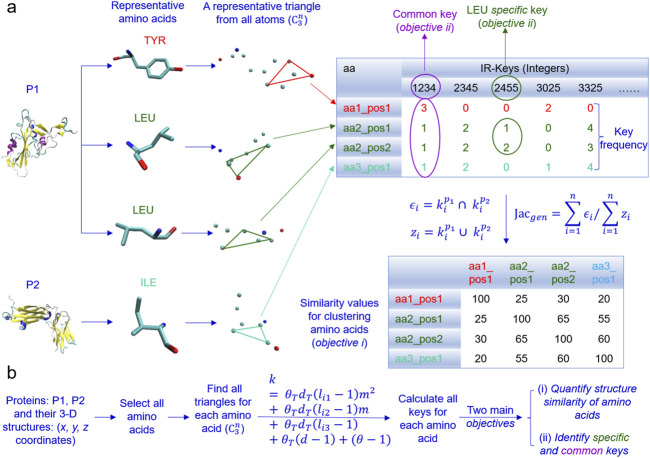
An overview of the design of the IR-TSR algorithm is present. **(a)** The schema of converting amino acid geometries to integers (IR-TSR keys) and calculating pairwise structural similarity using calculated keys and Generalized Jaccard coefficient approach is illustrated. An example of common and specific keys is shown; **(b)** The IR-TSR key generation formula and the main objectives are shown.

In the algorithm, given a dataset, we first select all the atoms of each amino acid of every protein and find all possible triangles formed by all the atoms for each amino acid (Figure 10A). Second, we calculate TSR keys for every triangle using Equation 2 and key occurrence frequencies. Third, we quantify structure similarity or dissimilarity of every amino acid pair using the Generalized Jaccard similarity through computing identical and nonidentical keys and their frequencies (Figure 10A). The approach does not require prior superimposition of amino acid 3D structures and is customized to be invariant to rotation and translation, but sensitive to size of the triangles. We named the keys for amino acids intra-residual (IR) TSR keys. *Common* and *specific* keys can be calculated. The keys present in every amino acid, a certain amino acid (e.g., Tyr) or a certain type of amino acids (e.g., aromatic amino acids) of a given dataset are defined *common* keys and the keys exclusively belonging to a certain amino acid or a certain type of amino acids are defined *specific* keys. The representative ACE2 binding site of the spike contains three Tyr, two Asn, one Gln and one Thr (Figure 5A). If a *specific* key or a *specific* key set (two or more keys) can be identified for a particular Tyr in the ACE2 binding site but not all other Tyr residues across the spike proteins in the dataset, it will provide insight into the role of the Tyr of the spike in ACE2 binding. If a *common* key or a *common* key set can be identified for all three Tyr residues in the ACE2 binding site across the spike proteins, it will also help to understand the general role of the Tyr residue in the ACE2 binding site. The main objectives of the IR-TSR algorithm are to quantify structure similarity and dissimilarity of amino acids and identify *common* and *specific* keys for mechanistic understanding of binding sites or conformational changes (Figure 10B).

#### 3.5.2 Evaluation of the IR-TSR algorithm

**FIGURE 11:**
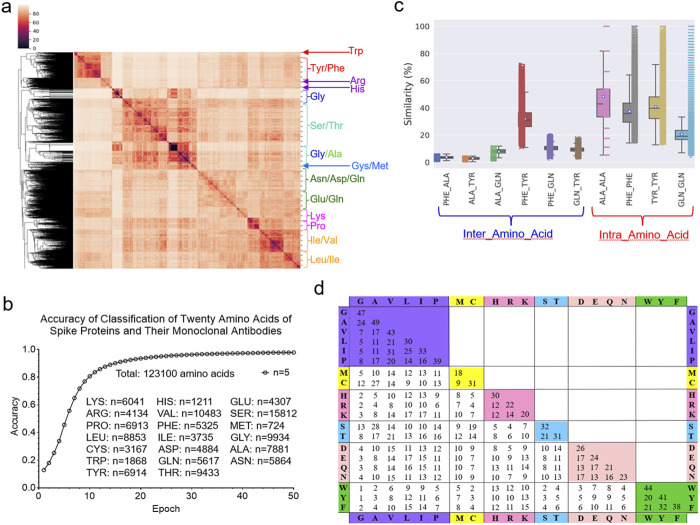
The IR-TSR-based method can quantify structural similarity of twenty different amino acids. **(a)** The hierarchical clustering analysis of the twenty amino acids from the representative proteins. The major clusters are labeled; **(b)** The accuracy increase vs. epoch of the classification study using the 6-layer fully connected neural network are present. The numbers of each type of amino acids and a total of amino acids are indicated. The classification was independently repeated for five times and the average is shown; **(c)** The selected pairwise structural similarities are shown. Intra amino-acid structural similarity means the pairwise structural compassion between same amino acids at different positions or different polypeptides. Inter amino-acid structural similarity means the pairwise structural compassion between different amino acids; **(d)** The pairwise structural similarities between twenty different amino acids were calculated from a total of 123,100 amino acids and the average values are presented, **(b, c)** The number for each type of the amino acid (c) is the same as that presented in **(b)**.

To evaluate the performance of the IR-TSR algorithm, we will focus on (i) the clustering and classification of twenty different amino acids and (ii) more detailed studies of Tyr, Leu and Ile. The hierarchical cluster analysis demonstrates that the IR-TSR algorithm can distinguish different categories of amino acids (e.g., aromatic, alcohol-containing, amide-containing/acidic, basic, sulfur-containing, small aliphatic), but as expected, cannot separate all twenty different amino acids (Figure 11A). For instance, we observed a cluster containing all aromatic amino acids (Tyr, Phe and Trp). However, some Tyr and Phe are grouped together (Figure 11A). A similar scenario was observed for the MDS analysis (Supplementary Figure S32). In contrast to the cluster study, the classification study shows that the ML approach can distinguish the twenty amino acids (Figure 11B; Supplementary Figure S33). The representative pairwise structure similarities are shown in Figure 11C. As expected, we observed that intra amino acids (between same amino acids) have higher structure similarities on average than inter amino acids (between different amino acids) (Figure 11C). We calculated the IR-TSR keys for all the amino acids of the spike proteins as well as the heavy and light chains. Based on those TSR keys from a total of 123,100 amino acids, we generated a chart showing the pairwise structure similarity of twenty amino acids (Figure 11D). We named this chart TSR-STRSUM. The highest structure similarity was observed for the Ala-Ala pairs and the lowest structure similarity was found in the Gly-Trp and Gly-Tyr pairs (Figure 11D). The lowest intra residue structure similarity was observed in the Met-Met pairs (Figure 11D; Supplementary Figure S34). The popular amino acid substitution matrices for protein sequence alignments are BLOSUM matrices where the substitution score is based on the rates at which various amino acids in proteins are being substituted by other residues over time, which is done by counting the relative frequencies of amino acids and their substitution probabilities (Trivedi and Nagarajaram, 2020). Sequence alignments make use of amino acid substitution matrices to discover structural, functional, and evolutionary relations of proteins. TSR-STRSUM provides an alternative way, that is structure-based, of using amino acid substitution matrices for sequence alignment and structural comparison.

**FIGURE 12:**
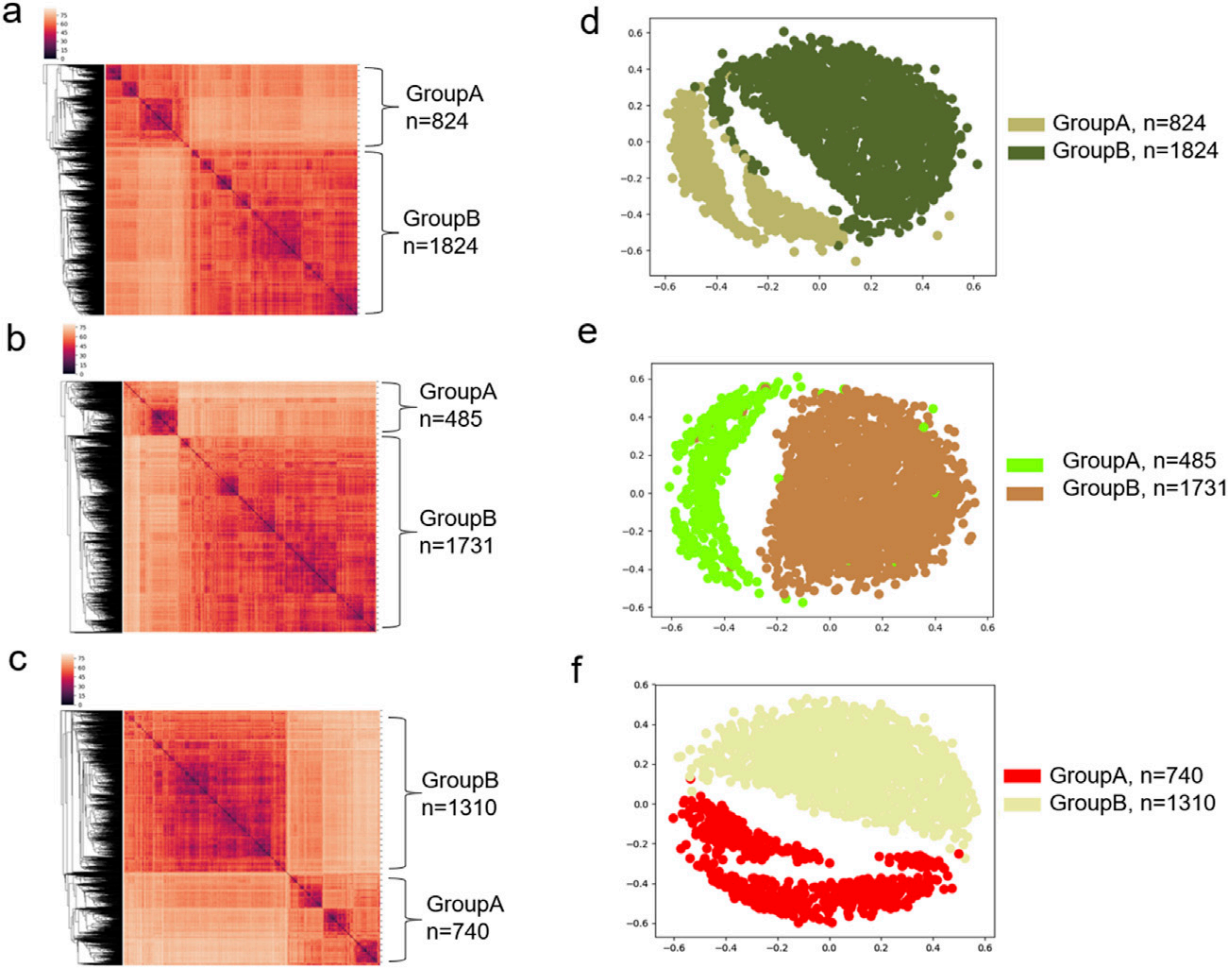
The hierarchical clustering and MDS analyses demonstrate two major clusters of tyrosine residues. **(a–c)** The hierarchical clustering results of the spike proteins **(a)**, and heavy chains **(b)** and light chains **(c)** of the mAbs against spike are shown; **(d–f)** The MDS results of the spike proteins **(d)**, and heavy chains **(e)** and light chains **(f)** of the mAbs against spike are shown; **(a–f)** Major clusters and numbers of the tyrosine residues in each cluster are labeled.

Because the most abundant amino acid in the ACE2 binding site is Tyr, we next focused on the more detailed evaluations of Tyr, Leu and Ile. The hierarchical cluster study shows two clusters of all Tyr residues from the spike proteins (Figure 12A). We also observed two Tyr clusters of the mAb heavy chains (Figure 12B) and the light chains (Figure 12C). The MDS analyses confirmed the two Tyr clusters for the spike proteins (Figure 12D), the heavy chains (Figure 12E) and the light chains (Figure 12F). We observed two Tyr clusters using either the hierarchical (Supplementary Figure S35A) or MDS (Supplementary Figure S35B) approach when we combined Tyr residues from the spike proteins and the heavy and light chains. The classification study confirmed the clustering result of two Tyr clusters (Supplementary Figures S35C, D). Representative Tyr residues from the two clusters are shown in Supplementary Figures S36A–E. The Tyr residues of the spike proteins and heavy and light chains have a total of 718 distinct IR-TSR keys (Supplementary Figure S37). The Tyr residues from each polypeptide have similar numbers of distinct keys (Supplementary Figures S38A–C). Only a small number of *specific* keys were identified exclusively for the spike proteins and the heavy and light chains (Supplementary Figure S39). The common substructures were identified for the spike proteins, the heavy chains and light chains (Supplementary Figure S40).

Leu and Ile are structural isomers (i.e., identical elemental composition and molecular weight). They will have the same number of IR-TSR keys. Therefore, we performed a more detailed study on Leu and Ile. The Leu residues of the spike proteins, the heavy chains and light chains form two clusters (Supplementary Figure S41). In contrast, we observed four Ile clusters (Supplementary Figure S42). The Ile residues have a slightly higher structure similarity on average than the Leu residue (Supplementary Figure S43). As predicted, the Leu-Ile pairs have a lower structure similarity than the Leu-Leu and Ile-Ile pairs (Supplementary Figure S43). Although the clustering technique (unsupervised ML algorithm) cannot distinguish Leu from Ile (Figure 11A), the classification approach (supervised ML algorithm) can distinguish Leu and Ile (Supplementary Figures S44A, B). It suggests that a training process using a set of predefined class labels is required for distinguishing Leu and Ile.

### 3.6 Study steric effects of the representative mAbs drugs on ACE2 binding site

Sotrovimab (Kd = 0.21 nM, Class 4) is a non-RBM mAb drug (Supplementary Figure S45) with the potential to block viral entry into healthy cells and clear infected cells (Miguez-Rey et al., 2022; Aggarwal et al., 2022). One advantage of non-RBM mAbs is that they can tolerate mutations. It has been demonstrated that sotrovimab retained activity against variants of interest and concern, including the alpha, beta, gamma, delta, and lambda variants *in vitro* (Aggarwal et al., 2022; Gupta et al., 2021). In contrast, many of the other mAbs bind to the RBM that engages ACE2; this is one of the most mutable and immunogenic regions of the virus, and in some cases, these mAbs do not retain activity against the variants (Gupta et al., 2021). Bamlanivimab (Kd = 1.5 nM) (Miguez-Rey et al., 2022) and Class 3 (Focosi et al., 2022) was developed by Eli Lilly after its discovery by researchers at AbCellera Biologics and at the Vaccine Research Center of the National Institute of Allergy and Infectious Diseases (Dougan et al., 2021). The study demonstrated that bamlanivimab plus etesevimab led to a lower incidence of COVID-19-related hospitalization and death (Dougan et al., 2021) and a significantly lower proportion of patients with persistently high viral load (Patel et al., 2024; Nichols et al., 2024). Cilgavimab [RBM class II (Focosi et al., 2022)] and tixagevimab [RBM class III (Focosi et al., 2022)] are used together to prevent the virus from binding to ACE2 and entering human cells (Suribhatla et al., 2023) through binding to independent segments of the SARS-CoV-2 spike protein. The clinical data demonstrated that they prevent COVID-19 complications in at-risk patients (Al-Obaidi et al., 2023; Chen et al., 2023). The data obtained from sotrovimab, bamlanivimab, cilgavimab and tixagevimab indicated the steric effect on ACE2 binding site. However, such steric effects have not been structurally quantified.

#### 3.6.1 Study steric effects of the representative mAbs on ACE2 binding site using CA- and ATOM-TSR keys

To evaluate the potential application of the TSR algorithm in quantifying steric effect, we focused on four mAb drugs: sotrovimab, bamlanivimab, cilgavimab and tixagevimab and seven residues in the ACE2 binding site, which consisted of three Tyr (Tyr449, Tyr501 and Tyr505), two Asn (Asn487, Asn501), one Gln (Gln493) and one Thr (Thr500) (Figure 5A). It is worth noting that more mAbs and other residues in the ACE2 can be included in the study. Before discussing the ACE2 binding site, we will provide a brief introduction to overall structures of sotrovimab, bamlanivimab, cilgavimab and tixagevimab and their corresponding spike proteins. For the ACE2 binding site, we will focus on the discussion on the ACE2 binding site using CA-TSR and ATOM-TSR keys (this section) as well as IR-TSR keys (next section).

The cluster analysis shows that the heavy chains of six sotrovimab structures are grouped together (Supplementary Figure S46). The heavy chains of two mAbs: CV05-163 (Class 2) (Yuan et al., 2021) and PDI 96 (Class 6) (Wheatley et al., 2021) are structurally similar to the heavy chains of sotrovimab (Supplementary Figure S46). The heavy chains of cilgavimab and tixagevimab are clustered together (Supplementary Figure S46) because both are smaller than the heavy chains of sotrovimab and bamlanivimab. The heavy chain of bamlanivimab is structurally similar to the heavy chains of sotrovimab (Supplementary Figure S46). The heavy chains of two mAbs: Beta-47 and 15033-7 are structurally similar to the heavy chain of bamlanivimab (Supplementary Figure S46). A similar result was obtained from the cluster analysis of the light chains of sotrovimab, bamlanivimab, cilgavimab and tixagevimab (Supplementary Figure S47). The light chain of CV30 is similar to that of sotrovimab and the light chains of C002 and CV38-142 are similar to those of bamlanivimab (Supplementary Figure S47). We were able to identify the *specific* CA and CCA keys for the heavy and light chains of sotrovimab, bamlanivimab, cilgavimab and tixagevimab (Supplementary Figure S48). The clustering result for the spike proteins with which sotrovimab, bamlanivimab, cilgavimab and tixagevimab interact is shown in Supplementary Figure S49.

We have introduced the overall structures of sotrovimab, bamlanivimab, cilgavimab and tixagevimab (last section). We will now discuss the overall structure of the spike - heavy chain complex and the spike - light chain complex as well as the interfaces between the spike and the heavy chain and between the spike and the light chain. The overall structural relationships of the spike - heavy chain complexes and the spike - light chain complexes are shown in Supplementary Figures S50, S51, respectively. As expected, all six spike - sotrovimab complexes are clustered together (Supplementary Figures S50, S51). The interfaces between the spike and the heavy chain and between the spike and the light chain are related to the functions. Using a similar approach, we evaluated the interfaces using CCA-TSR and CATOM-TSR keys. The clustering result using CCA keys shows that six spike - sotrovimab (heavy chain) interfaces are similar (Supplementary Figure S52). The interfaces of spike - bamlanivimab, spike - cilgavimab and spike - tixagevimab (heavy chain) are different (Supplementary Figure S52). The interface of spike - AZD8895 heavy chain is similar to that of spike - tixagevimab heavy chain using CCA keys (Supplementary Figure S52) and CATOM keys (Supplementary Figure S53). One spike - sotrovimab (heavy chain) interface (PDB: 7L0N) is different from the rest of the five interfaces when CATOM keys are used (Supplementary Figure S53). We found that the interfaces of spike - AZD1061 and spike - Fab06 (heavy chains) are similar to that of the spike - cilgavimab heavy chain, the interface of spike - EY6A (heavy chain) is similar to that of spike - bamlanivimab heavy chain and the interfaces of spike - MW01 and spike - nCoV617 (heavy chains) are similar to those of most of the spike - sotrovimab heavy chain pairs (Supplementary Figure S53). The interfaces of the six spike - sotrovimab light chain pairs are similar using both CCA (Supplementary Figure S54) and CATOM (Supplementary Figure S55) keys. We observed that the interfaces of both the spike - bamlanivimab and spike - cilgavimab are similar and the interface of spike - THSC20 interface resembles that of spike - tixagevimab (light chains and CATOM keys) (Supplementary Figure S55). The *specific* CCA keys (Supplementary Figure S55) and *specific* CATOM-key set (Supplementary Figure S56) for the interfaces between the spike proteins and heavy and light chains of sotrovimab, bamlanivimab, cilgavimab and tixagevimab were identified.

**FIGURE 13:**
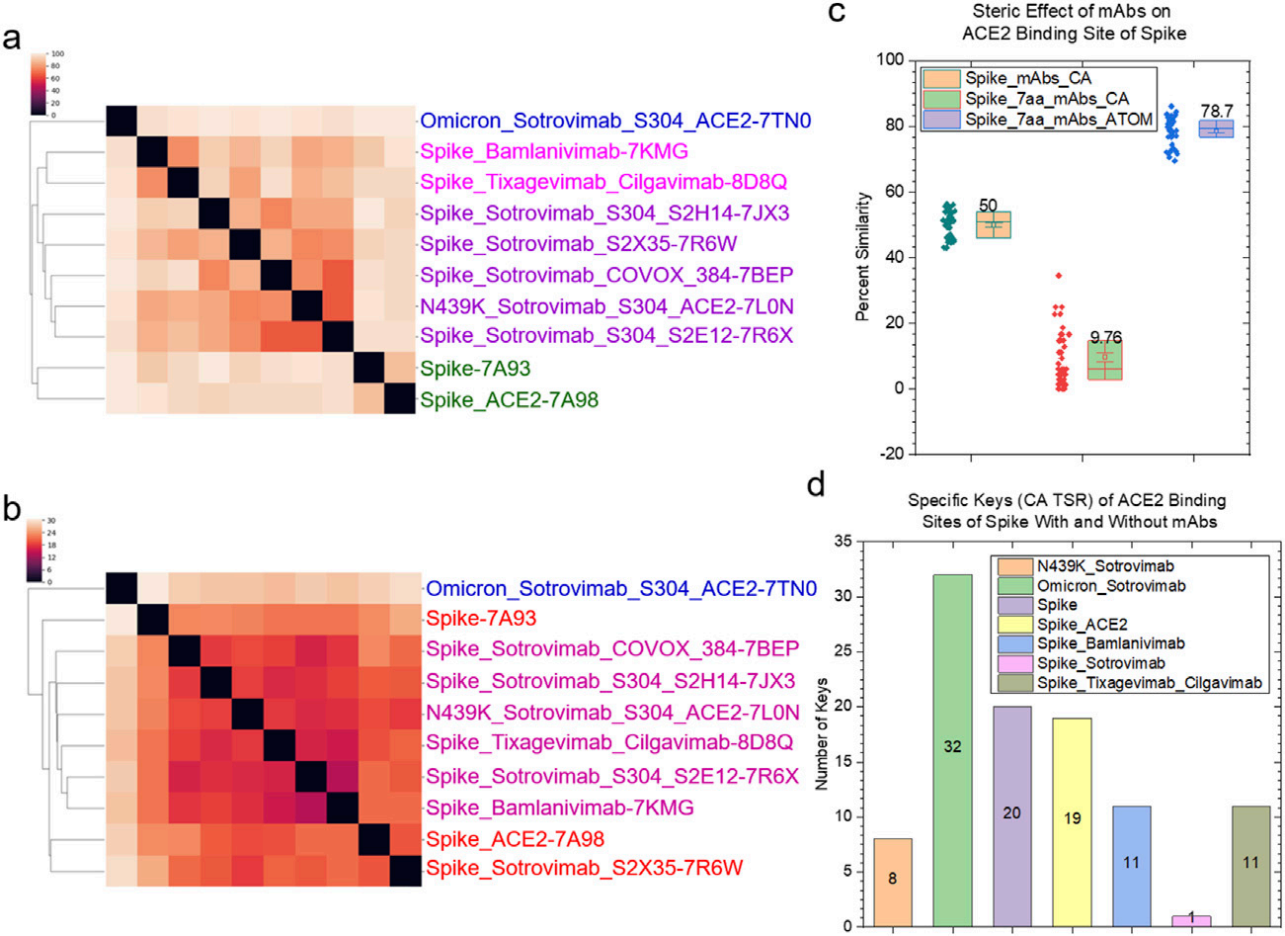
The steric effect of binding of the selected mAbs approved by FDA is shown. **(a, b)** The hierarchical clustering analyses show the structural differences of the spike regions that closely interact with ACE2 upon the binding of the selected mAbs using CCA **(a)** and CATOM keys **(b)**. The mAbs approved by FDA are indicated; **(c)** The structural similarities of the spike regions that closely interact with ACE2 quantified using the CA TSR keys; **(d)** The numbers of the specific CA-TSR keys of the spike proteins against each mAb were calculated and are presented; **(c, d)** Average values are labeled.

The structural relationships of spike proteins, heavy and light chains of mAbs, and spike - mAb complexes we have discussed in the last paragraphs will help to understand the steric effect of mAbs on the ACE2 binding sites. The backbone structures (CCA keys) of the seven amino acids in the ACE2 binding sites of the spike proteins without or with ACE2 or mAbs are different (Figure 13A). The binding of sotrovimab to the spike proteins has a similar steric effect on the ACE2 binding sites (Figure 13A). Bamlanivimab has a similar steric effect as cilgavimab plus tixagevimab (Figure 13A). Two of the seven amino acids were mutated in the omicron variant where Asn510 was changed to Tyr501 and Gln493 was mutated to Arg493. This omicron variant has a greater difference from the rest of the spike proteins with mAbs. The backbone structures of the seven amino acids of the spike proteins with mAbs are different from those of the spike only and the spike with ACE2 (Figure 13A), demonstrating the steric effect due to the binding of mAbs. In contrast to the backbone structures, the overall steric effect quantified using the CATOM keys caused by the binding of mAbs becomes smaller (Figure 13B). We calculated the pairwise structural similarities using CA keys for the entire spike proteins and using CA and CATOM keys for the seven amino acids in the ACE2 binding sites. The average structure similarity of the seven amino acids using CATOM is greater than that using CCA keys (Figure 13C). The average structure similarity of the entire spike proteins using CA keys is larger than the similarities of the seven amino acids using CCA keys, but it is smaller than those using CATOM keys (Figure 13C). The *specific* CCA (Figure 13D) and CATOM (Supplementary Figure S57) keys were identified for the ACE2 binding sites of the spike, N439K variant or omicron variant with or without sotrovimab, bamlanivimab, cilgavimab and tixagevimab. We could not find *common* CCA keys for the seven amino acids that were identified, which suggests a high diversity of the backbone structures. In contrast, we succeeded in identifying *common* CATOM keys (Supplementary Figure S58). It demonstrates that the ACE2 binding sites share a significant amount of common substructure.

#### 3.6.2 Study steric effects of the representative mAbs on ACE2 binding site using IR-TSR keys

**FIGURE 14:**
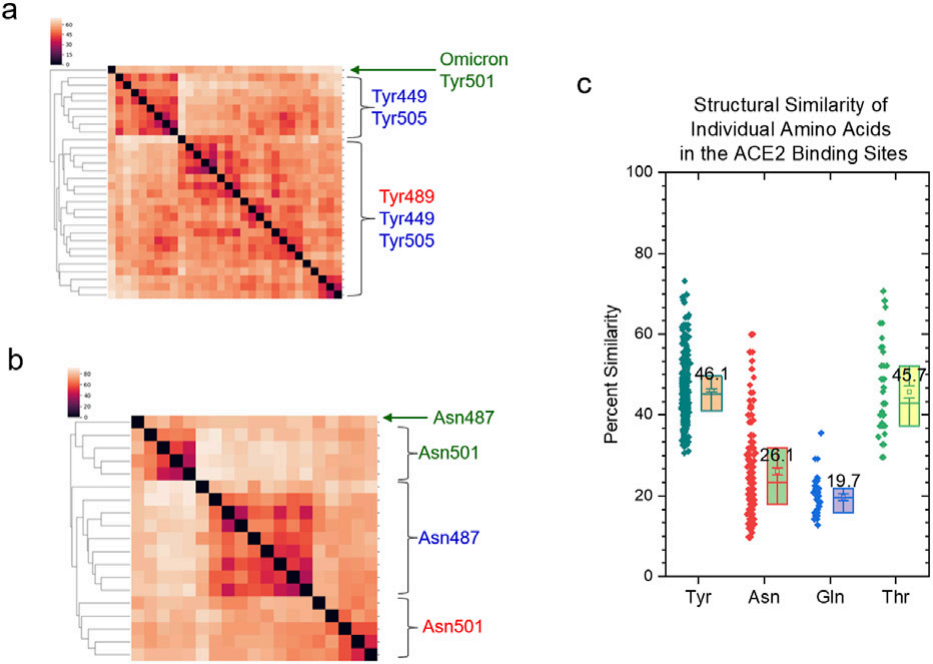
The amino acid position-dependent or partial amino acid position-dependent clusters are present. **(a, b)** The clustering analyses of the tyrosine **(a)** and asparagine **(b)** residues in the ACE2 binding sites of the selected spike proteins against the FDA approved mAbs drugs. Amino acid positions and SARS-CoV-2 variant are labeled; **(c)** The pairwise structural similarities of the tyrosine, asparagine, glutamine and threonine residues in the ACE2 binding sites were calculated and are presented. The average values are labeled.

We have discussed the steric effects of mAb binding on the ACE2 binding site from the angles of the backbone and overall structures. In this section, we will discuss the steric effects at the individual amino acid level (three Tyr, two Asn, one Gln and one Thr). The most abundant amino acid in the spike proteins is Asn (Supplementary Figure S59) and the most abundant amino acid in the mAb heavy (Supplementary Figure S60) and light (Supplementary Figure S61) chains is Ser. Tyr is the most abundant residue in the ACE2 binding site. All Tyr residues have different structures, and no amino acid position-dependent clusters are found (Figure 14A). Tyr501 from the omicron variant is clustered separately from other Tyr residues (Figure 14A). We observed amino acid position-dependent clusters for the Asn residues. The Asn501 residues form two clusters and most Asn487 except for Asn487 from the spike alone (with an interacting polypeptide) form one large cluster (Figure 14B). The Gln493 (Supplementary Figure S62) and Thr500 (Supplementary Figure S63) residues are structurally different. We could not find a particular mAb-dependent cluster for either Gln or Thr. The ordering of structural diversity from high to low is Gln > Asn > Thr > Tyr (Figure 14C). The tyrosine side chain has a ring structure that is ridge, thus the Tyr residues are more similar than Thr, Asn and Gln. It was reported that tyrosine side chains were capable of mediating most of the contacts necessary for high-affinity antigen recognition (Fellouse et al., 2004). The structural uniqueness of Tyr may contribute to the molecular recognition, especially between antigens and antibodies.

## 4 Discussions and future directions

It is estimated that more than 5,000 mAb structures were deposited in the PDB. Among these 5,000 mAbs, ∼200 mAbs against the spike were investigated in this study. This study has two main objectives: (i) to develop new computational methods and software tools and (ii) to achieve discoveries and provide insight into spike - mAbs interaction. We focused more on the method and tool development, and evaluation. Once the methods are developed, the associated tools will be available for the investigators to study the mAbs (e.g., gp120 of HIV) or other proteins of interest to accelerate the antibody drug development. This small-scale study shows that the heavy chains of the mAbs against gp120 are structurally different from their corresponding light chains (Supplementary Figure S64). The heavy chains of the mAbs against the spike and gp120 are grouped together into a large cluster, and the same thing also happens with the light chains (Supplementary Figure S65). However, the heavy chains of the mAbs against the spike are not separated from those against gp120 (Supplementary Figure S65), suggesting that some heavy chains of the spike and gp120 mAbs are structurally more similar than those of the spike mAbs or those of the gp120 mAbs. A similar situation was observed for the light chains of the mAbs against the spike and gp120 (Supplementary Figure S65). A comprehensive study of all mAbs and CDRs (CDRH1, CDRH2, CDRH3, CDRL1, CDRL2 and CDRL3) will provide insight into antigen-antibody interactions. Annotation of such a dataset will need considerable amount of effort. A recent study of 1,456 structures has led to the discovery of previously unrealized interfaces: β-sheet dimers and variable-constant elbow dimers, among antibodies (Yin et al., 2022).

**FIGURE 15:**
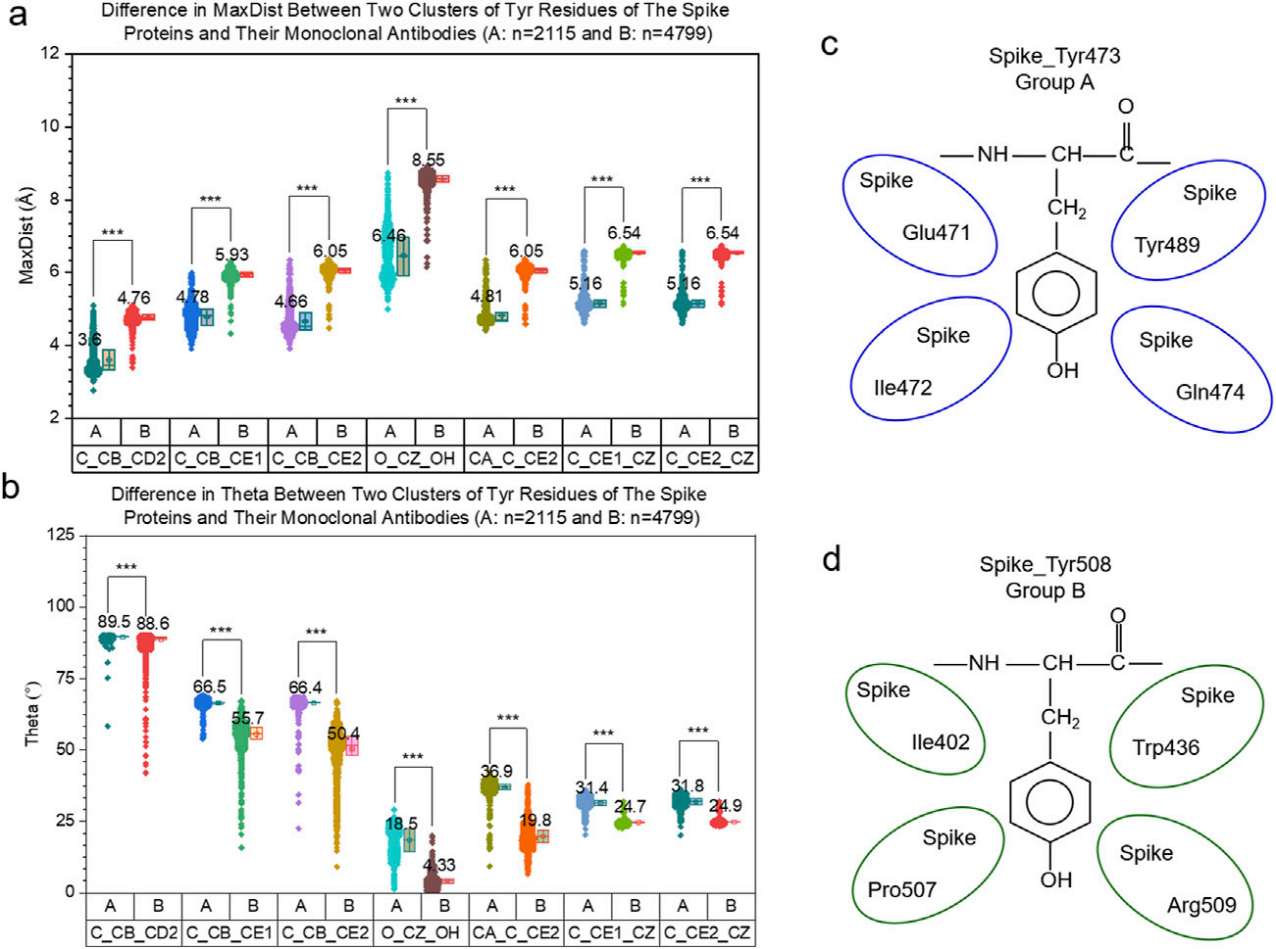
The difference in geometry of two tyrosine clusters are present. **(a, b)** The difference in MaxDist **(a)** and Theta **(b)** of the two clusters of the tyrosine residues are shown. Numbers of the tyrosine residues in each group are indicated. Average values are labeled. The *t*-test analyses were used and *** means a *p* value < 0.001; **(c, d)** Examples of protein environments that determine the tyrosine clusters A **(c)** and B **(d)** are present.

We observed two Tyr clusters from spike proteins, heavy chains and light chains both alone and combined. To demonstrate whether the two Tyr clusters are dependent on the dataset, we included different types of proteins (protein receptors). The hierarchical cluster study of protein receptors shows the two Tyr clusters (Supplementary Figure S66A) and the MDS study agrees with the hierarchical clustering result (Supplementary Figure S66B). The additional Tyr study from the protein receptor family suggests the two clusters could be independent on datasets. To show the difference in the geometry of the Tyr residues of the spike proteins and their mAbs from the two clusters, we calculated the MaxDist and Theta values. The MaxDist distance calculations show that the triangles constituted from (C, CB and CD2), (C, CB and CE1), (C, CB and CE2), (O, CZ and OH), (CA, C and CE2), (C, CE1 and CZ) and (C, CE2 and CZ) have larger MaxDist values in one group and smaller MaxDist values in another (*t*-test, *p* < 0.001) (Figure 15A). The triangles with larger MaxDist values have smaller Theta values (*t*-test, *p* < 0.001) (Figure 15B). For ease of discussion, we define the group with the smaller MaxDist and larger Theta values as Group A. The group with larger MaxDist and smaller Theta values is defined as Group B. The same scenario for MaxDist (Supplementary Figure S67) and Theta (Supplementary Figure S68) was observed from the Tyr residue of the protein receptors. To further demonstrate whether the local protein environments determine whether a Tyr residue will belong to Group A or B, we found that a high percentage of Tyr351, Tyr423, Tyr453 and Tyr473 of the spike belong to Group A while a high percentage of Tyr451, Tyr489, Tyr495 and Tyr508 belong to Group B. The top four residues with a high percentage of the amino acids surrounding Tyr473 (Figure 15C) and Tyr508 (Figure 15D) residues are shown in Figure 15. Similar analyses show the local environments for Tyr351, Tyr423 and Tyr453 of Group A (Supplementary Figure S69) and those for Tyr451, Tyr489 and Tyr495 of Group B (Supplementary Figure S70). The local environment study indicates that the cluster assignment of Tyr is dependent on amino acid position. Therefore, we reasonably hypothesize that the protein’s local environments determine the cluster to which a Tyr residue belongs. Although we have examined a few of the representative protein environments in determining the clusters of Tyr, we cannot induce a general rule.

CCAI-TSR or CATOM-TSR keys are specifically designed for quantifying interfaces between two molecules. We observed that the structure similarity using CATOM-TSR keys is higher than that using CCAI-TSR keys. For CCA-TSR keys, we assign each C_α_ atom an integer. Two triangles will have different keys even though they have similar or identical shapes if one or more amino acids between the two triangles are different. For CATOM-TSR keys, the atoms carbon, nitrogen, oxygen, and sulfur are assigned different integer labels during the key computation. However, we did not consider atom types. For example, we assign the same integer to a C_α_, a carbonyl carbon, a carbon in the carboxylic group, and a carbon linked to a hydroxyl group. We will, in the future, include atom types in the CATOM key generation formula.

One of the advantages of the TSR algorithms is that the integer nature of data structure allows ML-based algorithms developed in the AI field to be easily adapted for prediction purposes. We used a 6-layer fully connected neural network for classifying proteins using CA-TSR keys or amino acids using IR-TSR keys. We will integrate CA-TSR keys with IR-TSR keys for further discretizing global and local structures. The data structure for individual proteins or amino acids is a vector of integers. The data structure, therefore, will be a matrix when we integrate CA-TSR and IR-TSR keys for each protein. Integrating both keys will enable us to distinguish finer differences between ligand or substrate binding sites and eventually use a ML approach to predict binding sites.

## 5 Conclusions

The two objectives of this study are to introduce a new computational methodology and provide mechanistic understanding of spike - mAb interactions. From the methodology perspective, *cross* TSR keys using C_α_ atoms (CCA) and all atoms (CATOM) and *intra-residual* (IR) TSR keys were developed specifically for defining and quantifying structural characteristics of protein binding sites. From the perspective of method evaluation and mechanistic understanding of spike - mAb interactions, key findings are summarized. (i) *Specific* CCAI keys were exclusively identified for Class 1 mAbs, (ii) Six clusters were identified for CDRH3 and three clusters were found for CDRL3, (iii) The steric effects of binding of mAb drugs: sotrovimab, bamlanivimab, cilgavimab and tixagevimab on the ACE2 binding site were quantified, (iv) Asn487 residues of the spike interacting with ACE2, sotrovimab, bamlanivimab, cilgavimab or tixagevimab have their specific structural characteristics, (v) A new structure-based matrix, TSR-STRSUM, is introduced as an alternative way, instead of using BLOSUM, for protein sequence and structure comparison, (vi) IR-TSR keys demonstrated two clusters of Tyr structures.

## Data Availability

The datasets presented in this study can be found in online repositories. The names of the repository/repositories and accession number(s) can be found in the article/Supplementary Material.
