# Supplementary material for: Development of the TSR-based computational method to investigate spike and monoclonal antibody interactions
Source: Front Chem. 2025 Mar 19;13:1395374. doi: 10.3389/fchem.2025.1395374 (PMC11962798; doi:10.3389/fchem.2025.1395374)
Supplement: Supplementary file 1 [file DataSheet1.pdf]

Supplementary Figure Legend

Supplementary Figure 1. The mechanism of how a mAb works is illustrated. The mAb can bind ACE2 binding site of spike and compete with ACE2 for the binding to spike. If the binding of such mAb has a higher binding affinity, it will eventually replace ACE2 or block the binding of ACE2. In another scenario, the mAb will bind spike at a different site from the ACE2 binding but the mAb has a long-distance steric effect for altering the structures of the ACE2 binding sites, indirectly weakening ACE2 binding to spike.

Supplementary Figure 2. The amino acid sequence similarities of the spike proteins, and heavy and light chains of the mAbs using the TM-align and Muscle algorithms are present. The TM-align method is based on pairwise sequence comparison while the Muscle approach is based on multiple sequence comparison. Average values are labeled. S_H_L: all spike and heavy and light chains together; S: spike; H: heavy chain of mAbs and L: heavy chain of mAbs.

Supplementary Figure 3. The classification result of the spike proteins and heavy and light chains of the mAbs against spike using the 6-layer fully connected neural network are illustrated. a, The accuracy increase vs epoch of the classification study using the 6-layer fully connected neural network are present; b, The entropy loss vs epoch of the classification study using the 6-layer fully connected neural network are present. a-b, Numbers of the spike proteins and heavy and light chains of mAbs are labeled. The classification was independently repeated for five times and the average is shown.

Supplementary Figure 4. The geometry of the *common* and *uncommon* keys represented using MaxDist and Theta values, key frequencies (occurrences) and percent of the selected amino acids of the spike proteins and heavy and light chains of mAbs are present. a-b, MaxDist and Theta values, key frequencies and valine contents of all polypeptides including the spike proteins and heavy and light chains of mAbs (a) and spike proteins alone (b) are shown; c-d, The MaxDist and Theta values, key frequencies and serine contents of heavy (c) and light (d) chains of mAbs are shown; a-d, The side-by-side comparison between the *common* and *uncommon* keys are shown. Average values are labeled.

Supplementary Figure 5. The Venn diagram shows the structural relationships among the spike proteins and heavy and light chains of the mAbs against spike. Numbers of the total distinct keys and the portions for spike alone, heavy chain alone, light chain alone and the overlapped by three polypeptides are labeled. The percentage for each portion was calculated and is presented.

Supplementary Figure 6. The numbers of the *specific* keys for the spike alone, the spike with ACE2 or mAbs and the spike variants alone or with mAbs are shown. The *specific* keys represent the label- and geometry-based triangles that are unique for the certain spike structures.

Supplementary Figure 7. The representative examples of how mAbs and/or ACE2 interact with spike are illustrated. a, Both ACE2 and CR3022 (heavy and light chains) interact with spike; b, Both CR3022 and CC12.1 interact with spike; c, Both CR3022 and CC12.3 interact

with spike; d, The heavy and light chains of CC12.1 interact with spike; e, The heavy and light chains of CC12.1 interact with spike; a-e, PDB IDs, polypeptides and ACE2 binding sites are labeled.

Supplementary Figure 8. The hierarchical organization of the spike structures with or without an interacting polypeptide is illustrated. Nodes and levels are labeled and color-coded. Numbers of the *specific* keys associated with each node at every level are indicated.

Supplementary Figure 9. The representative examples of the *specific* keys associated with the node of spike complexed with CR3022 and two more polypeptides at the level 4 are shown. a, The CA-TSR key of 7799302 associated triangle is constituted from C361, V362 and C525 of spike; b, The CA-TSR key of 8756479 associated triangle is constituted from K417, R454 and L455 of spike. This triangle is close to G97 of the heavy chain of CC12.3; a-b, PDB IDs are indicated.

Supplementary Figure 10. The hierarchical clustering result of the heavy and light chains of the selected mAbs and ACE2 are present. The heavy and light chains of the CR3022-related mAbs, CR3022 heavy and light chains and ACE2 are labeled.

Supplementary Figure 11. The hierarchical organization of CR3022 heavy chains is illustrated. CR3022 interacting proteins, PDB IDs and the numbers of the *specific* keys are labeled for each structure and each structure cluster.

Supplementary Figure 12. An example of the *specific* keys unique for all the heavy chains of CR3022 is present. PDB ID, amino acids and CA keys are labeled.

Supplementary Figure 13. An example of the *specific* keys unique for heavy chains of CR3022 that do not have an interacting polypeptide is present. PDB ID, polypeptides, amino acids and CA keys are labeled.

Supplementary Figure 14. An example of the *specific* keys unique for heavy chains of CR3022 that have one or more interacting polypeptides is present. PDB ID, polypeptides, amino acids and CA keys are labeled.

Supplementary Figure 15. A side-by-side comparison of the spike regions that interact with either the heavy chains or the light chains of CR3022 is shown. a-b, The hierarchical clustering results show the difference between the spike regions that interact the heavy (a) and light (b) chains of CR3022. PDB ID, CR3022 and other mAbs are indicated; c, It shows side-by-side comparison of structure similarity of the spike region interacting with the heavy chains vs the spike region interacting with the light chains.

Supplementary Figure 16. The multiple sequence alignment shows the difference in amino acid sequences of the CR3022 heavy chains from different PDB entries. PDB IDs are shown.

Supplementary Figure 17. The multiple sequence alignment shows the difference in amino acid sequences of the CR3022 light chains from different PDB entries. PDB IDs are shown.

Supplementary Figure 18. The spike can interact with two mAbs: CR3022 and C099 is illustrated. PDB ID, polypeptides, ACE2 binding site and CR3022 binding site are labeled.

Supplementary Figure 19. A side-by-side comparison of the specific keys for the spike regions that interact with the heavy chains vs those that interact with the light chains is present. mAbs and average values are indicated. The number of the CR3022 structures is eleven, the numbers of the structures for C099, CC12.1 and CC12.3 are two, and the numbers of the structures for BG4, NB_D4 and NB_H11 are one.

Supplementary Figure 20. The

Supplementary Figure 21. The

Supplementary Figure 22. The hierarchical clustering result shows that using CCA keys cannot distinguish different classes of mAbs. Spike-heavy chain pairs and spike-light chain pairs, and classes of mAbs are labeled.

Supplementary Figure 23. The hierarchical clustering result shows that using CA keys cannot distinguish different classes of mAbs. Heavy and light chains and classes of mAbs are labeled.

Supplementary Figure 24. The hierarchical clustering result shows that using CCA keys with applying size filtering can distinguish Class 1 of mAbs from the other classes (2, 3 and 4). The cutoff for size filtering is 10 Å. The clusters for the spike-heavy chain and the spike-light pairs of Class 1 mAbs are labeled.

Supplementary Figure 25. The hierarchical clustering result shows that using CCAI keys can distinguish the heavy chains of Class 1 mAbs from those of the other classes (2, 3 and 4). The cluster for the spike-heavy chain pairs of Class 1 mAbs is labeled.

Supplementary Figure 26. The hierarchical clustering study of the heavy chains and CDRH3 of the mAbs against spike using CA keys. a, The clusters of the heavy chains of the mAbs using the CA keys are illustrated; b, The clusters of the CDRH3s of the mAbs are shown; c, The clusters of the selected CDRH3s of the mAbs are present. The amino acid sequences for groups GA and GB are shown; a-c, Numbers of the structures are labeled; d, The structural similarity of the heavy chains, CDRH3, GA and GB are present. Average values are labeled.

Supplementary Figure 27. The hierarchical clustering study of the light chains and CDRL3 of the mAbs against spike using CA keys. a, The clusters of the light chains of the mAbs using the CA keys are shown; b, The clusters of the CDRL3s of the mAbs are present; c, The clusters of the selected CDRL3s of the mAbs are present. The amino acid sequences for groups CA, CD and DN are shown; a-c, Numbers of the structures are labeled; d, The structural similarity of the light chains, CDRL3, CA, CD and DN is illustrated. Average values are labeled.

Supplementary Figure 28. The numbers of distinct and total *common* CA keys of the heavy chains, CDRH3, GA, GB and GA and GB combined (GA_GB) are present. Average values are labeled.

Supplementary Figure 29. The numbers of distinct and total *common* CA keys of the light chains, CDRL3, CA, CD, DN and CA, CD and DN combined (CA_CD_DN) are shown. Average values are labeled.

Supplementary Figure 30. The specific keys for GA and GB were calculated and are presented. a, Numbers of the *specific* CA keys identified and numbers of structures used for specific key analysis are indicated; b, One specific key for GA is shown. PDB ID, polypeptides, key-associated amino acids and interacting amino acids and key are shown.

Supplementary Figure 31. The specific keys for CA, CD and DN were calculated and are presented. Numbers of the *specific* CA keys identified and numbers of structures used for specific key analysis are indicated.

Supplementary Figure 32. The MDS result of the twenty amino acids is present.

Supplementary Figure 33. The entropy loss vs epoch of the classification study of the amino acids of the spike proteins and their mAbs using IR-TSR keys are illustrated. Numbers of each type of amino acids and total amino acids are indicated. The classification was independently repeated for five times and the average is represented.

Supplementary Figure 34. The structural similarities of the amino acids of the spike proteins and their mAbs using IR-TSR keys are present. The numbers of twenty amino acid can be found here (LYS: n=6041; HIS: n=1211; GLU: n=4307; ARG: n=4134; VAL: n=10483; SER: n=15812; PRO: n=6913; PHE: n=5325; MET: n=724; LEU: n=8853; ILE: n=3735; GLY: n=9934; CYS: n=3167; ASP: n=4884; ALA: n=7881; TRP: n=1868; GLN: n=5617; ASN: n=5864; TYR: n=6914; THR: n=9433).

Supplementary Figure 35. The classification study shows the two clusters of the tyrosine residues of the spike proteins and their mAbs. a, The hierarchical clustering of the tyrosine residues from the spike proteins and their mAbs; b, The MDS analysis of the tyrosine residues from the spike proteins and their mAbs; c, The classification of the tyrosine residues from the spike proteins and their mAbs study shows the accuracy increase vs epoch; d, The classification of the tyrosine residues from the spike proteins and their mAbs study shows the entropy decrease vs epoch; a-d, The tyrosine residues are roughly divided into two groups: Group A and Group B. Numbers of the tyrosine residue in each group are labeled; c-d, The classification analysis was independently repeated for five times and the average values are presented.

Supplementary Figure 36. Examples of the tyrosine structures randomly selected from Group A or Group B are shown. a-e, PDB IDs, Polypeptides, Amino Acids and Groups of tyrosine are labeled.

Supplementary Figure 37. The distribution of distinct keys of the tyrosine residues of the spike proteins and their mAbs is present. Total tyrosine residue number and distinct key number are labeled.

Supplementary Figure 38. The distributions of distinct keys of the tyrosine residues of the spike proteins and the heavy chains and light chains of their mAbs separately are present. a, The spike proteins; b, The heavy chains of mAbs; c, The light chains of mAbs; a-c, Total tyrosine residue number and distinct key number are labeled.

Supplementary Figure 39. The Venn diagram shows the structural relationships of the tyrosine residues from the spike proteins and the heavy and light chains of their mAbs. Numbers of the total distinct keys and the portions for spike alone, heavy chain alone, light chain alone and the overlapped by three polypeptides are labeled. Percentage for each portion was calculated and is presented.

Supplementary Figure 40. The numbers of distinct, total, distinct common and total common IR-TSR keys of the tyrosine residues from the spike proteins and the heavy and light chains of their mAbs were calculated and are present. Average values are labeled. S_H_L means spike, heavy chain and light chain combined.

Supplementary Figure 41. The hierarchical clustering study show two large clusters of the leucine residues from the spike proteins and the heavy and light chains of their mAbs. Total number of the leucine residues is labeled.

Supplementary Figure 42. The hierarchical clustering study show four large clusters of the isoleucine residues from the spike proteins and the heavy and light chains of their mAbs. Total number of the isoleucine residues is labeled.

Supplementary Figure 43. The structural similarity of the leucine and isoleucine residues from the spike proteins, their mAbs and the protein receptors is present. Numbers of leucine and isoleucine residues and total number are labeled.

Supplementary Figure 44. The classification result of the leucine and isoleucine residues of the spike proteins, their mAbs and the protein receptors using IR-TSR keys is present. a, The accuracy increase vs epoch of the classification study using the 6-layer fully connected neural network are present; b, The entropy loss vs epoch of the classification study using the 6-layer fully connected neural network are shown. a-b, Numbers of the leucine and isoleucine residues and the total number are labeled. The classification was independently repeated for five times and the average is shown.

Supplementary Figure 45. The spike of the omicron variant can interact with two mAbs: S304 and S309 and ACE2 are illustrated. PDB ID, polypeptides and ACE2 binding site are labeled. S309 is also named sotrovimab.

Supplementary Figure 46. The hierarchical clustering result of the heavy chains of the mAbs approved by FDA and the heavy chains of some representative related mAbs is shown. PDB IDs and mAbs are indicated. CA-TSR keys are used.

Supplementary Figure 47. The hierarchical clustering result of the light chains of the mAbs approved by FDA and the light chains of some representative related mAbs is present. PDB IDs and mAbs are indicated. CA-TSR keys are used.

Supplementary Figure 48. The specific CA for the heavy and light chains of the mAbs approved by FDA and the specific CCA keys for the spike-mAb complexes were identified and are present. Average values are labeled.

Supplementary Figure 49. The hierarchical clustering result of the spike proteins that interact with the mAbs approved by FDA is present. Two structures: spike alone and spike with ACE2 are included as the references. PDB IDs and mAbs are indicated. CA-TSR keys are used.

Supplementary Figure 50. The hierarchical clustering result of the spike-heavy chain complexes using CCA keys is present. PDB IDs and mAbs are indicated. mAbs approved by FDA and the selected related mAbs are included.

Supplementary Figure 51. The hierarchical clustering result of the spike-light chain complexes using CCA keys is shown. PDB IDs and mAbs are indicated. mAbs approved by FDA and the selected related mAbs are included.

Supplementary Figure 52. The hierarchical clustering result of the spike-heavy chain interfaces using CCAI keys is shown. PDB IDs and mAbs are indicated. mAbs approved by FDA and the selected related mAbs are included.

Supplementary Figure 53. The hierarchical clustering result of the spike-heavy chain interfaces using CATOM keys is shown. PDB IDs and mAbs are indicated. mAbs approved by FDA and the selected related mAbs are included.

Supplementary Figure 54. The hierarchical clustering result of the spike-light chain interfaces using CCAI keys is present. PDB IDs and mAbs are indicated. mAbs approved by FDA and the selected related mAbs are included.

Supplementary Figure 55. The hierarchical clustering result of the spike-light chain interfaces using CATOM keys is present. PDB IDs and mAbs are indicated. mAbs approved by FDA and the selected related mAbs are included.

Supplementary Figure 56. A side-by-side comparison of the *specific* CATOM keys between the spike-heavy chain interfaces and the spike-light chain interfaces is present. Number of the structures and average values are labeled. The specific keyset of two are used.

Supplementary Figure 57. The *specific* CATOM keys were identified for the regions of the spike and its variants with or without mAbs approved by FDA that closely interact with ACE2. The variants and mAbs are indicated. Average values are labeled.

Supplementary Figure 58. The distinct, total, distinct *common* and total *common* CATOM keys were identified for the spike regions that closely interact with ACE2. The spike and its variants with or without mAbs approved by FDA are included. Average values are labeled.

Supplementary Figure 59. The content of twenty amino acids of spike was calculated and is present. Average values are labeled. Number of the spike proteins is indicated.

Supplementary Figure 60. The content of twenty amino acids of the heavy chains of the mAbs was calculated and is present. Average values are labeled. Number of the heavy chains is indicated.

Supplementary Figure 61. The content of twenty amino acids of the light chains of the mAbs was calculated and is present. Average values are labeled. Number of the light chains is indicated.

Supplementary Figure 62. The hierarchical clustering result of the glutamine residues from the spike proteins that interact with FDA-approved mAbs using IR-TSR keys. Glutamine

from spike alone and spike complexed with ACE2 are included in the analysis. Glutamine position, polypeptides and PDB IDs are indicated.

Supplementary Figure 63. The hierarchical clustering result of the threonine residues from the spike proteins that interact with FDA-approved mAbs using IR-TSR keys is present. Threonine from spike alone and spike complexed with ACE2 are included in the analysis. Threonine position, polypeptides and PDB IDs are indicated.

Supplementary Figure 64. The hierarchical clustering result of the heavy and light chains of the mAbs against gp120 of HIV using CA keys is present. Numbers of the heavy and light chains are labeled.

Supplementary Figure 65. The hierarchical clustering result of the heavy and light chains of the mAbs against gp120 of HIV or spike using CA keys is present. Numbers of the heavy and light chains of the mAbs against spike and gp120 are labeled.

Supplementary Figure 66. The hierarchical clustering and MDS analyses demonstrate two clusters of the tyrosine residues from the protein receptors. a, The hierarchical clustering study; b, The MDS analysis; a-b, Numbers of the tyrosine residues in Group A and Group B are labeled.

Supplementary Figure 67. The distance calculations demonstrate the difference in MaxDist of two tyrosine clusters. Numbers of the tyrosine residues in each group are indicated. The

average values are labeled. The *t*-test analyses were used and *** means *p* value < 0.001. The tyrosine residues are from the protein receptor family (The number of the receptors is 1,113).

Supplementary Figure 68. The angle calculations demonstrate the difference in Theta of two tyrosine clusters. Numbers of the tyrosine residues in each group are indicated. The average values are labeled. The *t*-test analyses were used and *** means *p* value < 0.001. The tyrosine residues are from the protein receptor family (The number of the receptors is 1,113).

Supplementary Figure 69. The examples of protein environments that determine the tyrosine cluster A. The tyrosine residues were selected from spike.

Supplementary Figure 70. Examples of protein environments that determine the tyrosine cluster B are illustrated. The tyrosine residues were selected from spike.

Supplementary Figure 1

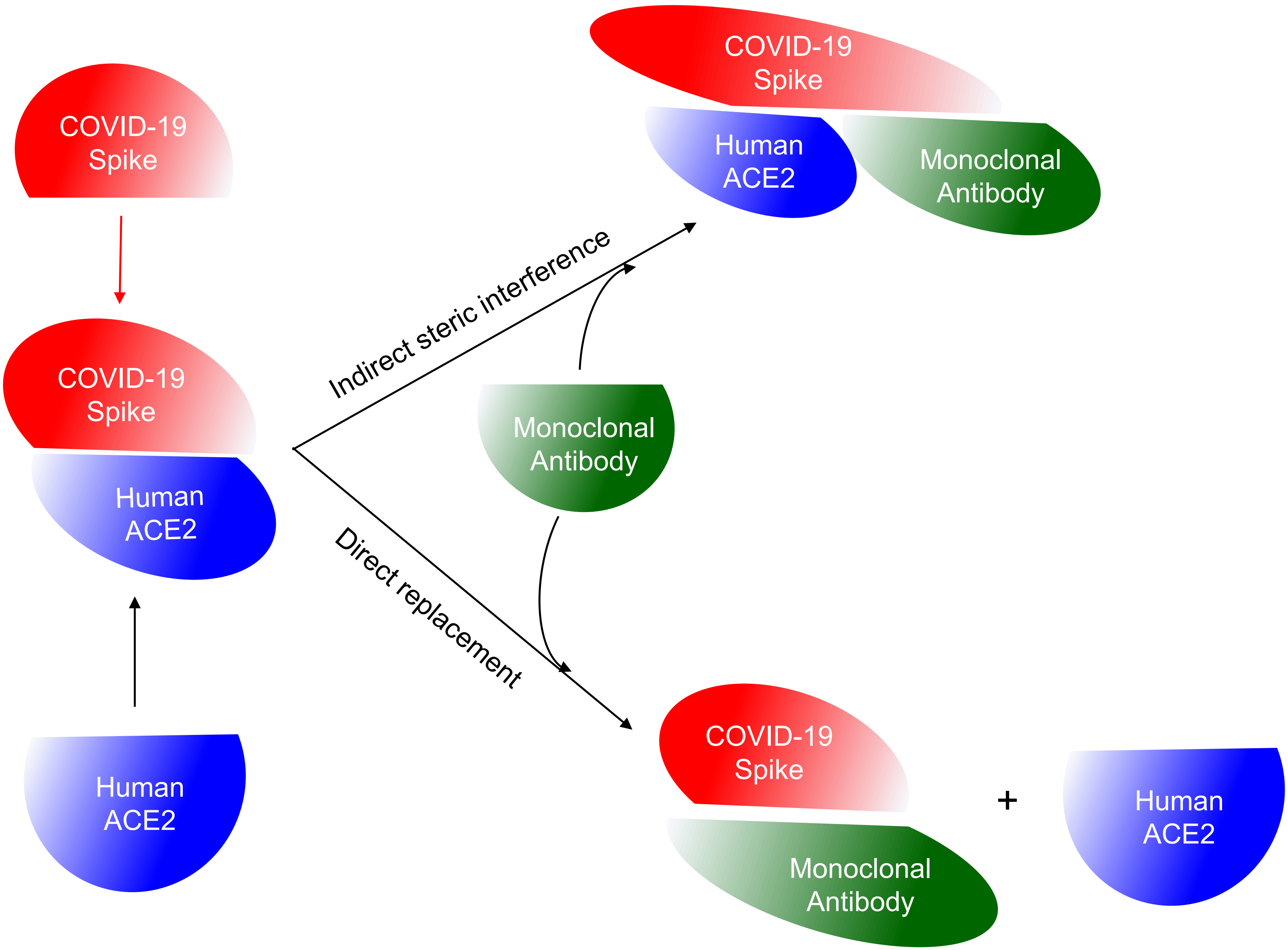

Supplementary Figure 2

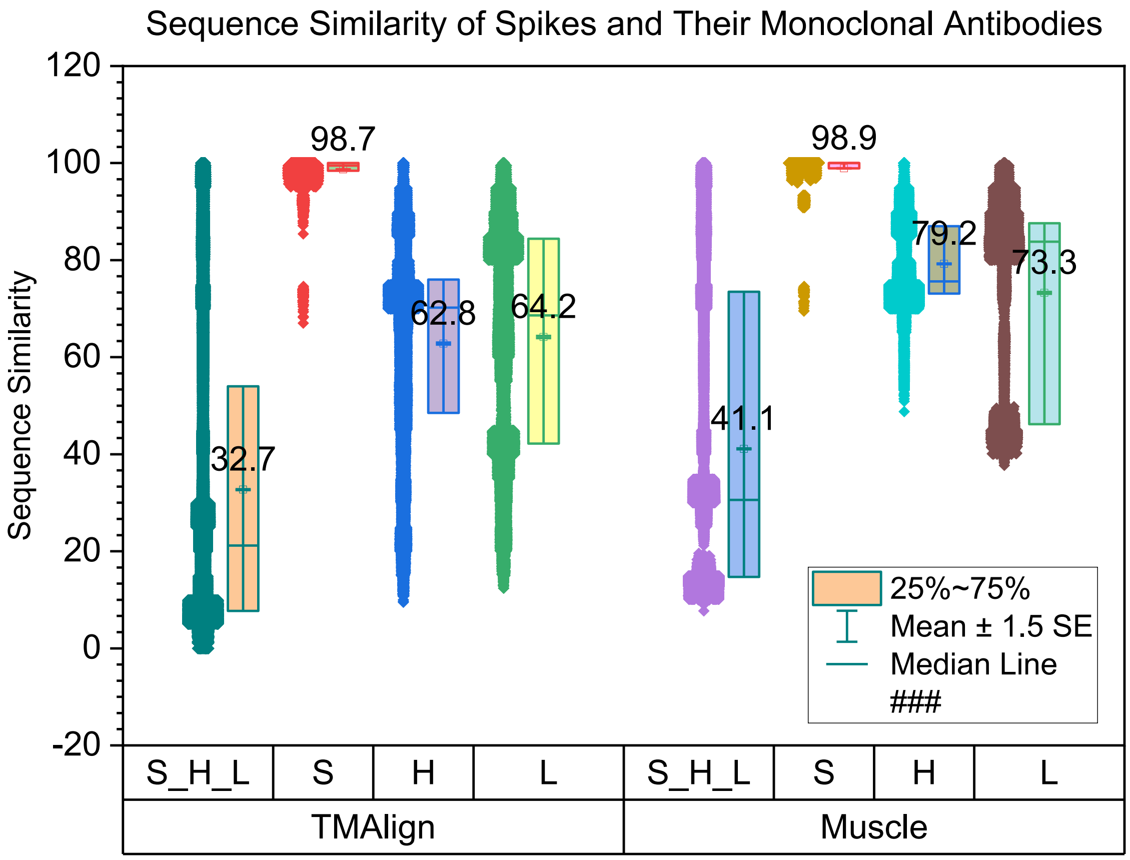

Supplementary Figure 3

a

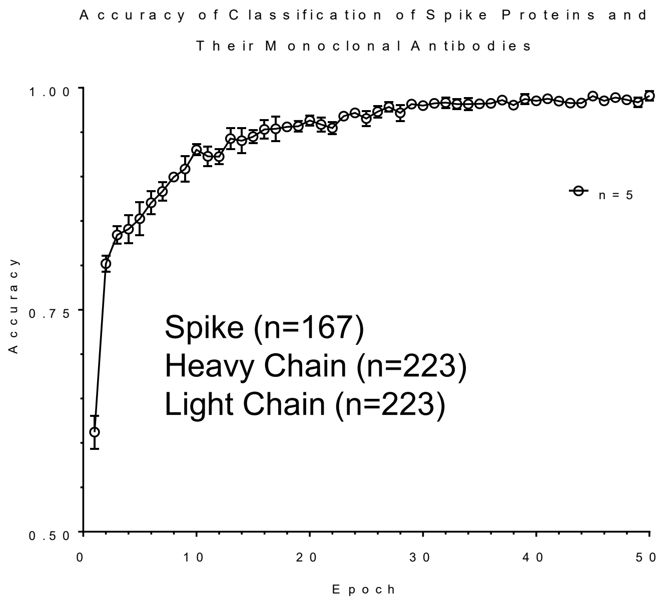

b

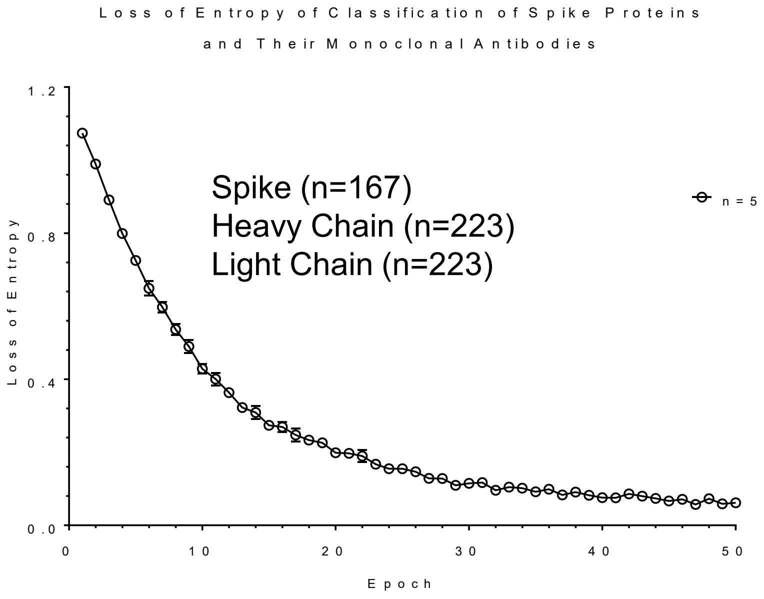

Supplementary Figure 4

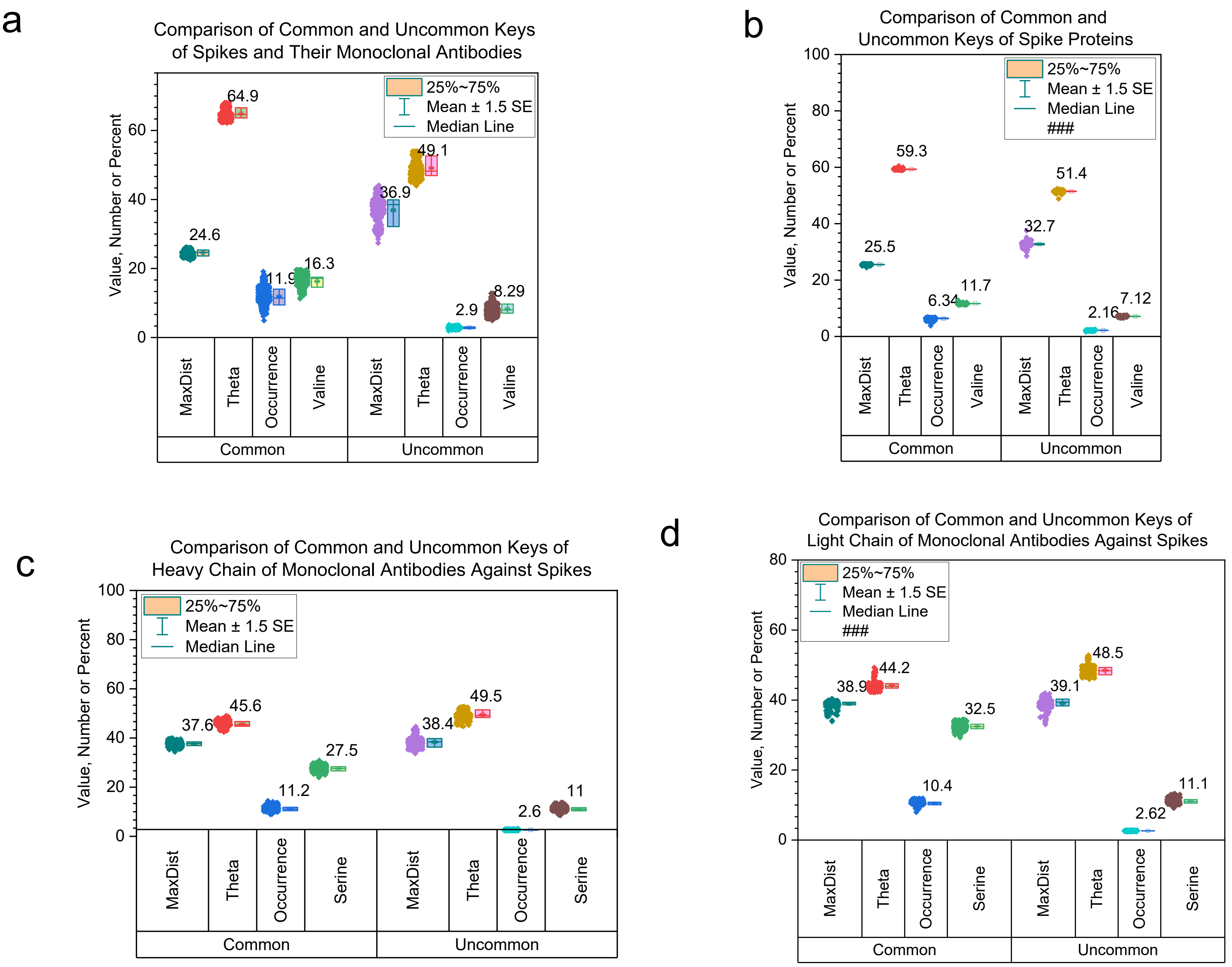

Supplementary Figure 5

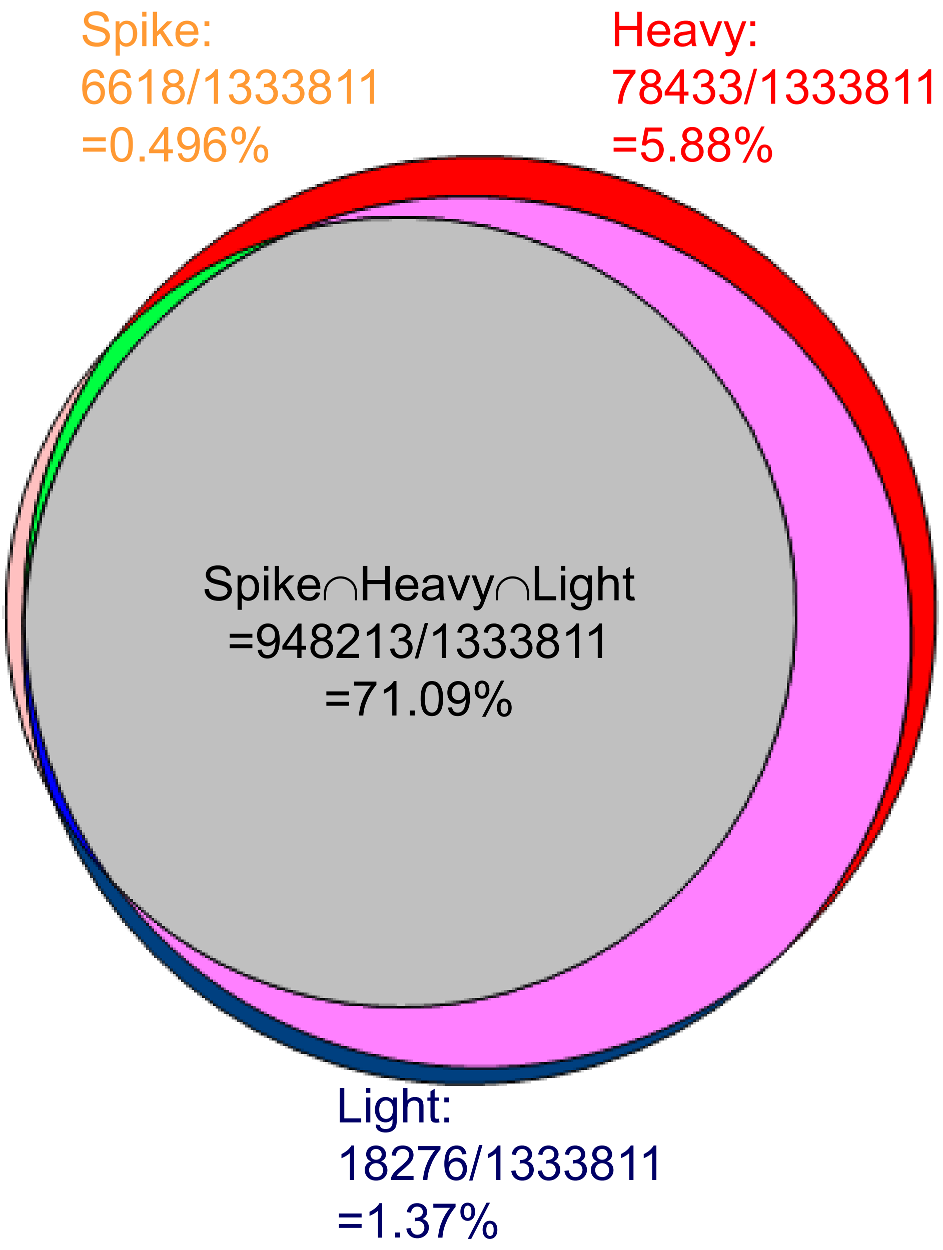

Supplementary Figure 6

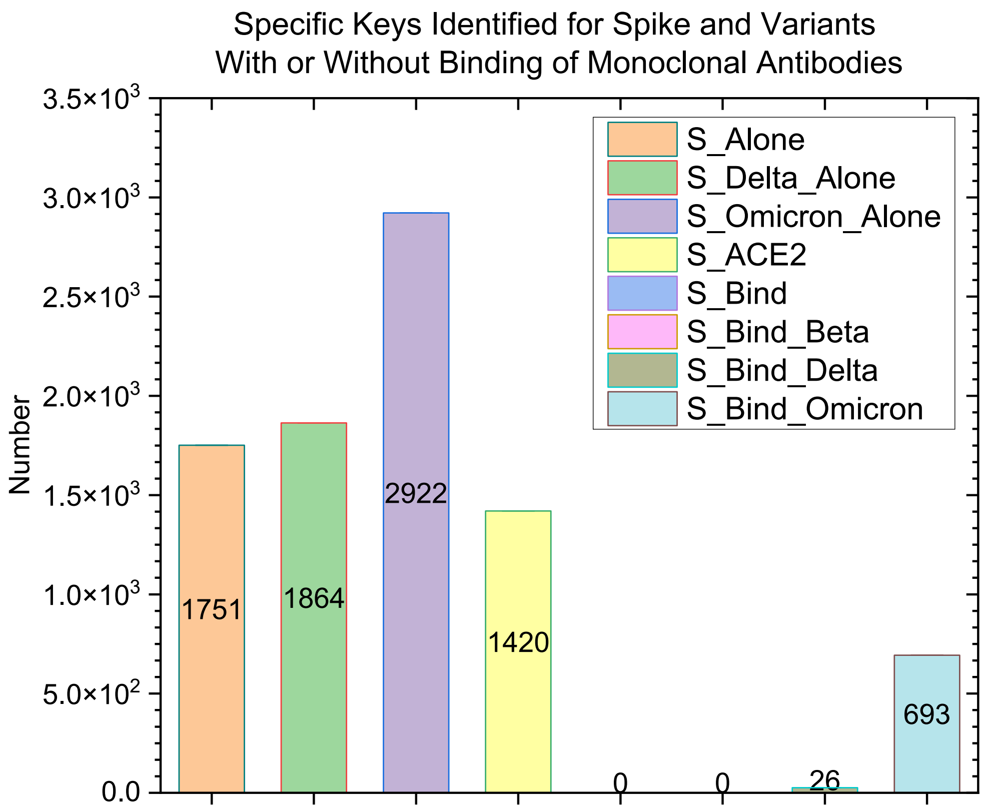

Supplementary Figure 7

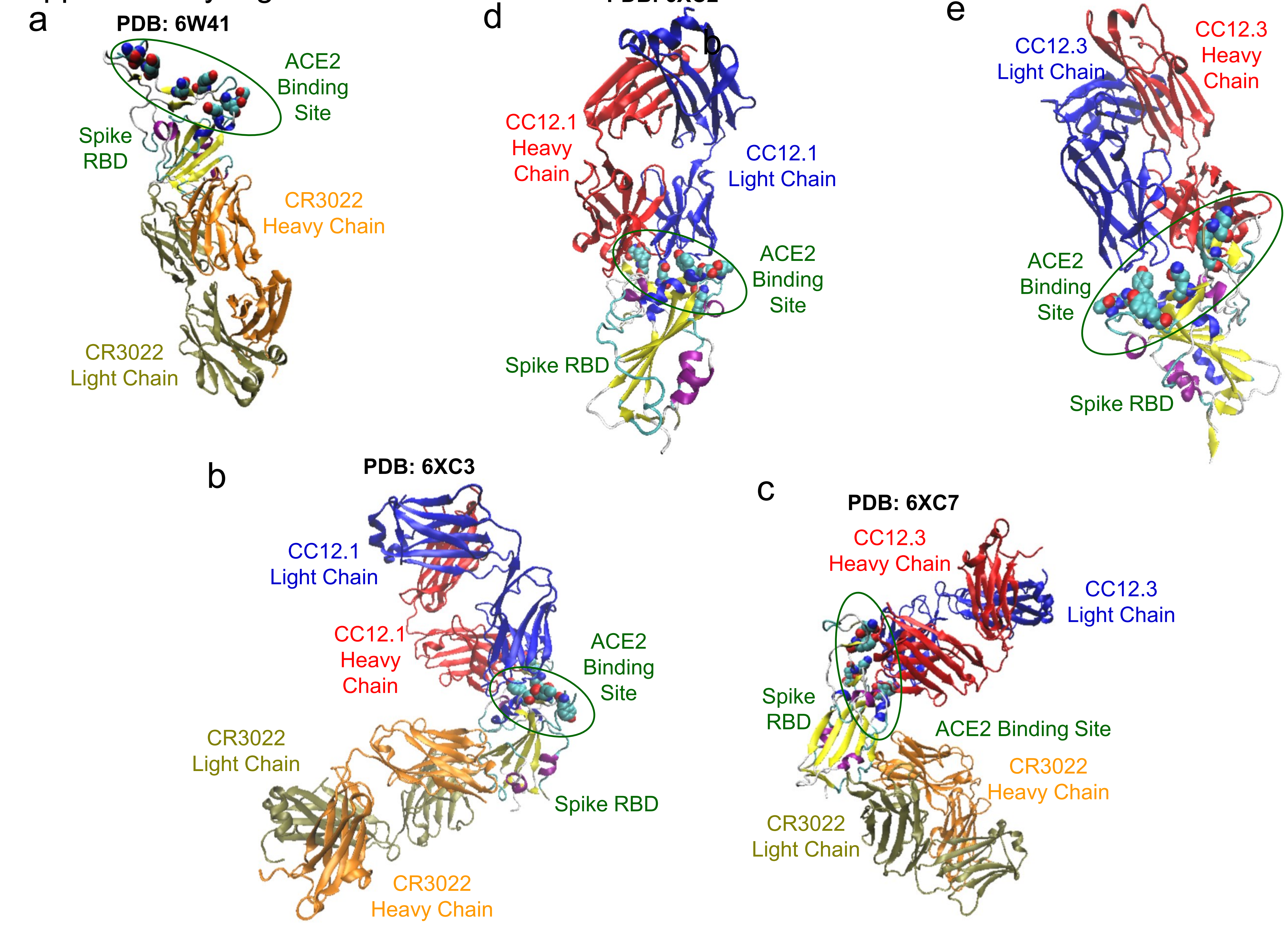

Supplementary Figure 8

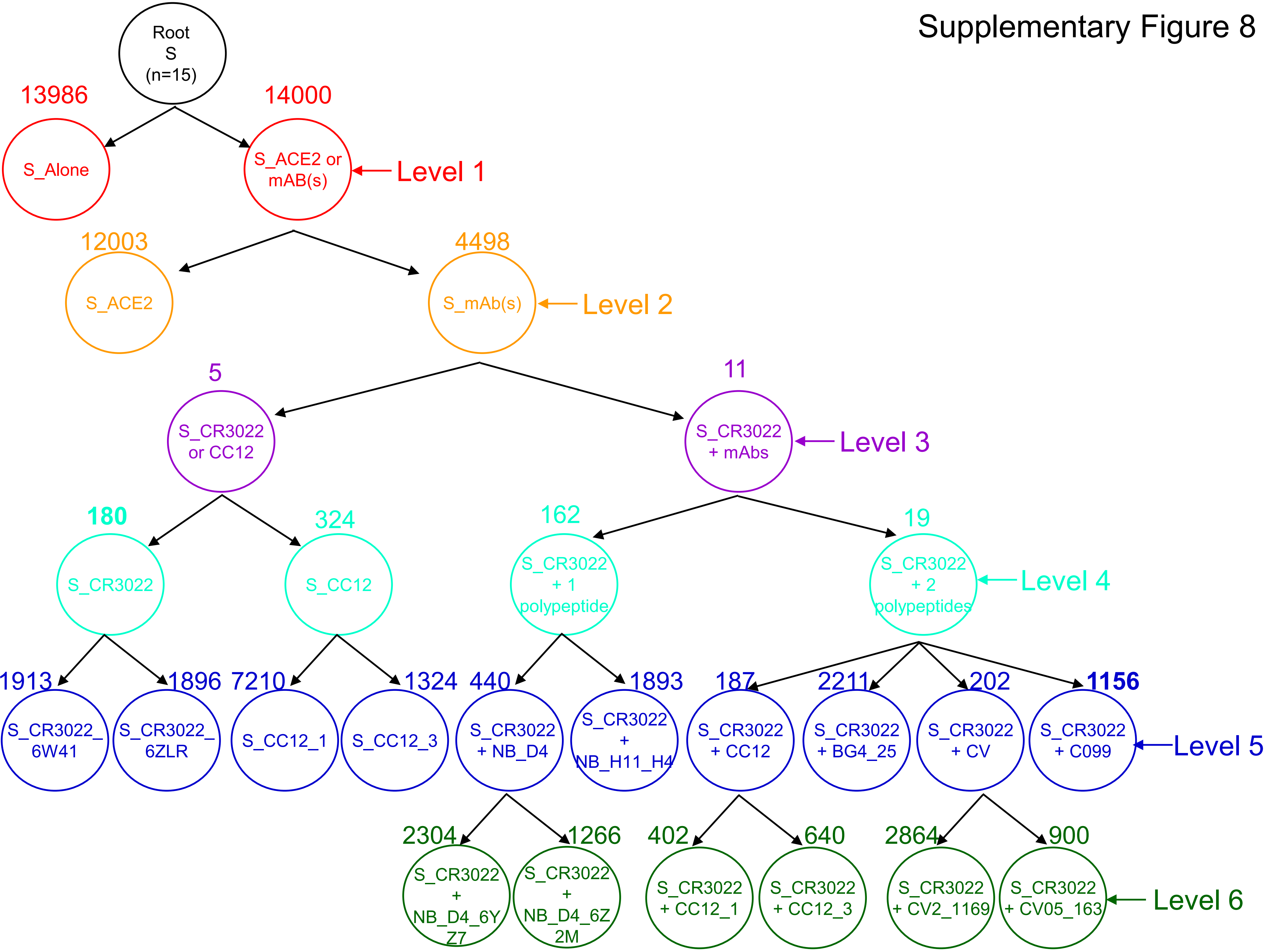

Supplementary Figure 9

a

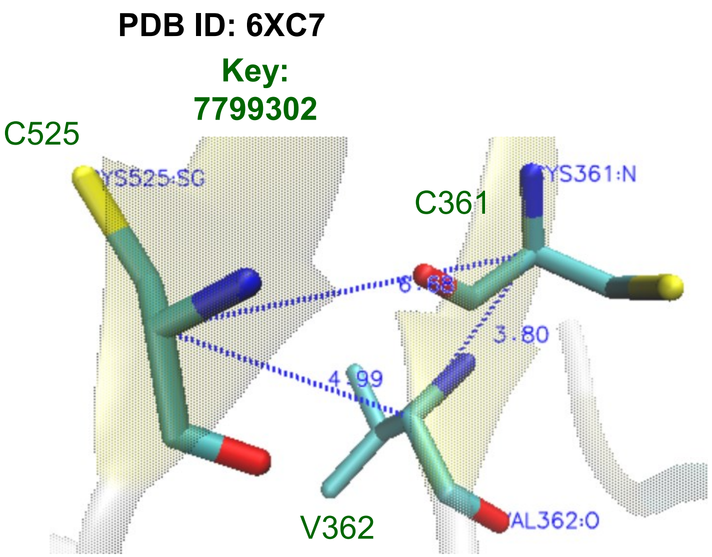

b

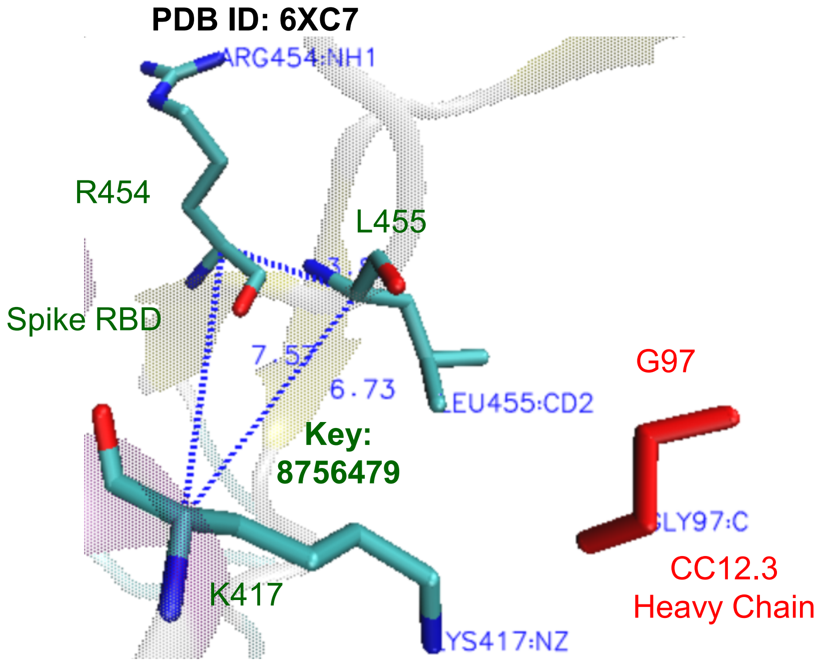

Supplementary Figure 10

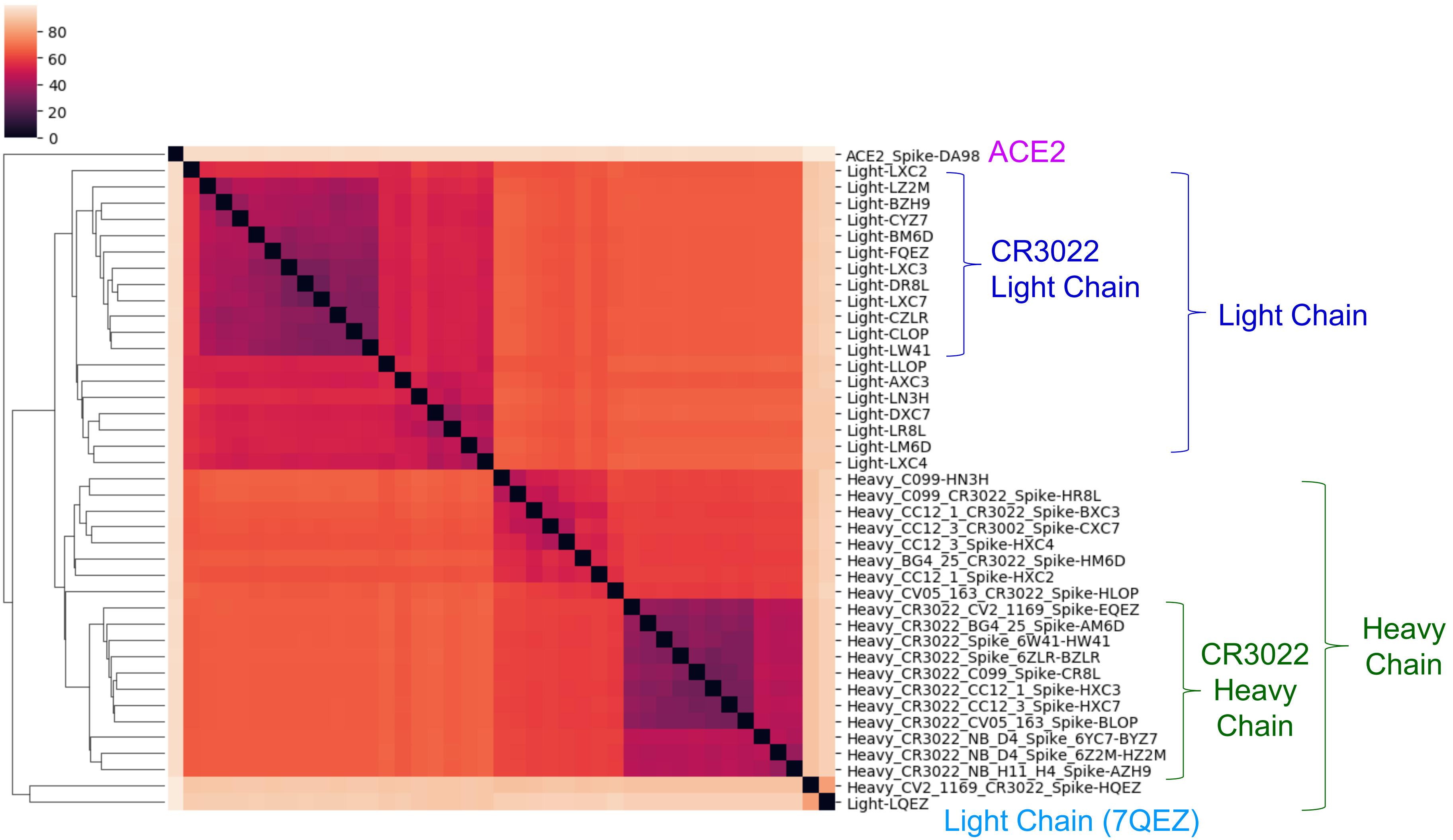

Supplementary Figure 11

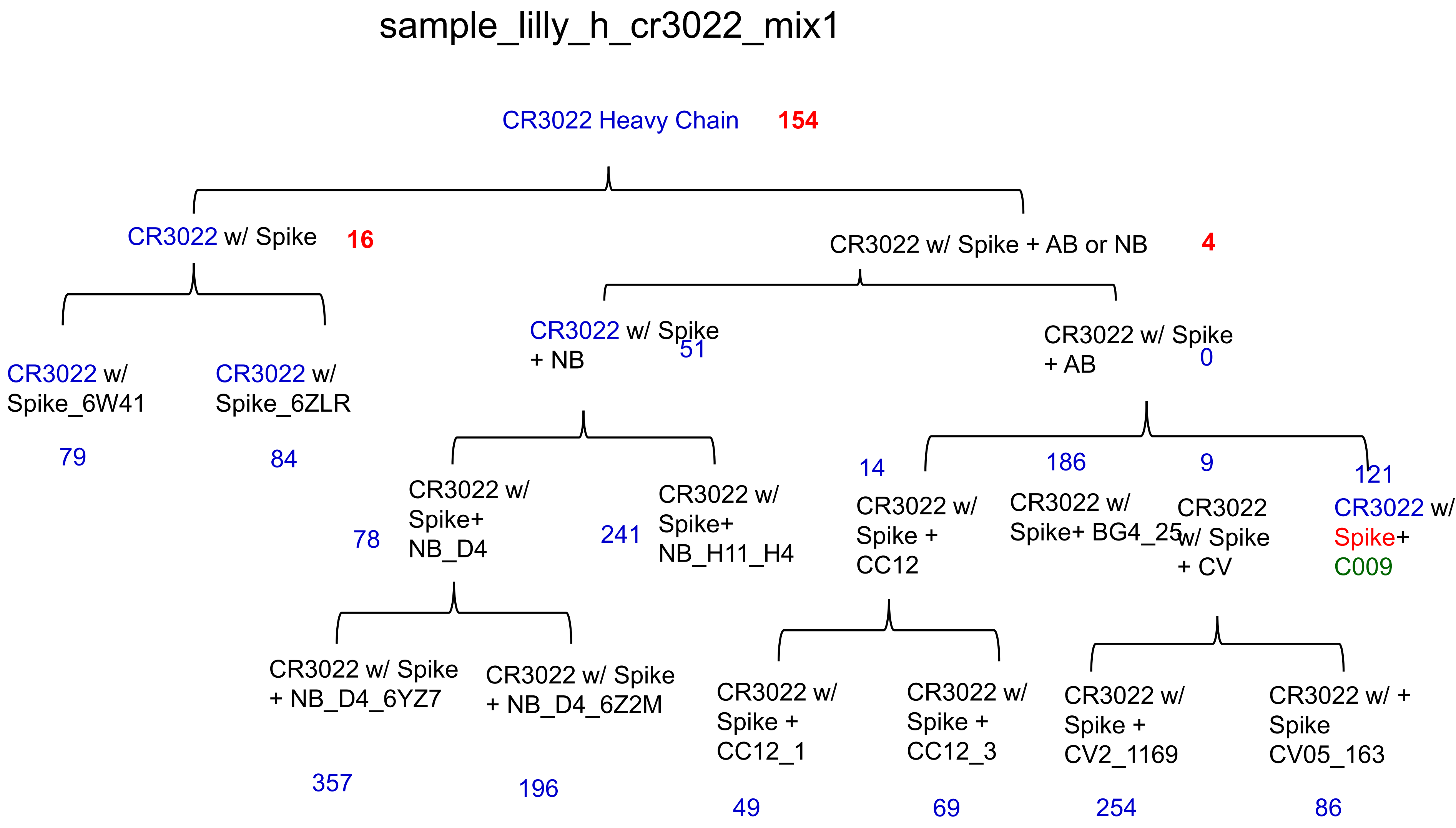

Supplementary Figure 12

6ZLR 3/154 CR3022 Heavy Specific Keys

PDB ID: 6ZLR

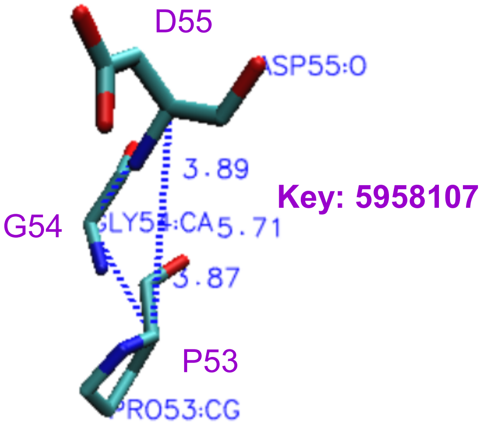

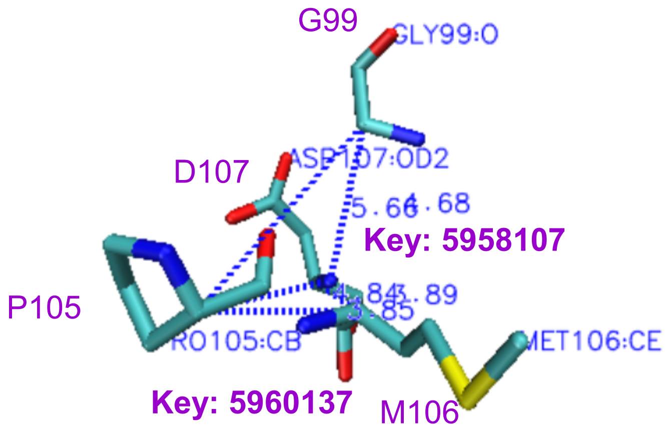

Supplementary Figure 13

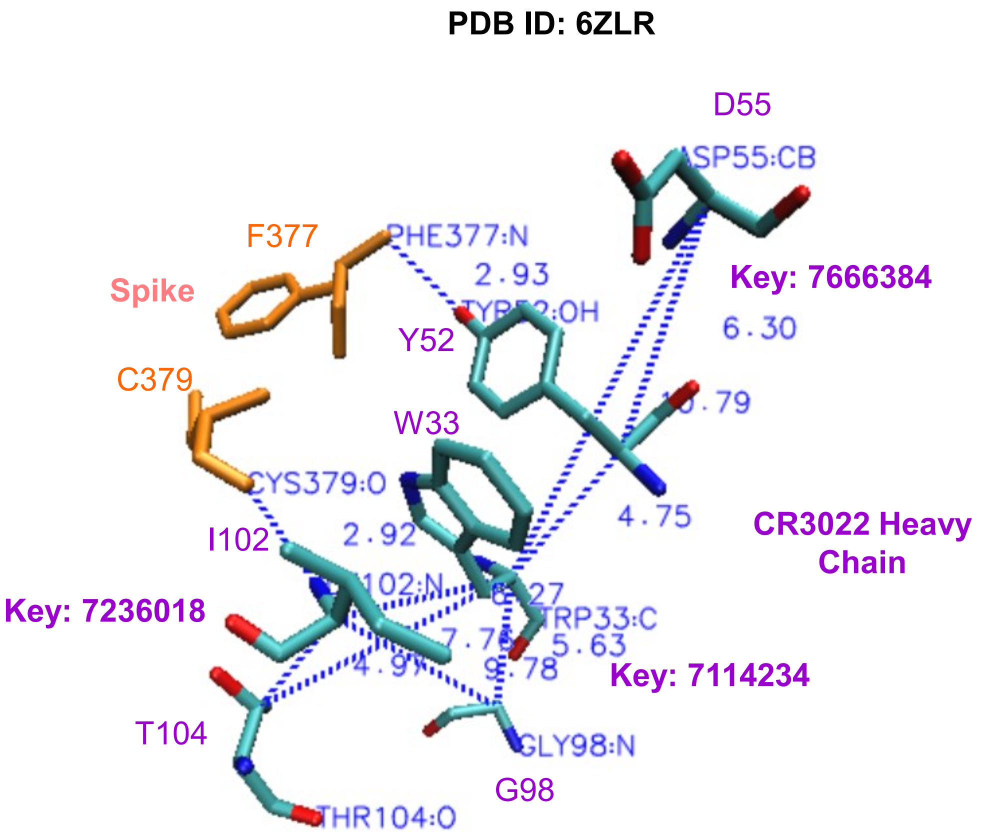

Supplementary Figure 14

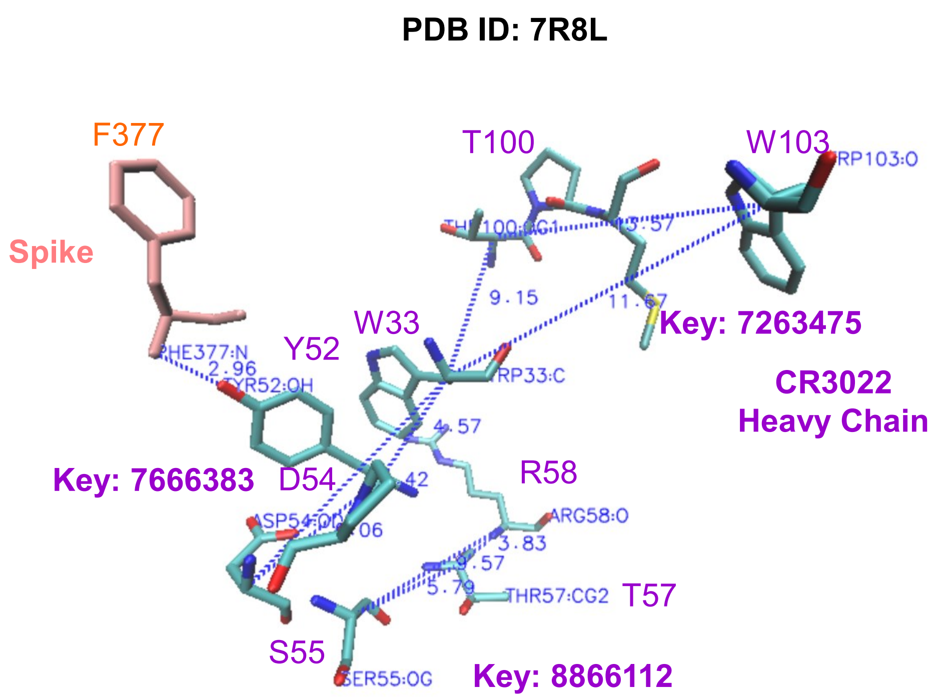

Supplementary Figure 15

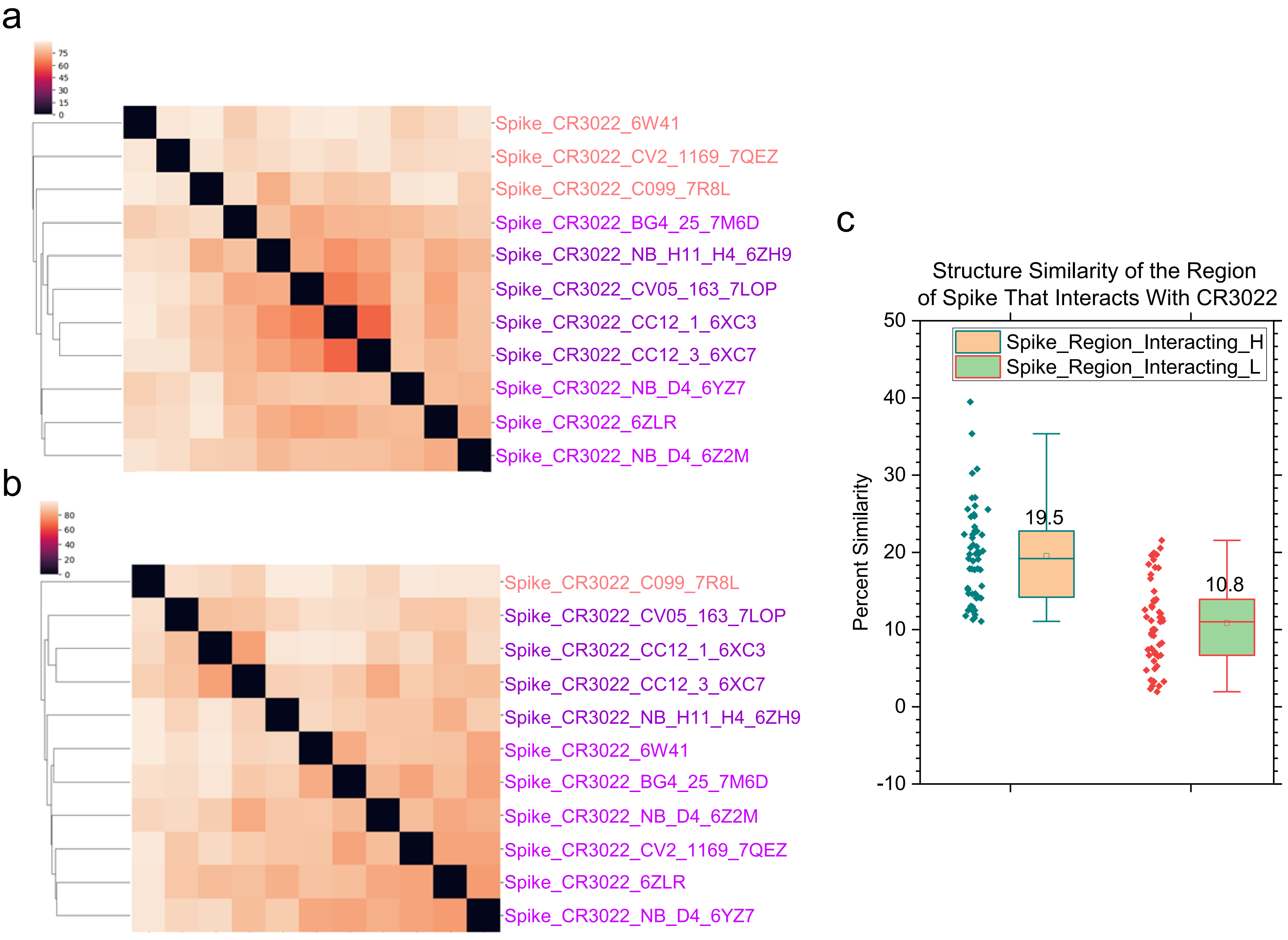

Supplementary Figure 16

```
Consensus      -QMQLVQSGTEVKKPGESLKISCKGSGYGFITYWIGWVRQMPGKGLEWMGIIYPGDSETRYSPSFQGQVTISADKSINTAYLQW-SLKASDTAIYYCAGG
6W41_H_CR3022  -QMQLVQSGTEVKKPGESLKISCKGSGYGFITYWIGWVRQMPGKGLEWMGIIYPGDSETRYSPSFQGQVTISADKSINTAYLQW-SLKASDTAIYYCAGG 98
6XC3_H_CR3022  -QMQLVQSGTEVKKPGESLKISCKGSGYGFITYWIGWVRQMPGKGLEWMGIIYPGDSETRYSPSFQGQVTISADKSINTAYLQW-SLKASDTAIYYCAGG 98
6XC7_H_CR3022  -QMQLVQSGTEVKKPGESLKISCKGSGYGFITYWIGWVRQMPGKGLEWMGIIYPGDSETRYSPSFQGQVTISADKSINTAYLQW-SLKASDTAIYYCAGG 98
6YZ7_H_CR3022  TQMQLVQSGTEVKKPGESLKISCKGSGYGFITYWIGWVRQMPGKGLEWMGIIYPGDSETRYSPSFQGQVTISADKSINTAYLQWSSLKASDTAIYYCAGG 100
6Z2M_H_CR3022  TQMQLVQSGTEVKKPGESLKISCKGSGYGFITYWIGWVRQMPGKGLEWMGIIYPGDSETRYSPSFQGQVTISADKSINTAYLQWSSLKASDTAIYYCAGG 100
6ZH9_H_CR3022  TQMQLVQSGTEVKKPGESLKISCKGSGYGFITYWIGWVRQMPGKGLEWMGIIYPGDSETRYSPSFQGQVTISADKSINTAYLQWSSLKASDTAIYYCAGG 100
6ZLR_H_CR3022  -QMQLVQSGTEVKKPGESLKISCKGSGYGFITYWIGWVRQMPGKGLEWMGIIYPGDSETRYSPSFQGQVTISADKSINTAYLQWSSLKASDTAIYYCAGG 99
7LOP_H_CR3022  -QMQLVQSGTEVKKPGESLKISCKGSGYGFITYWIGWVRQMPGKGLEWMGIIYPGDSETRYSPSFQGQVTISADKSINTAYLQW-SLKASDTAIYYCAGG 98
7M6D_H_CR3022  -QMQLVQSGTEVKKPGESLKISCKGSGYGFITYWIGWVRQMPGKGLEWMGIIYPGDSETRYSPSFQGQVTISADKSINTAYLQW-SLKASDTAIYYCAGG 98
7QEZ_H_CR3022  -QMQLVQSGTEVKKPGESLKISCKGSGYGFITYWIGWVRQMPGKGLEWMGIIYPGDSETRYSPSFQGQVTISADKSINTAYLQWSSLKASDTAIYYCAGG 99
7R8L_H_CR3022  -QMQLVQSGTEVKKPGESLKISCKGSGYGFITYWIGWVRQMPGKGLEWMGIIYPGDSETRYSPSFQGQVTISADKSINTAYLQW-SLKASDTAIYYCAGG 98

Consensus      SGISTPMDVWGQGTTVTVSSASTKGPSVFPLAPS----SGGTAALGCLVKDYFPEPVTVSWNSGALTSGVHTFPAVLQSSGLYSLSSVVTVPSSSLGTQT
6W41_H_CR3022  SGISTPMDVWGQGTTVTVSSASTKGPSVFPLAPSSKSTSGGTAALGCLVKDYFPEPVTVSWNSGALTSGVHTFPAVLQSSGLYSLSSVVTVPSSSLGTQT 198
6XC3_H_CR3022  SGISTPMDVWGQGTTVTVSSASTKGPSVFPLAPSSKSTSGGTAALGCLVKDYFPEPVTVSWNSGALTSGVHTFPAVLQSSGLYSLSSVVTVPSSSLGTQT 198
6XC7_H_CR3022  SGISTPMDVWGQGTTVTVSSASTKGPSVFPLAPS----SGGTAALGCLVKDYFPEPVTVSWNSGALTSGVHTFPAVLQSSGLYSLSSVVTVPSSSLGTQT 194
6YZ7_H_CR3022  SGISTPMDVWGQGTTVTV--ASTKGPSVFPLAPS----SGGTAALGCLVKDYFPEPVTVSWNSGALTSGVHTFPAVLQSSGLYSLSSVVTVPSSSLGTQT 194
6Z2M_H_CR3022  SGISTPMDVWGQGTTVTV--ASTKGPSVFPLAPS----SGGTAALGCLVKDYFPEPVTVSWNSGALTSGVHTFPAVLQSSGLYSLSSVVTVPSSSLGTQT 194
6ZH9_H_CR3022  SGISTPMDVWGQGTTVTV--ASTKGPSVFPLAPS----SGGTAALGCLVKDYFPEPVTVSWNSGALTSGVHTFPAVLQSSGLYSLSSVVTVPSSSLGTQT 194
6ZLR_H_CR3022  SGISTPMDVWGQGTTVTVSSASTKGPSVFPLAPSSKSTSGGTAALGCLVKDYFPEPVTVSWNSGALTSGVHTFPAVLQSSGLYSLSSVVTVPSSSLGTQT 199
7LOP_H_CR3022  SGISTPMDVWGQGTTVTVSSASTKGPSVFPLAPS----SGGTAALGCLVKDYFPEPVTVSWNSGALTSGVHTFPAVLQSSGLYSLSSVVTVPSSSLGTQT 194
7M6D_H_CR3022  SGISTPMDVWGQGTTVTVSSASTKGPSVFPLAPSSKSTSGGTAALGCLVKDYFPEPVTVSWNSGALTSGVHTFPAVLQSSGLYSLSSVVTVPSSSLGTQT 198
7QEZ_H_CR3022  SGISTPMDVWGQGTTVTVSSASTKGPSVFPLAP-------GTAALGCLVKDYFPEPVTVSWNSGALTSGVHTFPAVLQSSGLYSLSSVVTVPSSSLGTQT 192
7R8L_H_CR3022  SGISTPMDVWGQGTTVTVSSASTKGPSVFPLAPSSKSTSGGTAALGCLVKDYFPEPVTVSWNSGALTSGVHTFPAVLQSSGLYSLSSVVTVPSSSLGTQT 198

Consensus      YICNVNHKPSNTKVDKKVEPKSC
6W41_H_CR3022  YICNVNHKPSNTKVDKKVEPKSC 221
6XC3_H_CR3022  YICNVNHKPSNTKVDKKVEPKSC 221
6XC7_H_CR3022  YICNVNHKPSNTKVDKKVEPKSC 217
6YZ7_H_CR3022  YICNVNHKPSNTKVDKKVEPKS- 216
6Z2M_H_CR3022  YICNVNHKPSNTKVDKKVEPKS- 216
6ZH9_H_CR3022  YICNVNHKPSNTKVDKKVEPKS- 216
6ZLR_H_CR3022  YICNVNHKPSNTKVDKKVEPKSC 222
7LOP_H_CR3022  YICNVNHKPSNTKVDKKVEPKSC 217
7M6D_H_CR3022  YICNVNHKPSNTKVDKKVEPKSC 221
7QEZ_H_CR3022  YICNVNHKPSNTKVDKKVEPKS- 214
7R8L_H_CR3022  YICNVNHKPSNTKVDKKVEPKSC 221
```

Supplementary Figure 17

```
Consensus      DIQLTQSPDSLAVSLGERATINCKSSQSVLY--INKNYLAWYQQKPGQPPKLLIYWASTRESGVPDRFSGSGSGTDFTLTISSLQAEDVAVYYCQQYYSTPYTFGQGTKV
6W41_...R3022  DIQLTQSPDSLAVSLGERATINCKSSQSVLY--INKNYLAWYQQKPGQPPKLLIYWASTRESGVPDRFSGSGSGTDFTLTISSLQAEDVAVYYCQQYYSTPYTFGQGTKV 108
6XC3_L_CR3022  DIQLTQSPDSLAVSLGERATINCKSSQSVLY--INKNYLAWYQQKPGQPPKLLIYWASTRESGVPDRFSGSGSGTDFTLTISSLQAEDVAVYYCQQYYSTPYTFGQGTKV 108
6XC7_L_CR3022  DIQLTQSPDSLAVSLGERATINCKSSQSVLY--INKNYLAWYQQKPGQPPKLLIYWASTRESGVPDRFSGSGSGTDFTLTISSLQAEDVAVYYCQQYYSTPYTFGQGTKV 108
6YZ7_L_CR3022  DIQLTQSPDSLAVSLGERATINCKSSQSVLYSSINKNYLAWYQQKPGQPPKLLIYWASTRESGVPDRFSGSGSGTDFTLTISSLQAEDVAVYYCQQYYSTPYTFGQGTKV 110
6Z2M_...R3022  DIQLTQSPDSLAVSLGERATINCKSSQSVLYSSINKNYLAWYQQKPGQPPKLLIYWASTRESGVPDRFSGSGSGTDFTLTISSLQAEDVAVYYCQQYYSTPYTFGQGTKV 110
6ZH9_L_CR3022  DIQLTQSPDSLAVSLGERATINCKSSQSVLYSSINKNYLAWYQQKPGQPPKLLIYWASTRESGVPDRFSGSGSGTDFTLTISSLQAEDVAVYYCQQYYSTPYTFGQGTKV 110
6ZLR_L_CR3022  DIQLTQSPDSLAVSLGERATINCKSSQSVLYSSINKNYLAWYQQKPGQPPKLLIYWASTRESGVPDRFSGSGSGTDFTLTISSLQAEDVAVYYCQQYYSTPYTFGQGTKV 110
7LOP_L_CR3022  DIQLTQSPDSLAVSLGERATINCKSSQSVLY--INKNYLAWYQQKPGQPPKLLIYWASTRESGVPDRFSGSGSGTDFTLTISSLQAEDVAVYYCQQYYSTPYTFGQGTKV 108
7M6D_...R3022  DIQLTQSPDSLAVSLGERATINCKSSQSVLY--INKNYLAWYQQKPGQPPKLLIYWASTRESGVPDRFSGSGSGTDFTLTISSLQAEDVAVYYCQQYYSTPYTFGQGTKV 108
7QEZ_...R3022  DIQLTQSPDSLAVSLGERATINCKSSQSVLYSSINKNYLAWYQQKPGQPPKLLIYWASTRESGVPDRFSGSGSGTDFTLTISSLQAEDVAVYYCQQYYSTPYTFGQGTKV 110
7R8L_L_CR3022  DIQLTQSPDSLAVSLGERATINCKSSQSVLY--INKNYLAWYQQKPGQPPKLLIYWASTRESGVPDRFSGSGSGTDFTLTISSLQAEDVAVYYCQQYYSTPYTFGQGTKV 108

Consensus      EIKRTVAAPSVFIFPPSDEQLKSGTASVVCLLNNFYPREAKVQWKVDNALQSGNSQESVTEQDSKDSTYSLSSTLTLSKADYEKHKVYACEVTHQGLSSPVTKSFNRGEC
6W41_...R3022  EIKRTVAAPSVFIFPPSDEQLKSGTASVVCLLNNFYPREAKVQWKVDNALQSGNSQESVTEQDSKDSTYSLSSTLTLSKADYEKHKVYACEVTHQGLSSPVTKSFNRGEC 218
6XC3_L_CR3022  EIKRTVAAPSVFIFPPSDEQLKSGTASVVCLLNNFYPREAKVQWKVDNALQSGNSQESVTEQDSKDSTYSLSSTLTLSKADYEKHKVYACEVTHQGLSSPVTKSFNRGEC 218
6XC7_L_CR3022  EIKRTVAAPSVFIFPPSDEQLKSGTASVVCLLNNFYPREAKVQWKVDNALQSGNSQESVTEQDSKDSTYSLSSTLTLSKADYEKHKVYACEVTHQGLSSPVTKSFNRGEC 218
6YZ7_L_CR3022  EIKRTVAAPSVFIFPPSDEQLKSGTASVVCLLNNFYPREAKVQWKVDNALQSGNSQESVTEQDSKDSTYSLSSTLTLSKADYEKHKVYACEVTHQGLSSPVTKSFNRGE- 219
6Z2M_...R3022  EIKRTVAAPSVFIFPPSDEQLKSGTASVVCLLNNFYPREAKVQWKVDNALQSGNSQESVTEQDSKDSTYSLSSTLTLSKADYEKHKVYACEVTHQGLSSPVTKSFNRGE- 219
6ZH9_L_CR3022  EIKRTVAAPSVFIFPPSDEQLKSGTASVVCLLNNFYPREAKVQWKVDNALQSGNSQESVTEQDSKDSTYSLSSTLTLSKADYEKHKVYACEVTHQGLSSPVTKSFNRGE- 219
6ZLR_L_CR3022  EIKRTVAAPSVFIFPPSDEQLKSGTASVVCLLNNFYPREAKVQWKVDNALQSGNSQESVTEQDSKDSTYSLSSTLTLSKADYEKHKVYACEVTHQGLSSPVTKSFNRGE- 219
7LOP_L_CR3022  EIKRTVAAPSVFIFPPSDEQLKSGTASVVCLLNNFYPREAKVQWKVDNALQSGNSQESVTEQDSKDSTYSLSSTLTLSKADYEKHKVYACEVTHQGLSSPVTKSFNRGEC 218
7M6D_...R3022  EIKRTVAAPSVFIFPPSDEQLKSGTASVVCLLNNFYPREAKVQWKVDNALQSGNSQESVTEQDSKDSTYSLSSTLTLSKADYEKHKVYACEVTHQGLSSPVTKSFNRGEC 218
7QEZ_...R3022  EIKRTVAAPSVFIFPPSDEQLKSGTASVVCLLNNFYPREAKVQWKVDNALQSGNSQESVTEQDSKDSTYSLSSTLTLSKADYEKHKVYACEVTHQGLSSPVTKSFNRG-- 218
7R8L_L_CR3022  EIKRTVAAPSVFIFPPSDEQLKSGTASVVCLLNNFYPREAKVQWKVDNALQSGNSQESVTEQDSKDSTYSLSSTLTLSKADYEKHKVYACEVTHQGLSSPVTKSFNRGEC 218

Consensus      S
6W41_...R3022  S 219
6XC3_L_CR3022  S 219
6XC7_L_CR3022  S 219
6YZ7_L_CR3022  - 219
6Z2M_...R3022  - 219
6ZH9_L_CR3022  - 219
6ZLR_L_CR3022  - 219
7LOP_L_CR3022  S 219
7M6D_...R3022  S 219
7QEZ_...R3022  - 218
7R8L_L_CR3022  S 219
```

Supplementary Figure 18

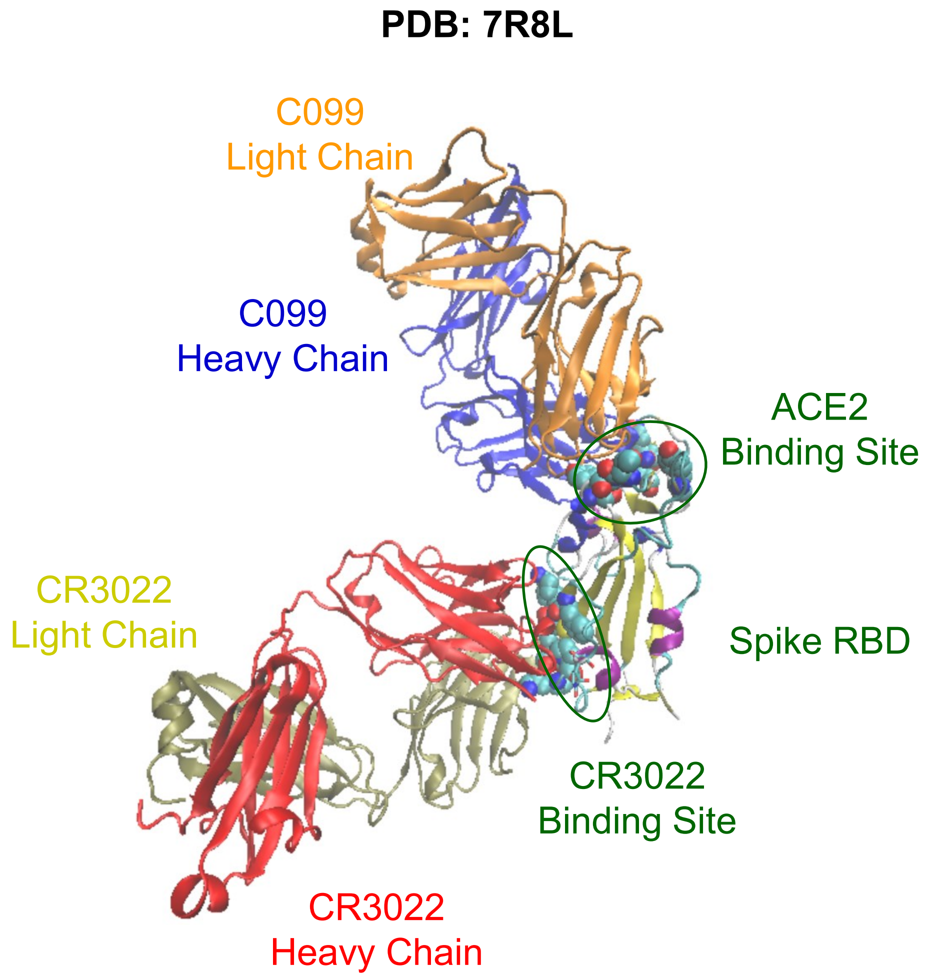

Supplementary Figure 19

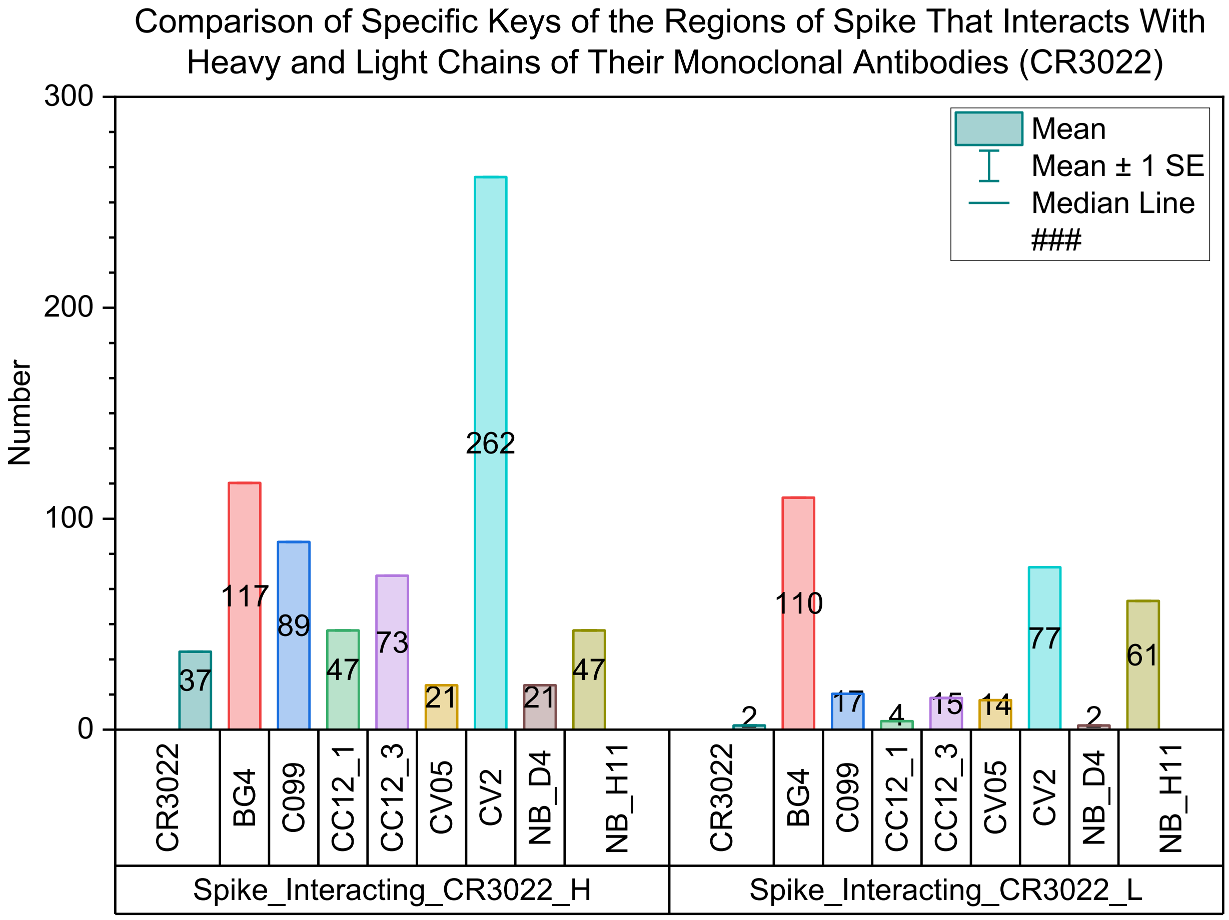

Supplementary Figure 20

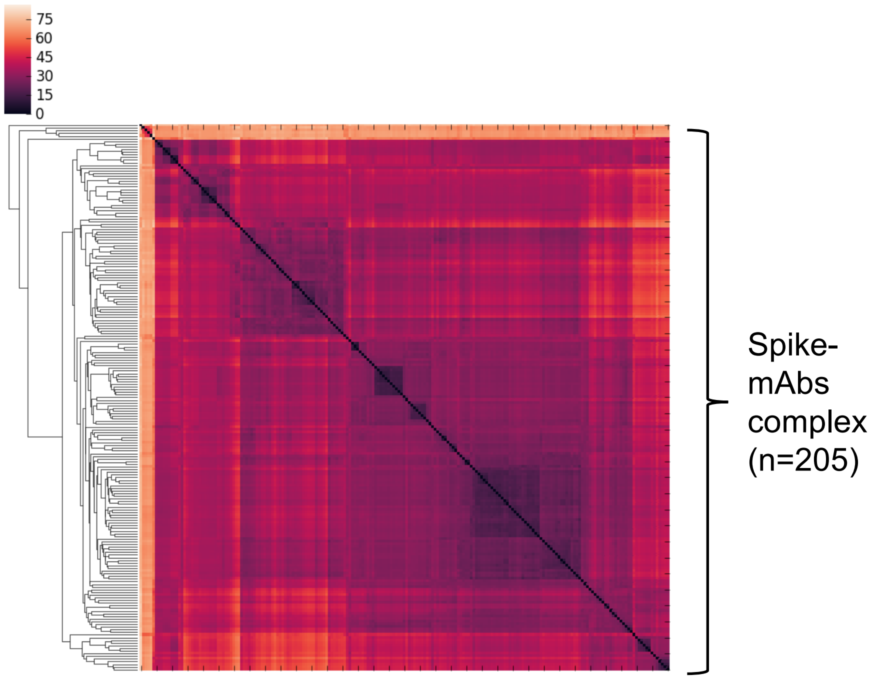

Supplementary Figure 21

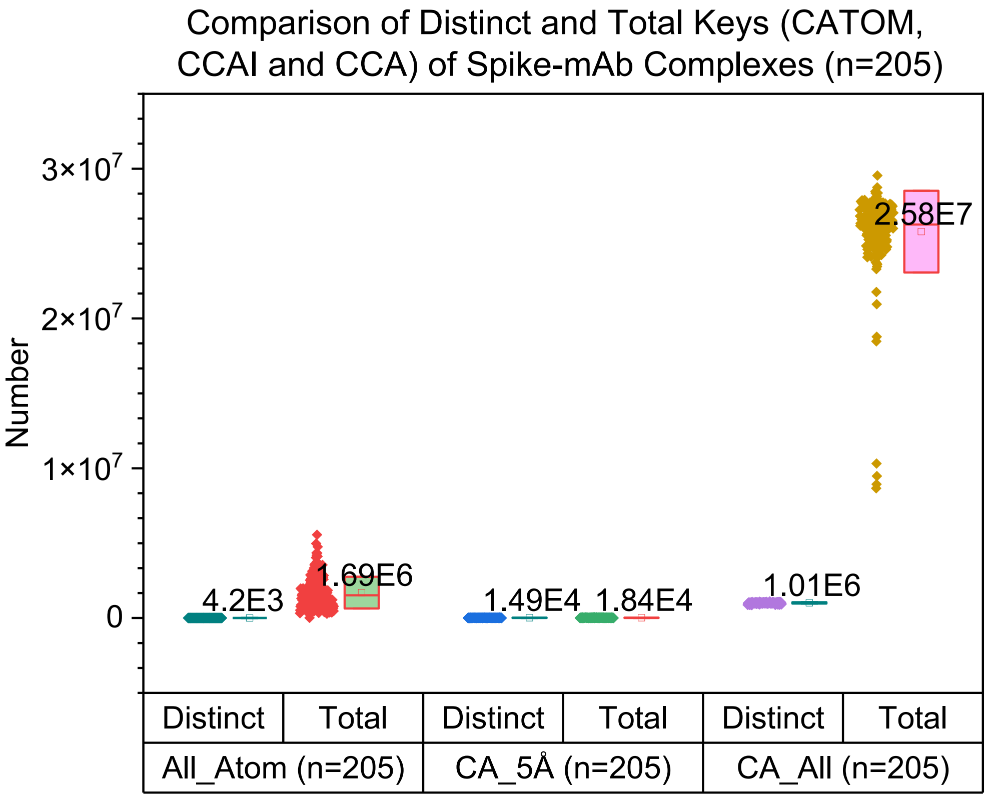

Supplementary Figure 22

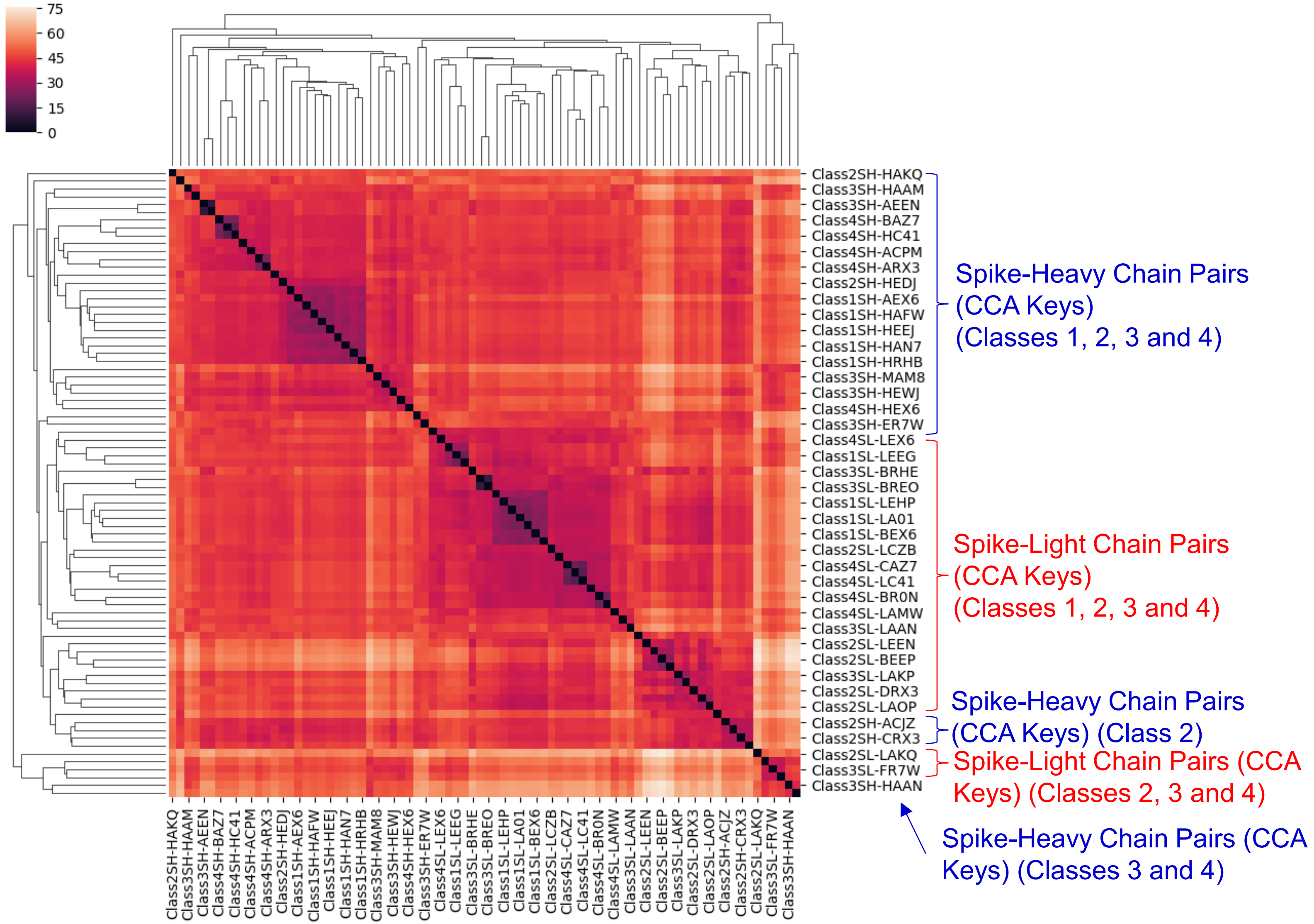

Supplementary Figure 30

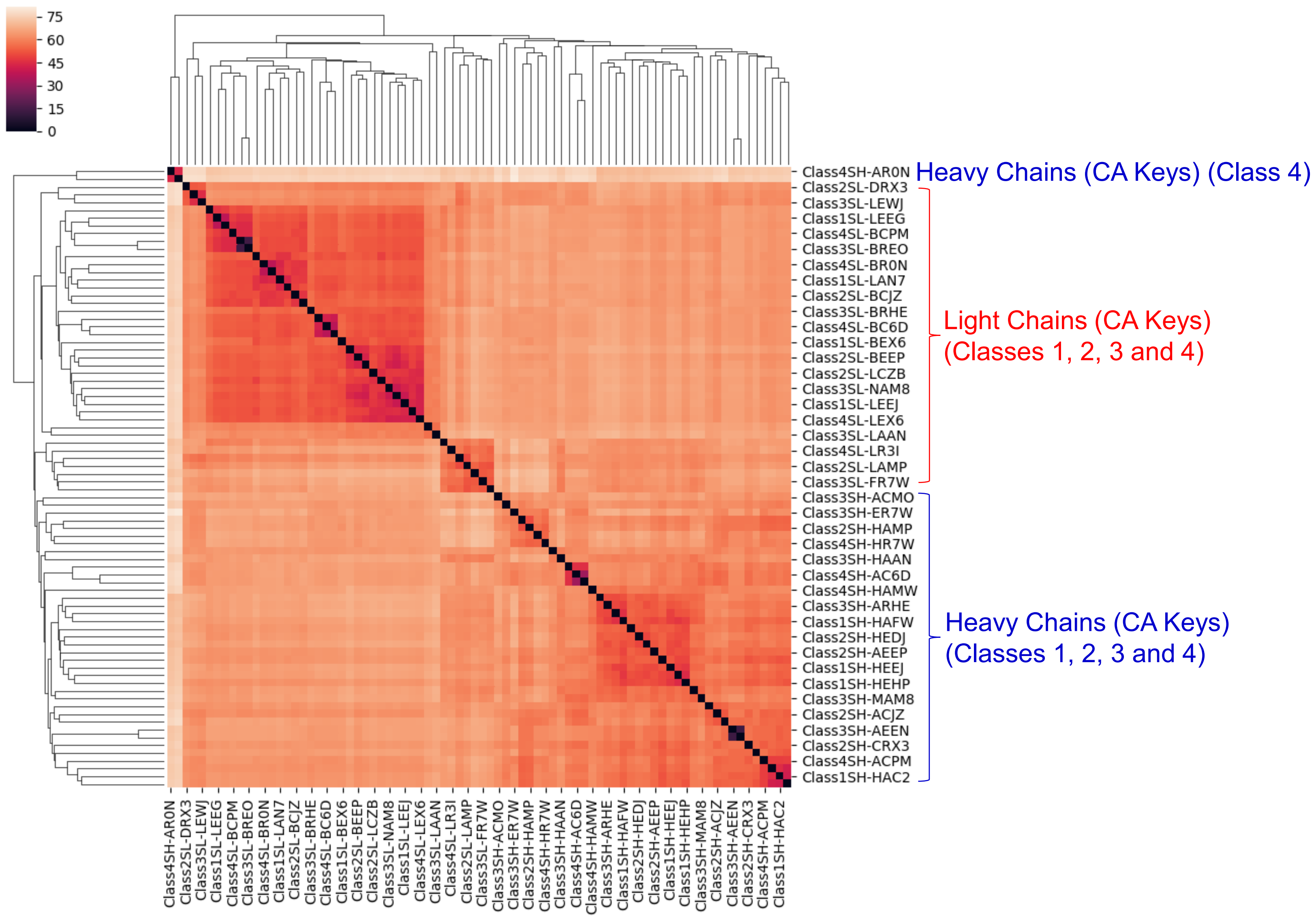

Supplementary Figure 24

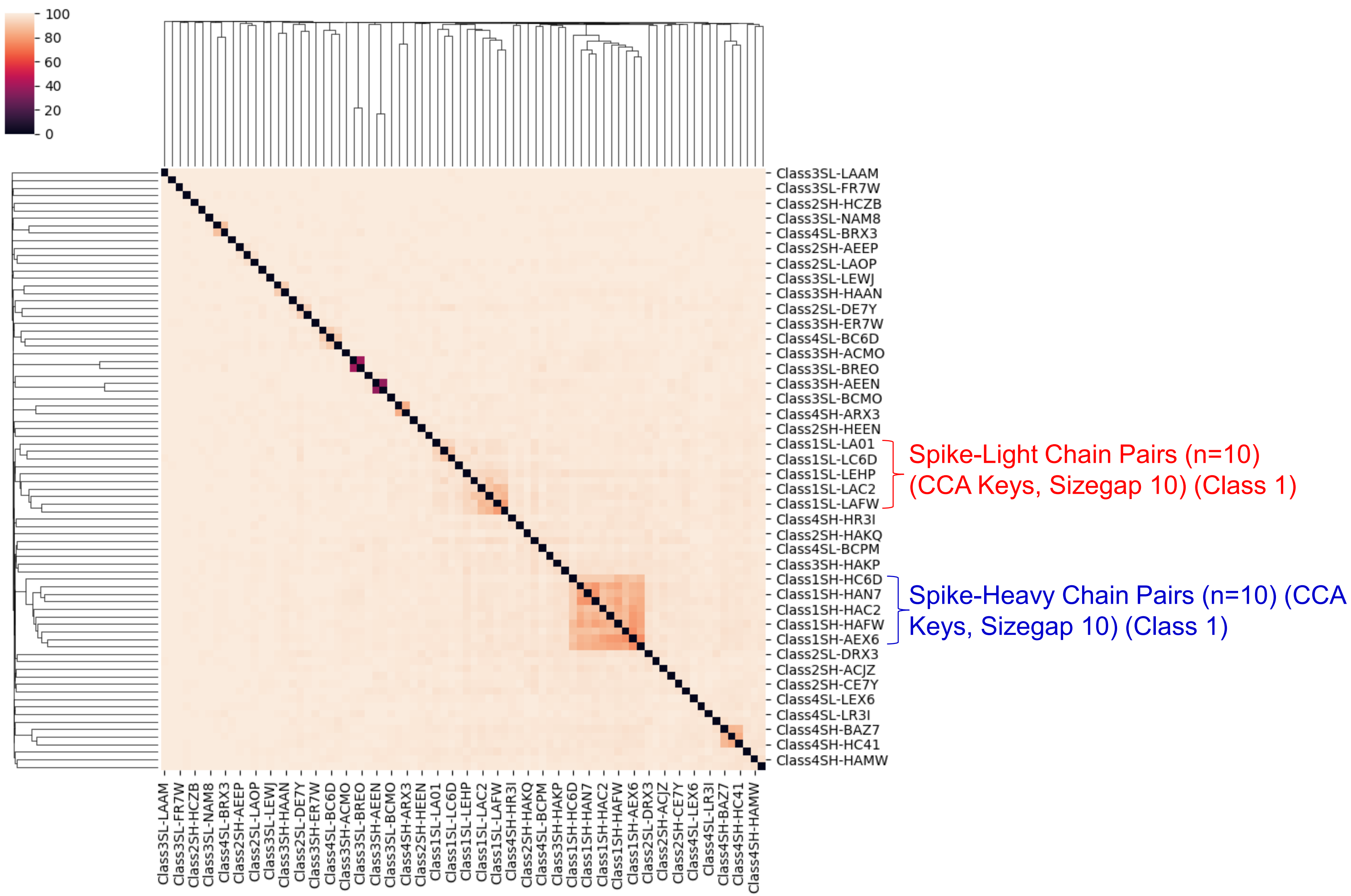

Supplementary Figure 25

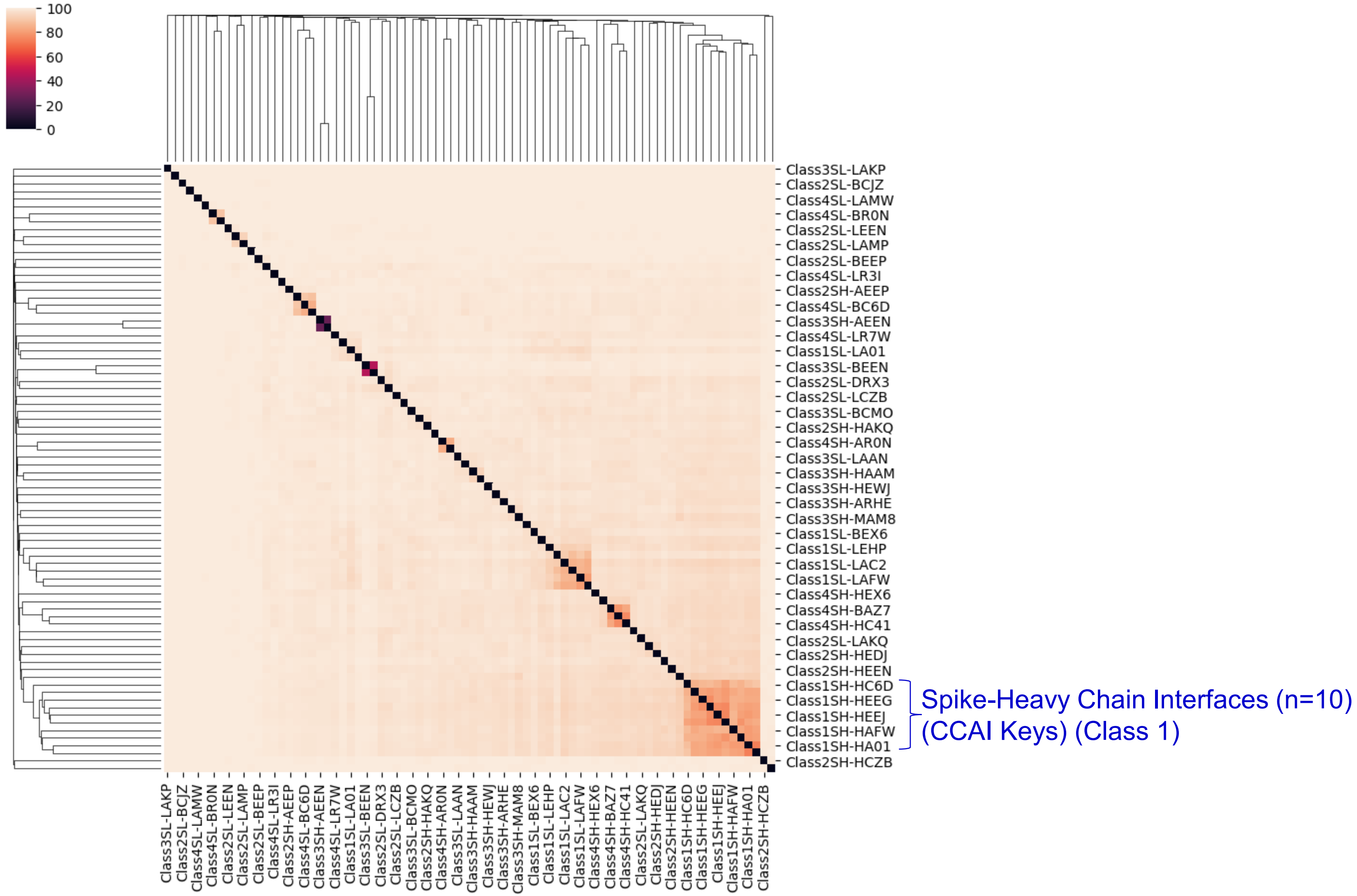

Supplementary Figure 26

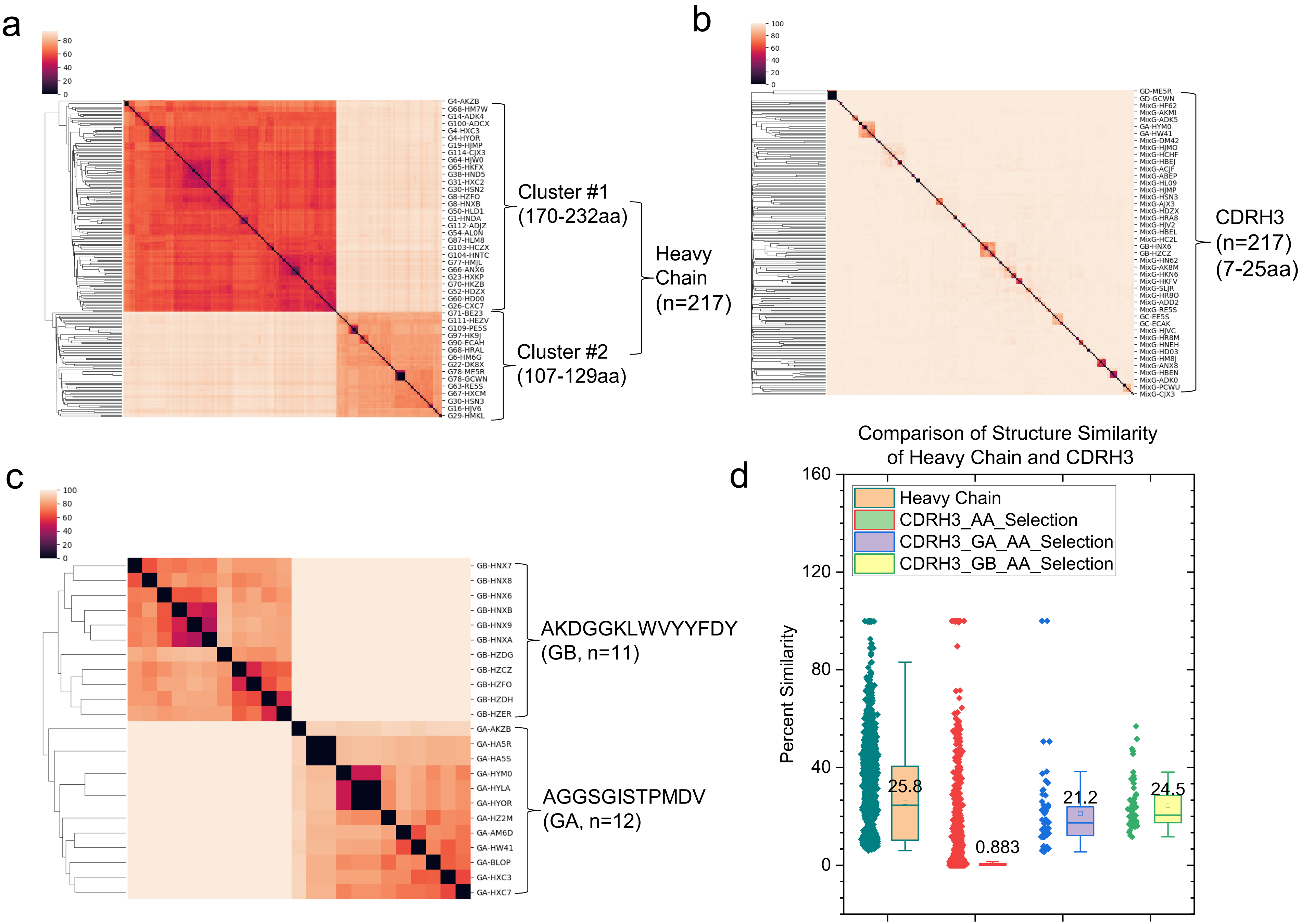

Supplementary Figure 27

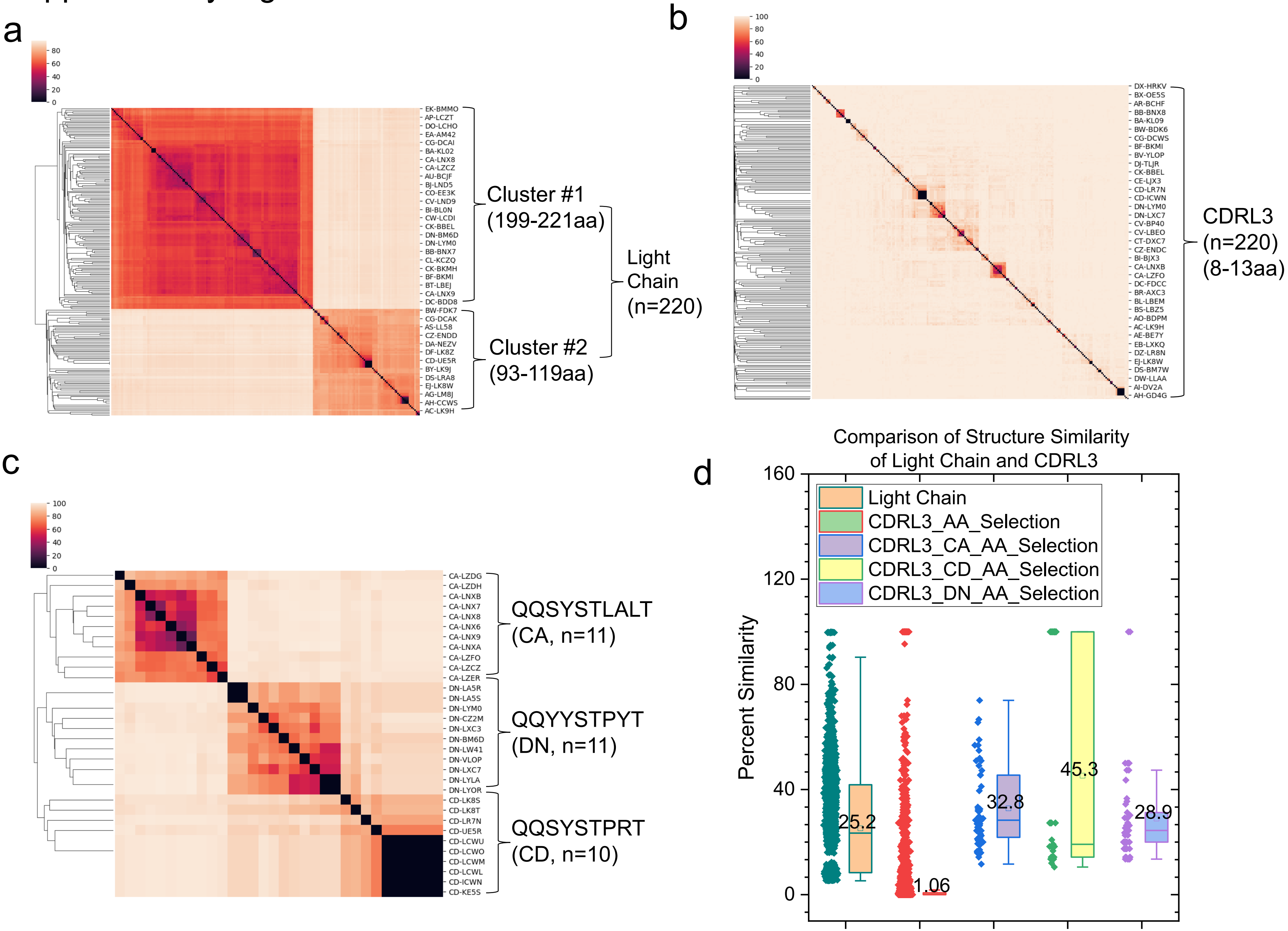

Supplementary Figure 28

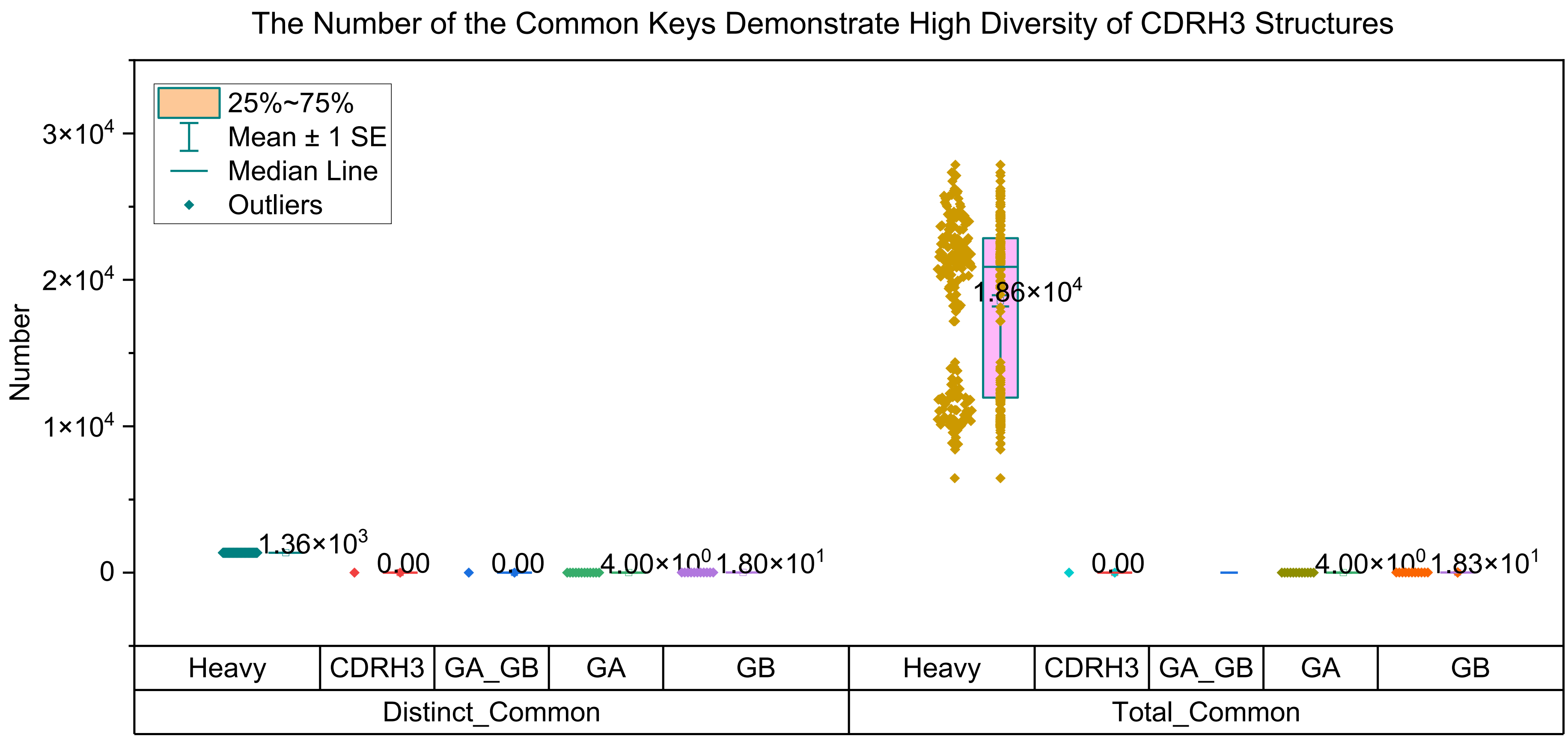

Supplementary Figure 29

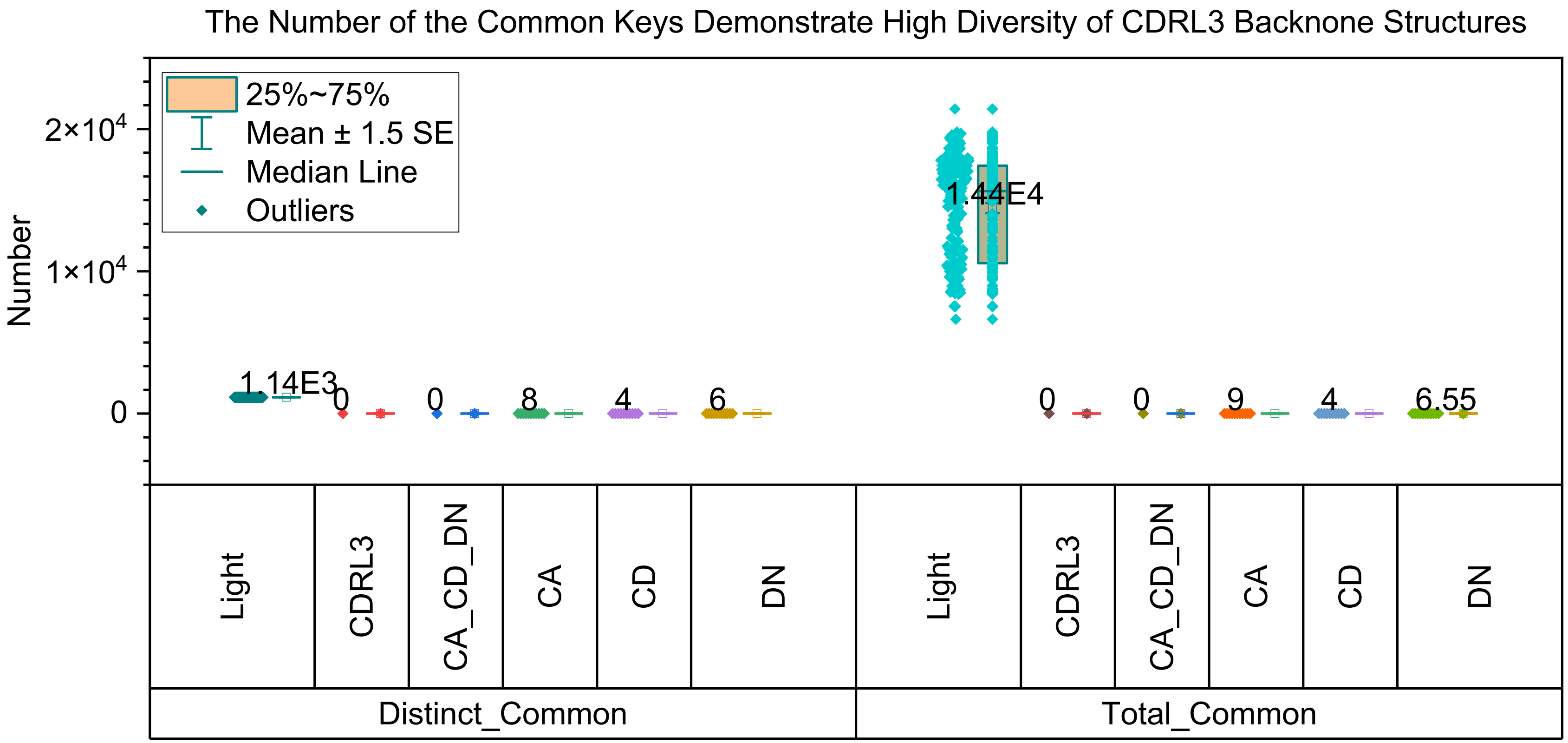

Supplementary Figure 30

a

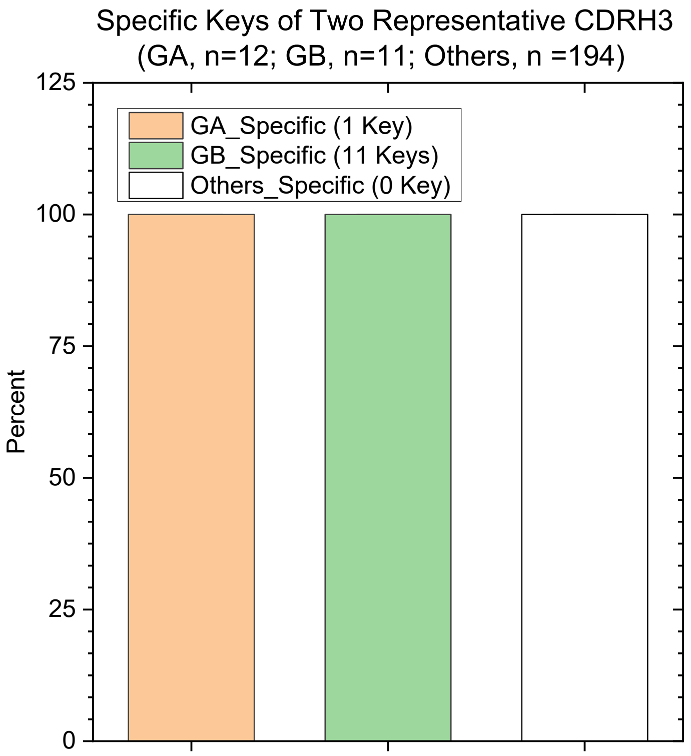

b

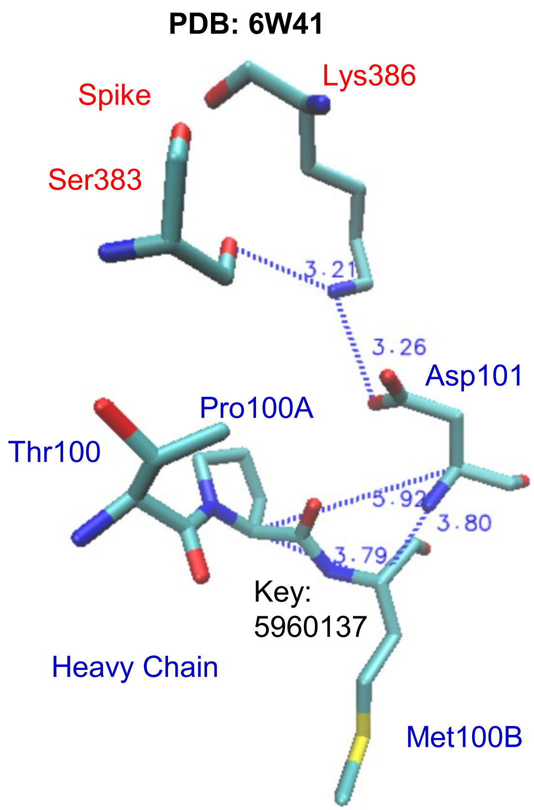

Supplementary Figure 31

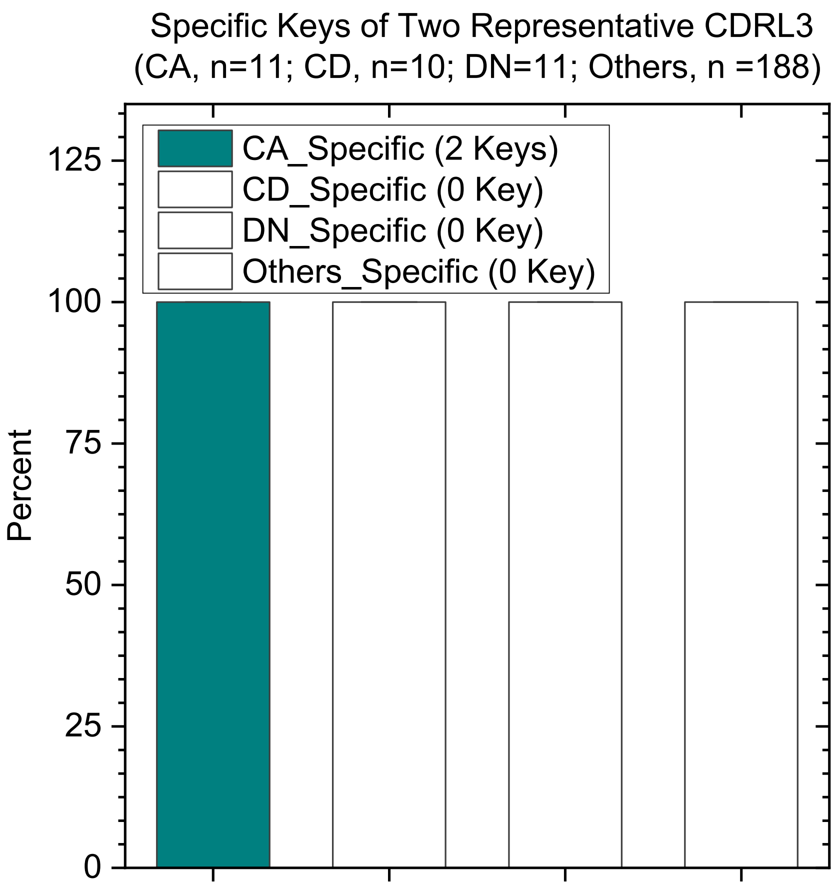

Supplementary Figure 32

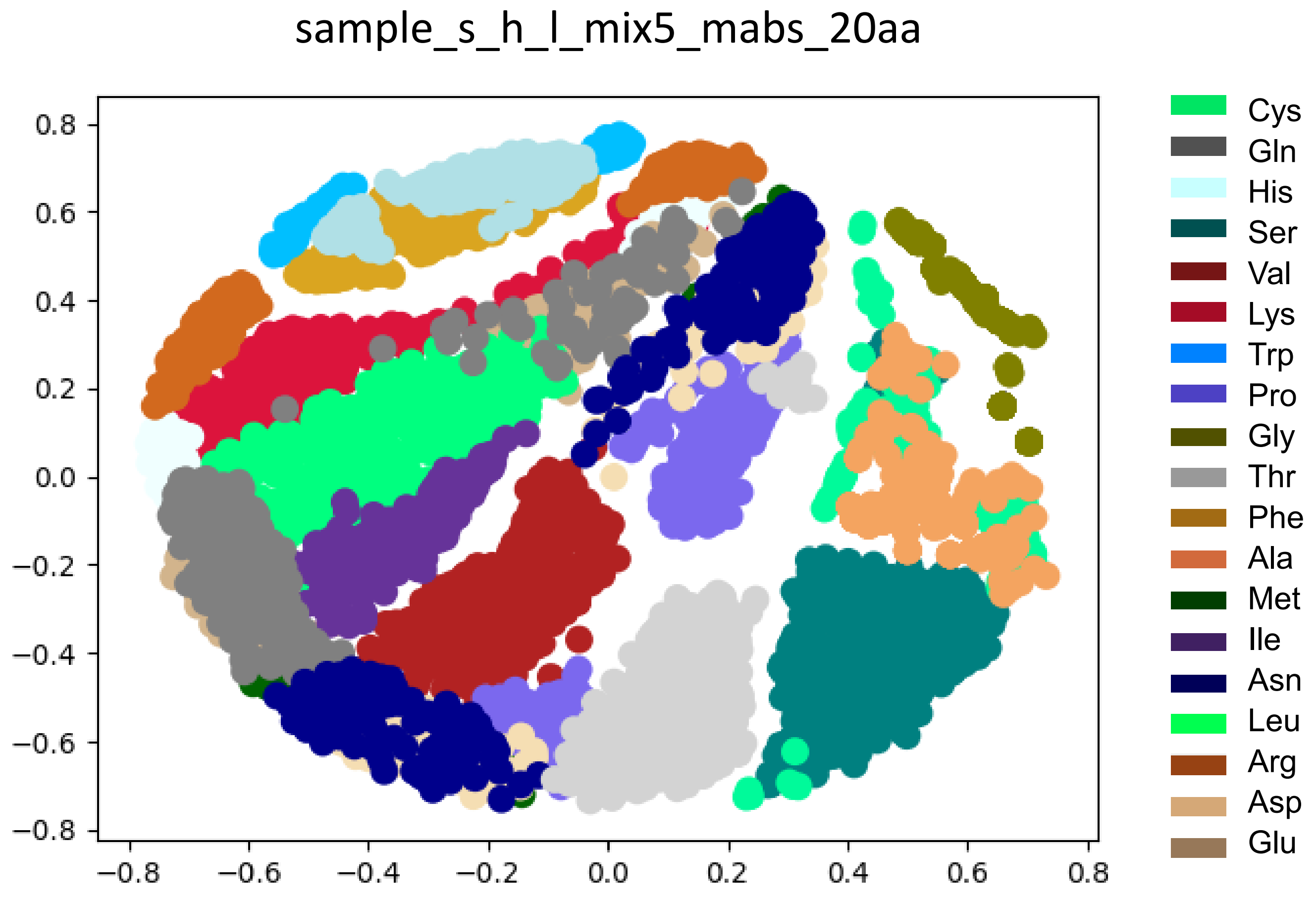

Supplementary Figure 33

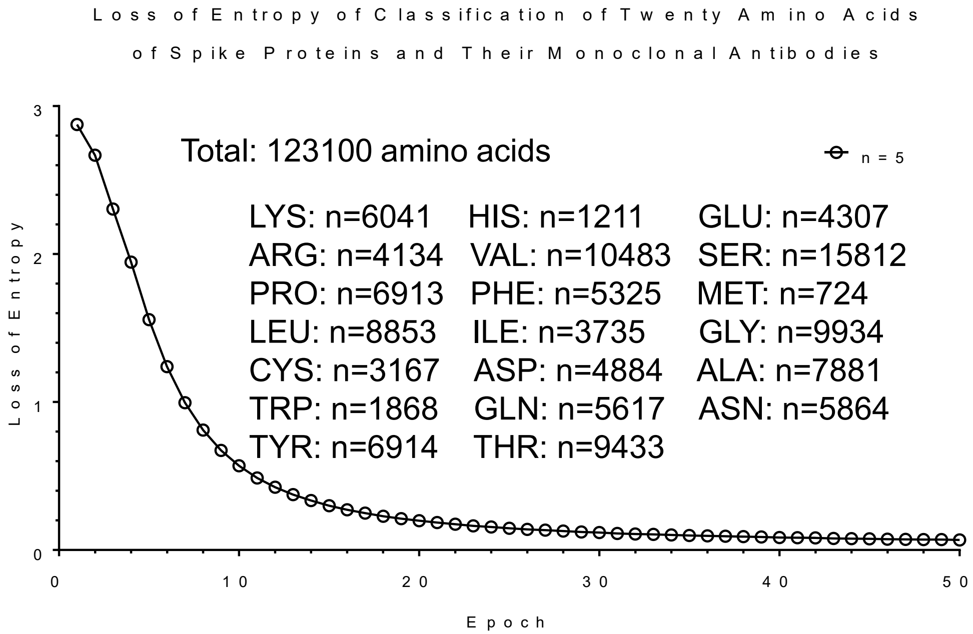

Supplementary Figure 34

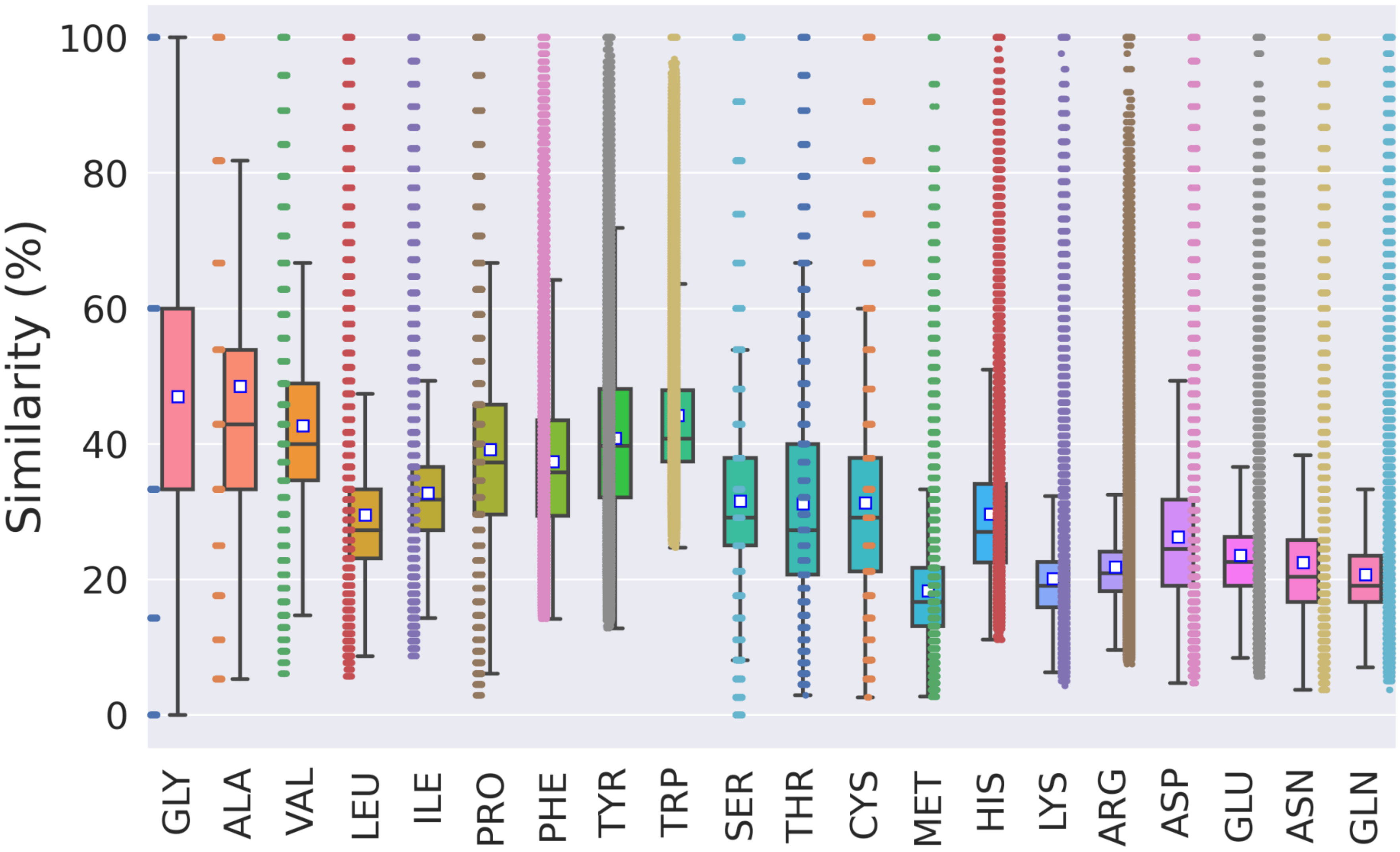

Supplementary Figure 35

a

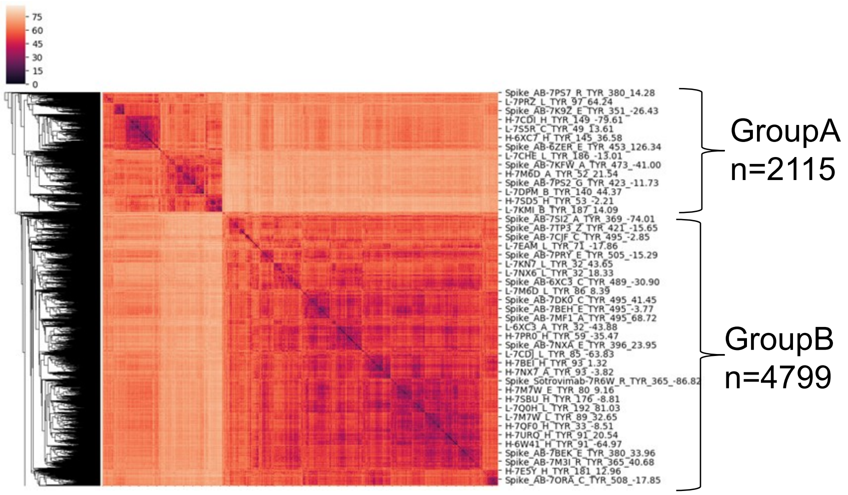

c

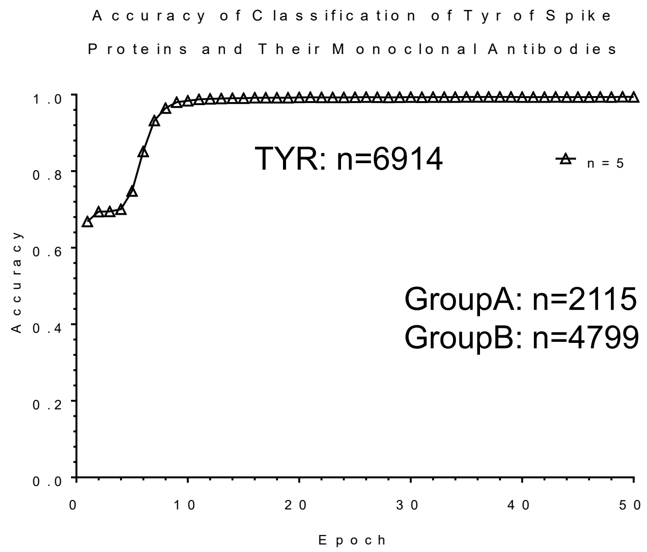

b

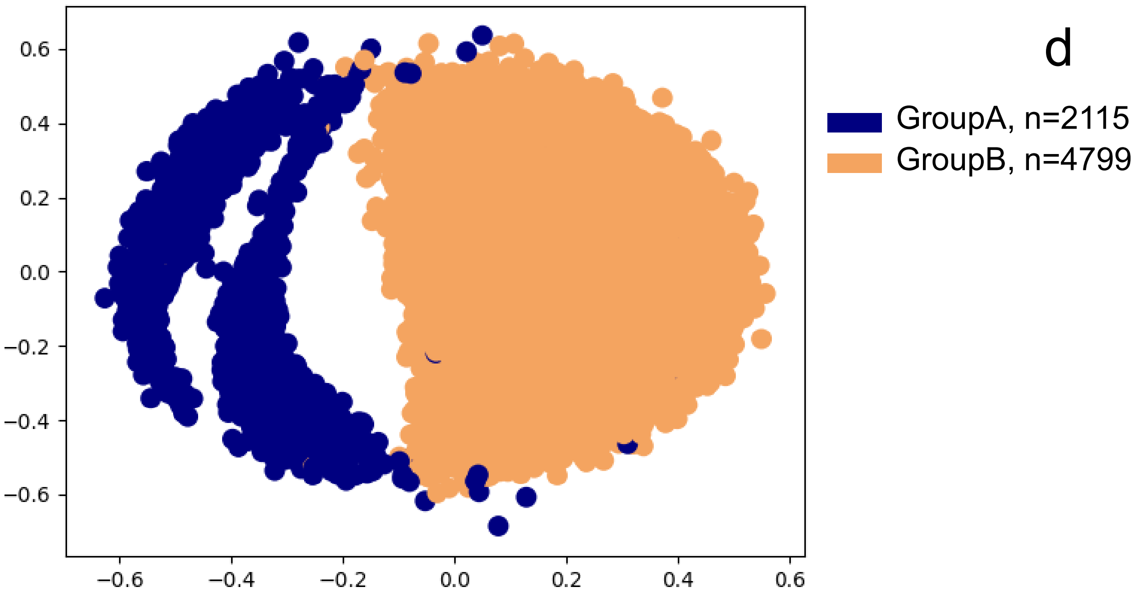

d

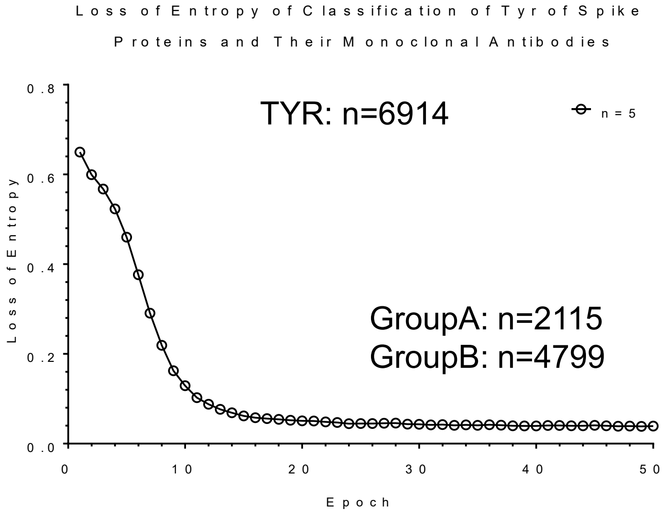

Supplementary Figure 36

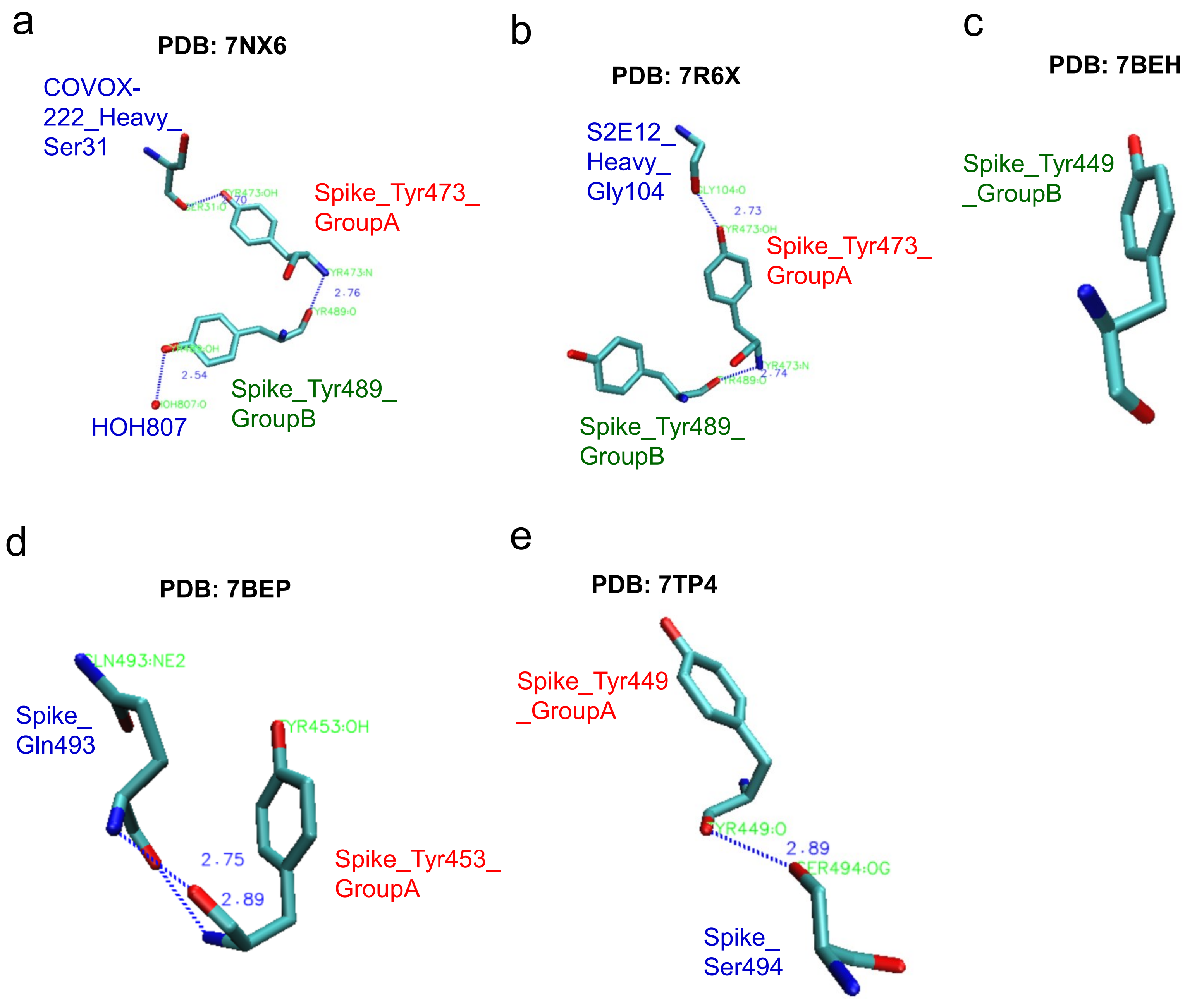

Supplementary Figure 37

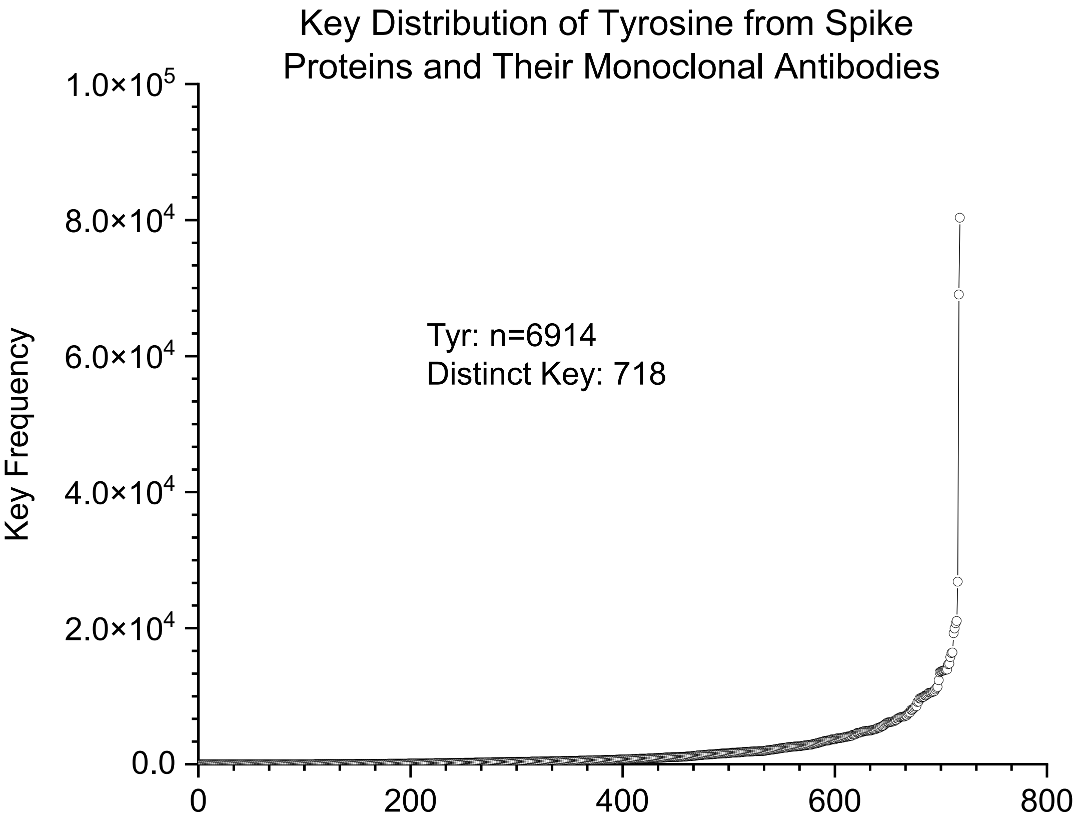

Supplementary Figure 38

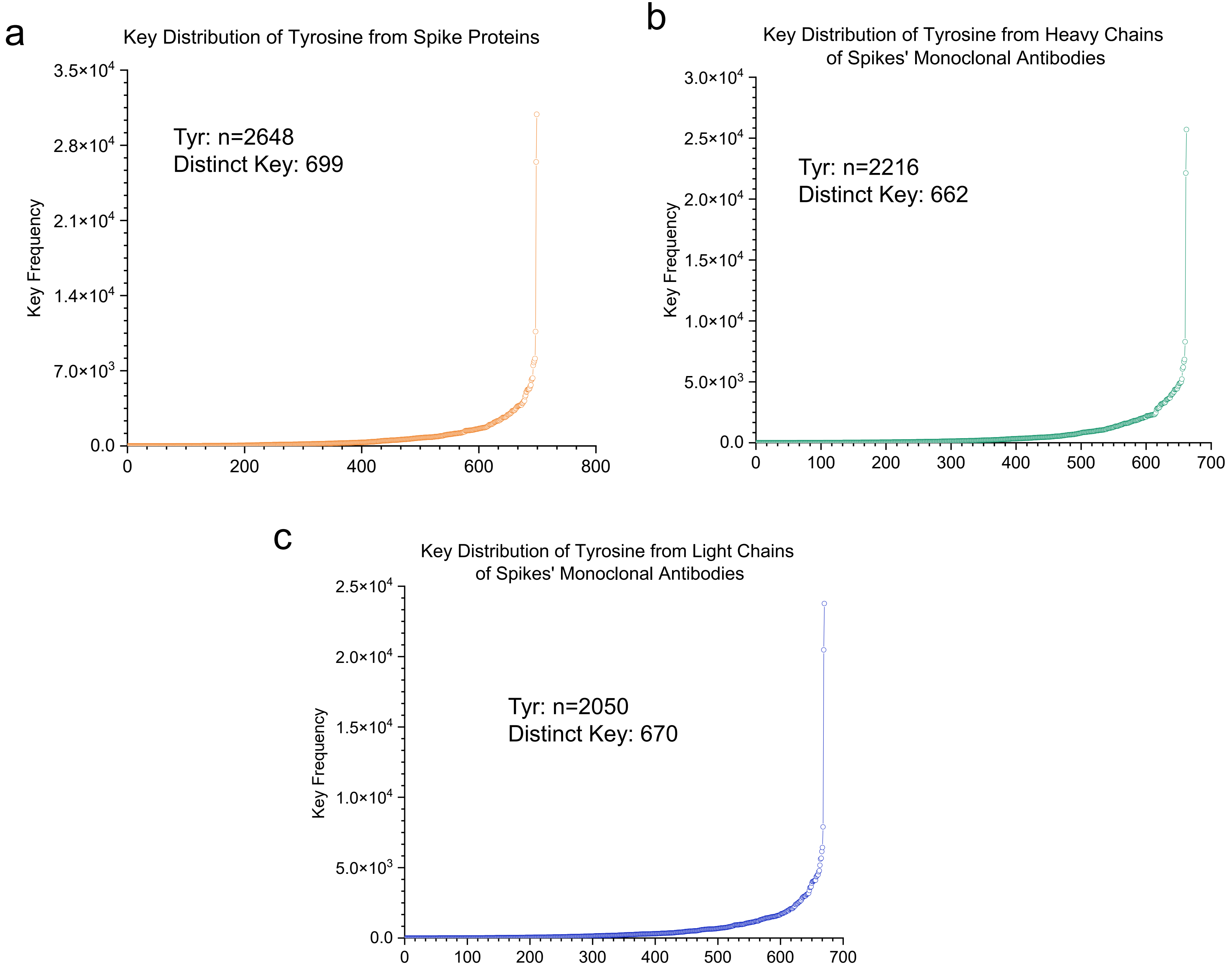

Supplementary Figure 39

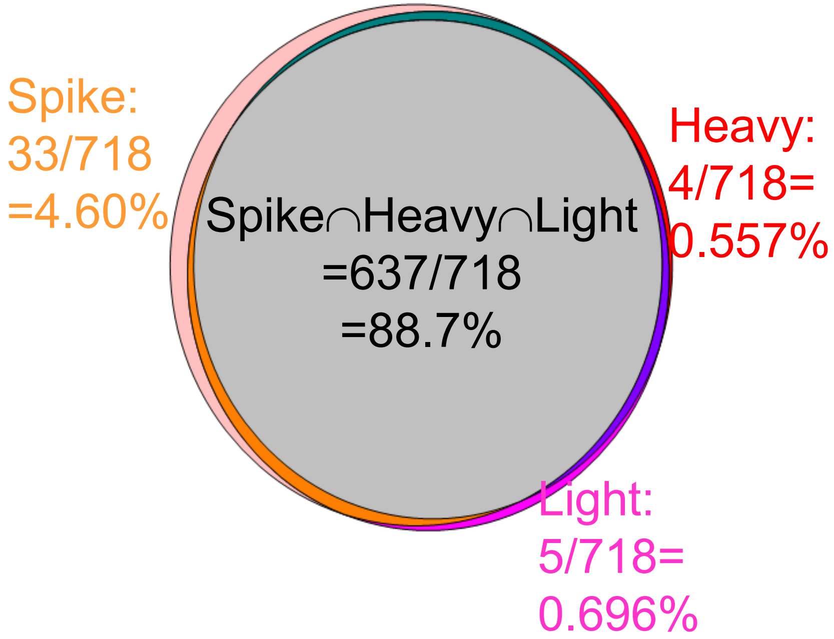

Supplementary Figure 40

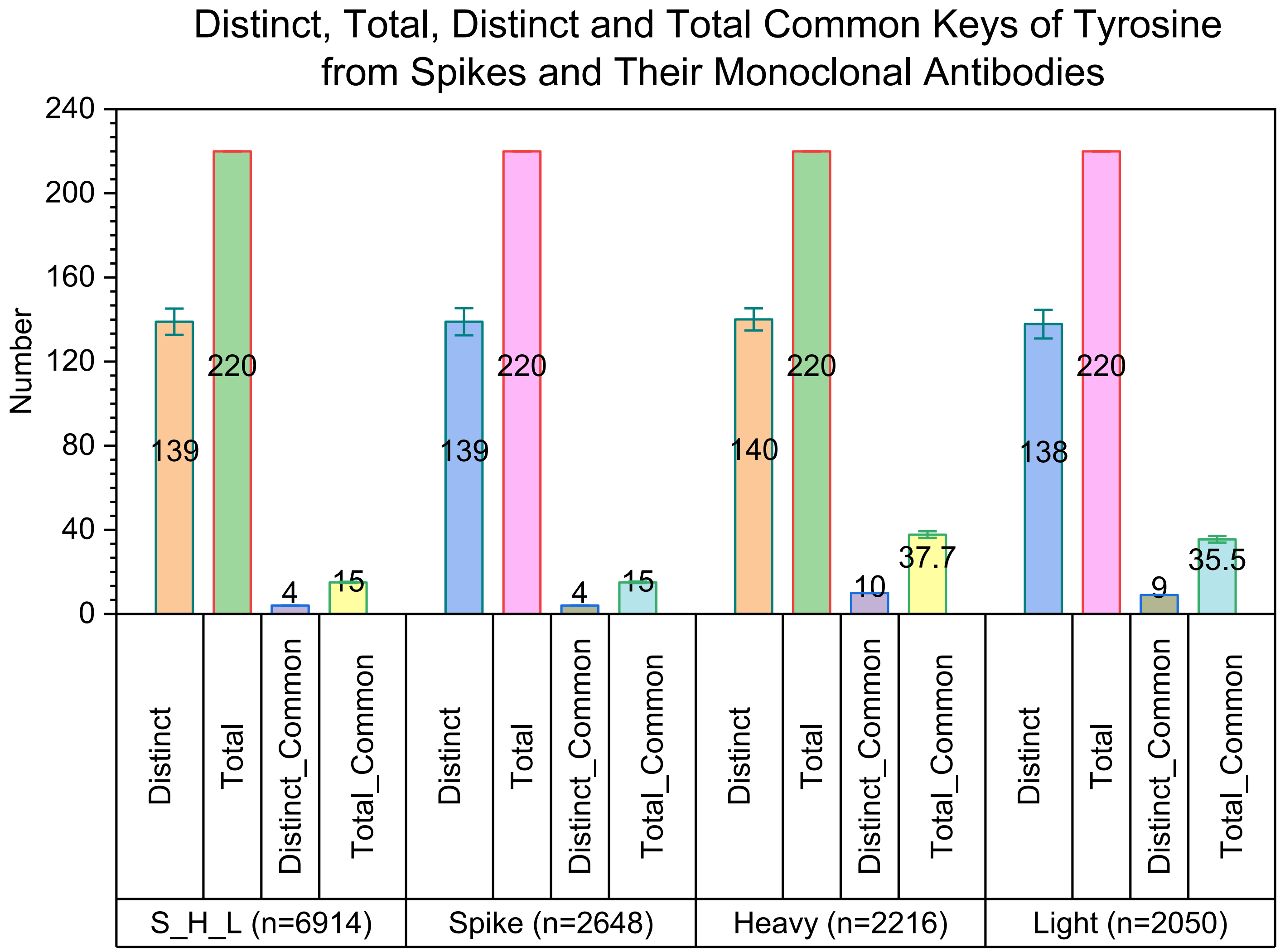

Supplementary Figure 41

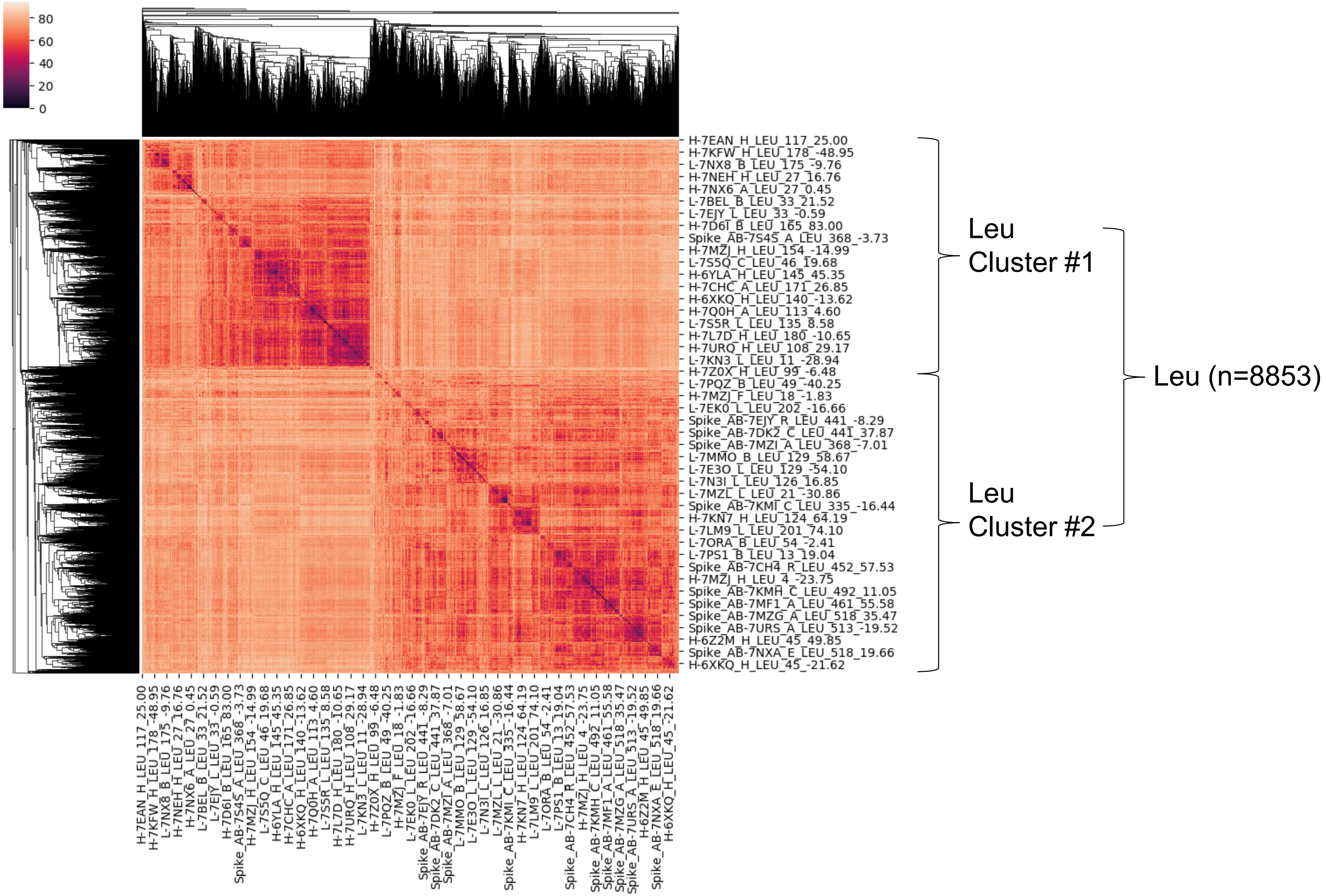

Supplementary Figure 42

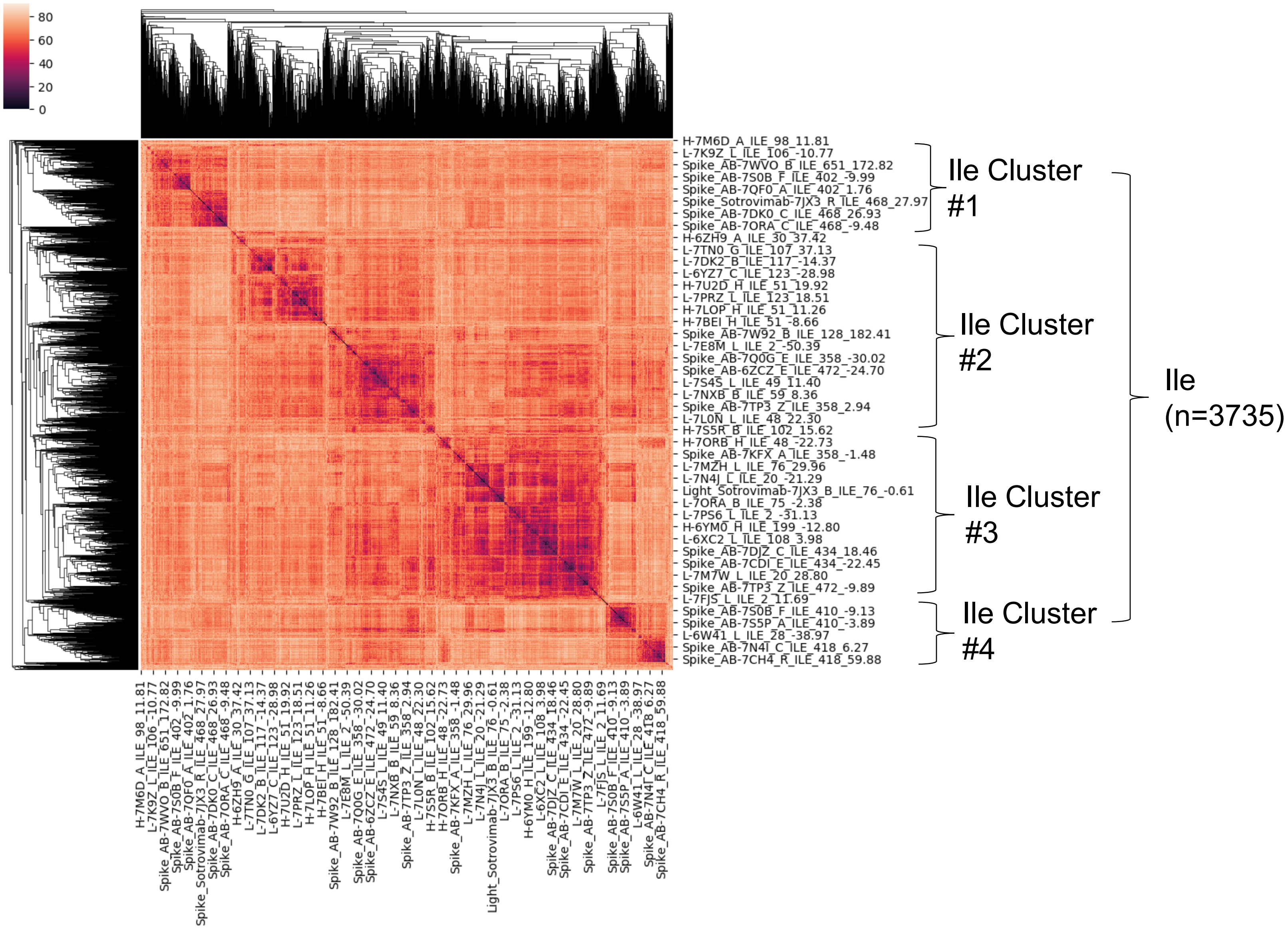

Supplementary Figure 43

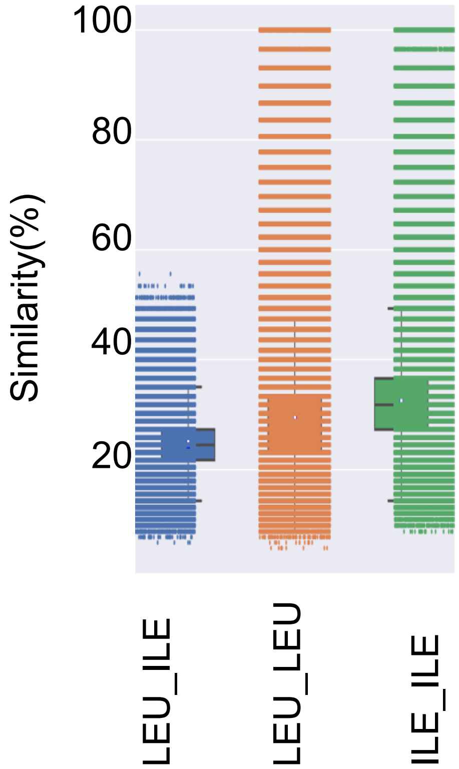

Total: 82216 LEU or ILE

LEU: 57054
ILE: 25162

Supplementary Figure 44

a

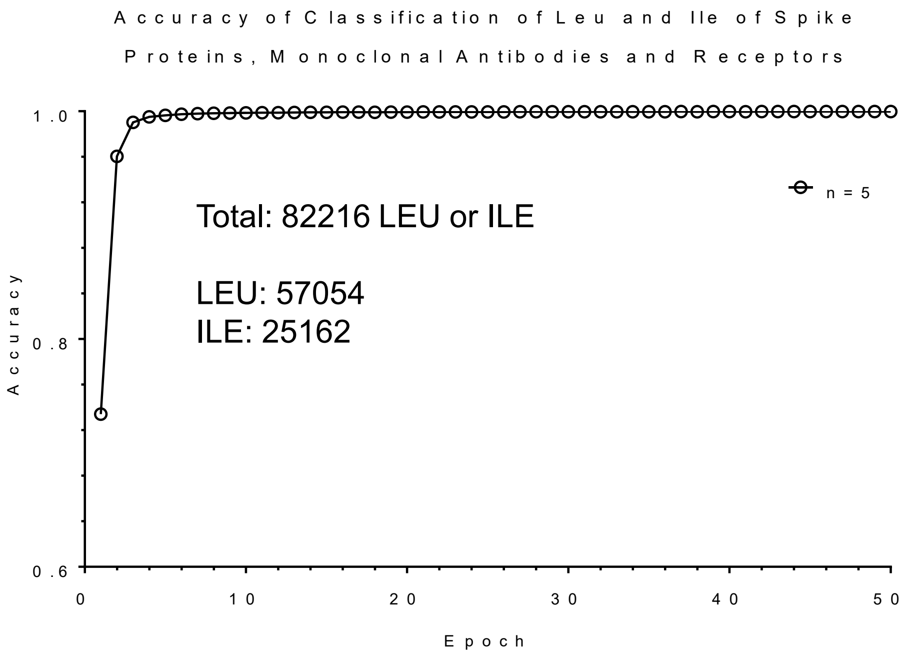

b

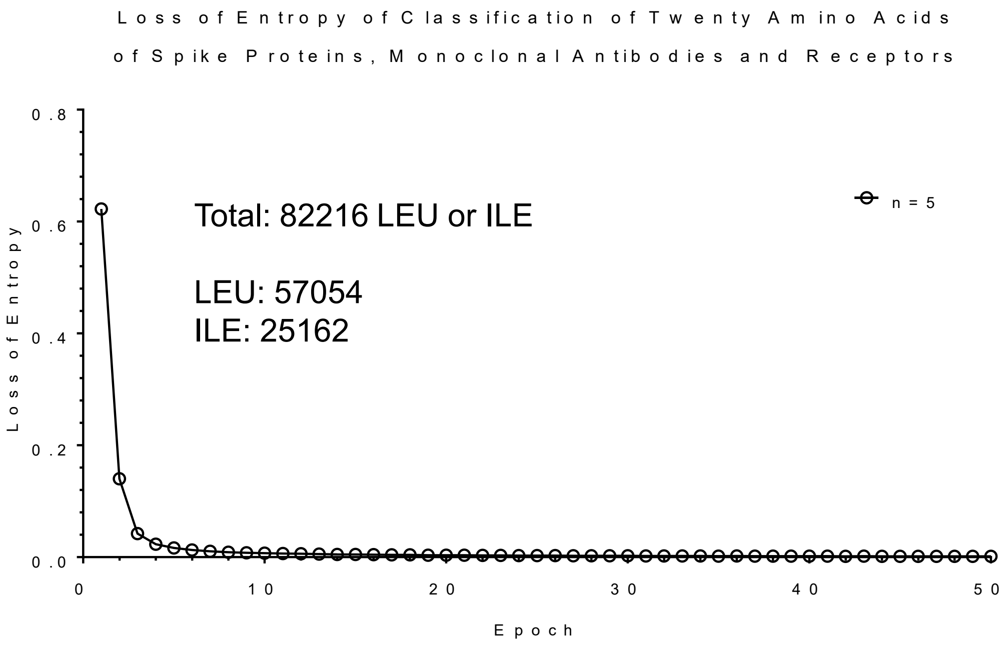

Supplementary Figure 45

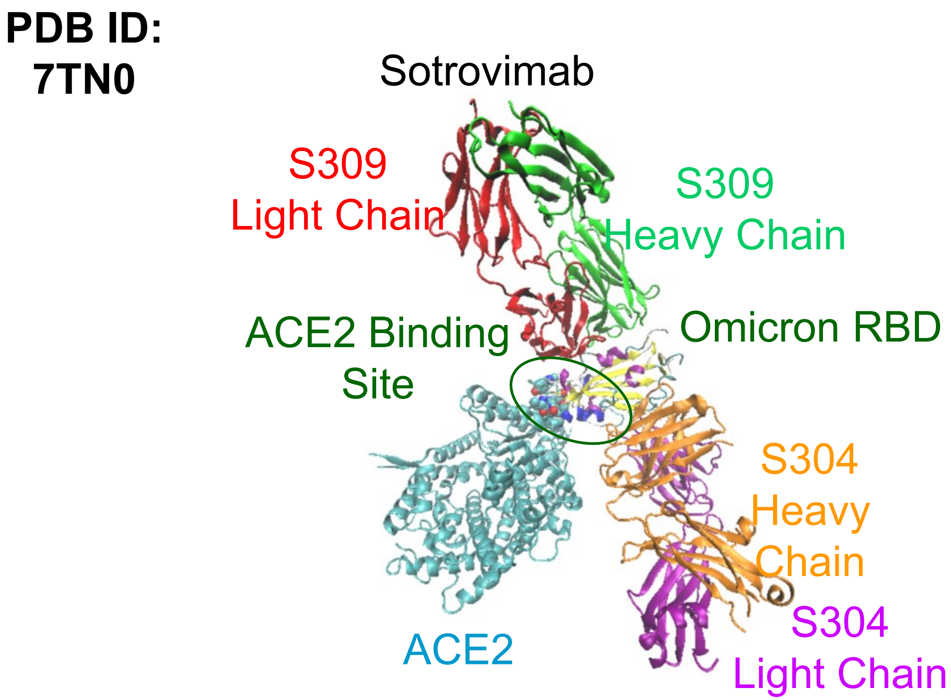

Supplementary Figure 46

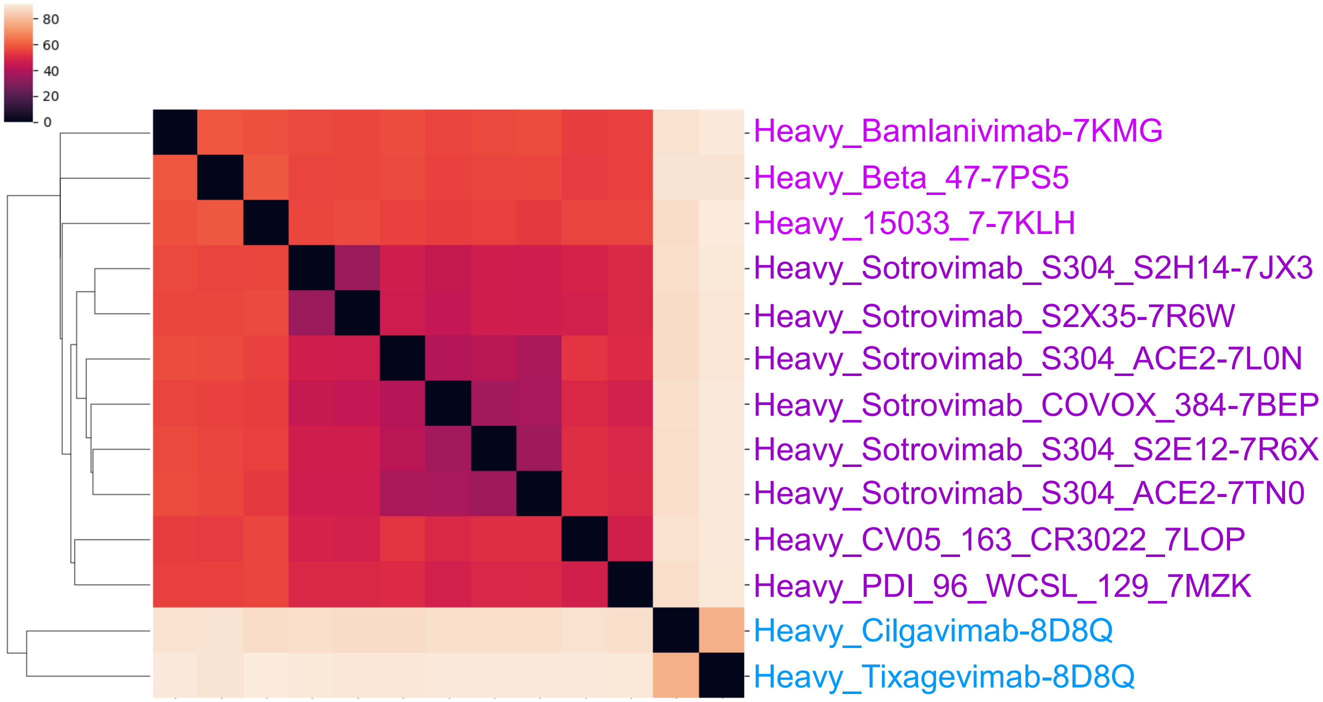

Supplementary Figure 47

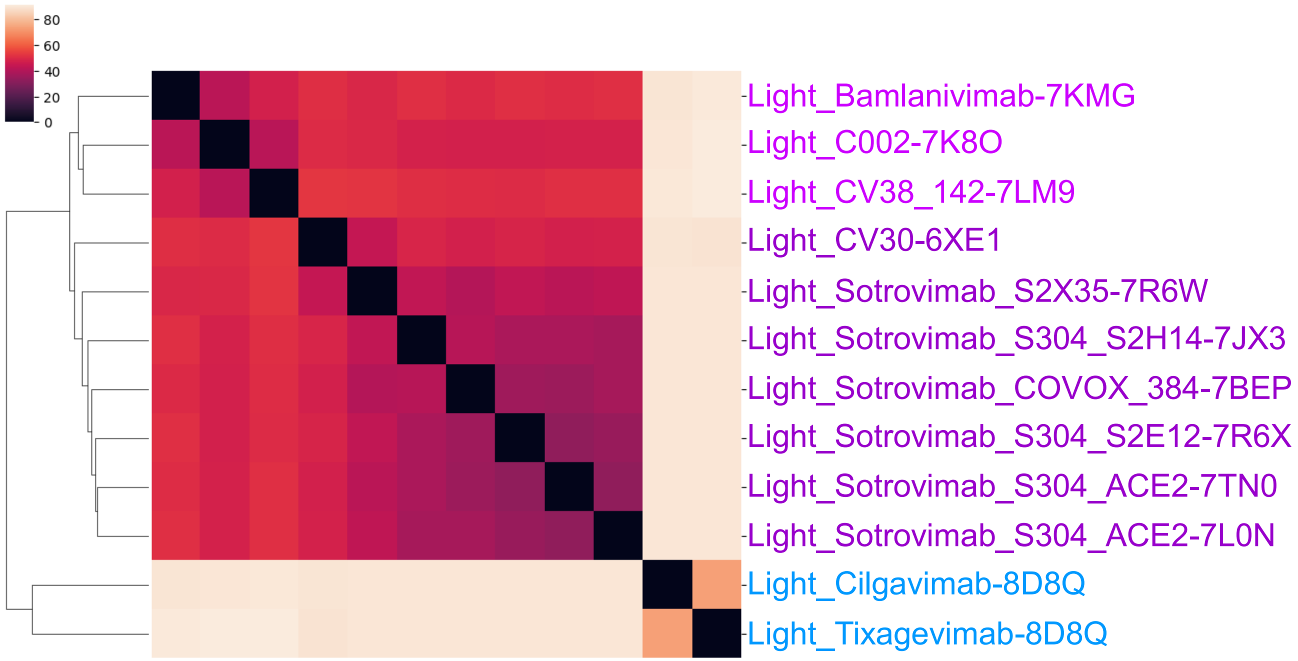

Supplementary Figure 48

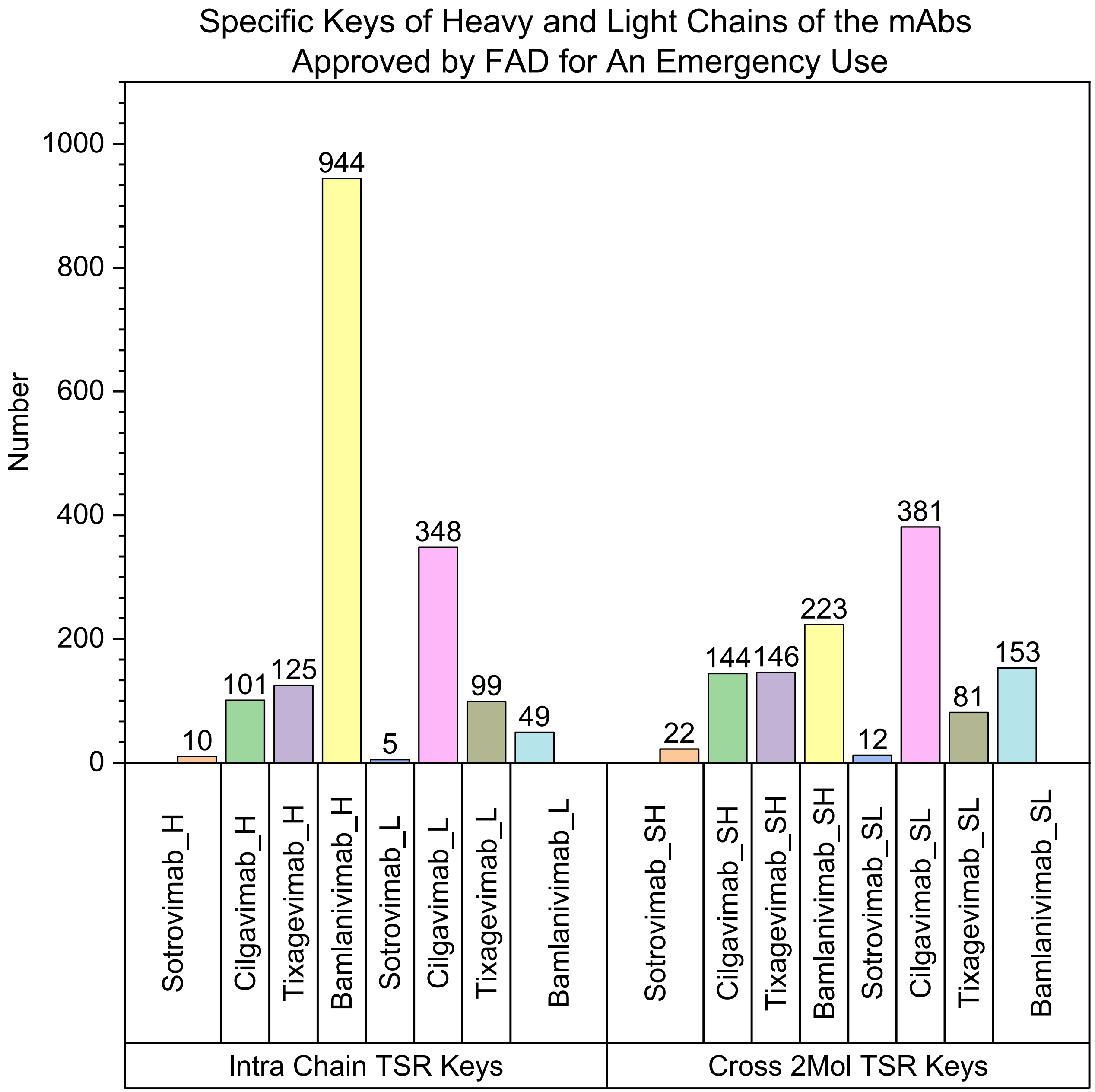

Supplementary Figure 49

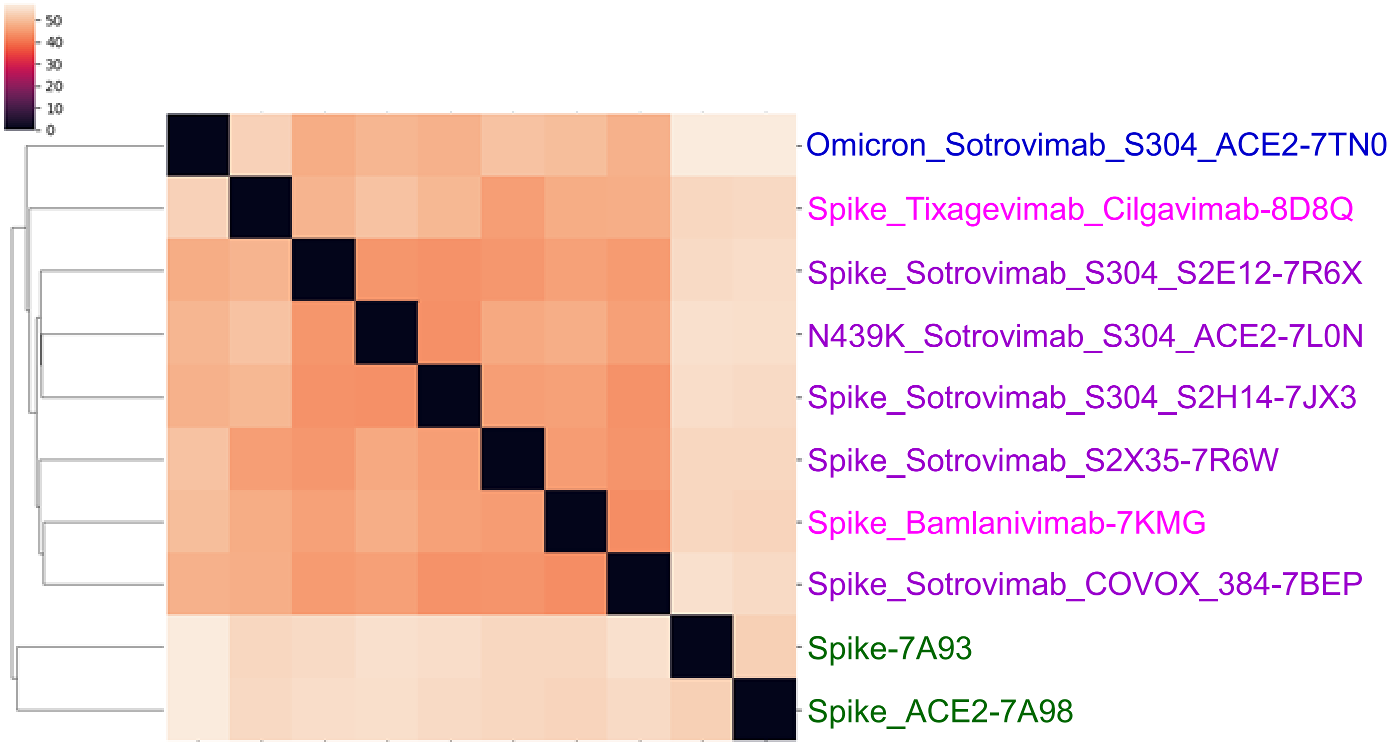

Supplementary Figure 50

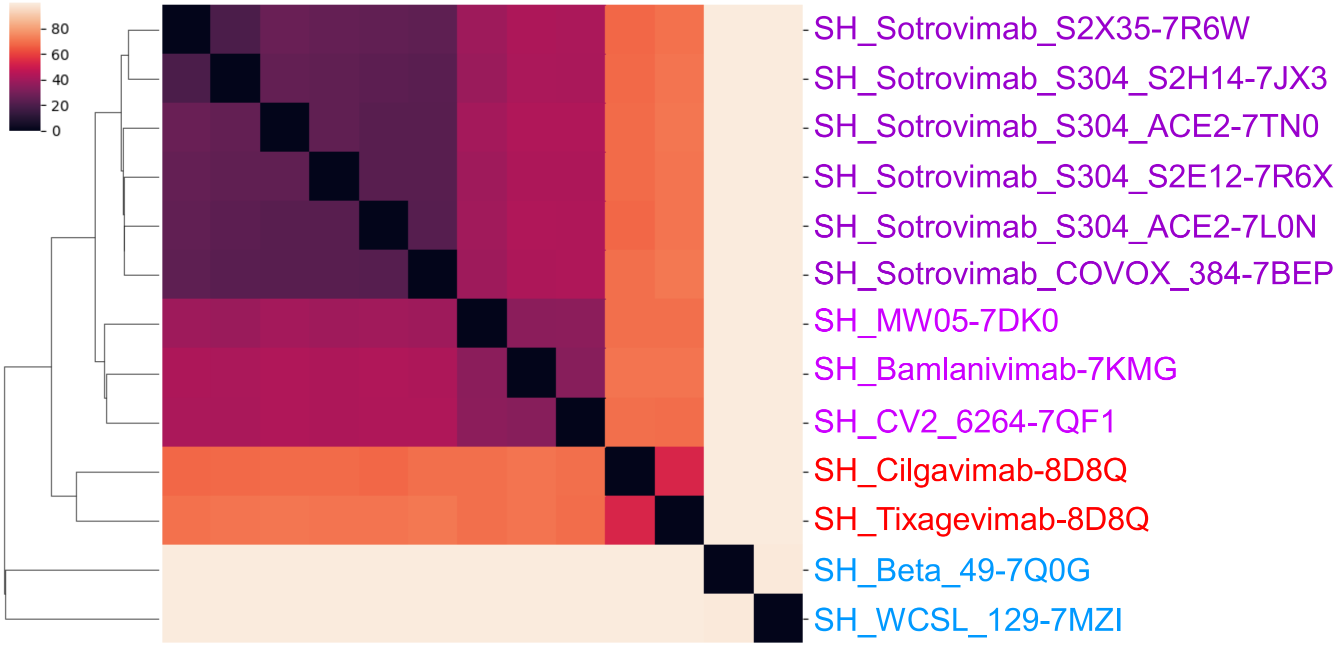

Supplementary Figure 51

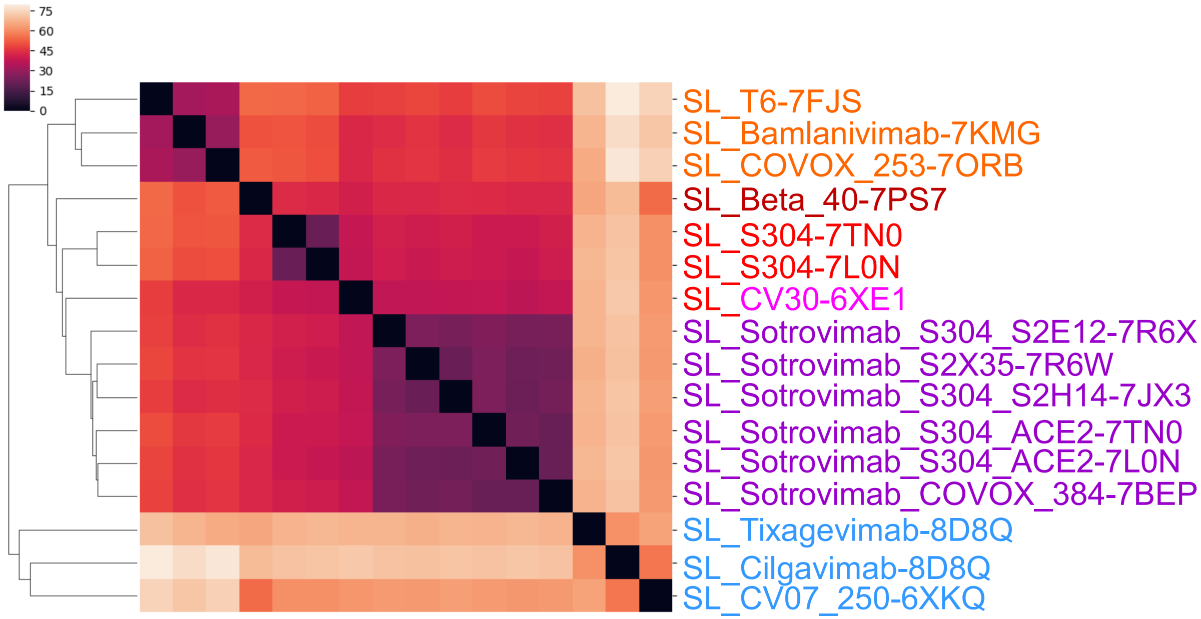

Supplementary Figure 52

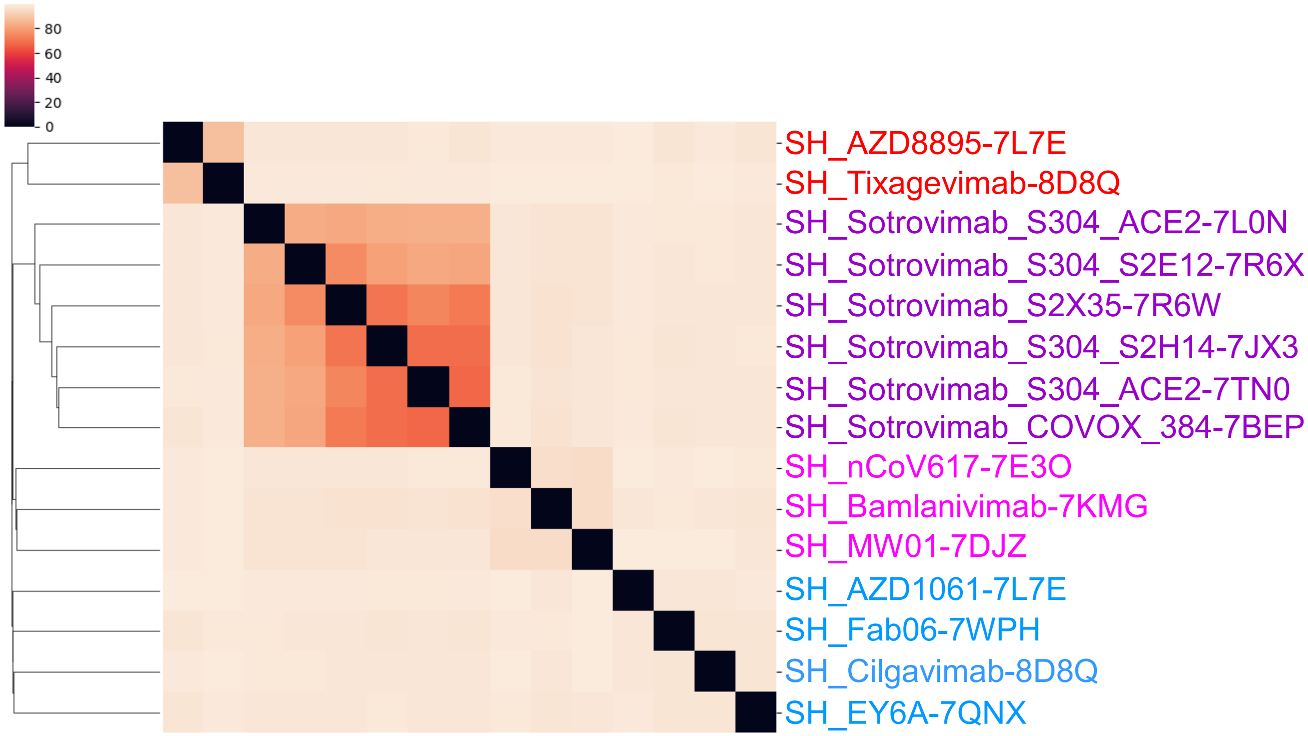

Supplementary Figure 53

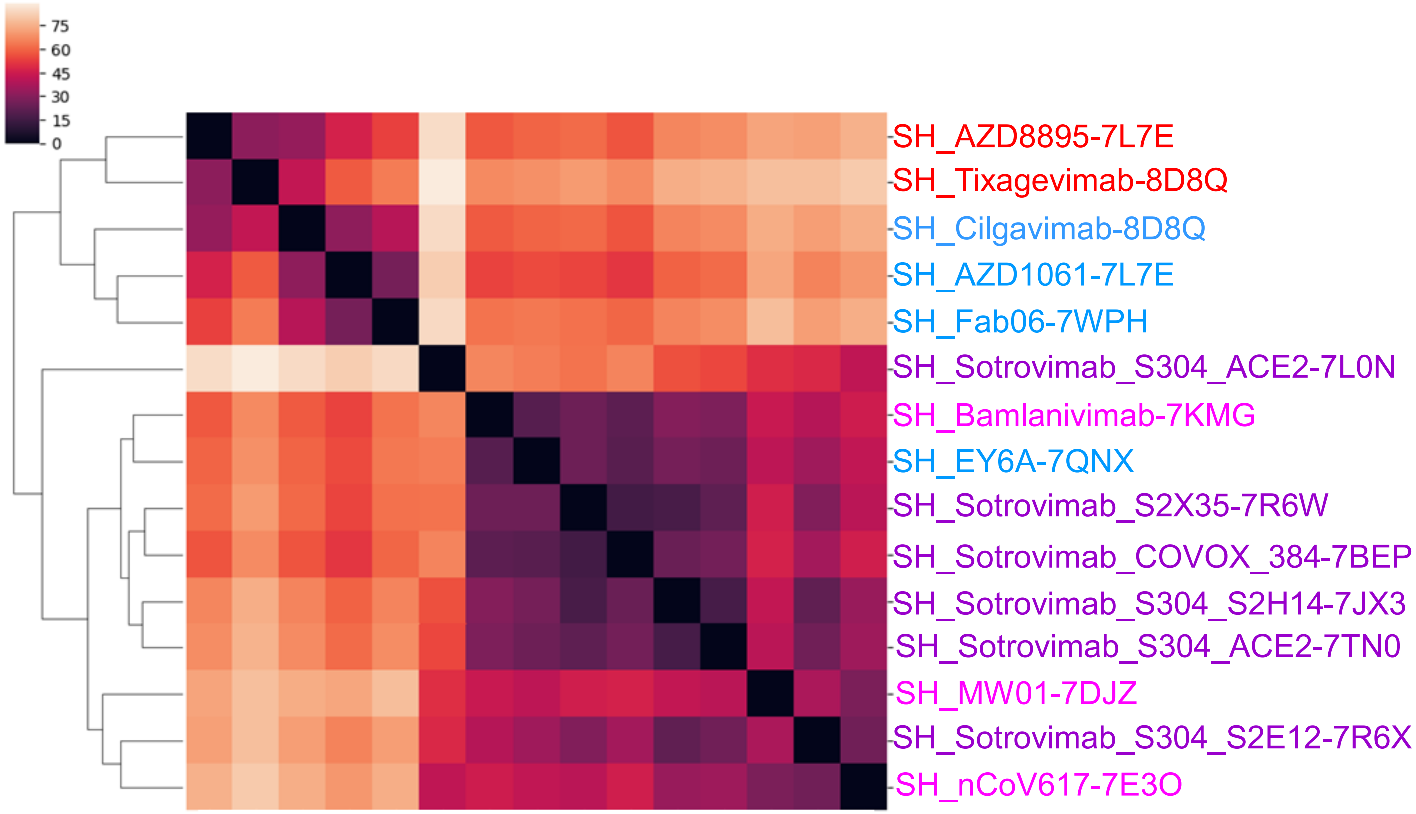

Supplementary Figure 54

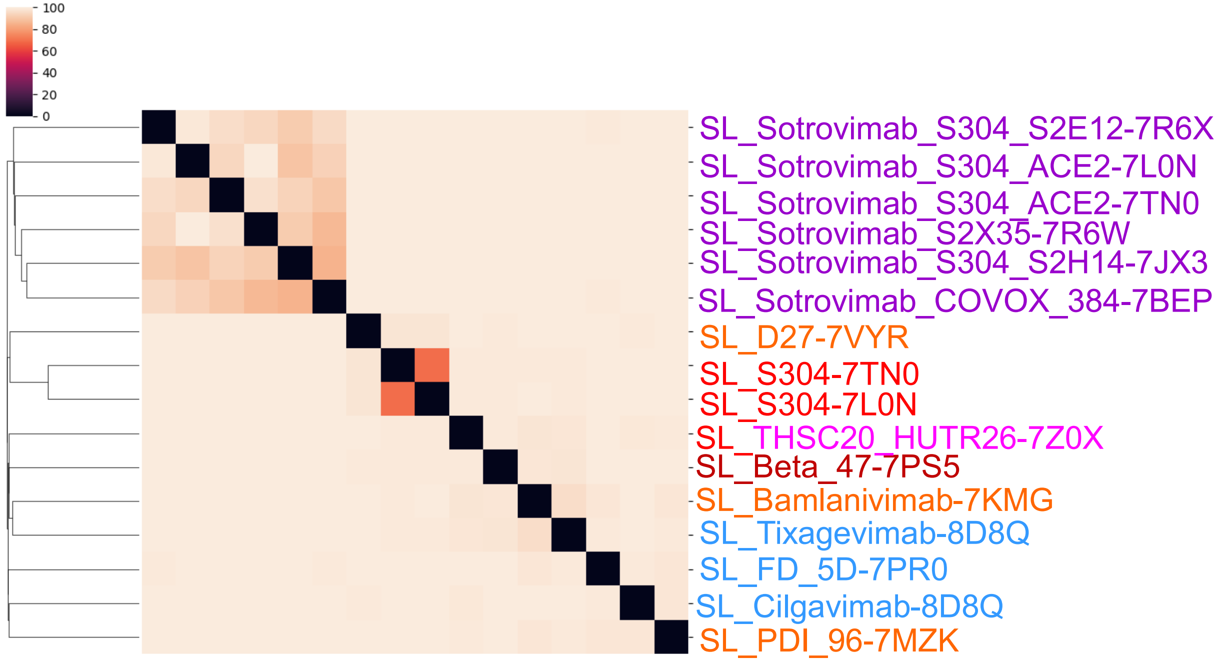

Supplementary Figure 55

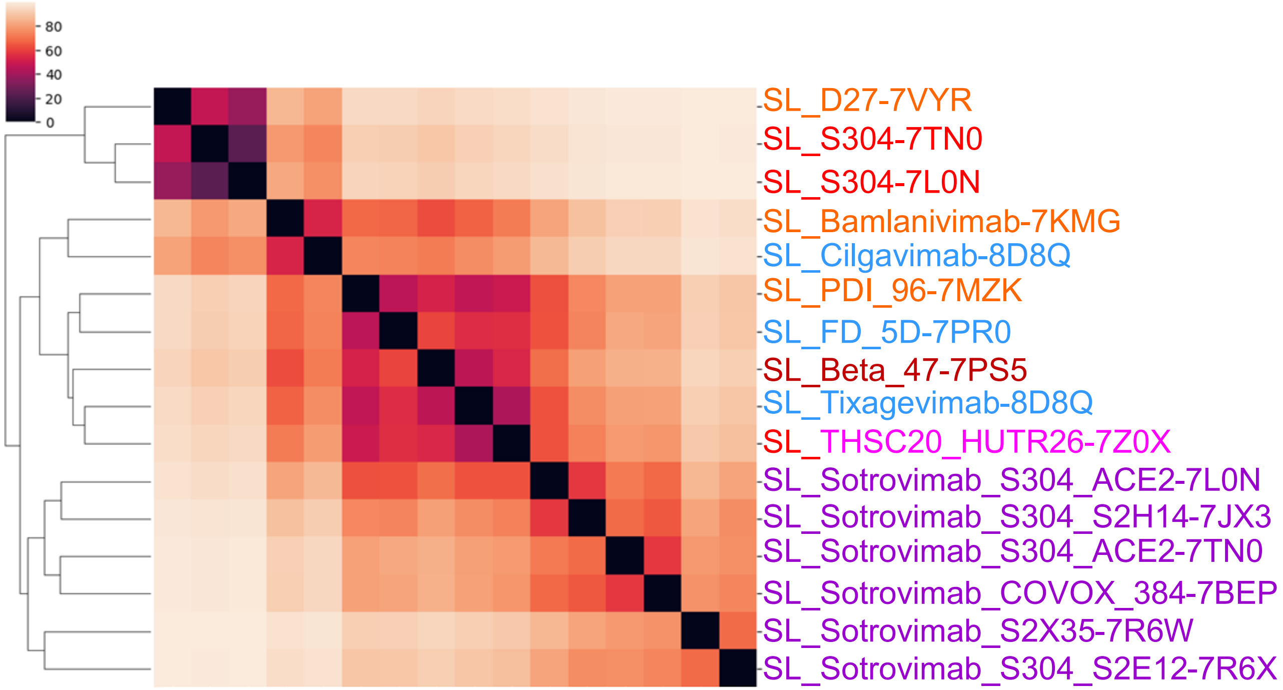

Supplementary Figure 56

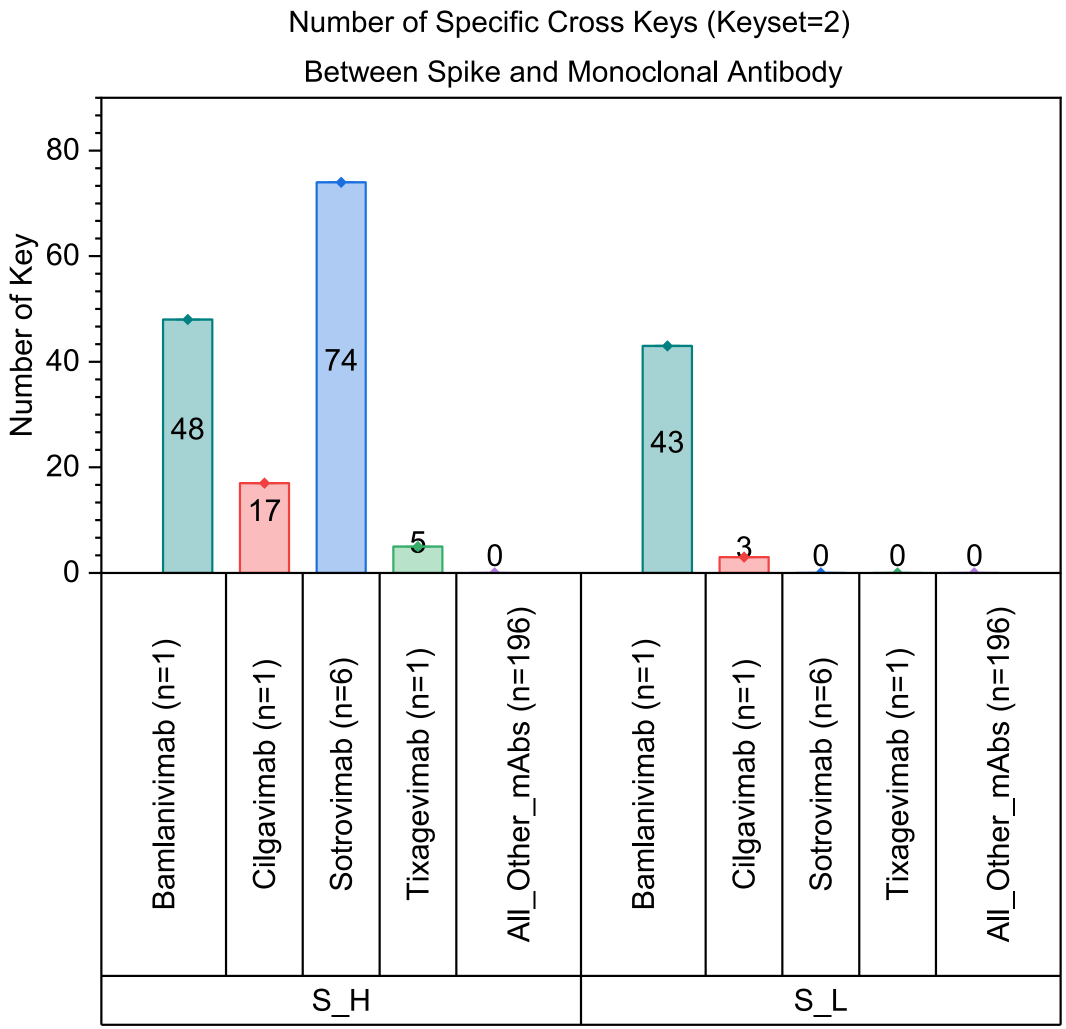

Supplementary Figure 57

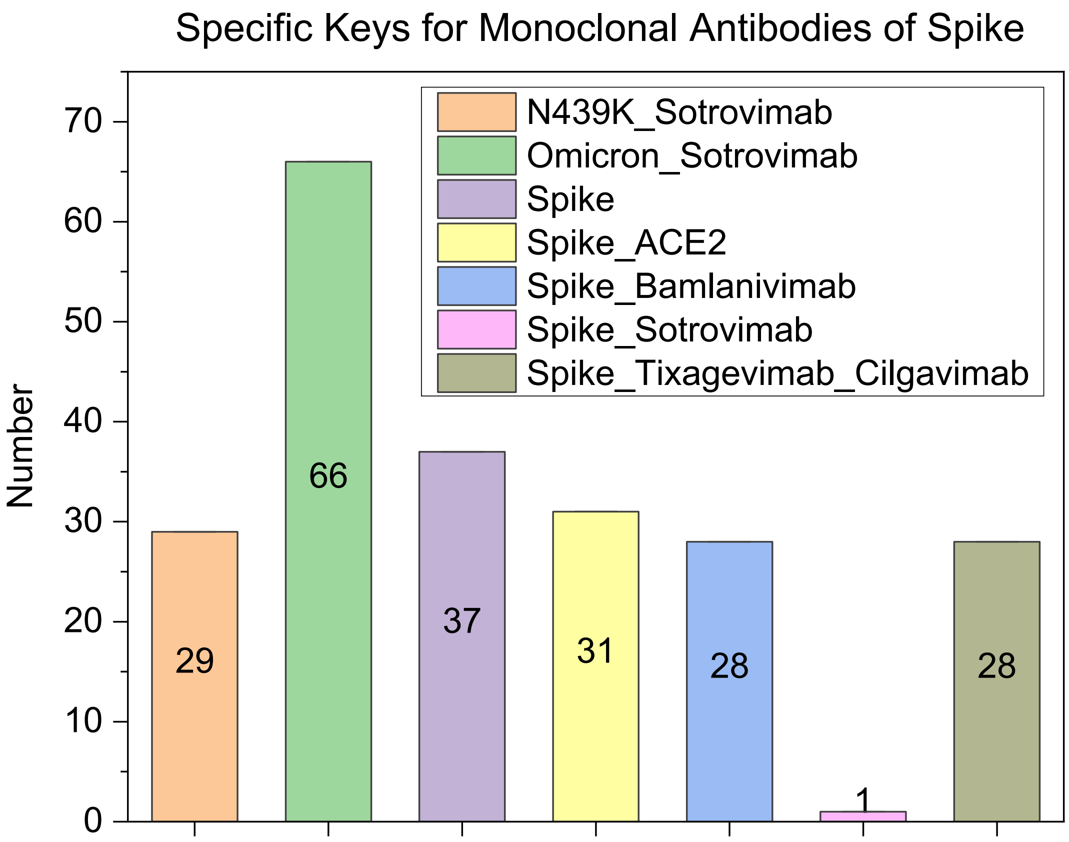

Supplementary Figure 58

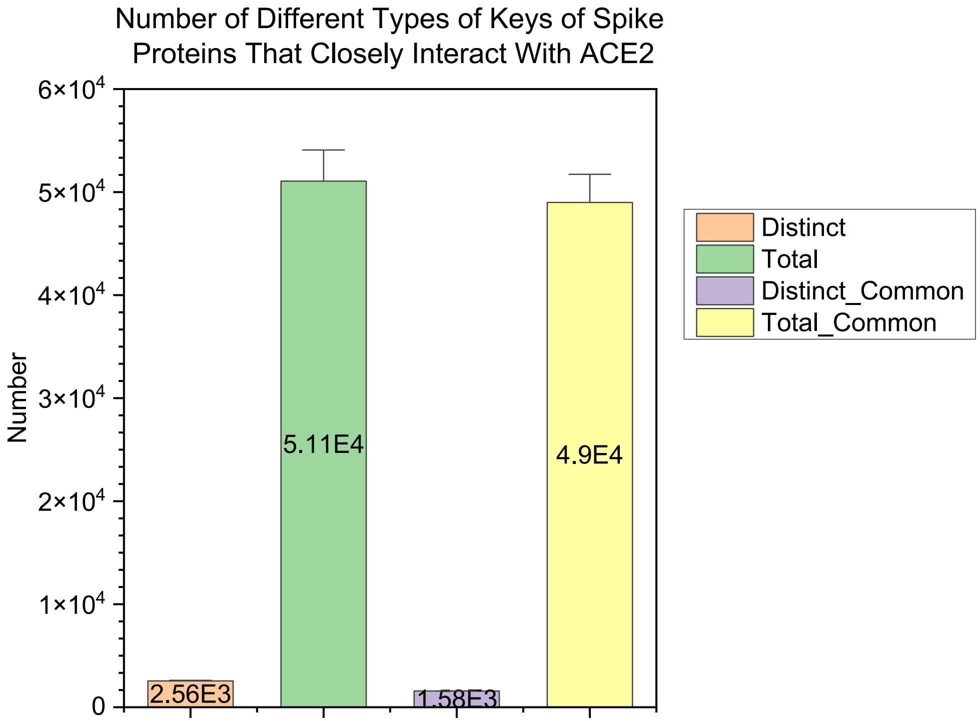

Supplementary Figure 59

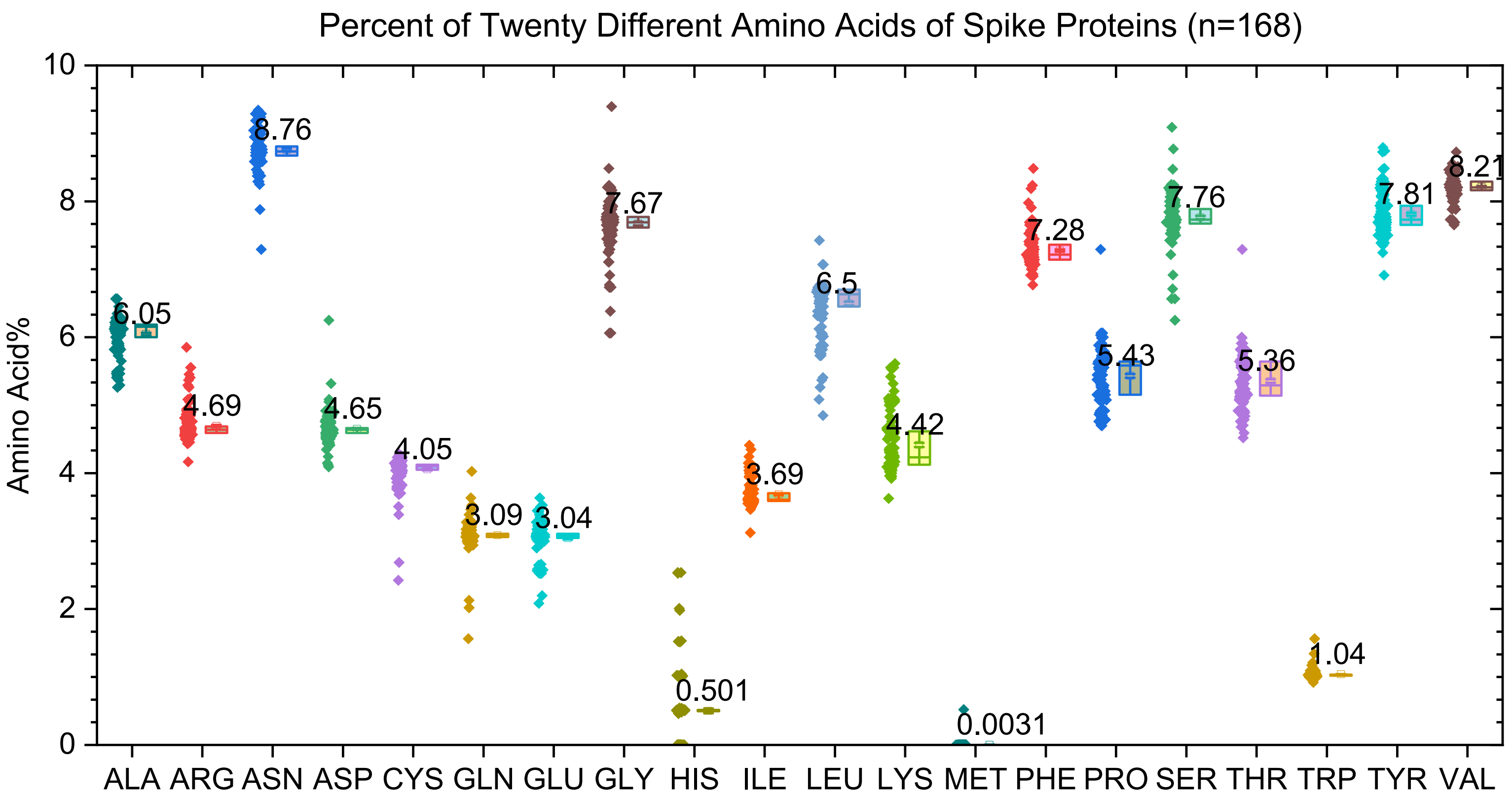

Supplementary Figure 60

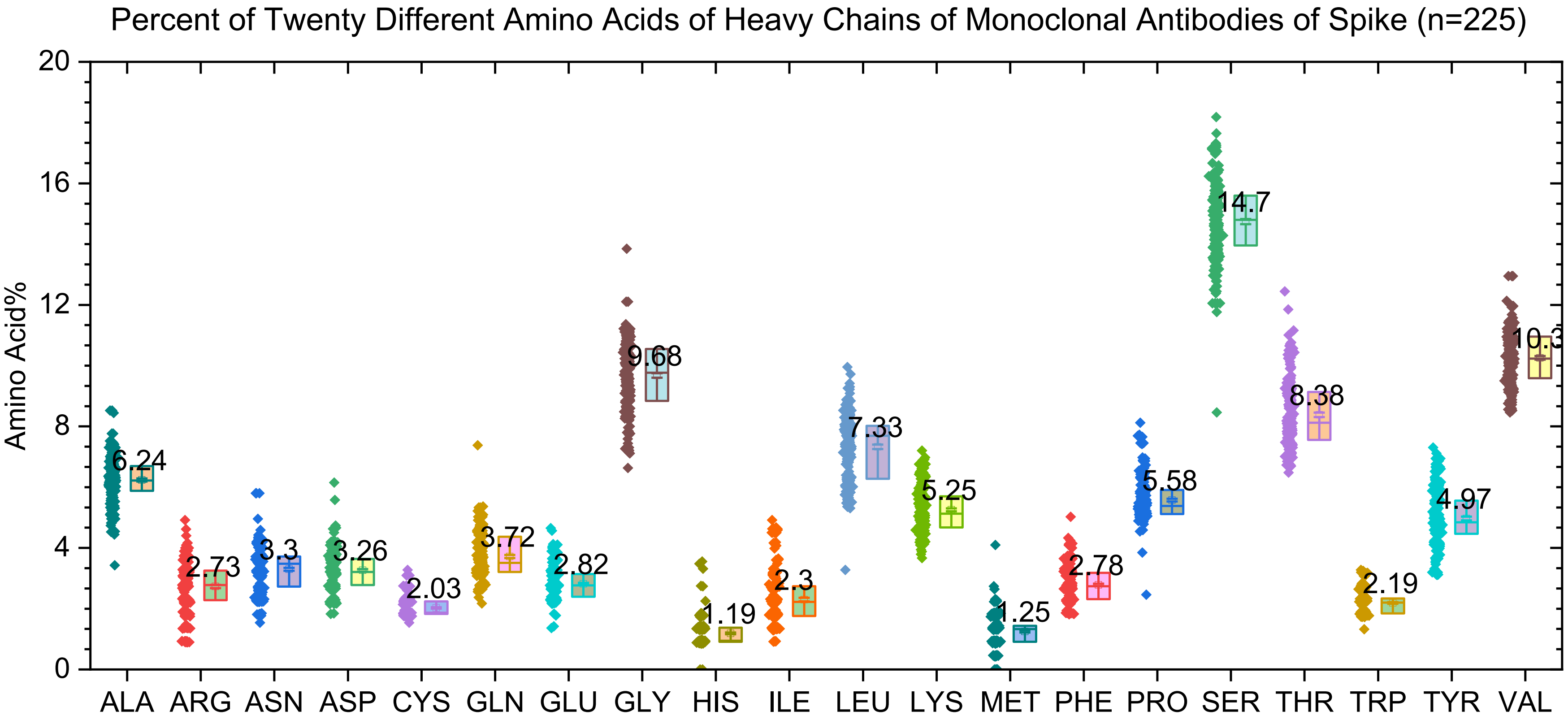

Supplementary Figure 61

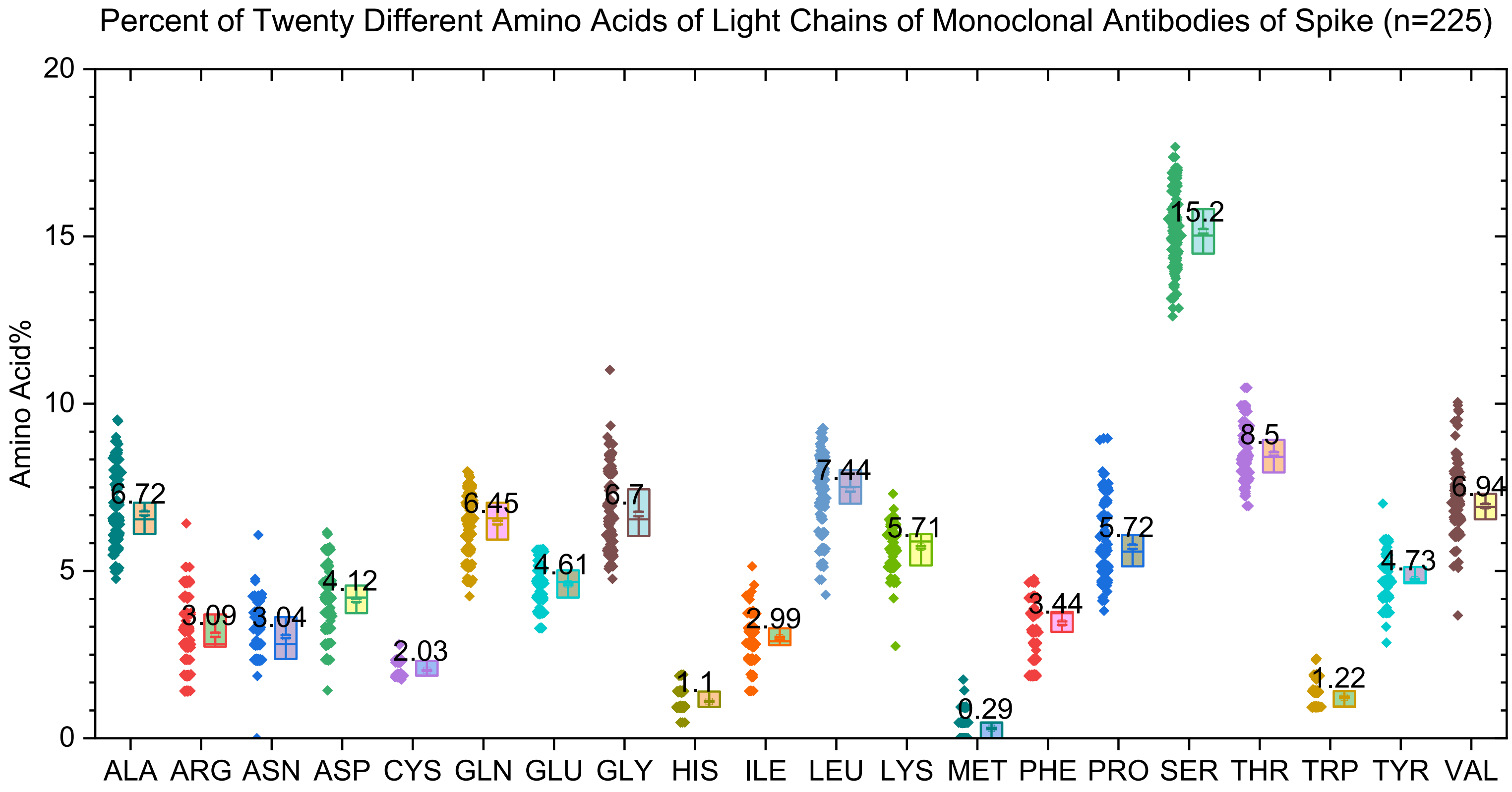

Supplementary Figure 62

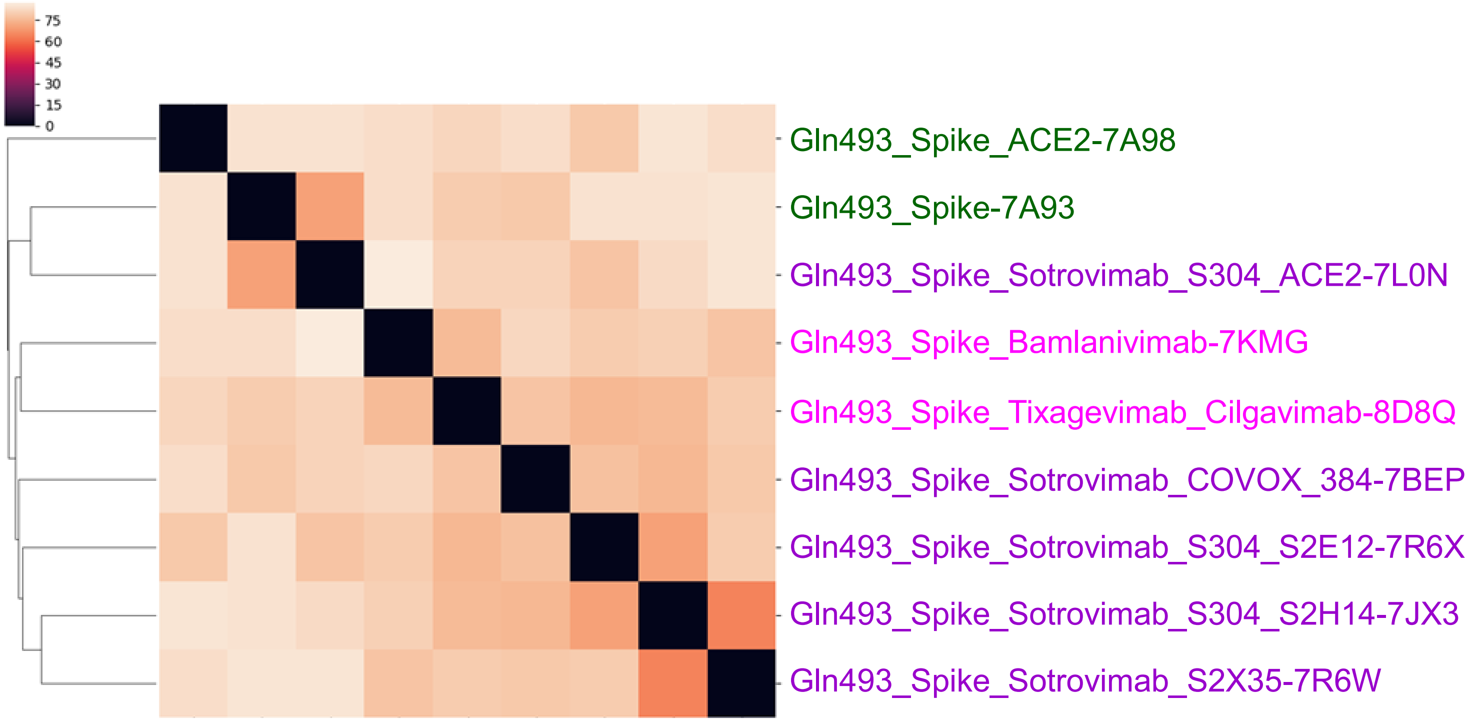

Supplementary Figure 63

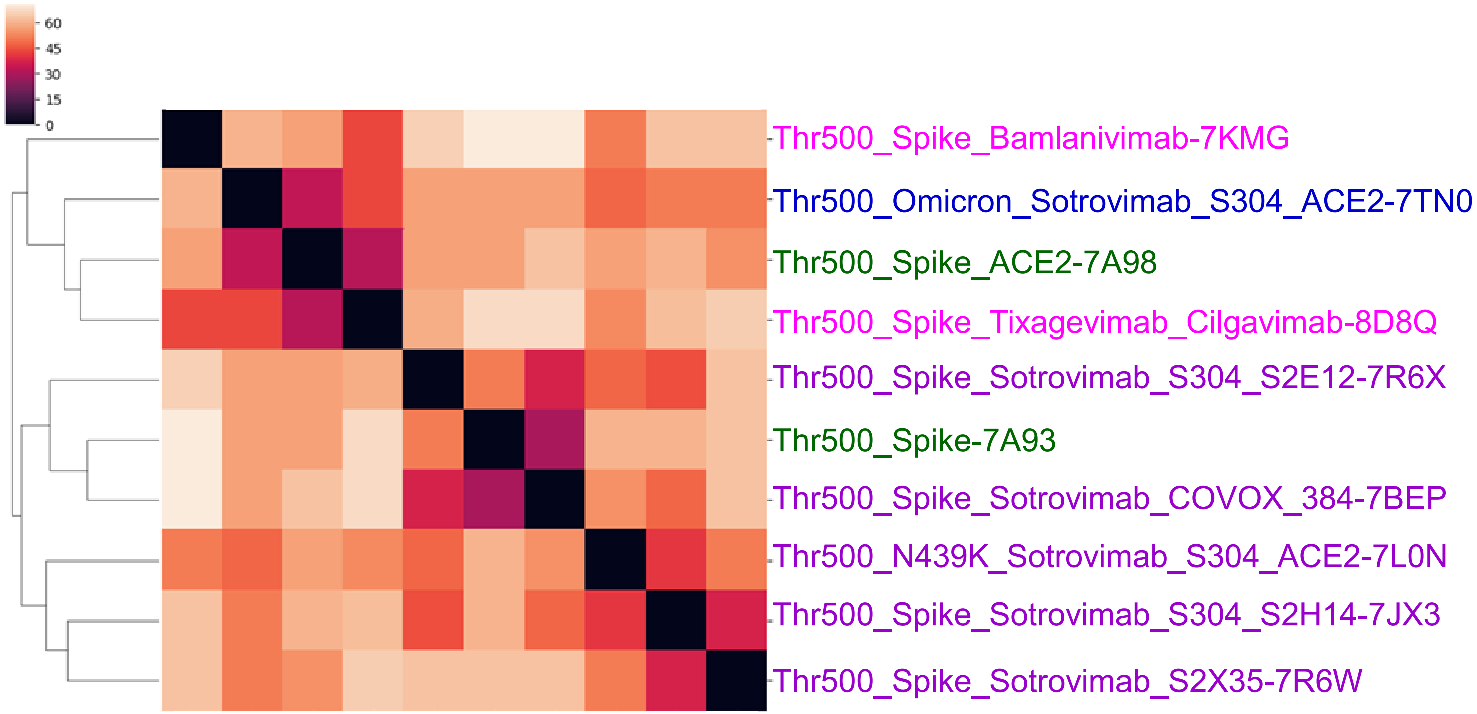

Supplementary Figure 64

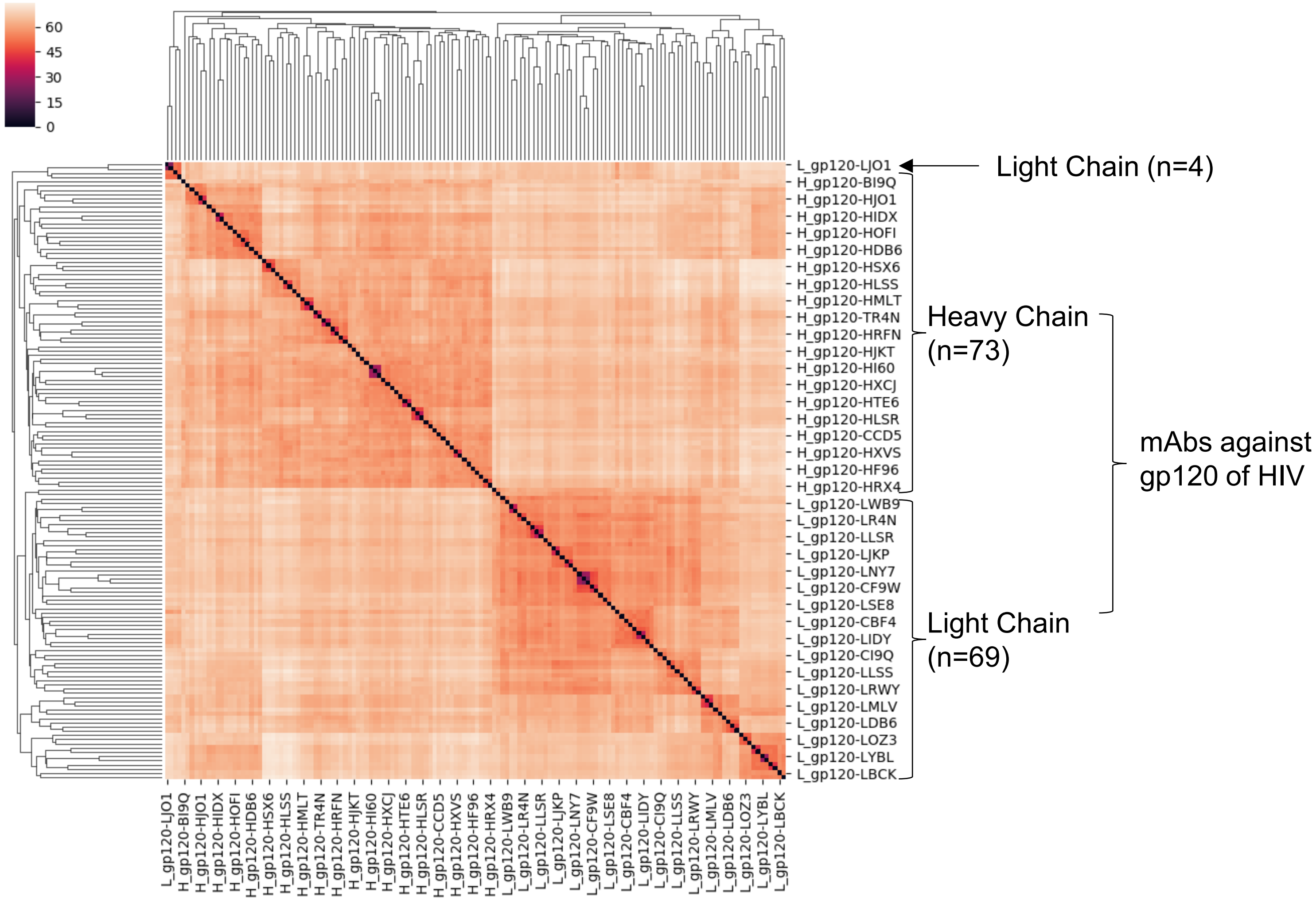

Supplementary Figure 65

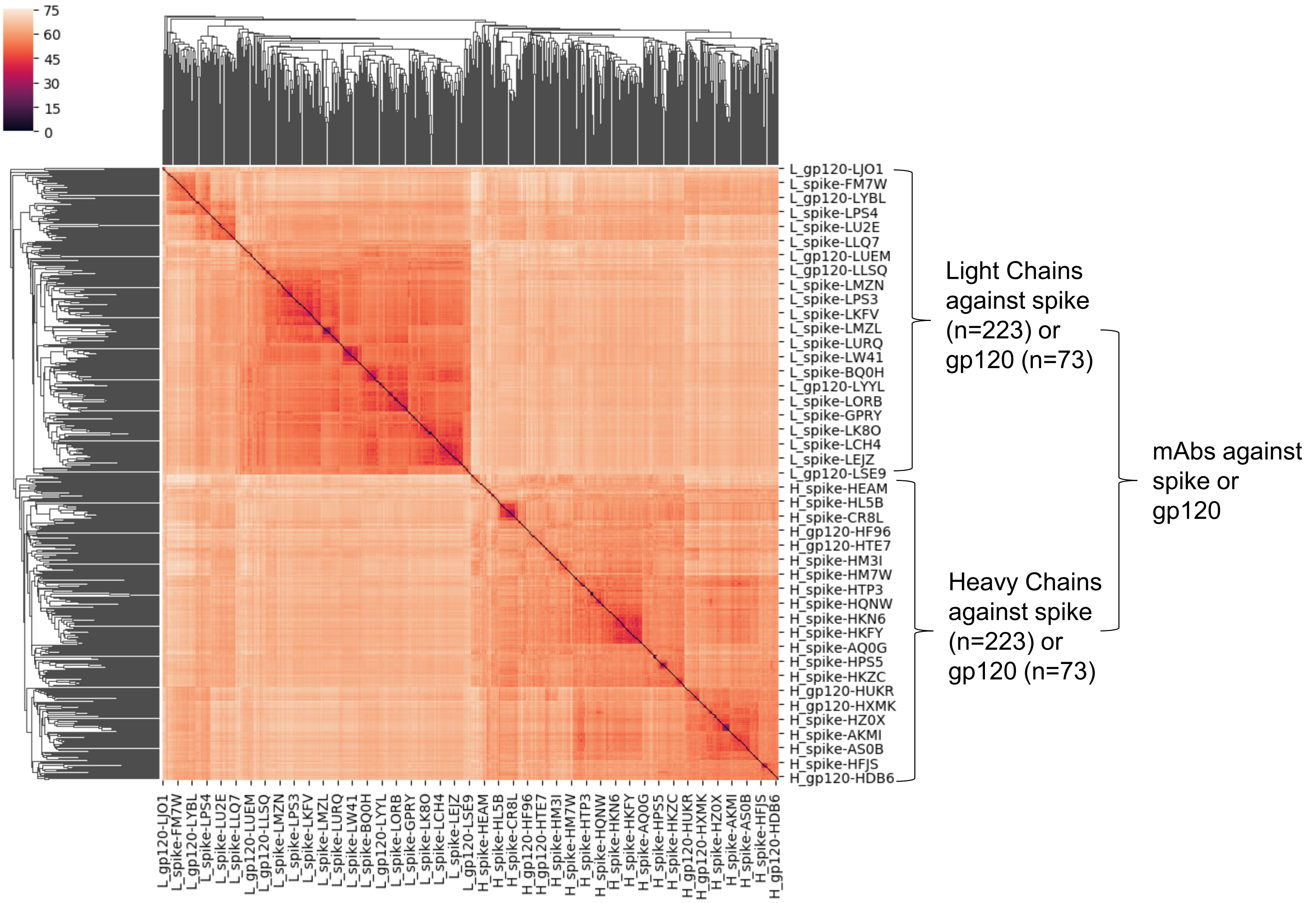

Supplementary Figure 66

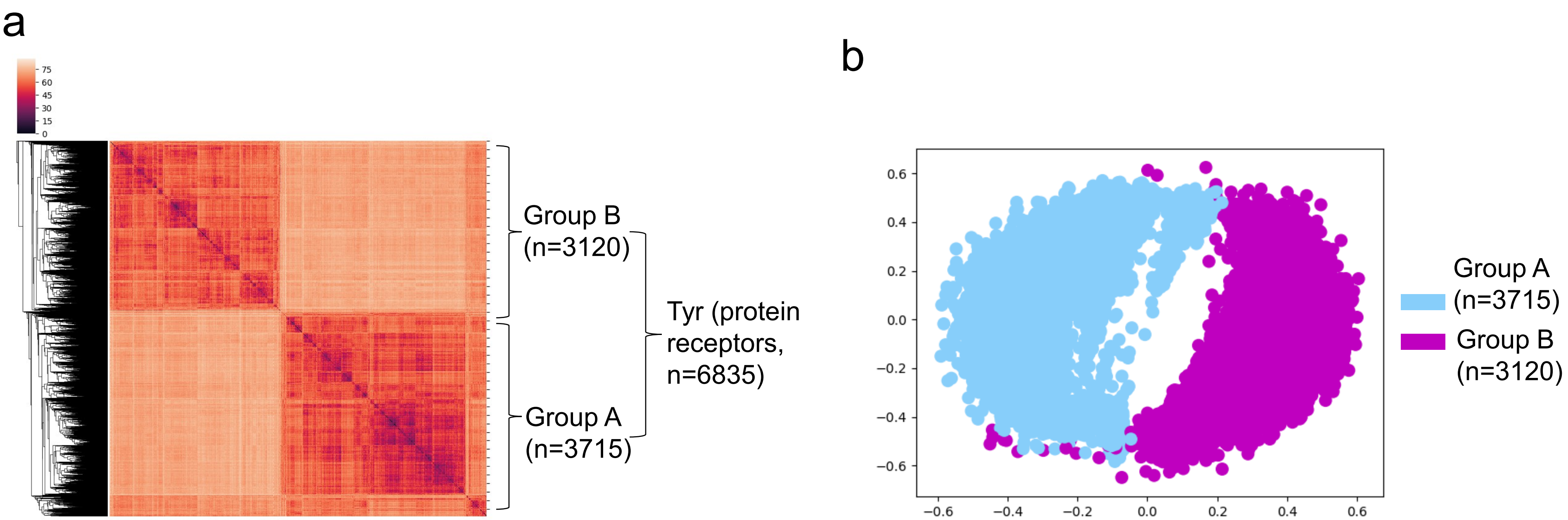

Supplementary Figure 67

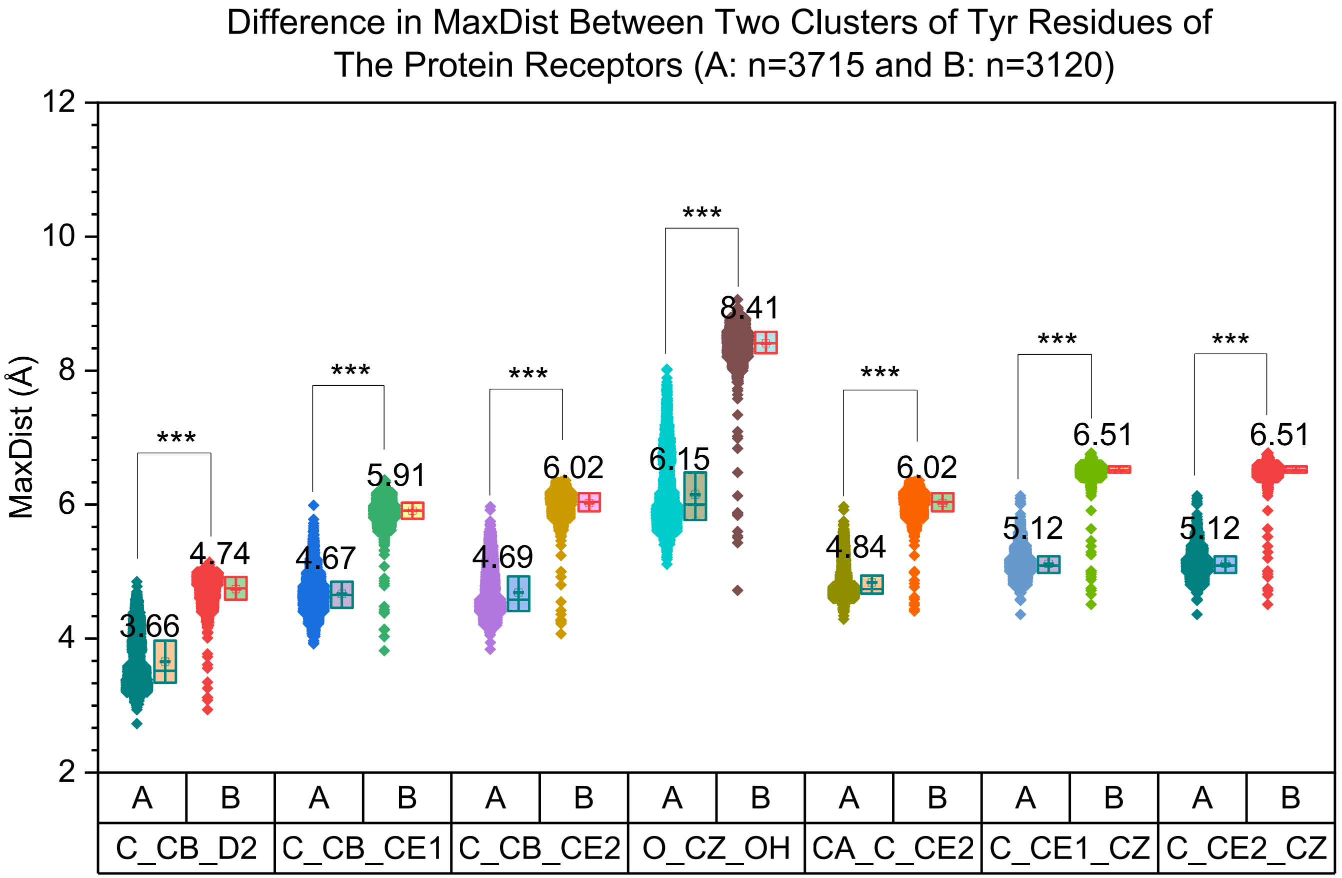

Supplementary Figure 68

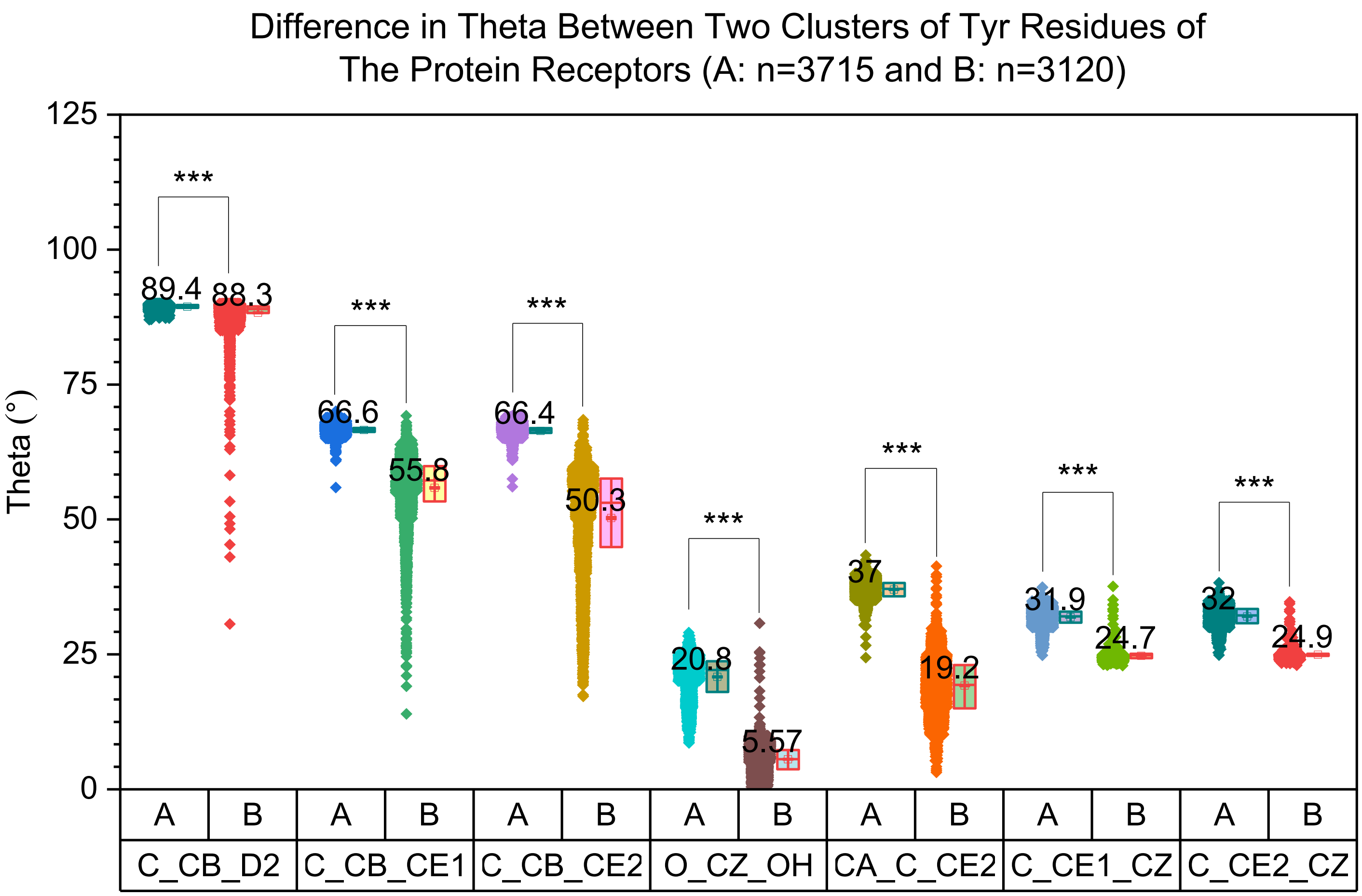

Supplementary Figure 77

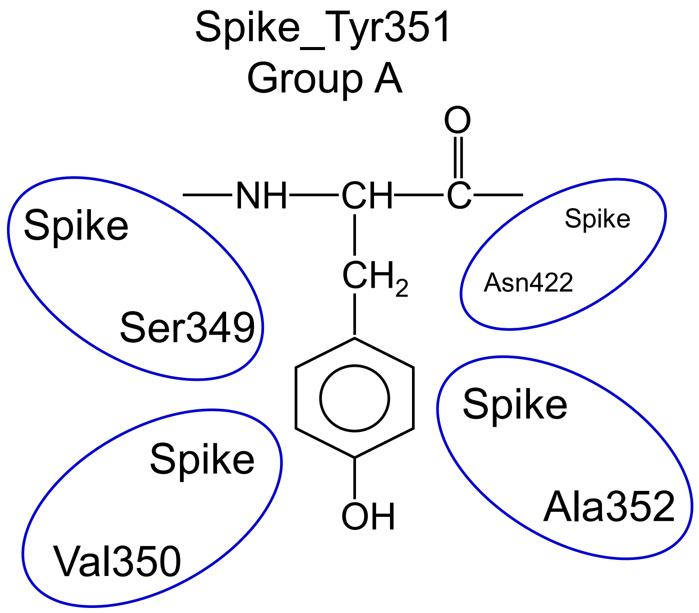

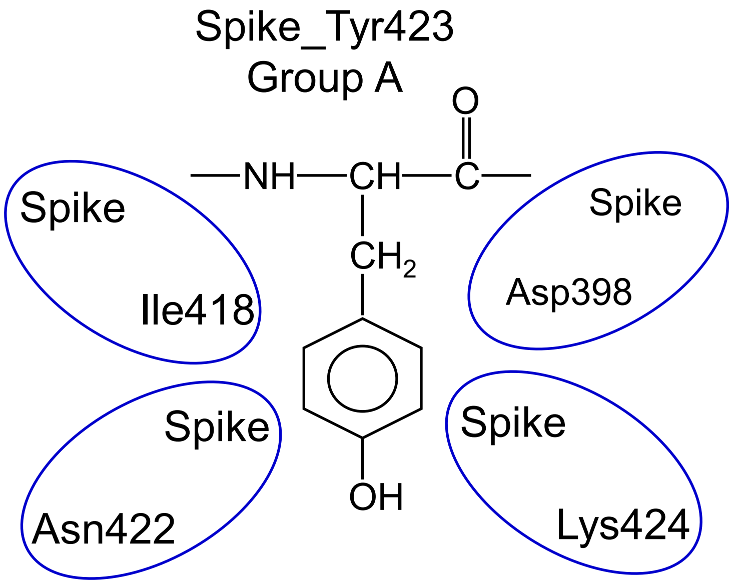

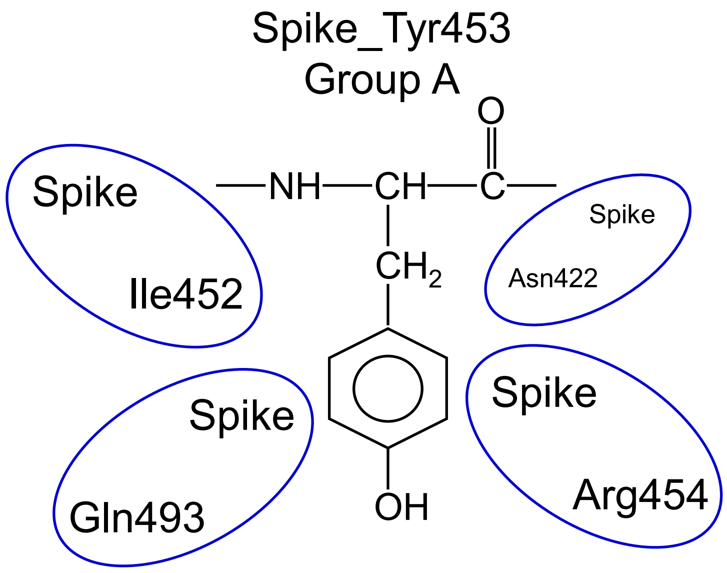

Supplementary Figure 78

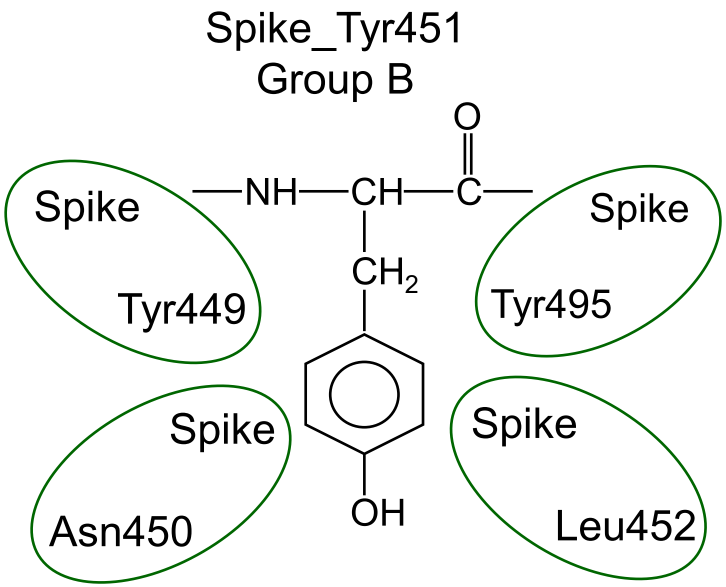

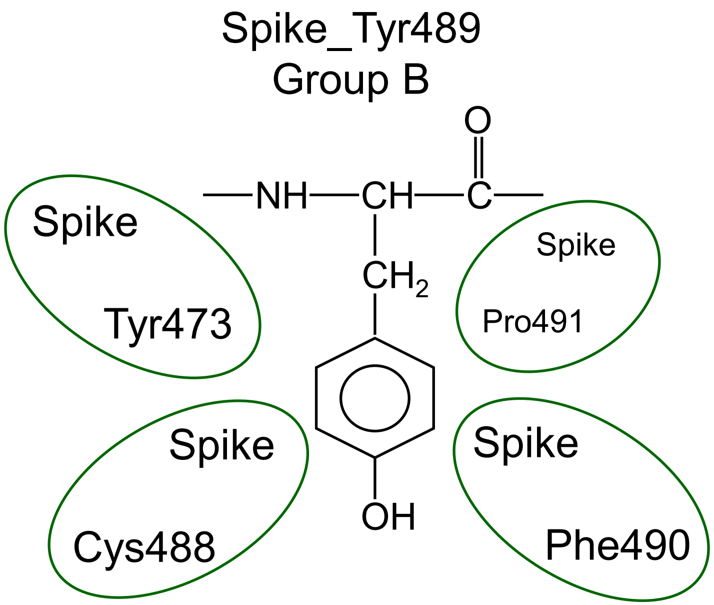

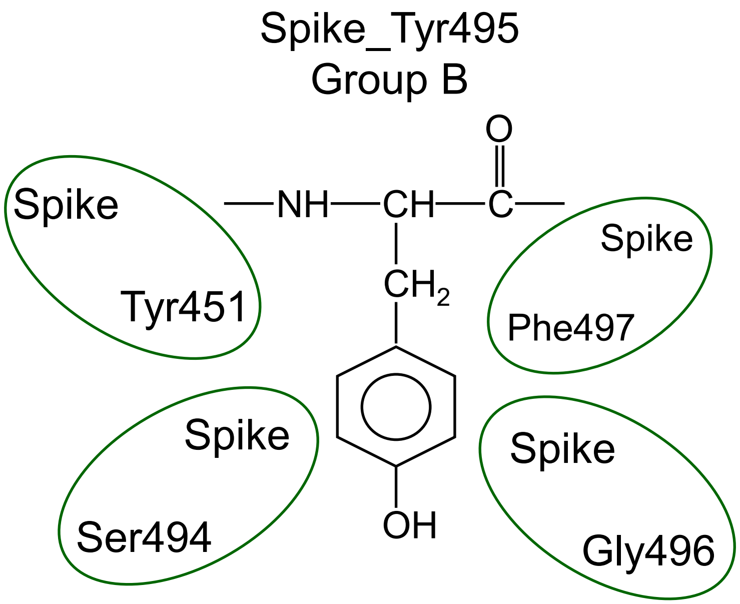

Supplementary Table 1. sample_lilly_s_cr3022_mix1

Spikes	Number of Amino Acids	Number of Distinct Keys	Number of Total Keys	Number of Distinct Common Keys	Number of Total Common Keys	Number of Distinct Specific Keys
Spike_7A93	190	442448	1125180	146480	567636	13986
Spike_ACE2_7A98	190	444695	1125180	146480	567191	12003
Spike_CR3022_6W41	190	445059	1125180	146480	597639	1913
Spike_CR3022_6ZLR	190	444074	1125180	146480	606847	896
Spike_CR3022_BG4_25_7M6D	190	444888	1125180	146480	599250	2211
Spike_CR3022_C099_7R8L	190	443875	1125180	146480	606774	1156
Spike_CR3022_CC12_1_6XC3	190	443556	1125180	146480	610223	402
Spike_CR3022_CC12_3_6XC7	190	443567	1125180	146480	607239	640
Spike_CR3022_CV05_163_7LOP	190	442570	1125180	146480	610053	900
Spike_CR3022_CV2_1169_7QEZ	190	443162	1125180	146480	597011	2864
Spike_CR3022_NB_D4_6YZ7	190	444821	1125180	146480	598325	2304
Spike_CR3022_NB_D4_6Z2M	190	443532	1125180	146480	606214	1266
Spike_CR3022_NB_H11_H4_6ZH9	190	443313	1125180	146480	601744	1893
Spike_CC12_1_6XC2	190	435857	1125180	146480	589795	7210
Spike_CC12_3_6XC4	190	443486	1125180	146480	605802	1324

Supplementary_File_1

protein	chain_1_mab	chain_2_spike	group
7KMG	A	C	SH
8D8Q	H	A	SH
7M6D	A	C	SH
6ZH9	A	C	SH
6ZLR	B	A	SH
7LOP	B	A	SH
6YZ7	B	A	SH
7R8L	C	E	SH
6XC7	H	A	SH
6W41	H	C	SH
6XC3	H	C	SH
6Z2M	H	E	SH
6YLA	H	E	SH
6YM0	H	E	SH
7CJF	A	C	SH
7KMH	A	C	SH
7KMI	A	C	SH
7OR9	A	E	SH
7PS1	A	E	SH
7NX6	A	E	SH
7NX7	A	E	SH
7NX8	A	E	SH
7NX9	A	E	SH
7NXA	A	E	SH
7NXB	A	E	SH
6XC3	B	C	SH
7C01	H	A	SH
7S4S	H	A	SH
7S5P	H	A	SH
6XC2	H	A	SH
6XC4	H	A	SH
7QF0	H	A	SH
7KFV	H	A	SH
7KFW	H	A	SH
7KFX	H	A	SH
7KFY	H	A	SH
7JMO	H	A	SH
7KN6	H	A	SH
7KN7	H	A	SH
7NP1	H	A	SH
7URQ	H	A	SH
7URS	H	A	SH
7BZ5	H	A	SH
7MZF	H	A	SH
7MZG	H	A	SH

7MZN	H	A	SH
7N3I	H	C	SH
7M6D	H	C	SH
7Q0G	H	E	SH
7B3O	H	E	SH
7R8L	H	E	SH
7E8M	H	E	SH
7CDI	H	E	SH
6XE1	H	E	SH
7NEG	H	E	SH
7NEH	H	E	SH
7BEI	H	E	SH
7BEJ	H	E	SH
7BEK	H	E	SH
7CHO	H	E	SH
7CHP	H	E	SH
7CHS	H	E	SH
7QNY	H	E	SH
7PQY	H	E	SH
7PQZ	H	E	SH
7E5Y	H	R	SH
7CH4	H	R	SH
7CH5	H	R	SH
7CHB	H	R	SH
7CHC	H	R	SH
7CHE	H	R	SH
7CHF	H	R	SH
7EJY	H	R	SH
7EJZ	H	R	SH
7EK0	H	R	SH
7DJZ	A	C	SH
7DK0	A	C	SH
7DK2	A	C	SH
7MMO	A	C	SH
7DPM	A	C	SH
7Q0G	A	E	SH
7Q0H	A	E	SH
7BEN	A	E	SH
7BEP	A	E	SH
7QNW	A	E	SH
7QNX	A	E	SH
7QNY	A	E	SH
7PQZ	A	E	SH
7S0B	A	F	SH
7L7E	A	G	SH
7PS2	A	G	SH
7BEL	A	R	SH

7BEO	A	R	SH
7CHC	A	R	SH
7CHE	A	R	SH
7CHF	A	R	SH
7ORB	A	R	SH
7S5Q	B	A	SH
7S5R	B	A	SH
7D6I	B	A	SH
7K9Z	B	E	SH
6XC7	C	A	SH
7QF1	C	A	SH
7WUE	C	A	SH
7F7E	C	E	SH
7E7Y	C	E	SH
7PS6	C	E	SH
7L7E	C	G	SH
7JX3	C	R	SH
7QEZ	E	A	SH
7BEH	H	E	SH
7M7W	E	R	SH
7MZJ	F	A	SH
7MZK	F	A	SH
7PRY	F	E	SH
7U2D	H	A	SH
7U2E	H	A	SH
7N4J	H	A	SH
7N4L	H	A	SH
7L5B	H	A	SH
7E5O	H	A	SH
7S5Q	H	A	SH
7S5R	H	A	SH
7EAM	H	A	SH
7EAN	H	A	SH
7SBU	H	A	SH
7SD5	H	A	SH
7MF1	H	A	SH
7SI2	H	A	SH
6XKP	H	A	SH
6XKQ	H	A	SH
7KLG	H	A	SH
7KLH	H	A	SH
7CM4	H	A	SH
7LM8	H	A	SH
7LM9	H	A	SH
7JMP	H	A	SH
7JMW	H	A	SH
7KN3	H	A	SH

7KN4	H	A	SH
7BNV	H	A	SH
7LOP	H	A	SH
7LQ7	H	A	SH
7MSQ	H	A	SH
7MZI	H	A	SH
7MZJ	H	A	SH
7MZK	H	A	SH
7MZL	H	A	SH
7MZM	H	A	SH
7WPH	H	B	SH
7N4I	H	C	SH
7ORA	H	C	SH
7KZB	H	C	SH
7Q0H	H	E	SH
7L7D	H	E	SH
7K9Z	H	E	SH
6ZCZ	H	E	SH
7CDJ	H	E	SH
7BEN	H	E	SH
6ZER	H	E	SH
7FJS	H	E	SH
7QNW	H	E	SH
7QNX	H	E	SH
7PR0	H	E	SH
7OR9	H	E	SH
7PRY	H	E	SH
7PRZ	H	E	SH
7PS0	H	E	SH
7PS4	H	E	SH
7PS5	H	E	SH
7PS6	H	E	SH
7BWJ	H	E	SH
7NX6	H	E	SH
7NX7	H	E	SH
7NX8	H	E	SH
7NX9	H	E	SH
7NXA	H	E	SH
7NXB	H	E	SH
7MZH	H	E	SH
7B0B	H	F	SH
7PS2	H	G	SH
7TN0	H	I	SH
7L0N	H	R	SH
7Z0X	H	R	SH
7Z0Y	H	R	SH
7M3I	H	R	SH

7E3O	H	R	SH
7R6W	H	R	SH
7M7W	H	R	SH
7BEO	H	R	SH
7ORB	H	R	SH
7PS7	H	R	SH
7JX3	H	R	SH
7VYR	H	R	SH
7TP3	H	Z	SH
7TP4	H	Z	SH
7LM8	M	A	SH
7LQ7	P	A	SH
7L0N	A	R	SH
7R6W	A	R	SH
7R6X	A	R	SH
7JX3	A	R	SH
7TN0	B	I	SH
7BEP	H	E	SH
8D8Q	C	A	SH
7KMG	B	C	SL
8D8Q	L	A	SL
6XC3	A	C	SL
7K9Z	A	E	SL
7QF1	B	A	SL
7M6D	B	C	SL
6ZH9	B	C	SL
7CJF	B	C	SL
7DJZ	B	C	SL
7DK0	B	C	SL
7DK2	B	C	SL
7KMH	B	C	SL
7KMI	B	C	SL
7MMO	B	C	SL
7DPM	B	C	SL
7ORA	B	C	SL
7Q0G	B	E	SL
7Q0H	B	E	SL
7BEN	B	E	SL
7QNW	B	E	SL
7QNX	B	E	SL
7QNY	B	E	SL
7PQZ	B	E	SL
7OR9	B	E	SL
7PS1	B	E	SL
7NX6	B	E	SL
7NX7	B	E	SL
7NX8	B	E	SL

7NX9	B	E	SL
7NXA	B	E	SL
7NXB	B	E	SL
7S0B	B	F	SL
7L7E	B	G	SL
7PS2	B	G	SL
7BEL	B	R	SL
7BEO	B	R	SL
7CHC	B	R	SL
7CHE	B	R	SL
7CHF	B	R	SL
7ORB	B	R	SL
7S5Q	C	A	SL
7S5R	C	A	SL
7D6I	C	A	SL
6ZLR	C	A	SL
7LOP	C	A	SL
6YZ7	C	A	SL
6XC7	D	A	SL
7WUE	D	A	SL
7E7Y	D	E	SL
7R8L	D	E	SL
7PS6	D	E	SL
7JX3	D	R	SL
7MZJ	E	A	SL
7MZK	E	A	SL
7BEH	L	E	SL
7QEZ	F	A	SL
7M7W	F	R	SL
7PRY	G	E	SL
7C01	L	A	SL
7U2D	L	A	SL
7U2E	L	A	SL
7N4J	L	A	SL
7N4L	L	A	SL
7S4S	L	A	SL
7L5B	L	A	SL
7E5O	L	A	SL
7S5P	L	A	SL
7S5R	L	A	SL
7EAM	L	A	SL
7EAN	L	A	SL
7SBU	L	A	SL
6XC2	L	A	SL
6XC4	L	A	SL
6XC7	L	A	SL
7SD5	L	A	SL

7QF0	L	A	SL
7MF1	L	A	SL
7KFV	L	A	SL
7KFW	L	A	SL
7KFX	L	A	SL
7KFY	L	A	SL
7SI2	L	A	SL
6XKP	L	A	SL
6XKQ	L	A	SL
7KLG	L	A	SL
7KLH	L	A	SL
7CM4	L	A	SL
7LM8	L	A	SL
7LM9	L	A	SL
7JMO	L	A	SL
7JMP	L	A	SL
7JMW	L	A	SL
7KN3	L	A	SL
7KN4	L	A	SL
7KN6	L	A	SL
7KN7	L	A	SL
7BNV	L	A	SL
7LOP	L	A	SL
7NP1	L	A	SL
7LQ7	L	A	SL
7URQ	L	A	SL
7URS	L	A	SL
7MSQ	L	A	SL
7BZ5	L	A	SL
7MZF	L	A	SL
7MZG	L	A	SL
7MZI	L	A	SL
7MZJ	L	A	SL
7MZK	L	A	SL
7MZL	L	A	SL
7MZN	L	A	SL
7RKU	L	B	SL
7WPH	L	B	SL
7N3I	L	C	SL
6W41	L	C	SL
7N4I	L	C	SL
7M6D	L	C	SL
6XC3	L	C	SL
7ORA	L	C	SL
7KZB	L	C	SL
7Q0G	L	E	SL
7Q0H	L	E	SL

6Z2M	L	E	SL
7B3O	L	E	SL
7L7D	L	E	SL
7F7E	L	E	SL
7R8L	L	E	SL
7E8M	L	E	SL
7K9Z	L	E	SL
6ZCZ	L	E	SL
7CDI	L	E	SL
7CDJ	L	E	SL
6XE1	L	E	SL
7NEG	L	E	SL
7NEH	L	E	SL
7BEI	L	E	SL
7BEJ	L	E	SL
7BEK	L	E	SL
7BEN	L	E	SL
6ZER	L	E	SL
7CHO	L	E	SL
7CHP	L	E	SL
7CHS	L	E	SL
7FJS	L	E	SL
6YLA	L	E	SL
6YM0	L	E	SL
7QNW	L	E	SL
7QNX	L	E	SL
7QNY	L	E	SL
7PQY	L	E	SL
7PQZ	L	E	SL
7PR0	L	E	SL
7OR9	L	E	SL
7PRY	L	E	SL
7PRZ	L	E	SL
7PS0	L	E	SL
7PS4	L	E	SL
7PS5	L	E	SL
7PS6	L	E	SL
7BWJ	L	E	SL
7NX6	L	E	SL
7NX7	L	E	SL
7NX8	L	E	SL
7NX9	L	E	SL
7NXA	L	E	SL
7NXB	L	E	SL
7MZH	L	E	SL
7B0B	L	F	SL
7PS2	L	G	SL

7L0N	L	R	SL
7Z0X	L	R	SL
7Z0Y	L	R	SL
7M3I	L	R	SL
7E3O	L	R	SL
7E5Y	L	R	SL
7R6W	L	R	SL
7M7W	L	R	SL
7BEO	L	R	SL
7CH4	L	R	SL
7CH5	L	R	SL
7CHB	L	R	SL
7CHC	L	R	SL
7CHE	L	R	SL
7CHF	L	R	SL
7EJY	L	R	SL
7EJZ	L	R	SL
7EK0	L	R	SL
7ORB	L	R	SL
7PS7	L	R	SL
7JX3	L	R	SL
7VYR	L	R	SL
7TP3	L	Z	SL
7TP4	L	Z	SL
7LM8	N	A	SL
7LQ7	Q	A	SL
7BEP	B	E	SL
7TN0	G	I	SL
7TN0	A	I	SL
7BEP	L	E	SL
7JX3	B	R	SL
7L0N	B	R	SL
7R6W	B	R	SL
7R6X	B	R	SL
8D8Q	B	A	SL

Supplementary_File_1A

**protein**	**chain**	**group**
6W41	C	Spike
6XC2	A	Spike
6XC3	C	Spike
6XC4	A	Spike
6XC7	A	Spike
6XE1	E	Spike
6XKP	A	Spike
6XKQ	A	Spike
6YLA	E	Spike
6YM0	E	Spike
6YZ7	A	Spike
6Z2M	E	Spike
6ZCZ	E	Spike
6ZER	E	Spike
6ZH9	C	Spike
6ZLR	A	Spike
7B0B	F	Spike
7B3O	E	Spike
7BEH	E	Spike
7BEI	E	Spike
7BEJ	E	Spike
7BEK	E	Spike
7BEL	R	Spike
7BEN	E	Spike
7BEO	R	Spike
7BEP	E	Spike
7BNV	A	Spike
7BWJ	E	Spike
7BZ5	A	Spike
7C01	A	Spike
7CDI	E	Spike
7CDJ	E	Spike
7CH4	R	Spike
7CH5	R	Spike
7CHB	R	Spike
7CHC	R	Spike
7CHE	R	Spike
7CHF	R	Spike
7CHO	E	Spike
7CHP	E	Spike
7CHS	E	Spike
7CJF	C	Spike
7CM4	A	Spike
7D6I	A	Spike
7DJZ	C	Spike

7DK0	C	Spike
7DK2	C	Spike
7DPM	C	Spike
7E3O	R	Spike
7E5O	A	Spike
7E5Y	R	Spike
7E7Y	E	Spike
7E8M	E	Spike
7EAM	A	Spike
7EAN	A	Spike
7EJY	R	Spike
7EJZ	R	Spike
7EK0	R	Spike
7F7E	E	Spike
7FJS	E	Spike
7JMO	A	Spike
7JMP	A	Spike
7JMW	A	Spike
7JX3	R	Spike
7K8M	E	Spike
7K9Z	E	Spike
7KFV	A	Spike
7KFW	A	Spike
7KFX	A	Spike
7KFY	A	Spike
7KLG	A	Spike
7KLH	A	Spike
7KMG	C	Spike
7KMH	C	Spike
7KMI	C	Spike
7KN3	A	Spike
7KN4	A	Spike
7KN6	A	Spike
7KN7	A	Spike
7KZB	C	Spike
7L0N	R	Spike
7L5B	A	Spike
7L7D	E	Spike
7L7E	G	Spike
7LM8	A	Spike
7LM9	A	Spike
7LOP	A	Spike
7LQ7	A	Spike
7M3I	R	Spike
7M6D	C	Spike
7M7W	R	Spike
7MF1	A	Spike

7MMO	C	Spike
7MSQ	A	Spike
7MZF	A	Spike
7MZG	A	Spike
7MZH	E	Spike
7MZI	A	Spike
7MZJ	A	Spike
7MZK	A	Spike
7MZL	A	Spike
7MZM	A	Spike
7MZN	A	Spike
7N3I	C	Spike
7N4I	C	Spike
7N4J	A	Spike
7N4L	A	Spike
7NEG	E	Spike
7NEH	E	Spike
7NP1	A	Spike
7NX6	E	Spike
7NX7	E	Spike
7NX8	E	Spike
7NX9	E	Spike
7NXA	E	Spike
7NXB	E	Spike
7OR9	E	Spike
7ORA	C	Spike
7ORB	R	Spike
7PQY	E	Spike
7PQZ	E	Spike
7PR0	E	Spike
7PRY	E	Spike
7PRZ	E	Spike
7PS0	E	Spike
7PS1	E	Spike
7PS2	G	Spike
7PS4	E	Spike
7PS5	E	Spike
7PS6	E	Spike
7PS7	R	Spike
7Q0G	E	Spike
7Q0H	E	Spike
7QEZ	A	Spike
7QF0	A	Spike
7QF1	A	Spike
7QNW	E	Spike
7QNX	E	Spike
7QNY	E	Spike

7R6W	R	Spike
7R6X	R	Spike
7R8L	E	Spike
7RKU	B	Spike
7S0B	F	Spike
7S4S	A	Spike
7S5P	A	Spike
7S5Q	A	Spike
7S5R	A	Spike
7SBU	A	Spike
7SD5	A	Spike
7SI2	A	Spike
7TN0	I	Spike
7TP3	Z	Spike
7TP4	Z	Spike
7U2D	A	Spike
7U2E	A	Spike
7URQ	A	Spike
7URS	A	Spike
7VYR	R	Spike
7WPH	B	Spike
7WUE	A	Spike
7Z0X	R	Spike
7Z0Y	R	Spike
7A93	A	Spike
7A98	A	Spike
7W92	B	Spike
7WVO	B	Spike
6W41	H	Heavy
6XC2	H	Heavy
6XC3	H	Heavy
6XC3	B	Heavy
6XC4	H	Heavy
6XC7	H	Heavy
6XC7	C	Heavy
6XCA	H	Heavy
6XE1	H	Heavy
6XKP	H	Heavy
6XKQ	H	Heavy
6YLA	H	Heavy
6YM0	H	Heavy
6YZ7	B	Heavy
6Z2M	H	Heavy
6ZCZ	H	Heavy
6ZER	H	Heavy
6ZH9	A	Heavy
6ZLR	B	Heavy

7B0B	H	Heavy
7B3O	H	Heavy
7BEH	H	Heavy
7BEI	H	Heavy
7BEJ	H	Heavy
7BEK	H	Heavy
7BEL	A	Heavy
7BEN	A	Heavy
7BEN	H	Heavy
7BEO	A	Heavy
7BEO	H	Heavy
7BEP	H	Heavy
7BEP	A	Heavy
7BNV	H	Heavy
7BWJ	H	Heavy
7BZ5	H	Heavy
7C01	H	Heavy
7CDI	H	Heavy
7CDJ	H	Heavy
7CH4	H	Heavy
7CH5	H	Heavy
7CHB	H	Heavy
7CHC	H	Heavy
7CHC	A	Heavy
7CHE	A	Heavy
7CHE	H	Heavy
7CHF	H	Heavy
7CHF	A	Heavy
7CHO	H	Heavy
7CHP	H	Heavy
7CHS	H	Heavy
7CJF	A	Heavy
7CM4	H	Heavy
7D6I	B	Heavy
7DJZ	A	Heavy
7DK0	A	Heavy
7DK2	A	Heavy
7DPM	A	Heavy
7E3O	H	Heavy
7E5O	H	Heavy
7E5Y	H	Heavy
7E7Y	C	Heavy
7E8M	H	Heavy
7EAM	H	Heavy
7EAN	H	Heavy
7EJY	H	Heavy
7EJZ	H	Heavy

7EK0	H	Heavy
7F7E	C	Heavy
7FJS	H	Heavy
7JMO	H	Heavy
7JMP	H	Heavy
7JMW	H	Heavy
7JX3	A	Heavy
7JX3	H	Heavy
7JX3	C	Heavy
7JXC	H	Heavy
7JXE	H	Heavy
7K3Q	H	Heavy
7K8M	A	Heavy
7K8N	A	Heavy
7K8O	H	Heavy
7K8P	H	Heavy
7K8Q	H	Heavy
7K8R	H	Heavy
7K9Z	H	Heavy
7K9Z	B	Heavy
7KFV	H	Heavy
7KFW	H	Heavy
7KFX	H	Heavy
7KFY	H	Heavy
7KLG	H	Heavy
7KLH	H	Heavy
7KMG	A	Heavy
7KMH	A	Heavy
7KMI	A	Heavy
7KN3	H	Heavy
7KN4	H	Heavy
7KN6	H	Heavy
7KN7	H	Heavy
7KZA	H	Heavy
7KZB	H	Heavy
7KZC	H	Heavy
7L0N	A	Heavy
7L0N	H	Heavy
7L5B	H	Heavy
7L7D	H	Heavy
7L7E	A	Heavy
7L7E	C	Heavy
7LKA	H	Heavy
7LM8	M	Heavy
7LM8	H	Heavy
7LM9	H	Heavy
7LOP	H	Heavy

7LOP	B	Heavy
7LQ7	H	Heavy
7LQ7	P	Heavy
7M3I	H	Heavy
7M6D	A	Heavy
7M6D	H	Heavy
7M7W	H	Heavy
7M7W	E	Heavy
7MF1	H	Heavy
7MMO	A	Heavy
7MSQ	H	Heavy
7MZF	H	Heavy
7MZG	H	Heavy
7MZH	H	Heavy
7MZI	H	Heavy
7MZJ	F	Heavy
7MZJ	H	Heavy
7MZK	F	Heavy
7MZK	H	Heavy
7MZL	H	Heavy
7MZM	H	Heavy
7MZN	H	Heavy
7N3E	H	Heavy
7N3F	H	Heavy
7N3G	H	Heavy
7N3H	H	Heavy
7N3I	H	Heavy
7N4I	H	Heavy
7N4J	H	Heavy
7N4L	H	Heavy
7NEG	H	Heavy
7NEH	H	Heavy
7NP1	H	Heavy
7NX6	H	Heavy
7NX6	A	Heavy
7NX7	H	Heavy
7NX7	A	Heavy
7NX8	H	Heavy
7NX8	A	Heavy
7NX9	H	Heavy
7NX9	A	Heavy
7NXA	H	Heavy
7NXA	A	Heavy
7NXB	H	Heavy
7NXB	A	Heavy
7OR9	H	Heavy
7OR9	A	Heavy

7ORA	A	Heavy
7ORA	H	Heavy
7ORB	A	Heavy
7ORB	H	Heavy
7PQY	H	Heavy
7PQZ	H	Heavy
7PQZ	A	Heavy
7PR0	H	Heavy
7PRY	F	Heavy
7PRY	H	Heavy
7PRZ	H	Heavy
7PS0	H	Heavy
7PS1	A	Heavy
7PS2	H	Heavy
7PS2	A	Heavy
7PS3	H	Heavy
7PS4	H	Heavy
7PS5	H	Heavy
7PS6	H	Heavy
7PS6	C	Heavy
7PS7	H	Heavy
7Q0G	H	Heavy
7Q0G	A	Heavy
7Q0H	H	Heavy
7Q0H	A	Heavy
7QEZ	E	Heavy
7QF0	H	Heavy
7QF1	C	Heavy
7QNW	H	Heavy
7QNW	A	Heavy
7QNX	H	Heavy
7QNX	A	Heavy
7QNY	A	Heavy
7QNY	H	Heavy
7R6W	A	Heavy
7R6W	H	Heavy
7R6X	A	Heavy
7R8L	C	Heavy
7R8L	H	Heavy
7RKU	H	Heavy
7S0B	A	Heavy
7S4S	H	Heavy
7S5P	H	Heavy
7S5Q	B	Heavy
7S5Q	H	Heavy
7S5R	B	Heavy
7S5R	H	Heavy

7SBU	H	Heavy
7SD5	H	Heavy
7SI2	H	Heavy
7TN0	B	Heavy
7TN0	H	Heavy
7TP3	H	Heavy
7TP4	H	Heavy
7U2D	H	Heavy
7U2E	H	Heavy
7URQ	H	Heavy
7URS	H	Heavy
7VYR	H	Heavy
7WPH	H	Heavy
7WUE	C	Heavy
7Z0X	H	Heavy
7Z0Y	H	Heavy
6W41	L	Light
6XC2	L	Light
6XC3	L	Light
6XC3	A	Light
6XC4	L	Light
6XC7	L	Light
6XC7	D	Light
6XCA	L	Light
6XE1	L	Light
6XKP	L	Light
6XKQ	L	Light
6YLA	L	Light
6YM0	L	Light
6YZ7	C	Light
6Z2M	L	Light
6ZCZ	L	Light
6ZER	L	Light
6ZH9	B	Light
6ZLR	C	Light
7B0B	L	Light
7B3O	L	Light
7BEH	L	Light
7BEI	L	Light
7BEJ	L	Light
7BEK	L	Light
7BEL	B	Light
7BEN	B	Light
7BEN	L	Light
7BEO	B	Light
7BEO	L	Light
7BEP	L	Light

7BEP	B	Light
7BNV	L	Light
7BWJ	L	Light
7BZ5	L	Light
7C01	L	Light
7CDI	L	Light
7CDJ	L	Light
7CH4	L	Light
7CH5	L	Light
7CHB	L	Light
7CHC	L	Light
7CHC	B	Light
7CHE	B	Light
7CHE	L	Light
7CHF	L	Light
7CHF	B	Light
7CHO	L	Light
7CHP	L	Light
7CHS	L	Light
7CJF	B	Light
7CM4	L	Light
7D6I	C	Light
7DJZ	B	Light
7DK0	B	Light
7DK2	B	Light
7DPM	B	Light
7E3O	L	Light
7E5O	L	Light
7E5Y	L	Light
7E7Y	D	Light
7E8M	L	Light
7EAM	L	Light
7EAN	L	Light
7EJY	L	Light
7EJZ	L	Light
7EK0	L	Light
7F7E	L	Light
7FJS	L	Light
7JMO	L	Light
7JMP	L	Light
7JMW	L	Light
7JX3	B	Light
7JX3	L	Light
7JX3	D	Light
7JXC	L	Light
7JXE	L	Light
7K3Q	L	Light

7K8M	B	Light
7K8N	B	Light
7K8O	L	Light
7K8P	L	Light
7K8Q	L	Light
7K8R	L	Light
7K9Z	L	Light
7K9Z	A	Light
7KFV	L	Light
7KFW	L	Light
7KFX	L	Light
7KFY	L	Light
7KLG	L	Light
7KLH	L	Light
7KMG	B	Light
7KMH	B	Light
7KMI	B	Light
7KN3	L	Light
7KN4	L	Light
7KN6	L	Light
7KN7	L	Light
7KZA	L	Light
7KZB	L	Light
7KZC	L	Light
7L0N	B	Light
7L0N	L	Light
7L5B	L	Light
7L7D	L	Light
7L7E	B	Light
7L7E	D	Light
7LKA	L	Light
7LM8	N	Light
7LM8	L	Light
7LM9	L	Light
7LOP	L	Light
7LOP	C	Light
7LQ7	L	Light
7LQ7	Q	Light
7M3I	L	Light
7M6D	B	Light
7M6D	L	Light
7M7W	L	Light
7M7W	F	Light
7MF1	L	Light
7MMO	B	Light
7MSQ	L	Light
7MZF	L	Light

7MZG	L	Light
7MZH	L	Light
7MZI	L	Light
7MZJ	E	Light
7MZJ	L	Light
7MZK	E	Light
7MZK	L	Light
7MZL	L	Light
7MZM	L	Light
7MZN	L	Light
7N3E	L	Light
7N3F	L	Light
7N3G	L	Light
7N3H	L	Light
7N3I	L	Light
7N4I	L	Light
7N4J	L	Light
7N4L	L	Light
7NEG	L	Light
7NEH	L	Light
7NP1	L	Light
7NX6	L	Light
7NX6	B	Light
7NX7	L	Light
7NX7	B	Light
7NX8	L	Light
7NX8	B	Light
7NX9	L	Light
7NX9	B	Light
7NXA	L	Light
7NXA	B	Light
7NXB	L	Light
7NXB	B	Light
7OR9	L	Light
7OR9	B	Light
7ORA	B	Light
7ORA	L	Light
7ORB	B	Light
7ORB	L	Light
7PQY	L	Light
7PQZ	L	Light
7PQZ	B	Light
7PR0	L	Light
7PRY	G	Light
7PRY	L	Light
7PRZ	L	Light
7PS0	L	Light

7PS1	B	Light
7PS2	L	Light
7PS2	B	Light
7PS3	L	Light
7PS4	L	Light
7PS5	L	Light
7PS6	L	Light
7PS6	D	Light
7PS7	L	Light
7Q0G	L	Light
7Q0G	B	Light
7Q0H	L	Light
7Q0H	B	Light
7QEZ	F	Light
7QF0	L	Light
7QF1	B	Light
7QNW	L	Light
7QNW	B	Light
7QNX	L	Light
7QNX	B	Light
7QNY	B	Light
7QNY	L	Light
7R6W	B	Light
7R6W	L	Light
7R6X	B	Light
7R8L	D	Light
7R8L	L	Light
7RKU	L	Light
7S0B	B	Light
7S4S	L	Light
7S5P	L	Light
7S5Q	C	Light
7S5Q	L	Light
7S5R	C	Light
7S5R	L	Light
7SBU	L	Light
7SD5	L	Light
7SI2	L	Light
7TN0	A	Light
7TN0	G	Light
7TP3	L	Light
7TP4	L	Light
7U2D	L	Light
7U2E	L	Light
7URQ	L	Light
7URS	L	Light
7VYR	L	Light

7WPH	L	Light
7WUE	D	Light
7Z0X	L	Light
7Z0Y	L	Light

Supplementary_File_1B

**Number**	**Label_PDB**
0	Light-BDK0
1	Light-DJX3
2	Light-LSD5
3	Light-BQNY
4	Light-LU2D
5	Light-LU2E
6	Light-LCHO
7	Light-LN3E
8	Light-LE3O
9	Light-LKN4
10	Light-LMF1
11	Light-LK8Q
12	Light-LZ0X
13	Light-LL5B
14	Light-LPS0
15	Light-LWPH
16	Light-LXKP
17	Light-LXCA
18	Light-LBWJ
19	Light-LLQ7
20	Light-LEAM
21	Light-LEAN
22	Light-LE5Y
23	Light-LF7E
24	Light-LKZC
25	Light-LVYR
26	Light-LPR0
27	Light-BCHC
28	Light-BCHE
29	Light-BCHF
30	Light-LMZL
31	Light-BOR9
32	Light-BNXB
33	Light-BNX7
34	Light-BNX8
35	Light-BNXA
36	Light-BNX6
37	Light-BNX9
38	Light-LN3H
39	Light-CS5Q
40	Light-CS5R
41	Light-BK8N
42	Light-LN3G
43	Light-LS4S
44	Light-LURQ

45 Light-DXC7
46 Light-LR8L
47 Light-BS0B
48 Light-LMZN
49 Light-LQ0G
50 Light-LN4L
51 Light-BKMI
52 Light-LPS3
53 Light-LPQY
54 Light-LPQZ
55 Light-BKMH
56 Light-LS5Q
57 Light-LS5R
58 Light-LLM8
59 Light-LJMW
60 Light-QLQ7
61 Light-LMZJ
62 Light-LJX3
63 Light-GTN0
64 Light-LL0N
65 Light-BQNW
66 Light-BQNX
67 Light-BBEP
68 Light-LBEJ
69 Light-LCHE
70 Light-LCHF
71 Light-LCDJ
72 Light-NLM8
73 Light-LKFV
74 Light-LKFW
75 Light-LMSQ
76 Light-LPS6
77 Light-LQ0H
78 Light-LCHP
79 Light-LKZB
80 Light-AXC3
81 Light-LC01
82 Light-LNP1
83 Light-LNXA
84 Light-LNX6
85 Light-LNX9
86 Light-AK9Z
87 Light-BM6D
88 Light-FQEZ
89 Light-LXC3
90 Light-DR8L
91 Light-LXC7

92 Light-CZLR
93 Light-CLOP
94 Light-LW41
95 Light-LYM0
96 Light-LYLA
97 Light-LZ2M
98 Light-BZH9
99 Light-CYZ7
100 Light-LPRZ
101 Light-LMZK
102 Light-BPS2
103 Light-LKN3
104 Light-DL7E
105 Light-DWUE
106 Light-LFJS
107 Light-LPS2
108 Light-LBNV
109 Light-LM6D
110 Light-LTP3
111 Light-BQ0G
112 Light-BQ0H
113 Light-LURS
114 Light-LXE1
115 Light-BR6W
116 Light-BPS1
117 Light-BK8M
118 Light-LCDI
119 Light-LE8M
120 Light-LKN6
121 Light-LN3I
122 Light-LJMO
123 Light-LPS5
124 Light-BDJZ
125 Light-LLOP
126 Light-LPRY
127 Light-BJX3
128 Light-LBEP
129 Light-BR6X
130 Light-ATN0
131 Light-BL0N
132 Light-LSBU
133 Light-LORA
134 Light-LORB
135 Light-LBEN
136 Light-LBEO
137 Light-LL7D
138 Light-BL7E

139 Light-LB0B
140 Light-LK3Q
141 Light-LMZM
142 Light-LCH5
143 Light-LCHC
144 Light-LKN7
145 Light-LXC4
146 Light-BBEL
147 Light-BORA
148 Light-GPRY
149 Light-LKLG
150 Light-LKLH
151 Light-LQNY
152 Light-LK8R
153 Light-LRKU
154 Light-LE5O
155 Light-LK9Z
156 Light-LKZA
157 Light-BQF1
158 Light-LLM9
159 Light-BKMG
160 Light-LK8O
161 Light-LNX8
162 Light-LNX7
163 Light-LNXB
164 Light-BORB
165 Light-BBEN
166 Light-BBEO
167 Light-BDK2
168 Light-LLKA
169 Light-BDPM
170 Light-LK8P
171 Light-LOR9
172 Light-LXC2
173 Light-LMZF
174 Light-LCH4
175 Light-LBEK
176 Light-LQF0
177 Light-LBEI
178 Light-LNEG
179 Light-LNEH
180 Light-LCHS
181 Light-LCHB
182 Light-LB3O
183 Light-LBZ5
184 Light-BCJF
185 Light-LKFX

186 Light-LKFY
187 Light-LEJY
188 Light-LEJZ
189 Light-LEK0
190 Light-LMZG
191 Light-LN4I
192 Light-LSI2
193 Light-LQNW
194 Light-LQNX
195 Light-LZCZ
196 Light-LZER
197 Heavy-HEAM
198 Heavy-HEAN
199 Light-BMMO
200 Light-DE7Y
201 Light-DPS6
202 Light-BPQZ
203 Light-LN3F
204 Light-LPS4
205 Light-LJXE
206 Light-LR6W
207 Light-EMZJ
208 Light-LCM4
209 Light-LM3I
210 Light-LPS7
211 Light-LS5P
212 Light-FM7W
213 Light-LBEH
214 Light-LXKQ
215 Light-CD6I
216 Light-LJXC
217 Light-LJMP
218 Light-LTP4
219 Light-LZ0Y
220 Light-LMZH
221 Light-EMZK
222 Light-LMZI
223 Light-LM7W
224 Light-LN4J
225 Heavy-AMMO
226 Heavy-HCM4
227 Heavy-HL5B
228 Heavy-HMZL
229 Heavy-HWPH
230 Heavy-HMSQ
231 Heavy-HKN4
232 Heavy-HSD5

233 Heavy-HBWJ
234 Heavy-AQNW
235 Heavy-AQNX
236 Heavy-HPS0
237 Heavy-HPS6
238 Heavy-HQ0H
239 Heavy-HE5Y
240 Heavy-HXKP
241 Heavy-HJX3
242 Heavy-HL0N
243 Heavy-HTN0
244 Heavy-HKZB
245 Heavy-ABEP
246 Heavy-CL7E
247 Heavy-HMZJ
248 Heavy-HN4L
249 Heavy-HCDJ
250 Heavy-HE3O
251 Heavy-HNXA
252 Heavy-HNX6
253 Heavy-HNX9
254 Heavy-ACHC
255 Heavy-ACHE
256 Heavy-ACHF
257 Heavy-AQNY
258 Heavy-HU2D
259 Heavy-HU2E
260 Heavy-HZ0X
261 Heavy-HKFV
262 Heavy-HKFW
263 Heavy-HPQY
264 Heavy-HPQZ
265 Heavy-AKMH
266 Heavy-AOR9
267 Heavy-ANXA
268 Heavy-ANX6
269 Heavy-ANX9
270 Heavy-ANXB
271 Heavy-ANX7
272 Heavy-ANX8
273 Heavy-HXCA
274 Heavy-HN3H
275 Heavy-AKMI
276 Heavy-HMZN
277 Heavy-BXC3
278 Heavy-HNP1
279 Heavy-HCHE

280 Heavy-HCHF
281 Heavy-HCHO
282 Heavy-HC01
283 Heavy-CXC7
284 Heavy-HCHP
285 Heavy-AK8N
286 Heavy-HN3G
287 Heavy-HQ0G
288 Heavy-HR8L
289 Heavy-AS0B
290 Heavy-HBEJ
291 Heavy-HS4S
292 Heavy-HURQ
293 Heavy-CWUE
294 Heavy-HFJS
295 Heavy-HPS3
296 Heavy-HLM8
297 Heavy-HS5Q
298 Heavy-HS5R
299 Heavy-HJMW
300 Heavy-PLQ7
301 Heavy-BS5Q
302 Heavy-BS5R
303 Heavy-HLQ7
304 Heavy-HMF1
305 Heavy-EM7W
306 Heavy-HPS4
307 Heavy-HPS2
308 Heavy-HN3F
309 Heavy-HN4I
310 Heavy-HK8P
311 Heavy-MLM8
312 Heavy-BYZ7
313 Heavy-HYLA
314 Heavy-HYM0
315 Heavy-AZH9
316 Heavy-HZ2M
317 Heavy-HKZA
318 Heavy-EQEZ
319 Heavy-AM6D
320 Heavy-HW41
321 Heavy-BZLR
322 Heavy-HXC3
323 Heavy-CR8L
324 Heavy-BLOP
325 Heavy-HXC7
326 Heavy-HN3E

327 Heavy-HBNV
328 Heavy-HLM9
329 Heavy-HM3I
330 Heavy-HN4J
331 Heavy-HPS7
332 Heavy-HPRY
333 Heavy-HRKU
334 Heavy-BD6I
335 Heavy-HJMP
336 Heavy-HS5P
337 Heavy-HTP4
338 Heavy-HZ0Y
339 Heavy-HJXC
340 Heavy-FMZK
341 Heavy-HMZI
342 Heavy-CE7Y
343 Heavy-APQZ
344 Heavy-FMZJ
345 Heavy-HM7W
346 Heavy-HXKQ
347 Heavy-HM6D
348 Heavy-HTP3
349 Heavy-ABEL
350 Heavy-AORA
351 Heavy-FPRY
352 Heavy-AORB
353 Heavy-ABEN
354 Heavy-ABEO
355 Heavy-HPRZ
356 Heavy-APS2
357 Heavy-HK8O
358 Heavy-HNXB
359 Heavy-HNX8
360 Heavy-HNX7
361 Heavy-HQNW
362 Heavy-HQNX
363 Heavy-HSI2
364 Heavy-HZCZ
365 Heavy-HZER
366 Heavy-HE5O
367 Heavy-ADK2
368 Heavy-HLKA
369 Heavy-HQNY
370 Heavy-HPR0
371 Heavy-ADPM
372 Heavy-CF7E
373 Heavy-HKN3

374 Heavy-AK8M
375 Heavy-HKN6
376 Heavy-HXC2
377 Heavy-APS1
378 Heavy-HCDI
379 Heavy-HE8M
380 Heavy-HXE1
381 Heavy-HCHB
382 Heavy-HB3O
383 Heavy-HBZ5
384 Heavy-HCHS
385 Heavy-HN3I
386 Heavy-HMZF
387 Heavy-ACJF
388 Heavy-HKFX
389 Heavy-HKFY
390 Heavy-HBEI
391 Heavy-HBEK
392 Heavy-HMZG
393 Heavy-HNEG
394 Heavy-HNEH
395 Heavy-HURS
396 Heavy-HJMO
397 Heavy-HQF0
398 Heavy-HCH4
399 Heavy-HEK0
400 Heavy-HEJY
401 Heavy-HEJZ
402 Heavy-CJX3
403 Heavy-HK8R
404 Heavy-HKN7
405 Heavy-HXC4
406 Heavy-HCH5
407 Heavy-HCHC
408 Heavy-HMZM
409 Heavy-HKLG
410 Heavy-HKLH
411 Heavy-AKMG
412 Heavy-HPS5
413 Heavy-HK3Q
414 Heavy-HL7D
415 Heavy-HORA
416 Heavy-HORB
417 Heavy-HBEN
418 Heavy-HBEO
419 Heavy-AL7E
420 Heavy-HB0B

421 Heavy-HK9Z
422 Heavy-HKZC
423 Heavy-BK9Z
424 Heavy-HVYR
425 Heavy-CQF1
426 Heavy-HSBU
427 Heavy-AQ0G
428 Heavy-AQ0H
429 Heavy-ADJZ
430 Heavy-ADK0
431 Heavy-HBEH
432 Heavy-HMZH
433 Heavy-HOR9
434 Heavy-CPS6
435 Heavy-HJXE
436 Heavy-HR6W
437 Heavy-HK8Q
438 Heavy-AJX3
439 Heavy-AR6W
440 Heavy-AL0N
441 Heavy-HBEP
442 Heavy-AR6X
443 Heavy-BTN0
444 Heavy-HLOP
445 Heavy-HMZK
446 Spike-6XKQ
447 Spike-7LM9
448 Spike-7L5B
449 Spike-7JMP
450 Spike-7NP1
451 Spike-7A93
452 Spike-7A98
453 Spike-7M3I
454 Spike-7CHP
455 Spike-7E3O
456 Spike-7QNW
457 Spike-7TN0
458 Spike-7PR0
459 Spike-7PRZ
460 Spike-7PS2
461 Spike-7N4L
462 Spike-6XC2
463 Spike-7E5Y
464 Spike-7EAM
465 Spike-7M7W
466 Spike-7MZJ
467 Spike-7DK2

468 Spike-7Z0Y
469 Spike-7K9Z
470 Spike-7PS6
471 Spike-7CHE
472 Spike-7CHF
473 Spike-7KN6
474 Spike-7KN7
475 Spike-7CH5
476 Spike-7CHC
477 Spike-7QNX
478 Spike-7QNY
479 Spike-7K8M
480 Spike-7B3O
481 Spike-7NEG
482 Spike-7JX3
483 Spike-7L0N
484 Spike-7QEZ
485 Spike-7FJS
486 Spike-7PS4
487 Spike-7Q0G
488 Spike-7CDJ
489 Spike-6XE1
490 Spike-7KLG
491 Spike-7KLH
492 Spike-7WUE
493 Spike-7N4I
494 Spike-7N4J
495 Spike-7BNV
496 Spike-7CM4
497 Spike-7F7E
498 Spike-7DPM
499 Spike-7KMI
500 Spike-7D6I
501 Spike-7S0B
502 Spike-7MMO
503 Spike-7SI2
504 Spike-7CHO
505 Spike-7R6X
506 Spike-7S5Q
507 Spike-7S5R
508 Spike-6W41
509 Spike-7M6D
510 Spike-6ZH9
511 Spike-7MSQ
512 Spike-6YZ7
513 Spike-6ZCZ
514 Spike-6ZER

515 Spike-6Z2M
516 Spike-6ZLR
517 Spike-6YLA
518 Spike-6YM0
519 Spike-7KMG
520 Spike-7MZM
521 Spike-7TP4
522 Spike-7DK0
523 Spike-7E7Y
524 Spike-7ORA
525 Spike-7BEL
526 Spike-7PRY
527 Spike-7KZB
528 Spike-7EJY
529 Spike-7EJZ
530 Spike-6XKP
531 Spike-7RKU
532 Spike-7LQ7
533 Spike-7U2D
534 Spike-7U2E
535 Spike-7MZL
536 Spike-7L7D
537 Spike-7BWJ
538 Spike-7WPH
539 Spike-7SBU
540 Spike-7BEP
541 Spike-7Q0H
542 Spike-7PS0
543 Spike-7PS5
544 Spike-7R6W
545 Spike-7QF1
546 Spike-7LOP
547 Spike-7TP3
548 Spike-7MZI
549 Spike-7MZK
550 Spike-7DJZ
551 Spike-7KMH
552 Spike-7MF1
553 Spike-7EAN
554 Spike-7Z0X
555 Spike-7JMW
556 Spike-7KN3
557 Spike-7LM8
558 Spike-7SD5
559 Spike-7S5P
560 Spike-7B0B
561 Spike-7L7E

562 Spike-7EK0
563 Spike-7BEH
564 Spike-7MZH
565 Spike-7S4S
566 Spike-7URQ
567 Spike-7URS
568 Spike-7CH4
569 Spike-7CHB
570 Spike-7C01
571 Spike-7E8M
572 Spike-7BZ5
573 Spike-7N3I
574 Spike-7CDI
575 Spike-7CHS
576 Spike-7BEJ
577 Spike-7PQZ
578 Spike-7PQY
579 Spike-7BEI
580 Spike-7BEK
581 Spike-7MZF
582 Spike-7CJF
583 Spike-7MZG
584 Spike-7JMO
585 Spike-7KFW
586 Spike-7KFV
587 Spike-7KFX
588 Spike-7KFY
589 Spike-7NEH
590 Spike-7QF0
591 Spike-7KN4
592 Spike-7PS1
593 Spike-7PS7
594 Spike-7OR9
595 Spike-7NXA
596 Spike-7NXB
597 Spike-7NX7
598 Spike-7NX8
599 Spike-7NX6
600 Spike-7NX9
601 Spike-7MZN
602 Spike-6XC4
603 Spike-7R8L
604 Spike-6XC3
605 Spike-6XC7
606 Spike-7VYR
607 Spike-7E5O
608 Spike-7ORB

609 Spike-7BEN
610 Spike-7BEO
611 Spike-7W92
612 Spike-7WVO

Supplementary File 2

protein	chain_heavy	chain_spike	group
7KMG	A	C	SH
8D8Q	H	A	SH
7M6D	A	C	SH
6ZH9	A	C	SH
6ZLR	B	A	SH
7LOP	B	A	SH
6YZ7	B	A	SH
7R8L	C	E	SH
6XC7	H	A	SH
6W41	H	C	SH
6XC3	H	C	SH
6Z2M	H	E	SH
6YLA	H	E	SH
6YM0	H	E	SH
7CJF	A	C	SH
7KMH	A	C	SH
7KMI	A	C	SH
7OR9	A	E	SH
7PS1	A	E	SH
7NX6	A	E	SH
7NX7	A	E	SH
7NX8	A	E	SH
7NX9	A	E	SH
7NXA	A	E	SH
7NXB	A	E	SH
6XC3	B	C	SH
7C01	H	A	SH
7S4S	H	A	SH
7S5P	H	A	SH
6XC2	H	A	SH
6XC4	H	A	SH
7QF0	H	A	SH
7KFV	H	A	SH
7KFW	H	A	SH
7KFX	H	A	SH
7KFY	H	A	SH
7JMO	H	A	SH
7KN6	H	A	SH
7KN7	H	A	SH
7NP1	H	A	SH
7URQ	H	A	SH
7URS	H	A	SH
7BZ5	H	A	SH
7MZF	H	A	SH
7MZG	H	A	SH

7MZN	H	A	SH
7N3I	H	C	SH
7M6D	H	C	SH
7Q0G	H	E	SH
7B3O	H	E	SH
7R8L	H	E	SH
7E8M	H	E	SH
7CDI	H	E	SH
6XE1	H	E	SH
7NEG	H	E	SH
7NEH	H	E	SH
7BEI	H	E	SH
7BEJ	H	E	SH
7BEK	H	E	SH
7CHO	H	E	SH
7CHP	H	E	SH
7CHS	H	E	SH
7QNY	H	E	SH
7PQY	H	E	SH
7PQZ	H	E	SH
7E5Y	H	R	SH
7CH4	H	R	SH
7CH5	H	R	SH
7CHB	H	R	SH
7CHC	H	R	SH
7CHE	H	R	SH
7CHF	H	R	SH
7EJY	H	R	SH
7EJZ	H	R	SH
7EK0	H	R	SH
7DJZ	A	C	SH
7DK0	A	C	SH
7DK2	A	C	SH
7MMO	A	C	SH
7DPM	A	C	SH
7Q0G	A	E	SH
7Q0H	A	E	SH
7BEN	A	E	SH
7BEP	A	E	SH
7QNW	A	E	SH
7QNX	A	E	SH
7QNY	A	E	SH
7PQZ	A	E	SH
7S0B	A	F	SH
7L7E	A	G	SH
7PS2	A	G	SH
7BEL	A	R	SH

7BEO	A	R	SH
7CHC	A	R	SH
7CHE	A	R	SH
7CHF	A	R	SH
7ORB	A	R	SH
7S5Q	B	A	SH
7S5R	B	A	SH
7D6I	B	A	SH
7K9Z	B	E	SH
6XC7	C	A	SH
7QF1	C	A	SH
7WUE	C	A	SH
7F7E	C	E	SH
7E7Y	C	E	SH
7PS6	C	E	SH
7L7E	C	G	SH
7JX3	C	R	SH
7QEZ	E	A	SH
7BEH	H	E	SH
7M7W	E	R	SH
7MZJ	F	A	SH
7MZK	F	A	SH
7PRY	F	E	SH
7U2D	H	A	SH
7U2E	H	A	SH
7N4J	H	A	SH
7N4L	H	A	SH
7L5B	H	A	SH
7E5O	H	A	SH
7S5Q	H	A	SH
7S5R	H	A	SH
7EAM	H	A	SH
7EAN	H	A	SH
7SBU	H	A	SH
7SD5	H	A	SH
7MF1	H	A	SH
7SI2	H	A	SH
6XKP	H	A	SH
6XKQ	H	A	SH
7KLG	H	A	SH
7KLH	H	A	SH
7CM4	H	A	SH
7LM8	H	A	SH
7LM9	H	A	SH
7JMP	H	A	SH
7JMW	H	A	SH
7KN3	H	A	SH

7KN4	H	A	SH
7BNV	H	A	SH
7LOP	H	A	SH
7LQ7	H	A	SH
7MSQ	H	A	SH
7MZI	H	A	SH
7MZJ	H	A	SH
7MZK	H	A	SH
7MZL	H	A	SH
7MZM	H	A	SH
7WPH	H	B	SH
7N4I	H	C	SH
7ORA	H	C	SH
7KZB	H	C	SH
7Q0H	H	E	SH
7L7D	H	E	SH
7K9Z	H	E	SH
6ZCZ	H	E	SH
7CDJ	H	E	SH
7BEN	H	E	SH
6ZER	H	E	SH
7FJS	H	E	SH
7QNW	H	E	SH
7QNX	H	E	SH
7PR0	H	E	SH
7OR9	H	E	SH
7PRY	H	E	SH
7PRZ	H	E	SH
7PS0	H	E	SH
7PS4	H	E	SH
7PS5	H	E	SH
7PS6	H	E	SH
7BWJ	H	E	SH
7NX6	H	E	SH
7NX7	H	E	SH
7NX8	H	E	SH
7NX9	H	E	SH
7NXA	H	E	SH
7NXB	H	E	SH
7MZH	H	E	SH
7B0B	H	F	SH
7PS2	H	G	SH
7TN0	H	I	SH
7L0N	H	R	SH
7Z0X	H	R	SH
7Z0Y	H	R	SH
7M3I	H	R	SH

7E3O	H	R	SH
7R6W	H	R	SH
7M7W	H	R	SH
7BEO	H	R	SH
7ORB	H	R	SH
7PS7	H	R	SH
7JX3	H	R	SH
7VYR	H	R	SH
7TP3	H	Z	SH
7TP4	H	Z	SH
7LM8	M	A	SH
7LQ7	P	A	SH
7JX3	A	R	SH
7BEP	H	E	SH
7L0N	A	R	SH
7R6W	A	R	SH
7R6X	A	R	SH
7TN0	B	I	SH
8D8Q	C	A	SH
**protein**	**chain_light**	**chain_spike**	**group**
7KMG	B	C	SL
8D8Q	L	A	SL
6XC3	A	C	SL
7K9Z	A	E	SL
7QF1	B	A	SL
7M6D	B	C	SL
6ZH9	B	C	SL
7CJF	B	C	SL
7DJZ	B	C	SL
7DK0	B	C	SL
7DK2	B	C	SL
7KMH	B	C	SL
7KMI	B	C	SL
7MMO	B	C	SL
7DPM	B	C	SL
7ORA	B	C	SL
7Q0G	B	E	SL
7Q0H	B	E	SL
7BEN	B	E	SL
7BEP	B	E	SL
7QNW	B	E	SL
7QNX	B	E	SL
7QNY	B	E	SL
7PQZ	B	E	SL
7OR9	B	E	SL
7PS1	B	E	SL
7NX6	B	E	SL

7NX7	B	E	SL
7NX8	B	E	SL
7NX9	B	E	SL
7NXA	B	E	SL
7NXB	B	E	SL
7S0B	B	F	SL
7L7E	B	G	SL
7PS2	B	G	SL
7BEL	B	R	SL
7BEO	B	R	SL
7CHC	B	R	SL
7CHE	B	R	SL
7CHF	B	R	SL
7ORB	B	R	SL
7S5Q	C	A	SL
7S5R	C	A	SL
7D6I	C	A	SL
6ZLR	C	A	SL
7LOP	C	A	SL
6YZ7	C	A	SL
6XC7	D	A	SL
7WUE	D	A	SL
7E7Y	D	E	SL
7R8L	D	E	SL
7PS6	D	E	SL
7JX3	D	R	SL
7MZJ	E	A	SL
7MZK	E	A	SL
7BEH	L	E	SL
7QEZ	F	A	SL
7M7W	F	R	SL
7PRY	G	E	SL
7C01	L	A	SL
7U2D	L	A	SL
7U2E	L	A	SL
7N4J	L	A	SL
7N4L	L	A	SL
7S4S	L	A	SL
7L5B	L	A	SL
7E5O	L	A	SL
7S5P	L	A	SL
7S5R	L	A	SL
7EAM	L	A	SL
7EAN	L	A	SL
7SBU	L	A	SL
6XC2	L	A	SL
6XC4	L	A	SL

6XC7	L	A	SL
7SD5	L	A	SL
7QF0	L	A	SL
7MF1	L	A	SL
7KFV	L	A	SL
7KFW	L	A	SL
7KFX	L	A	SL
7KFY	L	A	SL
7SI2	L	A	SL
6XKP	L	A	SL
6XKQ	L	A	SL
7KLG	L	A	SL
7KLH	L	A	SL
7CM4	L	A	SL
7LM8	L	A	SL
7LM9	L	A	SL
7JMO	L	A	SL
7JMP	L	A	SL
7JMW	L	A	SL
7KN3	L	A	SL
7KN4	L	A	SL
7KN6	L	A	SL
7KN7	L	A	SL
7BNV	L	A	SL
7LOP	L	A	SL
7NP1	L	A	SL
7LQ7	L	A	SL
7URQ	L	A	SL
7URS	L	A	SL
7MSQ	L	A	SL
7BZ5	L	A	SL
7MZF	L	A	SL
7MZG	L	A	SL
7MZI	L	A	SL
7MZJ	L	A	SL
7MZK	L	A	SL
7MZL	L	A	SL
7MZN	L	A	SL
7RKU	L	B	SL
7WPH	L	B	SL
7N3I	L	C	SL
6W41	L	C	SL
7N4I	L	C	SL
7M6D	L	C	SL
6XC3	L	C	SL
7ORA	L	C	SL
7KZB	L	C	SL

7Q0G	L	E	SL
7Q0H	L	E	SL
6Z2M	L	E	SL
7B3O	L	E	SL
7L7D	L	E	SL
7F7E	L	E	SL
7R8L	L	E	SL
7E8M	L	E	SL
7K9Z	L	E	SL
6ZCZ	L	E	SL
7CDI	L	E	SL
7CDJ	L	E	SL
6XE1	L	E	SL
7NEG	L	E	SL
7NEH	L	E	SL
7BEI	L	E	SL
7BEJ	L	E	SL
7BEK	L	E	SL
7BEN	L	E	SL
6ZER	L	E	SL
7CHO	L	E	SL
7CHP	L	E	SL
7CHS	L	E	SL
7FJS	L	E	SL
6YLA	L	E	SL
6YM0	L	E	SL
7QNW	L	E	SL
7QNX	L	E	SL
7QNY	L	E	SL
7PQY	L	E	SL
7PQZ	L	E	SL
7PR0	L	E	SL
7OR9	L	E	SL
7PRY	L	E	SL
7PRZ	L	E	SL
7PS0	L	E	SL
7PS4	L	E	SL
7PS5	L	E	SL
7PS6	L	E	SL
7BWJ	L	E	SL
7NX6	L	E	SL
7NX7	L	E	SL
7NX8	L	E	SL
7NX9	L	E	SL
7NXA	L	E	SL
7NXB	L	E	SL
7MZH	L	E	SL

7B0B	L	F	SL
7PS2	L	G	SL
7L0N	L	R	SL
7Z0X	L	R	SL
7Z0Y	L	R	SL
7M3I	L	R	SL
7E3O	L	R	SL
7E5Y	L	R	SL
7R6W	L	R	SL
7M7W	L	R	SL
7BEO	L	R	SL
7CH4	L	R	SL
7CH5	L	R	SL
7CHB	L	R	SL
7CHC	L	R	SL
7CHE	L	R	SL
7CHF	L	R	SL
7EJY	L	R	SL
7EJZ	L	R	SL
7EK0	L	R	SL
7ORB	L	R	SL
7PS7	L	R	SL
7VYR	L	R	SL
7TP3	L	Z	SL
7TP4	L	Z	SL
7LM8	N	A	SL
7LQ7	Q	A	SL
7TN0	G	I	SL
7JX3	L	R	SL
7JX3	B	R	SL
7TN0	A	I	SL
7L0N	B	R	SL
7R6W	B	R	SL
7R6X	B	R	SL
7BEP	L	E	SL
8D8Q	B	A	SL
